# Supplementary material for: Death effector domain-containing protein induces vulnerability to cell cycle inhibition in triple-negative breast cancer
Source: Nat Commun. 2019 Jun 28;10:2860. doi: 10.1038/s41467-019-10743-7 (PMC6599020; doi:10.1038/s41467-019-10743-7)
Supplement: Supplementary file 1 — Supplementary Information [file 41467_2019_10743_MOESM1_ESM.pdf]

Supplementary Information for

Death Effector Domain-Containing Protein Renders Vulnerability to Cell Cycle Inhibition in Triple-Negative Breast Cancer

Ni et al.

Correspondence to: szhang8@nd.edu

This PDF file includes:

Supplementary Figures 1 to 9

Other Supplementary Materials for this manuscript include the following:

Supplementary Data 1 (separate file). Exel file containing top 200 depleted gene list using MAGeCK method (Tab1), the MAGeCK ranking score of the top 200 depleted genes (Tab 2), raw counts (Tab 3), the alteration frequencies of the top 200 depleted genes in cBioportal TNBC clinical samples (Tab 4), and the selection notes for looking for alteration frequencies of top 200 depleted genes in cBioportal TNBC clinical samples (Tab 5).

Supplementary Data 2 (separate file). Exel file containing the list of the cytosol DEDD interacted protein IDs in MDA-MB-468 cells through immunoprecipitation assay and the raw spectral counts. The search is against the UniProt human Database. The FDR cutoff is 1%.

Source Data File (separate file). PPTX file containing the uncropped images of all the western blots presented in the manuscript main figures.

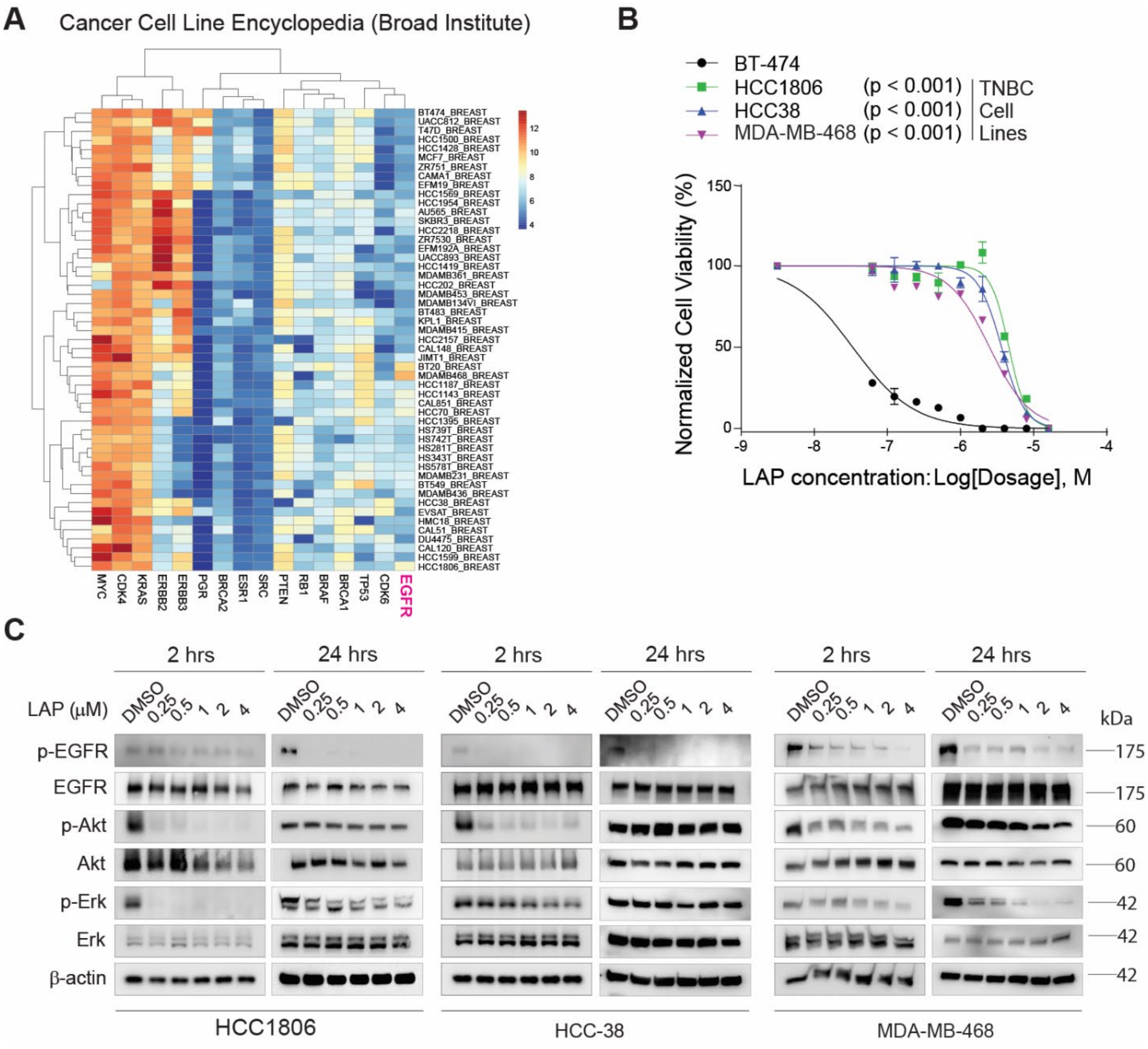

Supplementary Figure 1: EGFR overexpressed TNBCs are resistant to EGFR/HER2 inhibitor. (a) Selective gene expression of TNBC cell lines. mRNA expression levels were extracted from Cancer Cell Line Encyclopedia (Novartis/Broad, Nature 2012, cBioportal). (b) MTT assay showing cell viability of triple-negative and Her2+ breast cancer cell lines under different dosages of Lapatinib (LAP). (c) Immunoblots show EGFR as well as Akt and Erk expression in TNBC cell lines 2 hours and 24 hours post-treatment with LAP. All quantitative data were generated from a minimum of three replicates. P values were derived from one-way ANOVA with multiple comparison test (paired). Error bars represent means ± s.e.m.

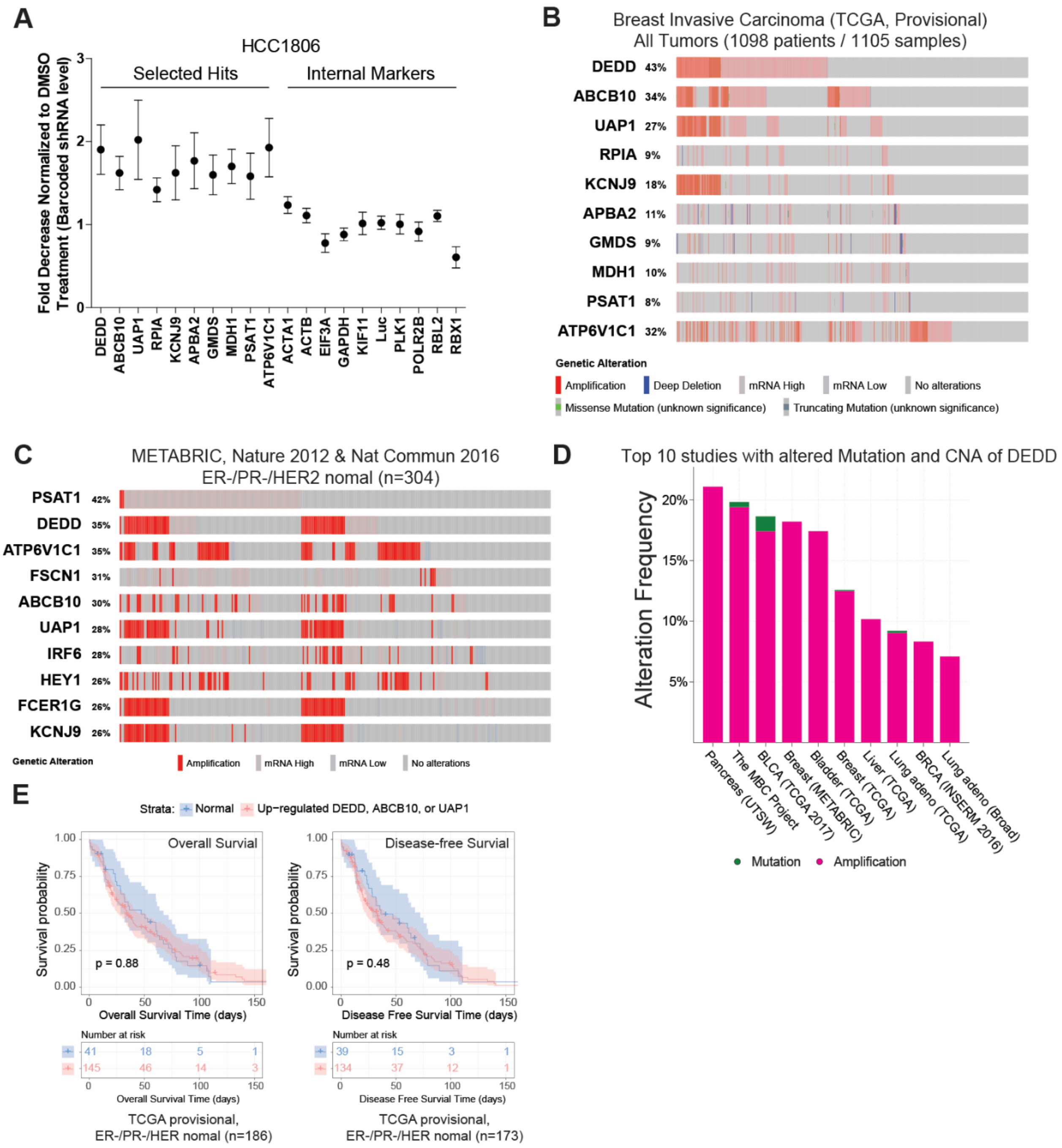

Supplementary Figure 2: The clinical relevance of 1q gene amplification in TNBCs. (a) Fold decrease of Barcoded shRNAs of the 10 upregulated genes (Figure 1c) in the functional screen. (b) Expression of Top 10 genes among 200 drop-out hits that are overexpressed in clinical total breast invasive carcinomas tissues from the Cancer Genome Atlas (TCGA). Z score of mRNA expression data of each tumor was computed by comparing the relative expression of an individual gene and tumor to the gene's expression distribution in all tumors in TCGA breast cancer dataset (reference population). The returned value indicates the number of standard deviations away from the mean of expression in the reference population (Z-score). Tissue samples with mRNA expression level of EXP(Z score) > 2 (e^2 = 7.4) are considered overexpressed. DEDD is overexpressed in 44% of 816 breast cancer patients. Breast Invasive Carcinoma (TCGA, Cell 2015, n=816). (c) Expression of Top 10 genes among 200 drop-out hits that are overexpressed in clinical triple-negative breast cancer tissues from the METABRIC. (d) Genome alteration frequency plot of top 10 cancer studies with DEDD alteration across 164 studies in cBioPortal. (e) Kaplan-Meier overall and disease-free survival analysis of PAM Basal Type Breast Invasive Carcinoma patients with or without up-regulation of DEDD, ABCB10 and UAP1. For a, error bars mean the range fold changes of different shRNAs for one gene.

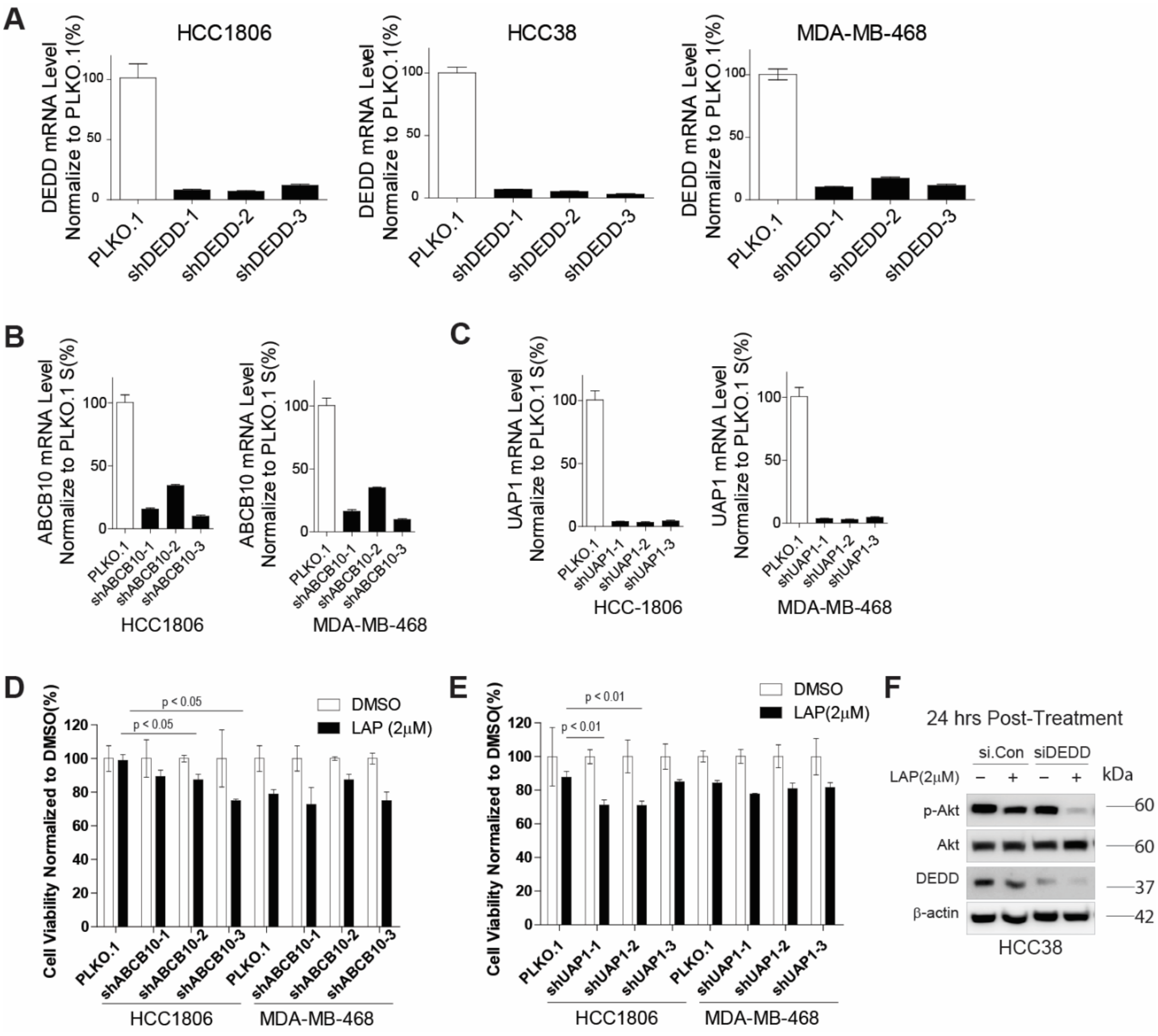

Supplementary Figure 3: Validation of DEDD on EGFR/HER2 inhibitor resistance in TNBCs. (a) Quantitative PCR showing DEDD gene expression in 3 different DEDD knockdown TNBC cell lines (HCC1806, HCC38, MDA-MB-468). (b) Quantitative PCR showing ABCB10 gene expression in 2 different ABCB10 knockdown TNBC cell lines (HCC1806, MDA-MB-468). (c) MTT assay showing cell viability in 2 different ABCB10 knockdown TNBC cells(HCC1806, MDA-MB-468). (d) Quantitative PCR showing UAP1 gene expression in 2 different UAP1 knockdown TNBC cell lines (HCC1806, MDA-MB-468). (e) MTT assay showing cell viability in 2 different UAP1 knockdown TNBC cells(HCC1806, MDA-MB-468). (f) Immunoblots showing Akt signaling changes in HCC38 cells with or without SiDEDD knockdown 24 hours post-treatment of LAP (2μM). Error bars represent means ± s.e.m. All quantitative data were generated from a minimum of three replicates. P values were derived from one-way ANOVA with Dunnett's Multiple Comparison Test comparing different shRNAs to the PLKO.1 group.

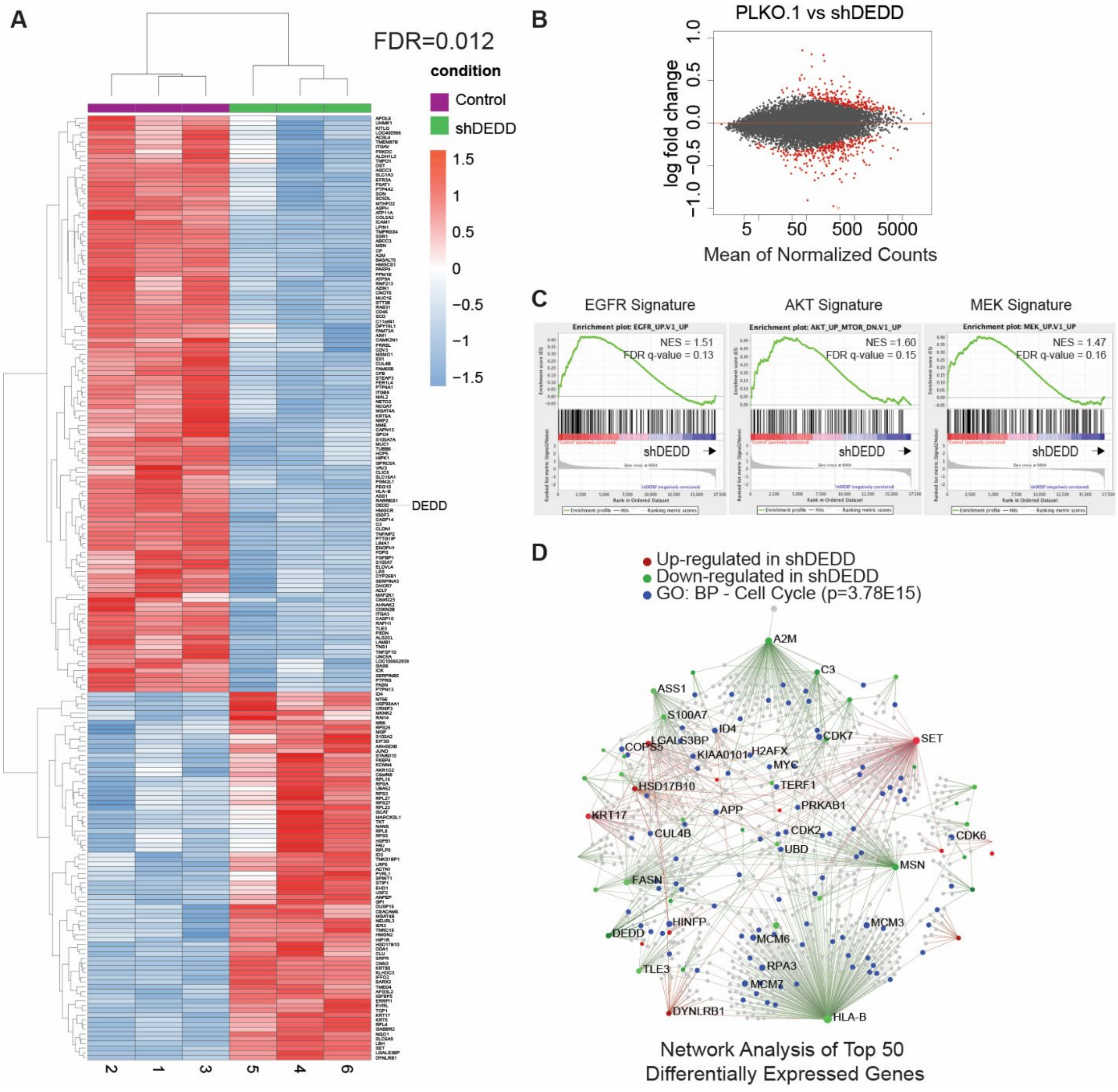

Supplementary Figure 4: DEDD mediates cell cycle related genes in TNBCs. (a) Heatmap showing the top 50 genes that were altered in DEDD knockdown versus PLKO.1 MDA-MB-468 cells. The color scale indicates a log10 ratio of the normalized signal intensities of altered genes.) (b) Log fold change of all differentially expressed genes in MDA-MB-468 cells expressing sh-DEDD RNA or PLKO.1. Red color dots mean selected hits have at least 2 fold change of expression alteration. (c) Genes most altered by DEDD knockdown in TNBC cells are enriched for Achilles cluster genes. Gene set enrichment analysis of Achilles cluster genes in comparison to genes expression in PLKO.1 group. GSEA-supplied p values are given. FDR<0.012 (d) NetworkAnalyst analysis shows that top 50 most differentiated genes in DEDD knockdown TNBC cells are enriched for genes participating in cell cycle regulation network.

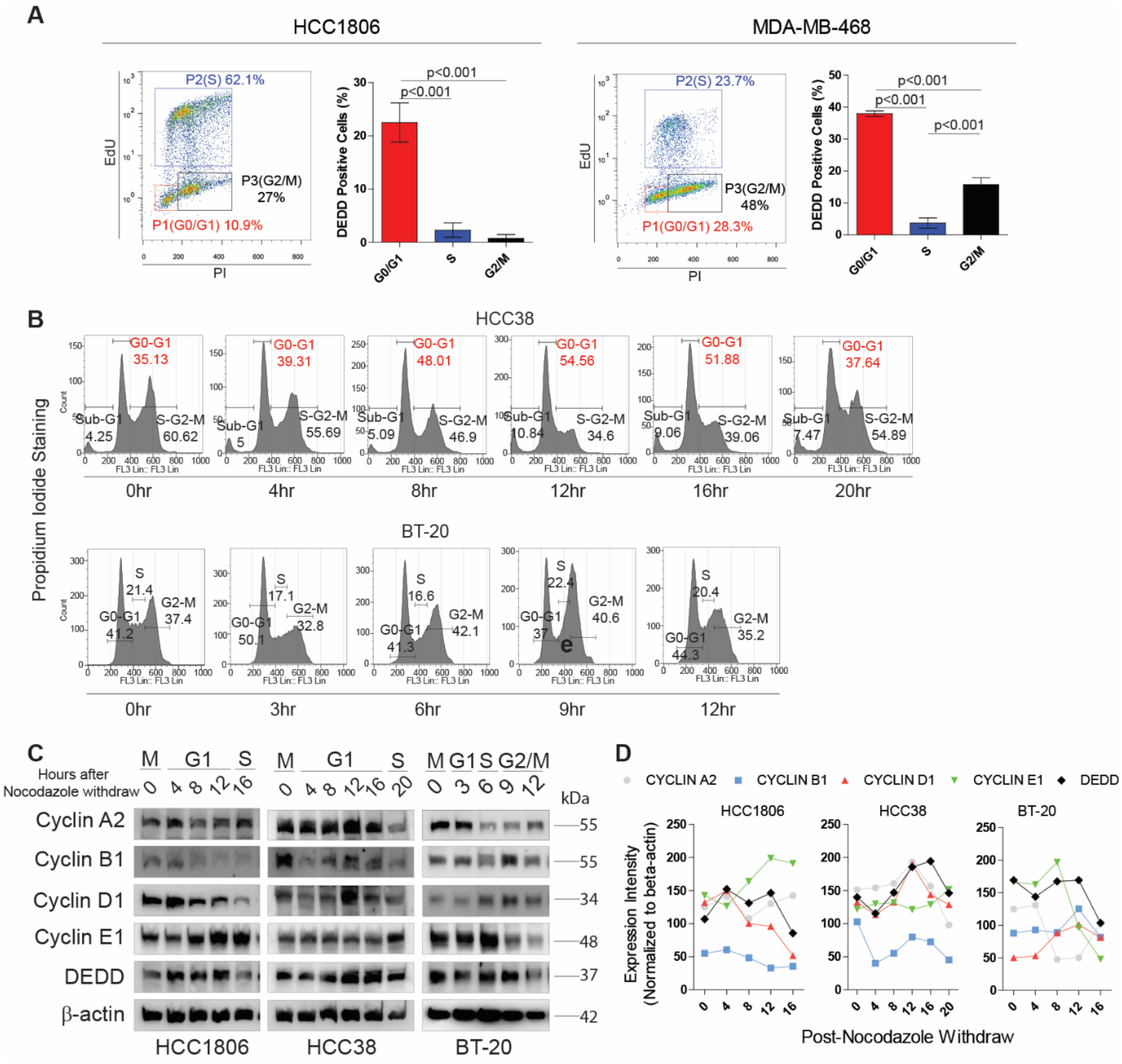

Supplementary Figure 5: DEDD associates with cyclin D1 expression in TNBCs. (a) For each TNBC cell line, Left: Representative flow cytometry biaxial plots of TNBC cell lines (HCC1806, MDA-MB-468) showing cells from different cell cycle phases are enriched for DEDD staining. Right: Quantification of DEDD positive cells in FACS sorted cell populations above. (b) Flow cytometry analysis showing cell cycle population distribution of TNBC cell lines (HCC38, BT-20) at different time points after cells released from Nocodazole treatment (100ng/ml). (c)Immunoblots showing cyclin profiles as well as DEDD expression of TNBC cell lines (HCC1806, HCC38, BT-20) at different time points after cells released from Nocodazole treatment (100ng/ml). (d) Quantification of cyclin expression levels in TNBC cell lines in (c) normalized to beta-actin. All quantitative data were generated from a minimum of three replicates. P values were derived from one-way ANOVA with multiple comparison test. Error bars represent means ± s.e.m.

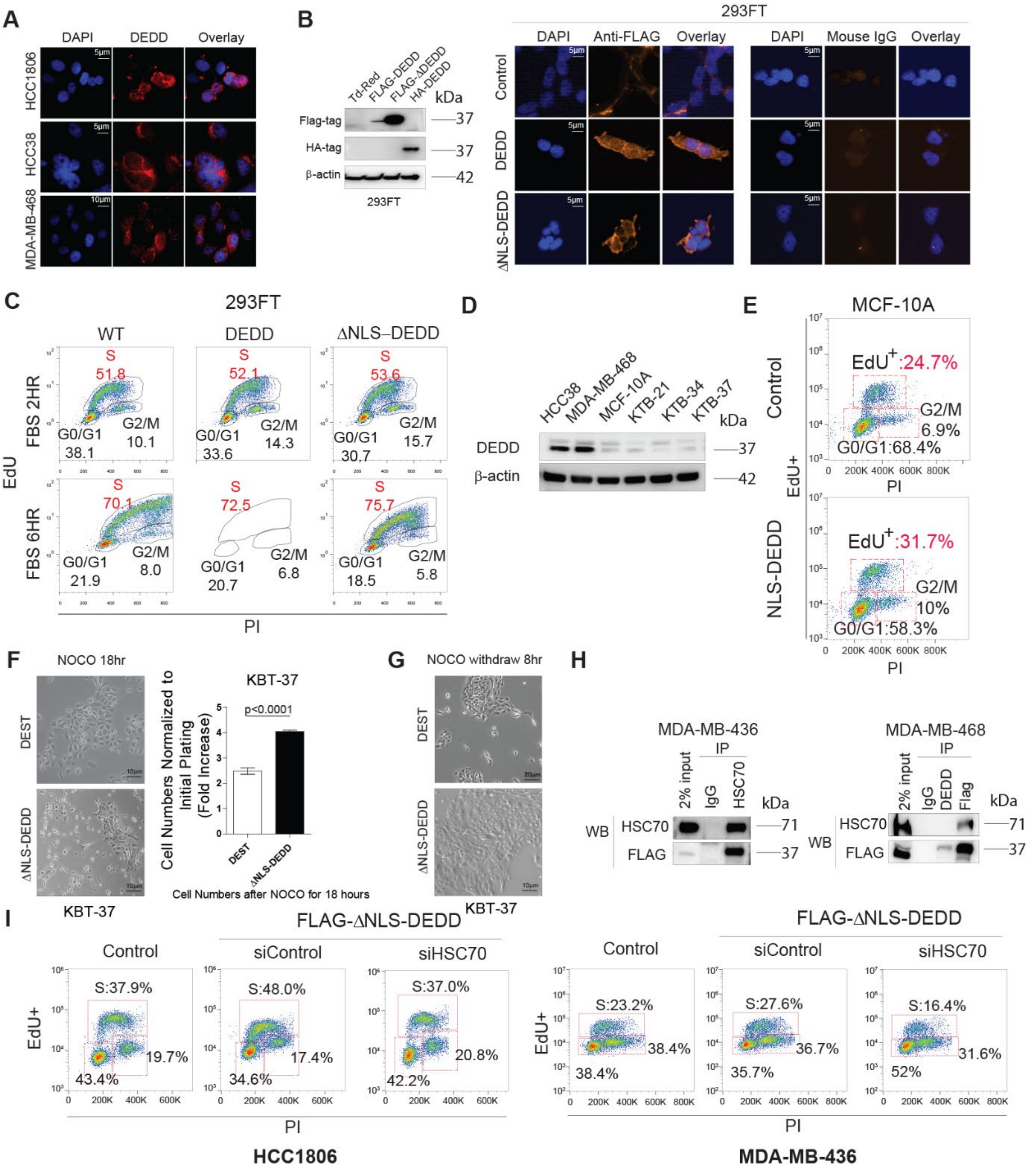

Supplementary Figure 6: Cytosolic DEDD promotes HSC70 dependent G1/S transitions. (a) Representative pictures of Immunofluorescent staining of DEDD in TNBC cell lines. (b) Left: Immunoblotting of Flag-DEDD, Flag-ΔDEDD and HA-DEDD in 293FT cells after transient transfection of indicated plasmids. Right: Immunofluorescent staining of Flag (red) and nuclear (blue) in 293FT cells transient transfected with indicated plasmids. (c) Representative EdU-incorperation flow cytometry biaxial plots show cell cycle population distribution at different time points after abolishing synchronization by FBS in 293FT cells expressing either WT-DEDD or ΔNLS-DEDD. The cells were labeled with EdU and propidium iodide (PI). (d) Western blots showing endogenous DEDD expression. (e) Representative flow biaxial plots of control plasmid or ΔNLS-DEDD overexpressed MCF-10A cells. (f) Representative picture of cell proliferation in control plasmid or ΔNLS-DEDD expressed KTB cells at 0hr of Nocodazole withdraw. Right: quantification of cell numbers in ktb-37 cells with or without nocodazole treatment (noco 18hr). (g) Representative picture of cell proliferation in control plasmid or ΔNLS-DEDD expressed KTB cells 8 hours after nocodazole release. (h) Immunoprecipitation assay showing the interaction between cytosolic DEDD and HSC70 in TNBC cell lines. (i) Representative flow cytometry biaxial plots showing EdU-incorporation of HSC70 knockdown TNBC cells with or without expression of cytosolic DEDD. P values were derived from two-tailed t test. Error bars represent means ± s.e.m.

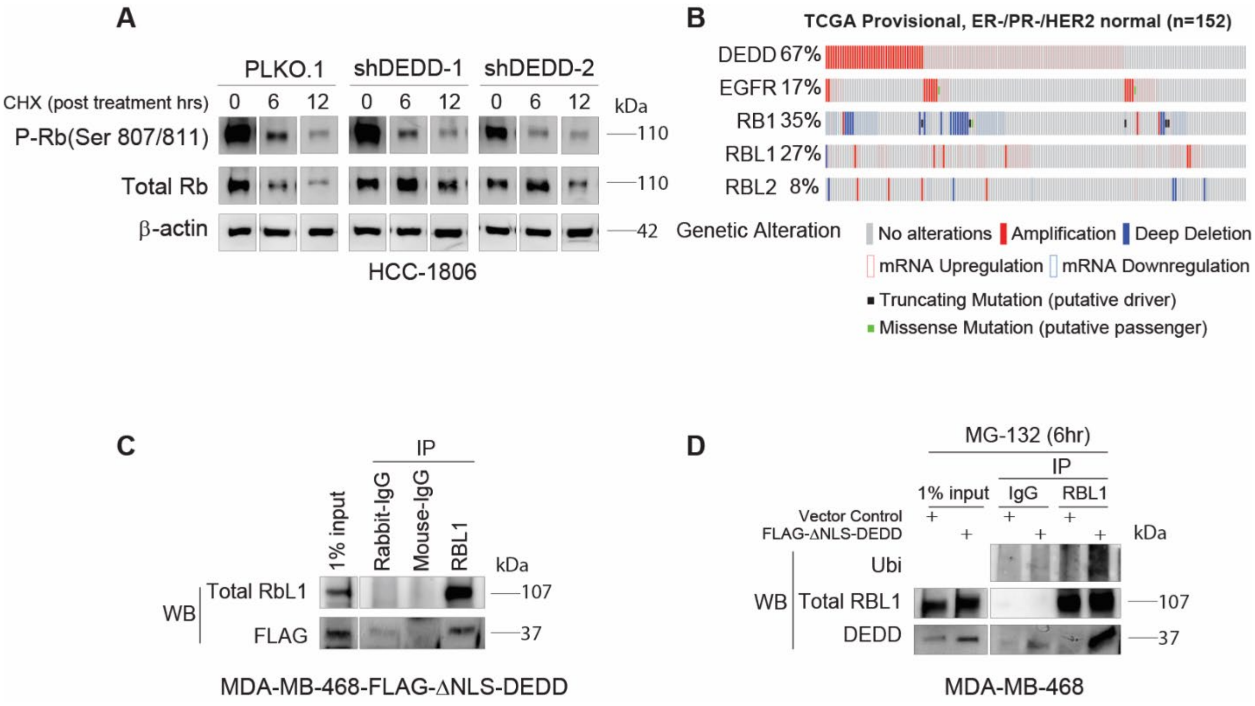

Supplementary Figure 7: Cytosolic DEDD mediates Rb/p107 proteasome degradation in TNBCs. (a) Western blots showing Rb protein expression in DEDD knockdown HCC1806 cells with Cycloheximide (CHX) treatment for indicated hours. (b) Genetic alterations of the Rb family proteins in the Cancer Genome Atlas (TCGA) ER-/PR-/HER2 normal tumors (n=187). (c) Immunoprecipitation assay showing the interaction between cytosolic DEDD and RbL1 protein after overexpressing 3X-Flag-ΔNLS-DEDD in MDA-MB-468 cells. (d) Immunoprecipitation assay showing RbL1 protein ubiquitination level after co-transfecting HA-ubiquitin and 3X-Flag-ΔNLS-DEDD in MDA-MB-468 cells.

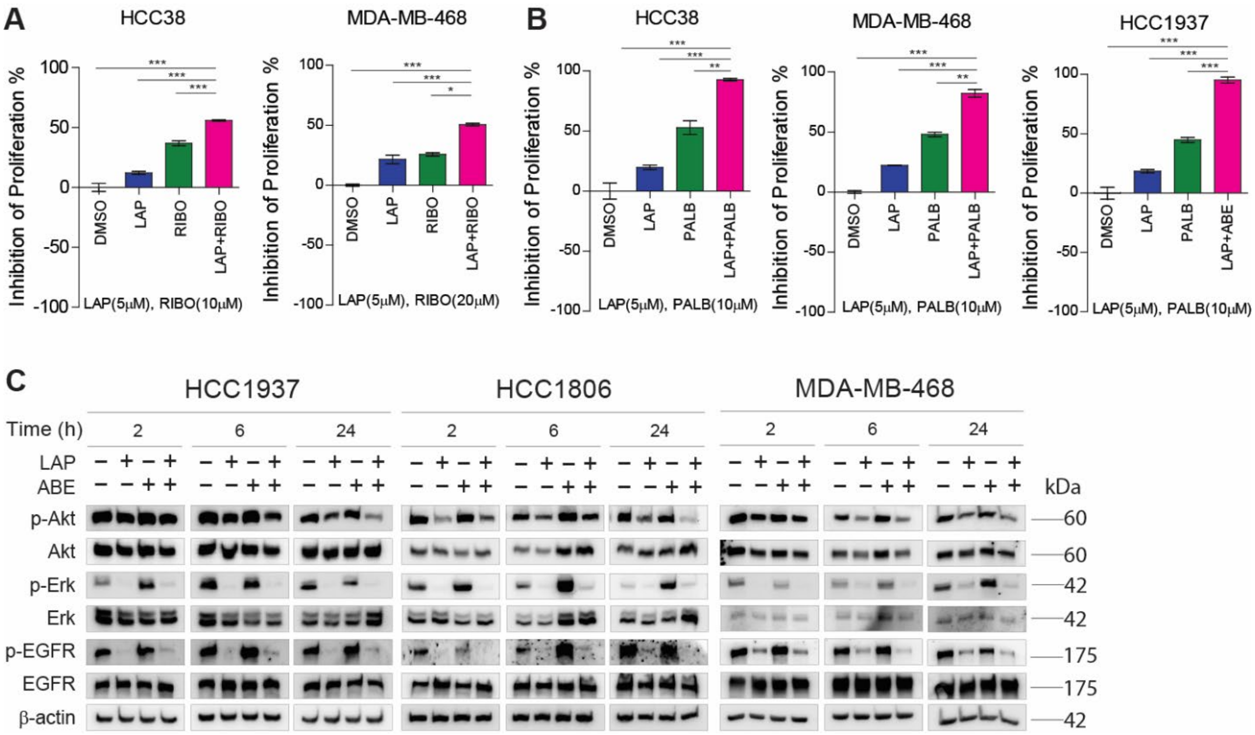

Supplementary Figure 8: CDK4/6 inhibitor synergizes with EGFR inhibitor to suppress TNBC proliferation. (a) MTT assay showing the inhibition of cell proliferation of TNBC cell lines treated with DMSO, LAP, Ribociclib (RIBO) or Combo (LAP+RIBO) for 48 hours. (b) MTT assay showing the inhibition of cell proliferation of TNBC cell lines treated with DMSO, LAP, Palbociclib (PALB) or Combo (LAP+PALB) for 48 hours. (c) Western blots showing downstreamsignaling changes in TNBC cell lines after designated treatment for the indicated time. Error bars represent means ± s.e.m. All quantitative data were generated from a minimum of three replicates. P values were derived from one-way ANOVA with multiple comparison test. Error bars represent means ± s.e.m.

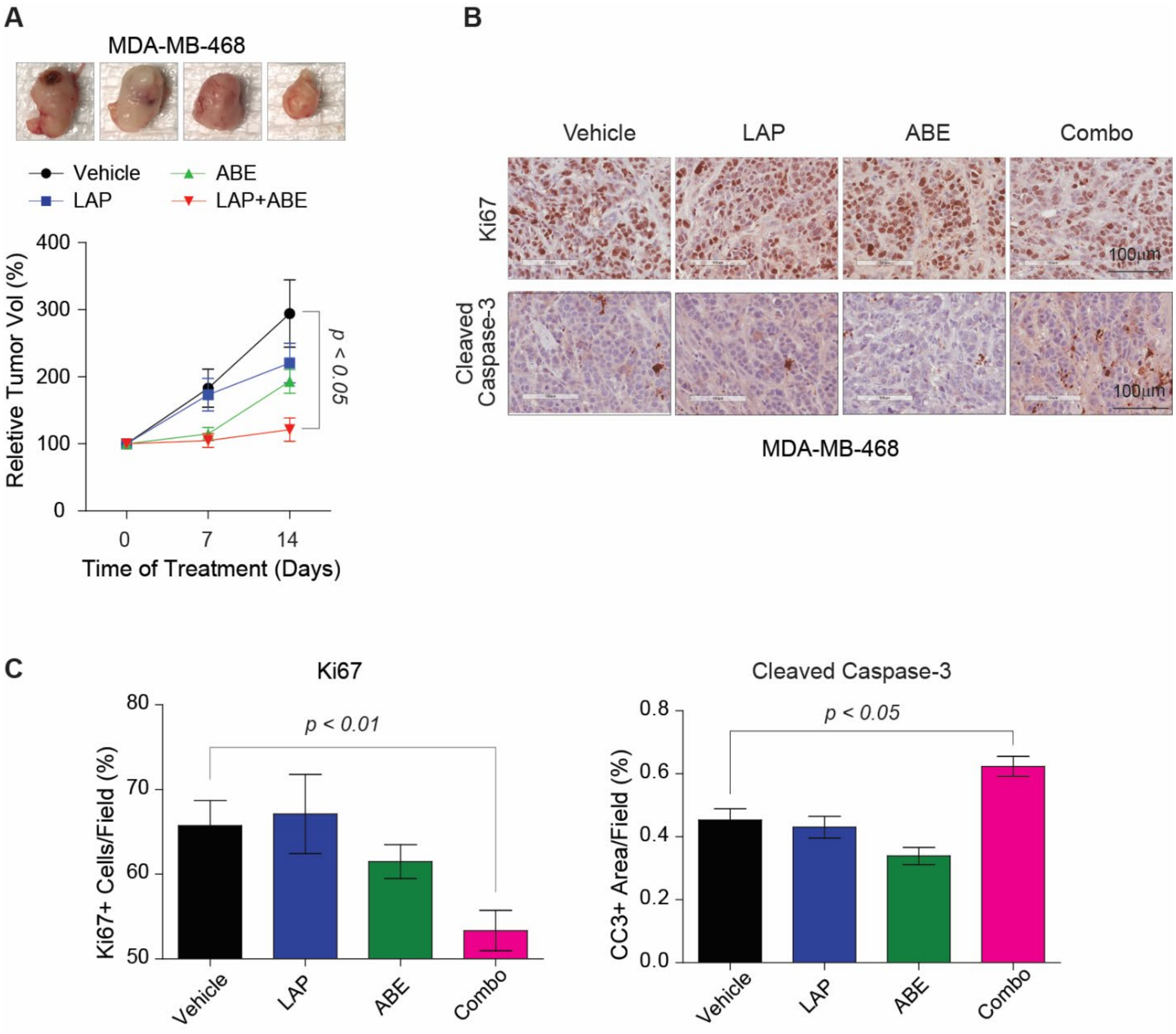

Supplementary Figure 9: CDK4/6 inhibitor synergizes with EGFR inhibitor to suppress Rb deficient TNBC tumor growth. (a) Top: Representative pictures of the MDA-MB-468 tumor xenografts showing the inhibition of tumor progression under different treatment strategies in MDA-MB-468 cells. Bottom: Comparison of MDA-MB-468 xenograft tumor volumes within mice treated with vehicle, LAP (2 mg/per mouse per day), ABE (0.5 mg/per mouse per day) and the Combo treatment. (b) Representative pictures of IHC staining showing Ki67 and cleaved caspase-3 expression in different treatment groups in MDA-MB-468 xenografts. (c) Quantification of IHC staining in panel b. All quantitative data were generated from a minimum of three replicates. P values were derived from one-way ANOVA with multiple comparison test. Error bars represent means ± s.e.m.
