# Supplementary material for: Death effector domain-containing protein induces vulnerability to cell cycle inhibition in triple-negative breast cancer
Source: Nat Commun. 2019 Jun 28;10:2860. doi: 10.1038/s41467-019-10743-7 (PMC6599020; doi:10.1038/s41467-019-10743-7)
Supplement: Supplementary file 3 — Description of Additional Supplementary Files [file 41467_2019_10743_MOESM3_ESM.pdf]

Description of Additional Supplementary Files

File Name: Supplementary Data 1

Description: Excel file containing top 200 depleted gene list using MAGeCK method (Tab1), the MAGeCK ranking score of the top 200 depleted genes (Tab 2), raw counts (Tab 3), the alteration frequencies of the top 200 depleted genes in cBioportal TNBC clinical samples (Tab 4), and the selection notes for looking for alteration frequencies of top 200 depleted genes in cBioportal TNBC clinical samples (Tab 5).

File Name: Supplementary Data 2

Description: Excel file containing the list of the cytosol DEDD interacted protein IDs in MDA-MB-468 cells through immunoprecipitation assay and the raw spectral counts. The search is against the UniProt human Database. The FDR cutoff is 1%.
