# Supplementary material for: Death effector domain-containing protein induces vulnerability to cell cycle inhibition in triple-negative breast cancer
Source: Nat Commun. 2019 Jun 28;10:2860. doi: 10.1038/s41467-019-10743-7 (PMC6599020; doi:10.1038/s41467-019-10743-7)
Supplement: Supplementary file 6 — Reporting Summary [file 41467_2019_10743_MOESM6_ESM.pdf]

nature research

Corresponding author(s): *Double-blind peer review submissions: write DBPR and your manuscript number here instead of author names.*

Last updated by author(s): *YYYY-MM-DD*

Reporting Summary

Nature Research wishes to improve the reproducibility of the work that we publish. This form provides structure for consistency and transparency in reporting. For further information on Nature Research policies, see Authors & Referees and the Editorial Policy Checklist.

Statistics

For all statistical analyses, confirm that the following items are present in the figure legend, table legend, main text, or Methods section.

n/a	Confirmed	
☐	☒	The exact sample size (*n*) for each experimental group/condition, given as a discrete number and unit of measurement
☐	☒	A statement on whether measurements were taken from distinct samples or whether the same sample was measured repeatedly
☐	☒	The statistical test(s) used AND whether they are one- or two-sided<br>*Only common tests should be described solely by name; describe more complex techniques in the Methods section.*
☐	☒	A description of all covariates tested
☐	☒	A description of any assumptions or corrections, such as tests of normality and adjustment for multiple comparisons
☐	☒	A full description of the statistical parameters including central tendency (e.g. means) or other basic estimates (e.g. regression coefficient) AND variation (e.g. standard deviation) or associated estimates of uncertainty (e.g. confidence intervals)
☒	☐	For null hypothesis testing, the test statistic (e.g. *F*, *t*, *r*) with confidence intervals, effect sizes, degrees of freedom and *P* value noted<br>*Give P values as exact values whenever suitable.*
☒	☐	For Bayesian analysis, information on the choice of priors and Markov chain Monte Carlo settings
☒	☐	For hierarchical and complex designs, identification of the appropriate level for tests and full reporting of outcomes
☒	☐	Estimates of effect sizes (e.g. Cohen's *d*, Pearson's *r*), indicating how they were calculated

Our web collection on statistics for biologists contains articles on many of the points above.

Software and code

Policy information about availability of computer code

Data collection	N/A
Data analysis	CompuSyn Software was used to analyze the drug combination synergism in multiple triple-negative breast cancer cell lines.<br>MAGeCK software was used to analyze the synthetic lethal screen results

For manuscripts utilizing custom algorithms or software that are central to the research but not yet described in published literature, software must be made available to editors/reviewers. We strongly encourage code deposition in a community repository (e.g. GitHub). See the Nature Research guidelines for submitting code & software for further information.

Data

Policy information about availability of data

All manuscripts must include a data availability statement. This statement should provide the following information, where applicable:

- Accession codes, unique identifiers, or web links for publicly available datasets
- A list of figures that have associated raw data
- A description of any restrictions on data availability

All sequencing data that support the findings of this study have been deposited in the National Center for Biotechnology Information Gene Expression Omnibus (GEO) and are accessible through the GEO Series accession number GSE131303 [https://www.ncbi.nlm.nih.gov/geo/query/acc.cgi?acc=GSE131303]. All other relevant data are available from the corresponding author on request. All other data involved in this manuscript (synthetic lethal screen hits, IP-Mass Spec Counts) are provided as supplementary information.

Field-specific reporting

Please select the one below that is the best fit for your research. If you are not sure, read the appropriate sections before making your selection.

☒ Life sciences ☐ Behavioural & social sciences ☐ Ecological, evolutionary & environmental sciences

For a reference copy of the document with all sections, see nature.com/documents/nr-reporting-summary-flat.pdf

Life sciences study design

All studies must disclose on these points even when the disclosure is negative.

Sample size	For in vivo experiment, sample size was selected based power analysis
Data exclusions	N/A
Replication	Minimum three biological repeat of the same experiment were performed in this manuscript.
Randomization	For in vivo experiments, mice were randomized before being assigned to the treatment groups.
Blinding	When conducting pathological analysis of tissue sample, the researchers were blinded to treatment groups.

Reporting for specific materials, systems and methods

We require information from authors about some types of materials, experimental systems and methods used in many studies. Here, indicate whether each material, system or method listed is relevant to your study. If you are not sure if a list item applies to your research, read the appropriate section before selecting a response.

Materials & experimental systems

n/a	Involved in the study	
☐	☒	Antibodies
☐	☒	Eukaryotic cell lines
☒	☐	Palaeontology
☐	☒	Animals and other organisms
☒	☐	Human research participants
☒	☐	Clinical data

Methods

n/a	Involved in the study	
☒	☐	ChIP-seq
☐	☒	Flow cytometry
☒	☐	MRI-based neuroimaging

Antibodies

Antibodies used	DEDD (Sc-271192) and HSC-70 (sc-7298) antibodies were purchased from Santa Cruz Biotechnology. β-actin (ab8226) was purchased from Abcam. Flag M2 (F1804) was purchased from Sigma-Aldrich. Cyclin B1 (#4138), Cyclin D1 (#2978), Cyclin A2 (#4656), Cyclin E1 (#4129), p-Erk (#4370), Erk (#4695), p-EGFR (#3777), EGFR (#4267), p-Akt (#4060), Akt (#4691), Rb (#9309), p-Rb (#9308), RBL1 (#89798), Ubi (#3933), p-FoxM1 (#14655) and FoxM1 (#5436) All other antibodies were purchased from Cell Signaling Technology.
Validation	All antibodies were used in the study are purchased and validated from commercial vendors.

Eukaryotic cell lines

Policy information about cell lines

Cell line source(s)	293FT and triple-negative breast cancer cell lines were purchased from ATCC, Normal human mammary epithelial lines from Komen Tissue Bank (KTB) were obtained from our collaborator Dr. Nakshatri.
Authentication	DNA analysis by Genetica Cell Line Testing.
Mycoplasma contamination	None
Commonly misidentified lines (See ICLAC register)	None

Animals and other organisms

Policy information about studies involving animals; ARRIVE guidelines recommended for reporting animal research

Laboratory animals	Rag1 knockout (Rag1-/-) and NOD SCID mice were obtained from Taconic Farms (Hudson, New York)
Wild animals	N/A
Field-collected samples	N/A
Ethics oversight	The studies was monitored by the Notre Dame Freimann Life Sciences Center under IACUC approved protocol that complys with the regulations.

Note that full information on the approval of the study protocol must also be provided in the manuscript.

Flow Cytometry

Plots

Confirm that:

☒ The axis labels state the marker and fluorochrome used (e.g. CD4-FITC).

☒ The axis scales are clearly visible. Include numbers along axes only for bottom left plot of group (a 'group' is an analysis of identical markers).

☒ All plots are contour plots with outliers or pseudocolor plots.

☒ A numerical value for number of cells or percentage (with statistics) is provided.

Methodology

Sample preparation	4% PFA or 75% ethanol fixed cells
Instrument	FC500
Software	FlowJo
Cell population abundance	minimum 10,000 events
Gating strategy	Normalized to the control groups, or compensate to negative or single color samples.

☒ Tick this box to confirm that a figure exemplifying the gating strategy is provided in the Supplementary Information.
