# Supplementary material for: Death effector domain-containing protein induces vulnerability to cell cycle inhibition in triple-negative breast cancer
Source: Nat Commun. 2019 Jun 28;10:2860. doi: 10.1038/s41467-019-10743-7 (PMC6599020; doi:10.1038/s41467-019-10743-7)
Supplement: Supplementary file 7 — Source Data [file 41467_2019_10743_MOESM7_ESM.pptx]

## Slide 1
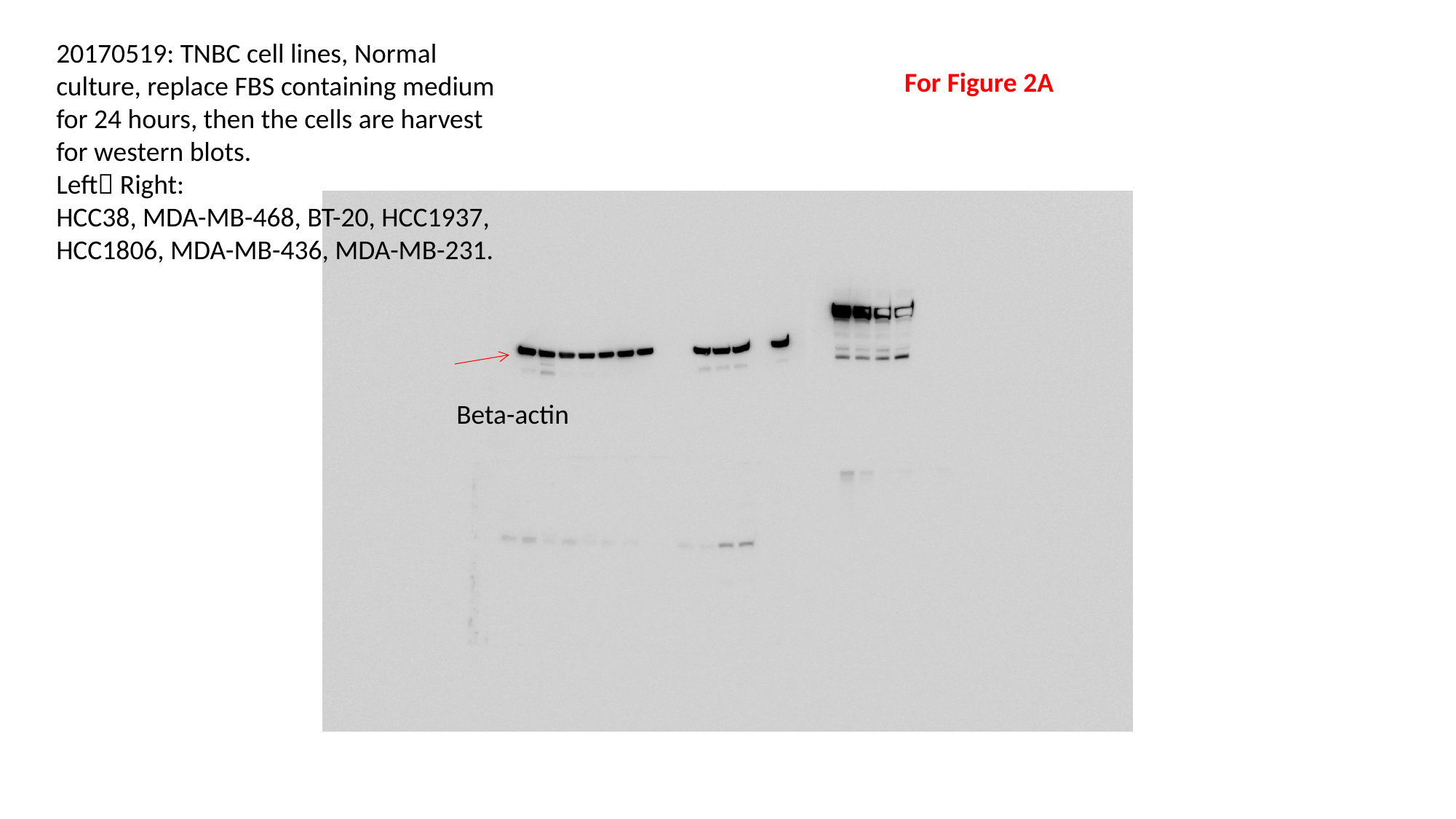

20170519: TNBC cell lines, Normal culture, replace FBS containing medium for 24 hours, then the cells are harvest for western blots.
Left Right:
HCC38, MDA-MB-468, BT-20, HCC1937, HCC1806, MDA-MB-436, MDA-MB-231.
For Figure 2A
Beta-actin

## Slide 2
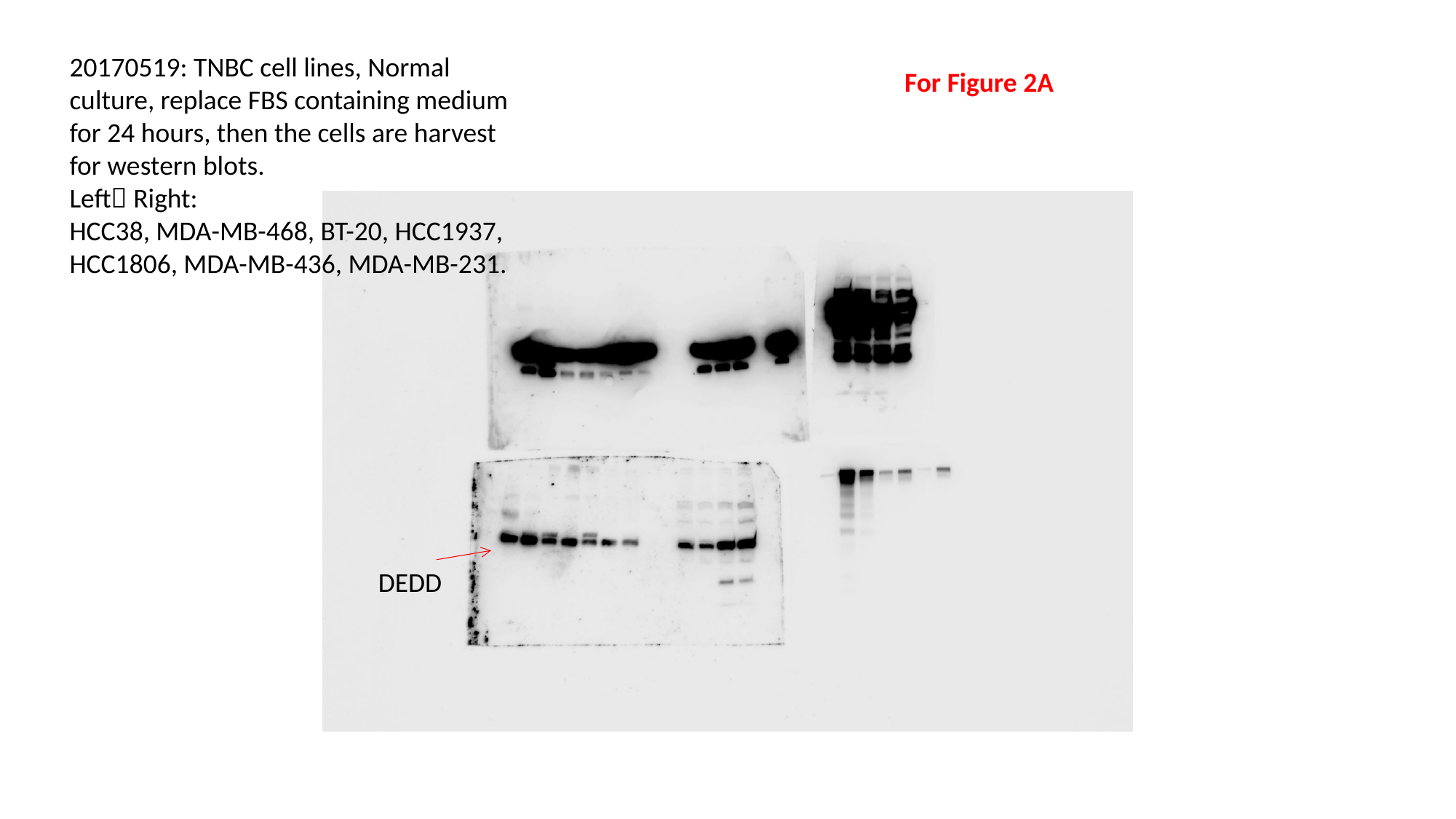

20170519: TNBC cell lines, Normal culture, replace FBS containing medium for 24 hours, then the cells are harvest for western blots.
Left Right:
HCC38, MDA-MB-468, BT-20, HCC1937, HCC1806, MDA-MB-436, MDA-MB-231.
For Figure 2A
DEDD

## Slide 3
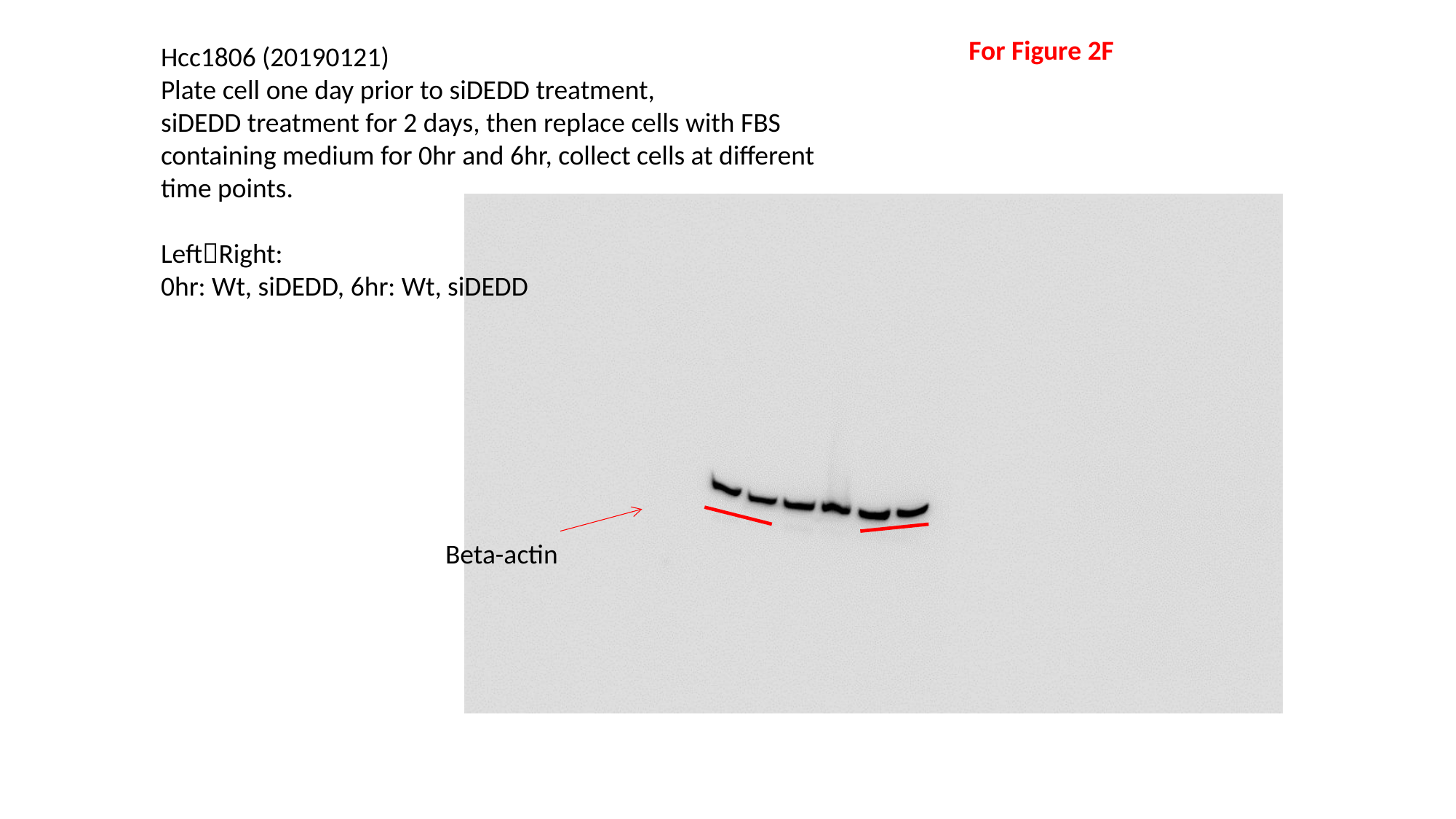

For Figure 2F
Hcc1806 (20190121)
Plate cell one day prior to siDEDD treatment,
siDEDD treatment for 2 days, then replace cells with FBS containing medium for 0hr and 6hr, collect cells at different time points.
LeftRight:
0hr: Wt, siDEDD, 6hr: Wt, siDEDD
Beta-actin

## Slide 4
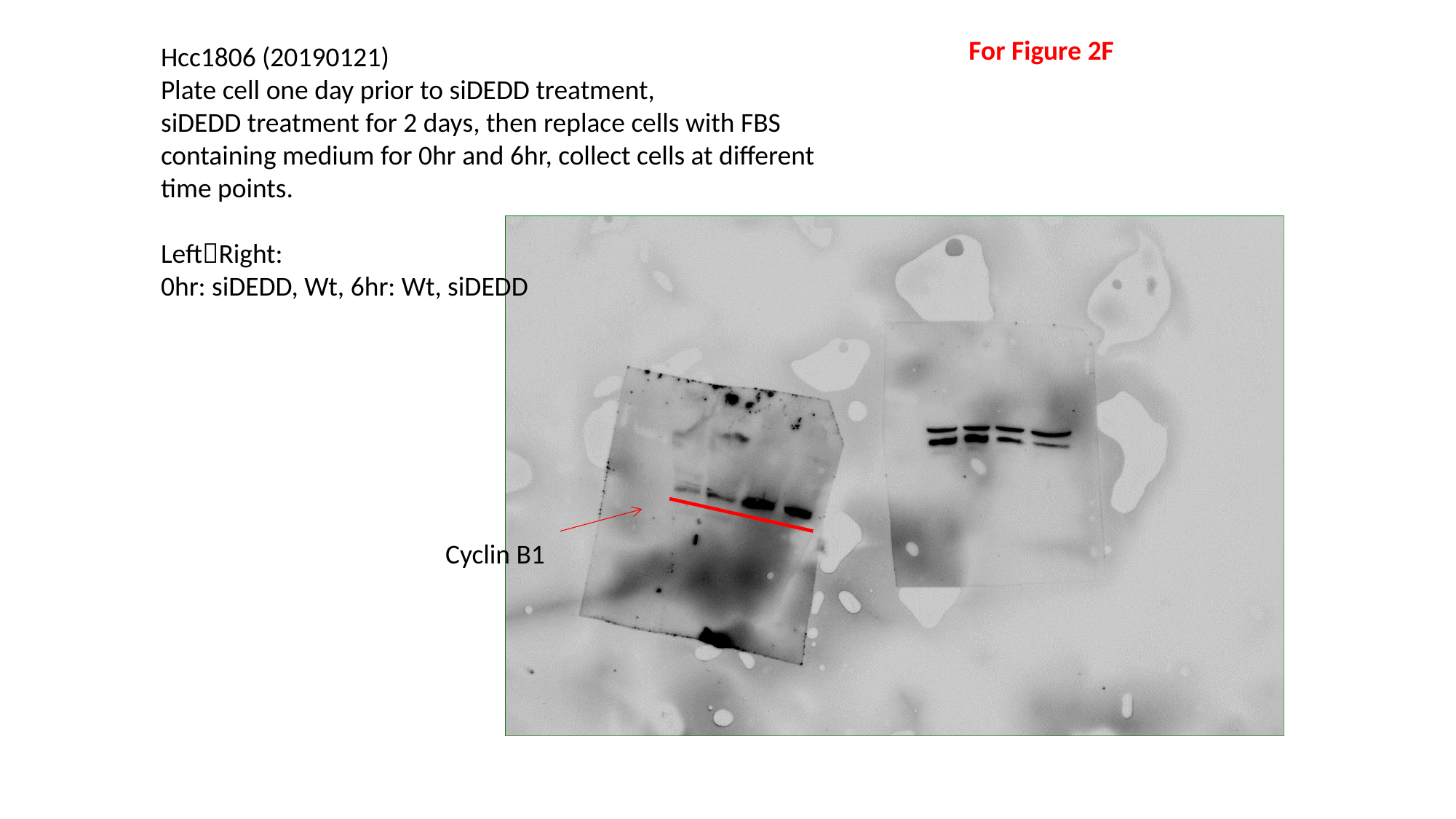

For Figure 2F
Hcc1806 (20190121)
Plate cell one day prior to siDEDD treatment,
siDEDD treatment for 2 days, then replace cells with FBS containing medium for 0hr and 6hr, collect cells at different time points.
LeftRight:
0hr: siDEDD, Wt, 6hr: Wt, siDEDD
Cyclin B1

## Slide 5
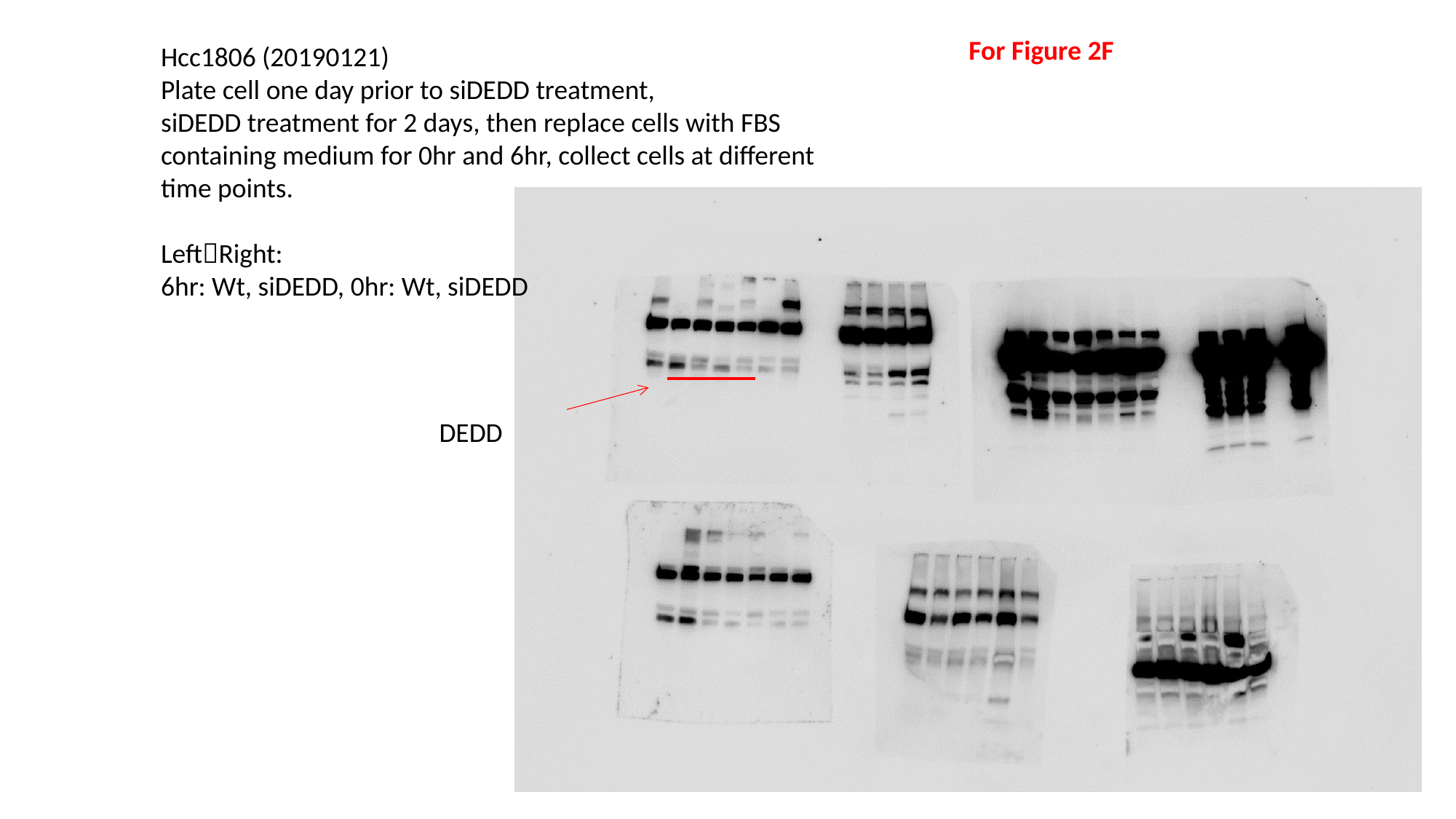

For Figure 2F
Hcc1806 (20190121)
Plate cell one day prior to siDEDD treatment,
siDEDD treatment for 2 days, then replace cells with FBS containing medium for 0hr and 6hr, collect cells at different time points.
LeftRight:
6hr: Wt, siDEDD, 0hr: Wt, siDEDD
DEDD

## Slide 6
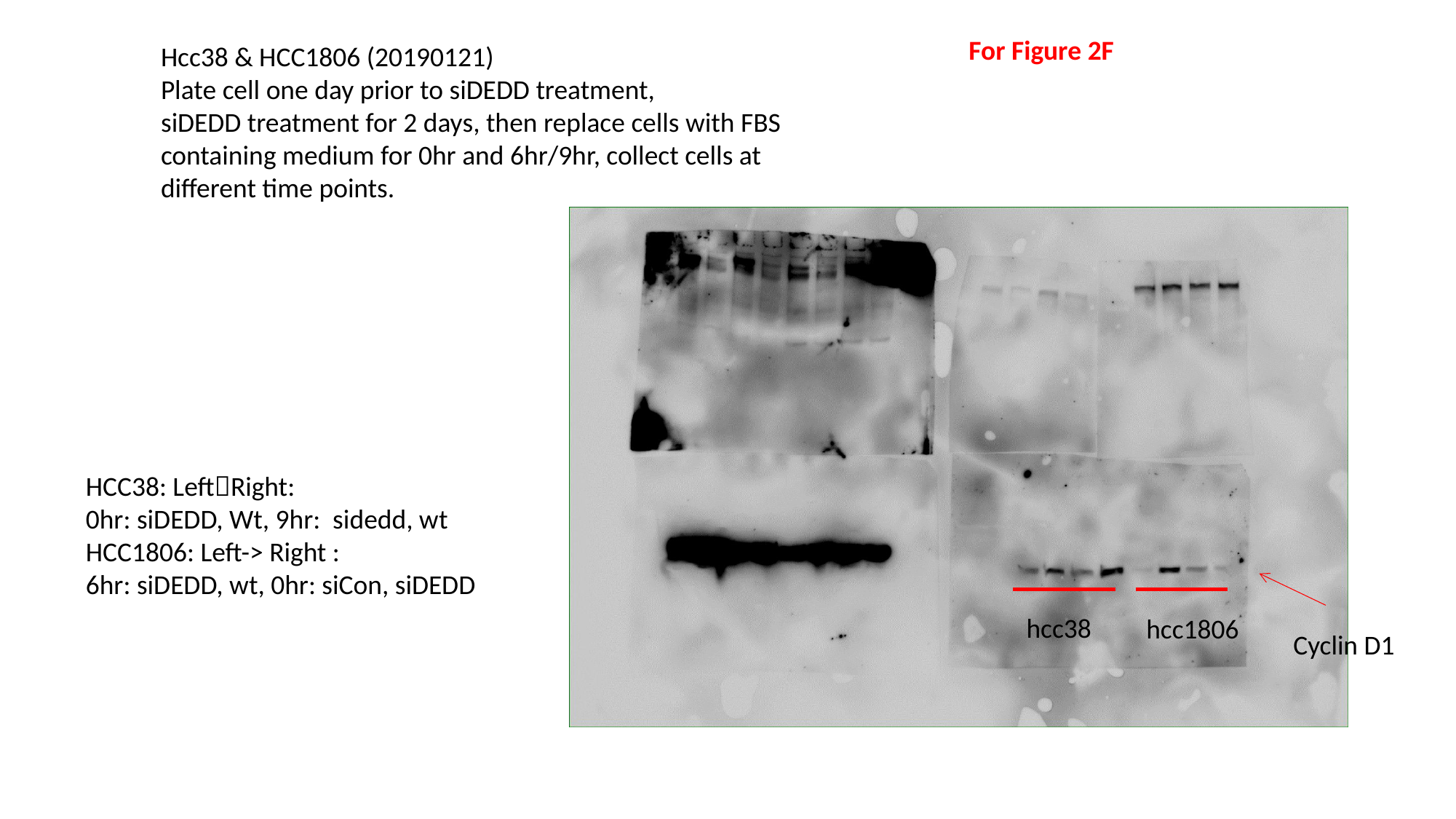

For Figure 2F
Hcc38 & HCC1806 (20190121)
Plate cell one day prior to siDEDD treatment,
siDEDD treatment for 2 days, then replace cells with FBS containing medium for 0hr and 6hr/9hr, collect cells at different time points.
HCC38: LeftRight:
0hr: siDEDD, Wt, 9hr: sidedd, wt
HCC1806: Left-> Right :
6hr: siDEDD, wt, 0hr: siCon, siDEDD
hcc38
hcc1806
Cyclin D1

## Slide 7
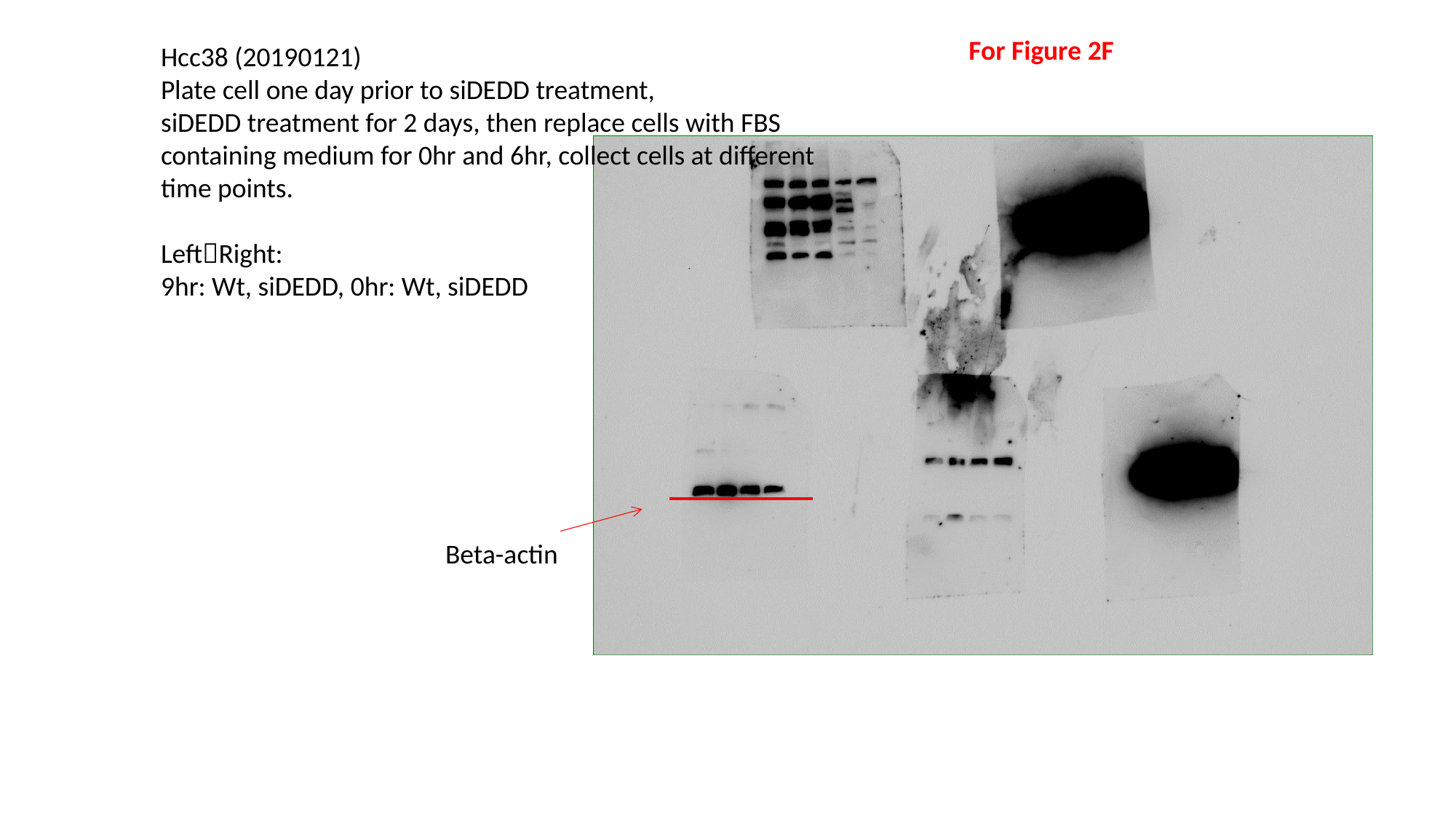

For Figure 2F
Hcc38 (20190121)
Plate cell one day prior to siDEDD treatment,
siDEDD treatment for 2 days, then replace cells with FBS containing medium for 0hr and 6hr, collect cells at different time points.
LeftRight:
9hr: Wt, siDEDD, 0hr: Wt, siDEDD
Beta-actin

## Slide 8
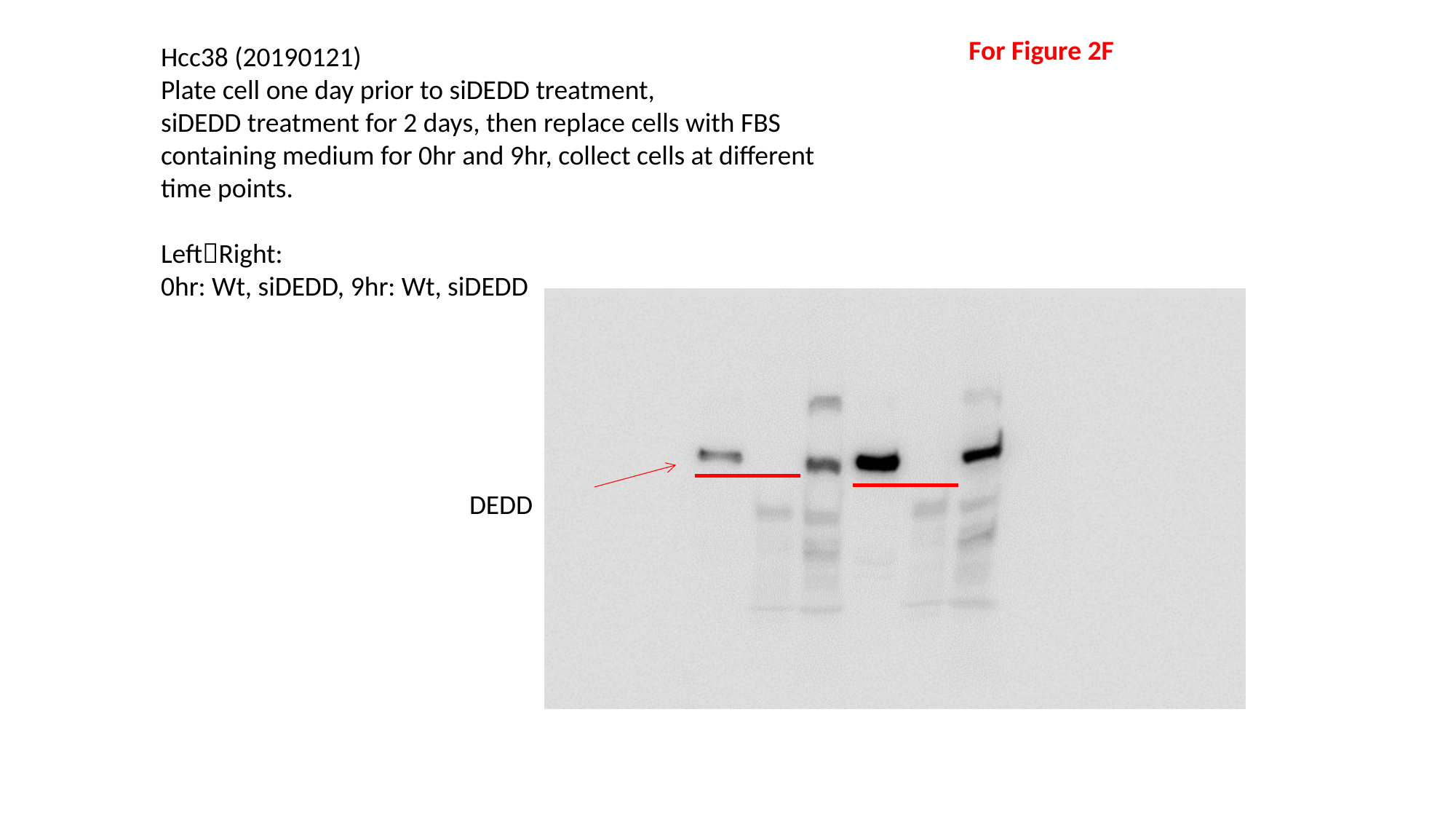

For Figure 2F
Hcc38 (20190121)
Plate cell one day prior to siDEDD treatment,
siDEDD treatment for 2 days, then replace cells with FBS containing medium for 0hr and 9hr, collect cells at different time points.
LeftRight:
0hr: Wt, siDEDD, 9hr: Wt, siDEDD
DEDD

## Slide 9
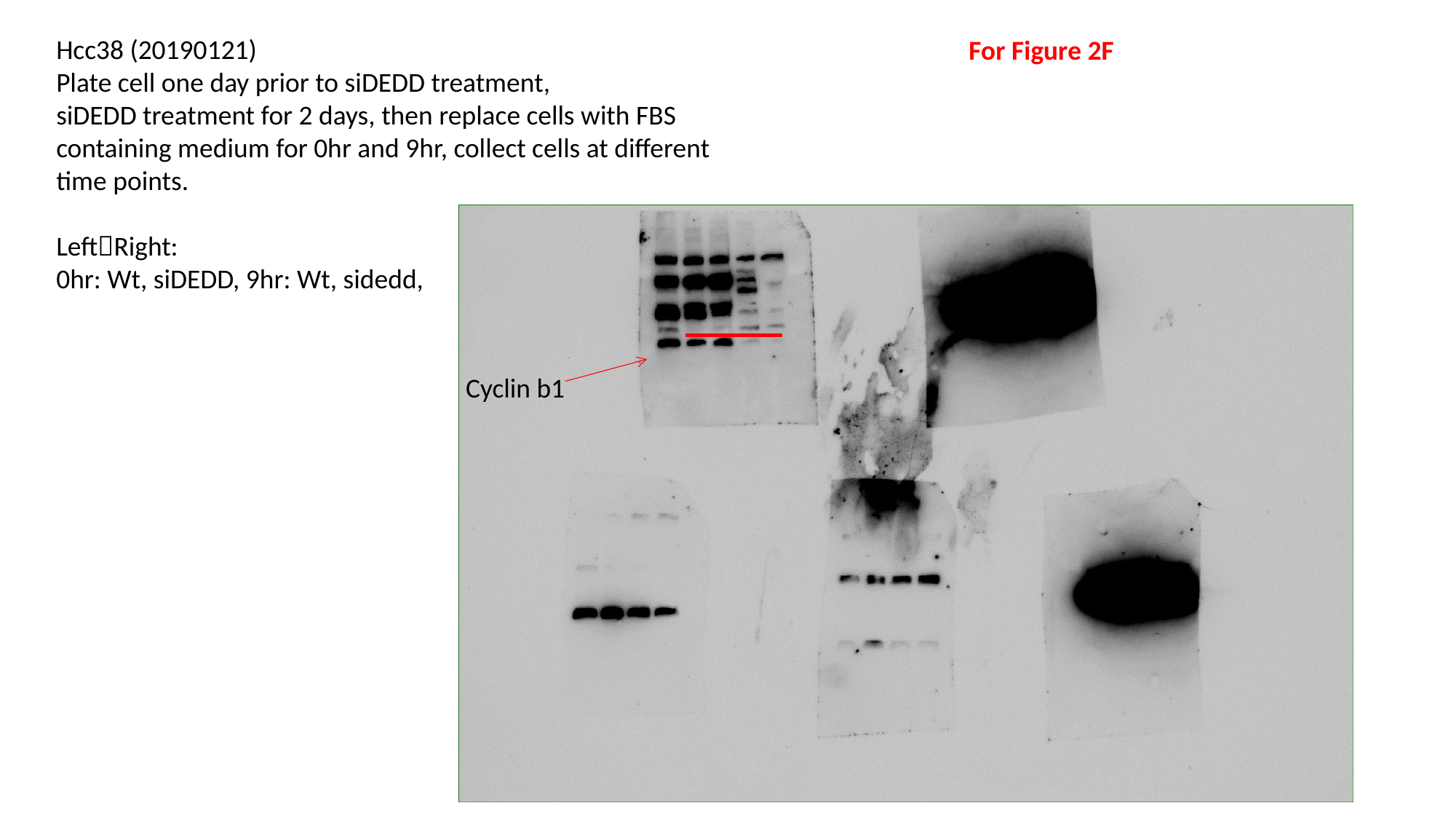

Hcc38 (20190121)
Plate cell one day prior to siDEDD treatment,
siDEDD treatment for 2 days, then replace cells with FBS containing medium for 0hr and 9hr, collect cells at different time points.
LeftRight:
0hr: Wt, siDEDD, 9hr: Wt, sidedd,
For Figure 2F
Cyclin b1

## Slide 10
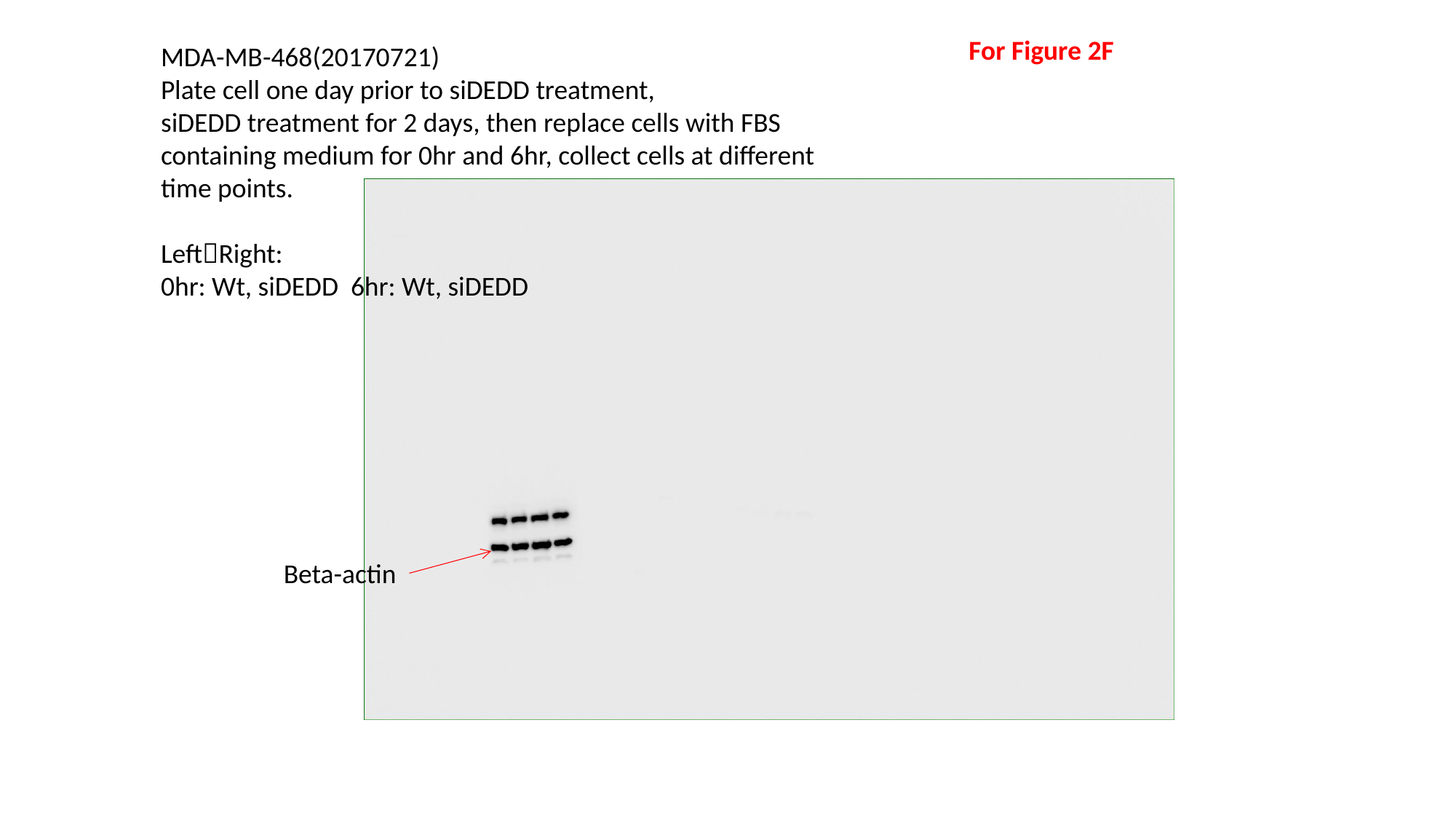

For Figure 2F
MDA-MB-468(20170721)
Plate cell one day prior to siDEDD treatment,
siDEDD treatment for 2 days, then replace cells with FBS containing medium for 0hr and 6hr, collect cells at different time points.
LeftRight:
0hr: Wt, siDEDD 6hr: Wt, siDEDD
Beta-actin

## Slide 11
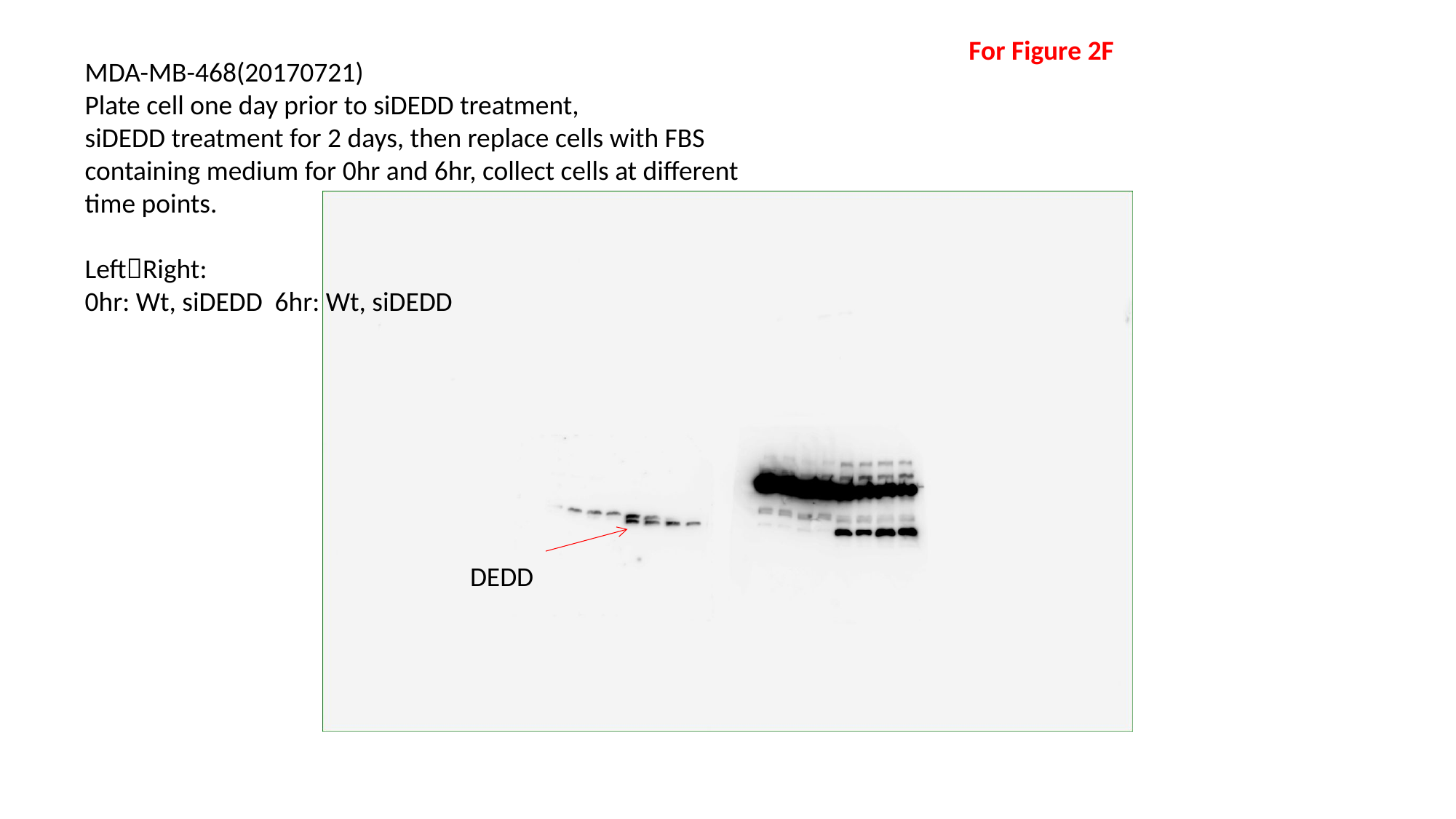

For Figure 2F
MDA-MB-468(20170721)
Plate cell one day prior to siDEDD treatment,
siDEDD treatment for 2 days, then replace cells with FBS containing medium for 0hr and 6hr, collect cells at different time points.
LeftRight:
0hr: Wt, siDEDD 6hr: Wt, siDEDD
DEDD

## Slide 12
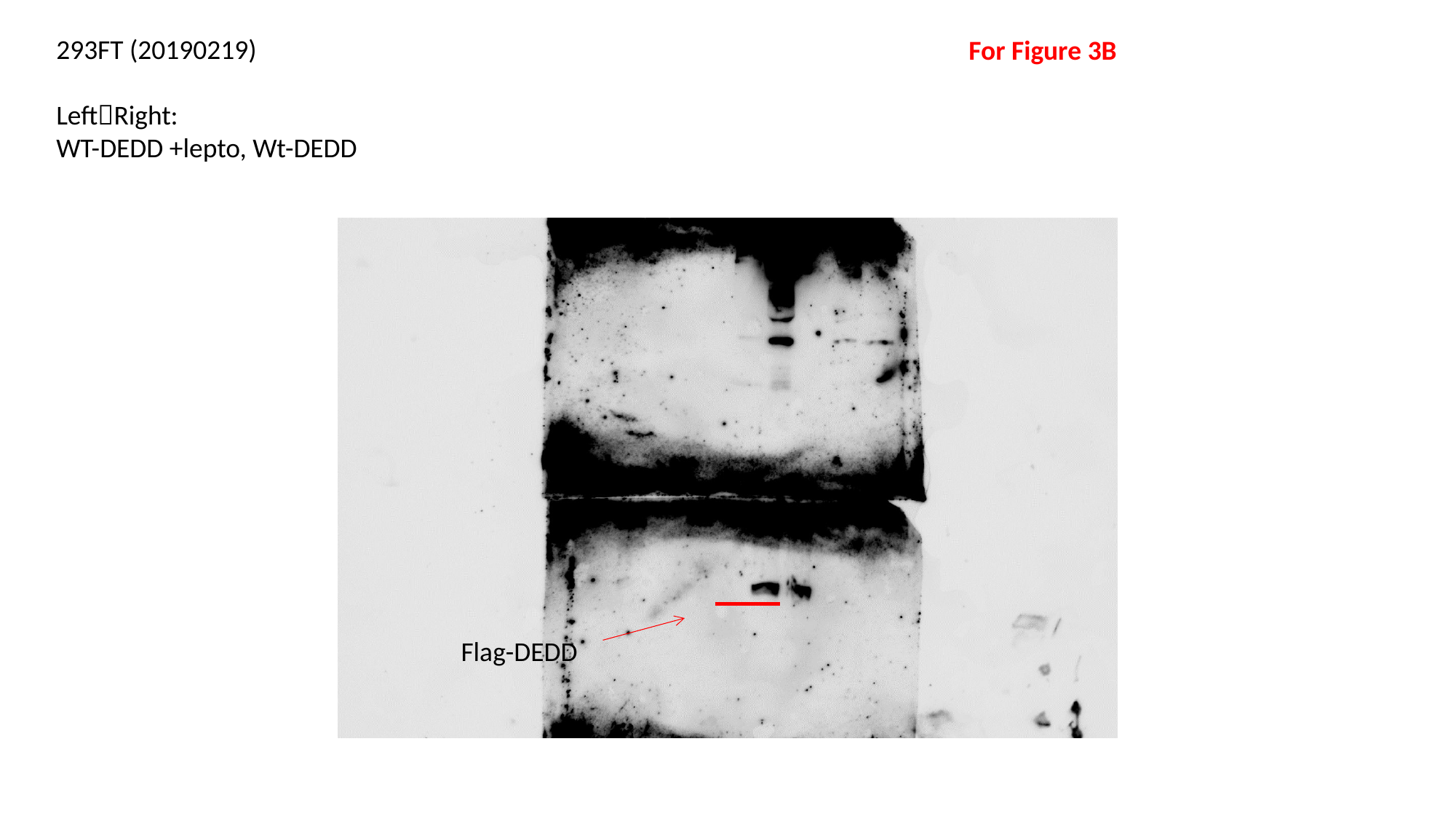

293FT (20190219)
LeftRight:
WT-DEDD +lepto, Wt-DEDD
For Figure 3B
Flag-DEDD

## Slide 13
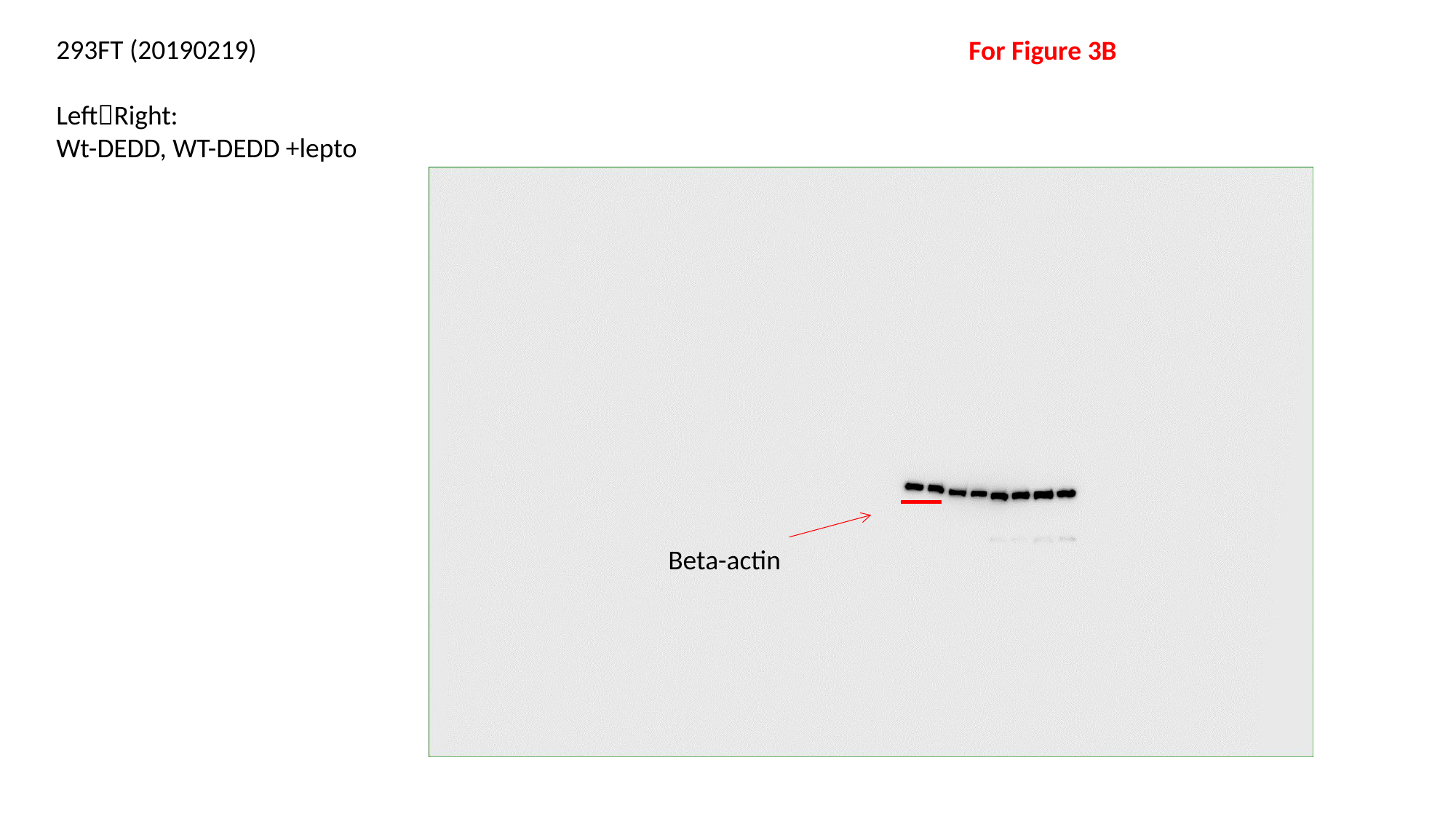

293FT (20190219)
LeftRight:
Wt-DEDD, WT-DEDD +lepto
For Figure 3B
Beta-actin

## Slide 14
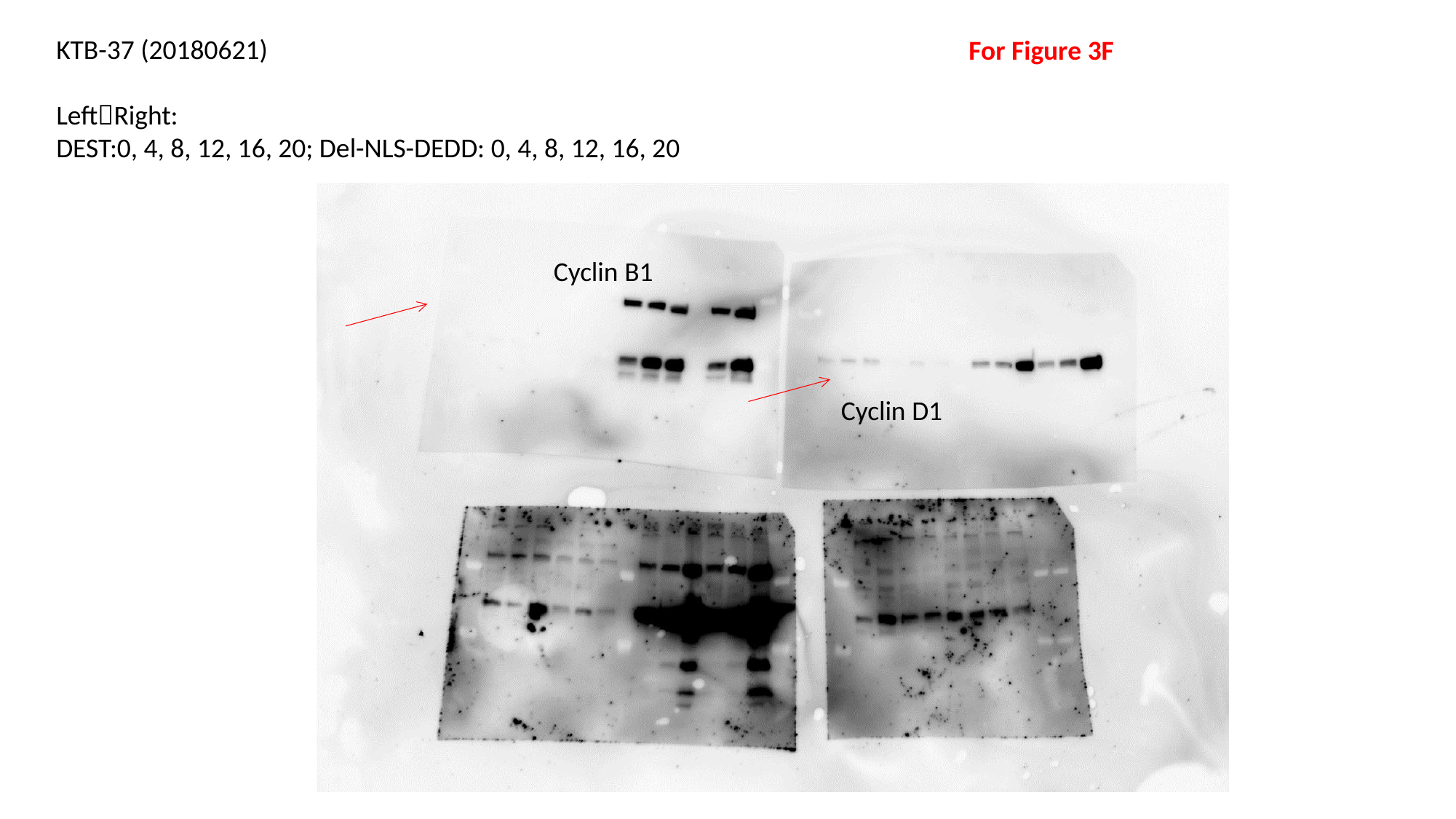

KTB-37 (20180621)
LeftRight:
DEST:0, 4, 8, 12, 16, 20; Del-NLS-DEDD: 0, 4, 8, 12, 16, 20
For Figure 3F
Cyclin B1
Cyclin D1

## Slide 15
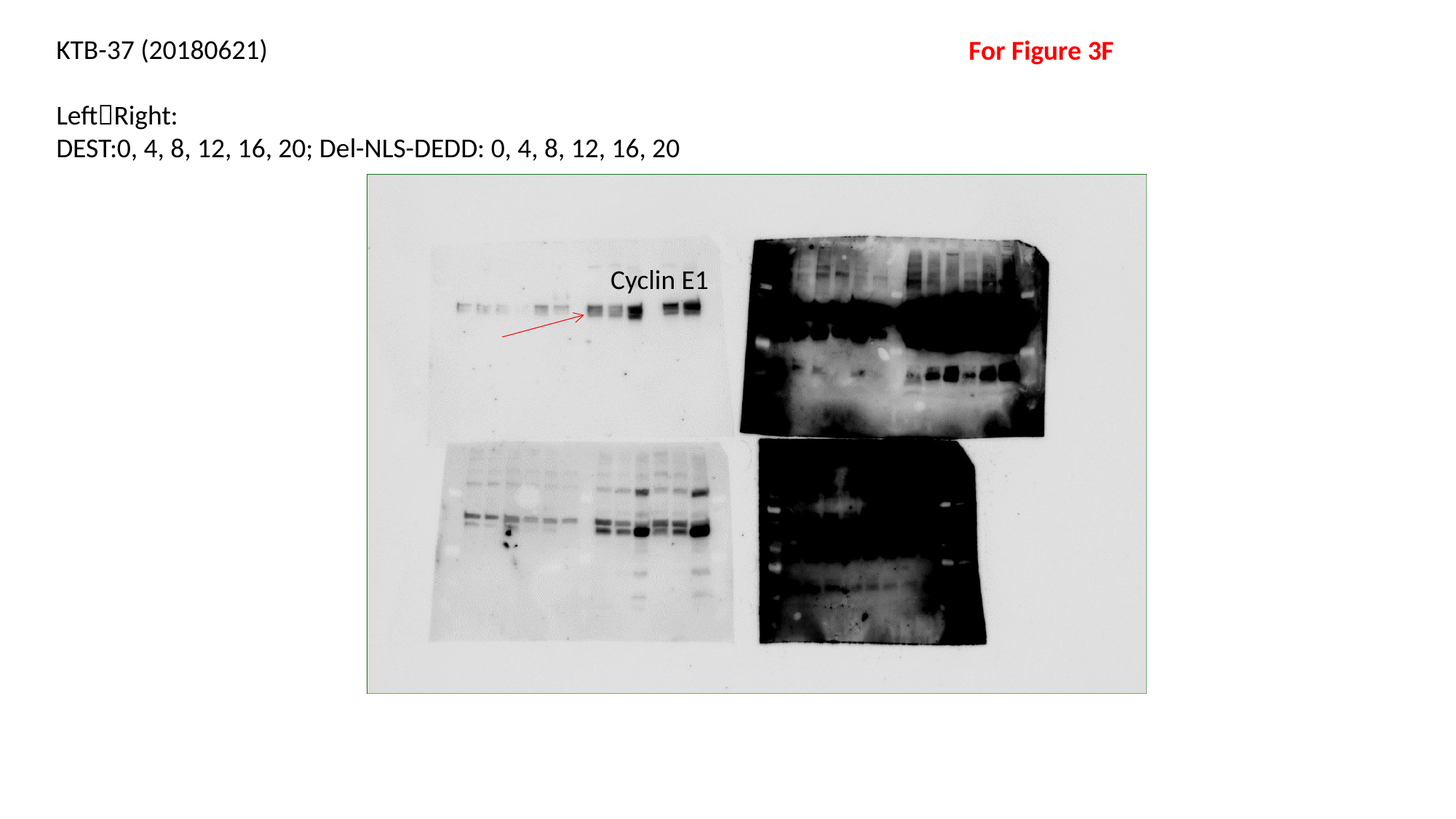

KTB-37 (20180621)
LeftRight:
DEST:0, 4, 8, 12, 16, 20; Del-NLS-DEDD: 0, 4, 8, 12, 16, 20
For Figure 3F
Cyclin E1

## Slide 16
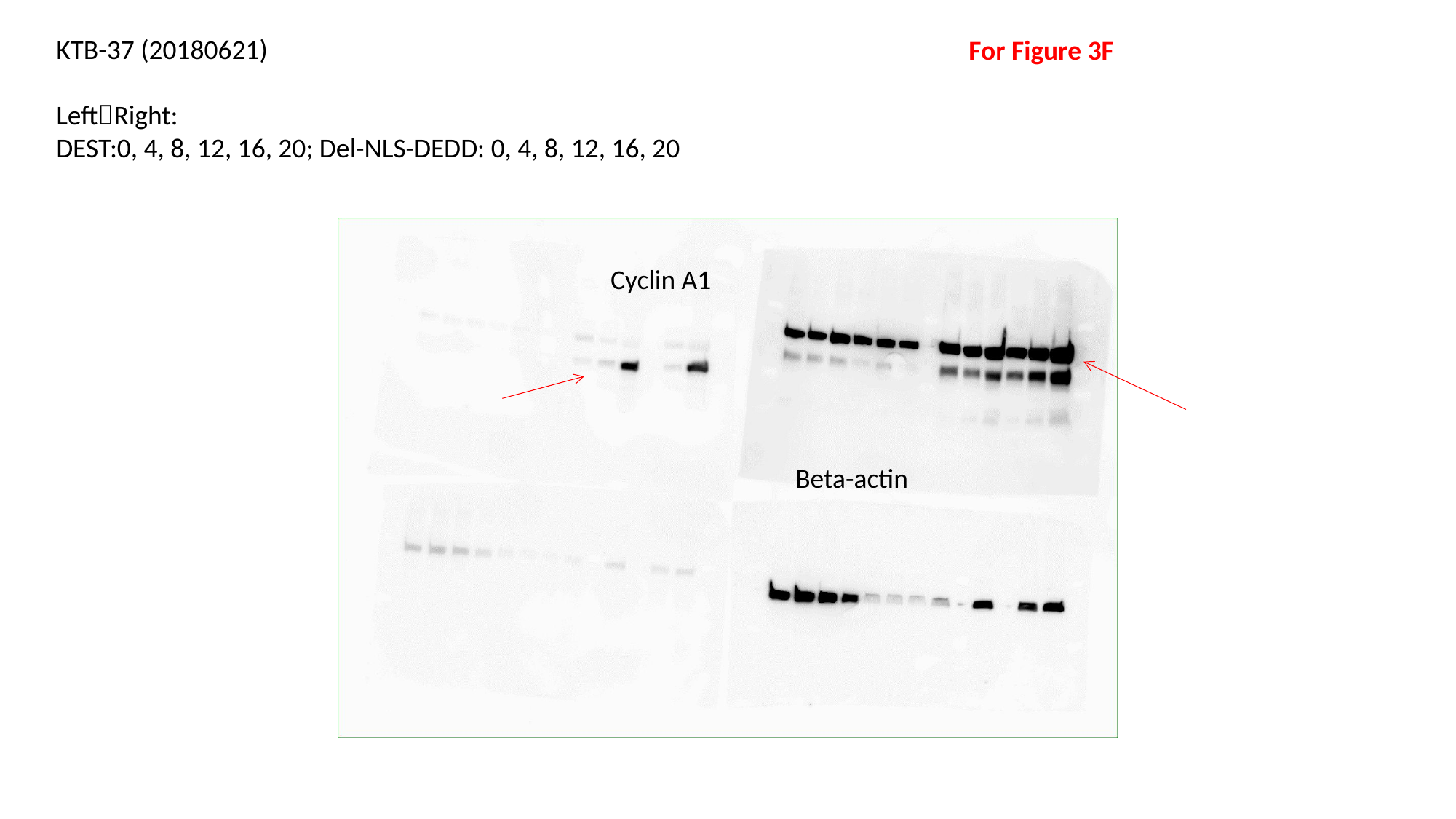

KTB-37 (20180621)
LeftRight:
DEST:0, 4, 8, 12, 16, 20; Del-NLS-DEDD: 0, 4, 8, 12, 16, 20
For Figure 3F
Cyclin A1
Beta-actin

## Slide 17
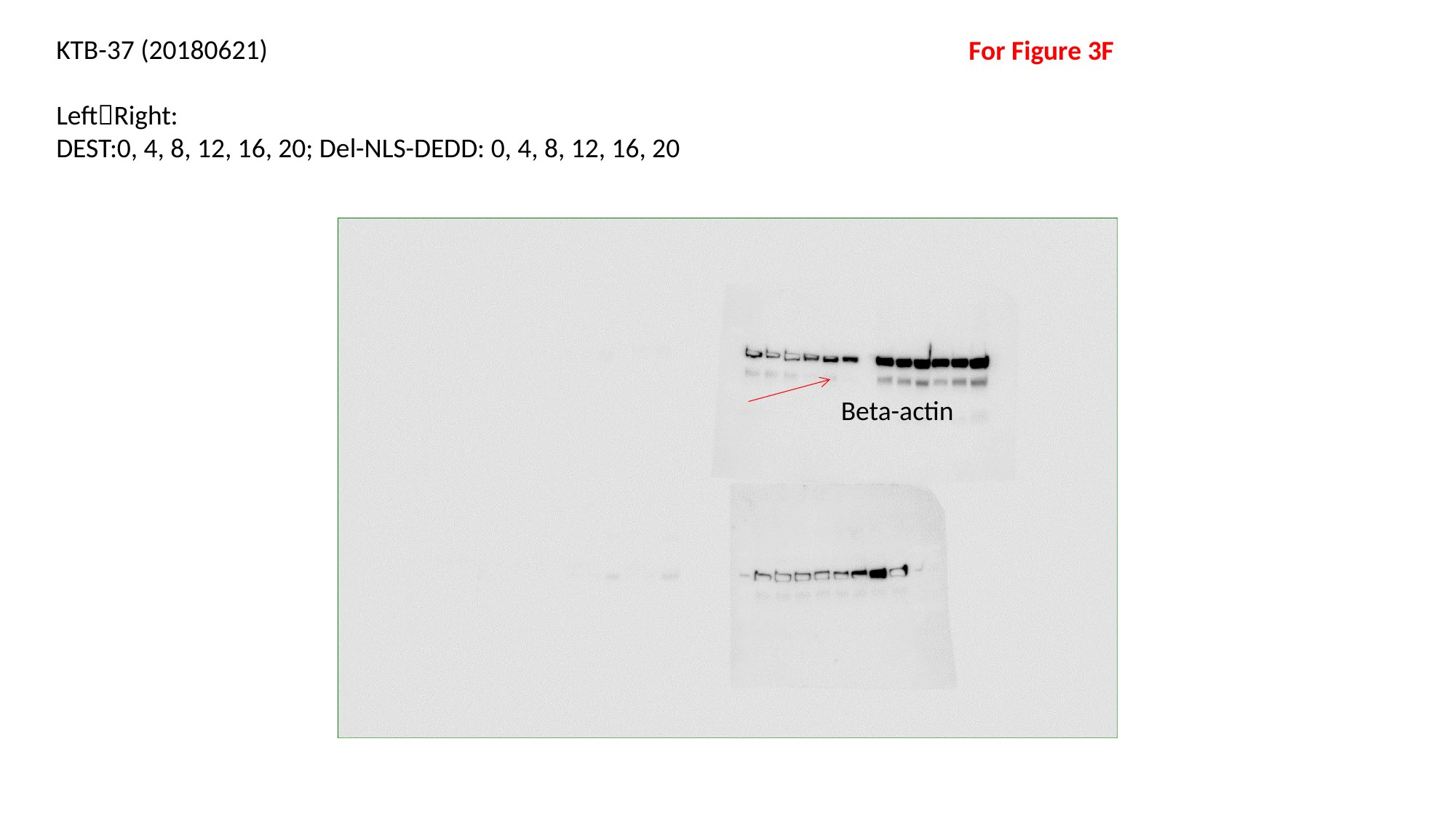

KTB-37 (20180621)
LeftRight:
DEST:0, 4, 8, 12, 16, 20; Del-NLS-DEDD: 0, 4, 8, 12, 16, 20
For Figure 3F
Beta-actin

## Slide 18
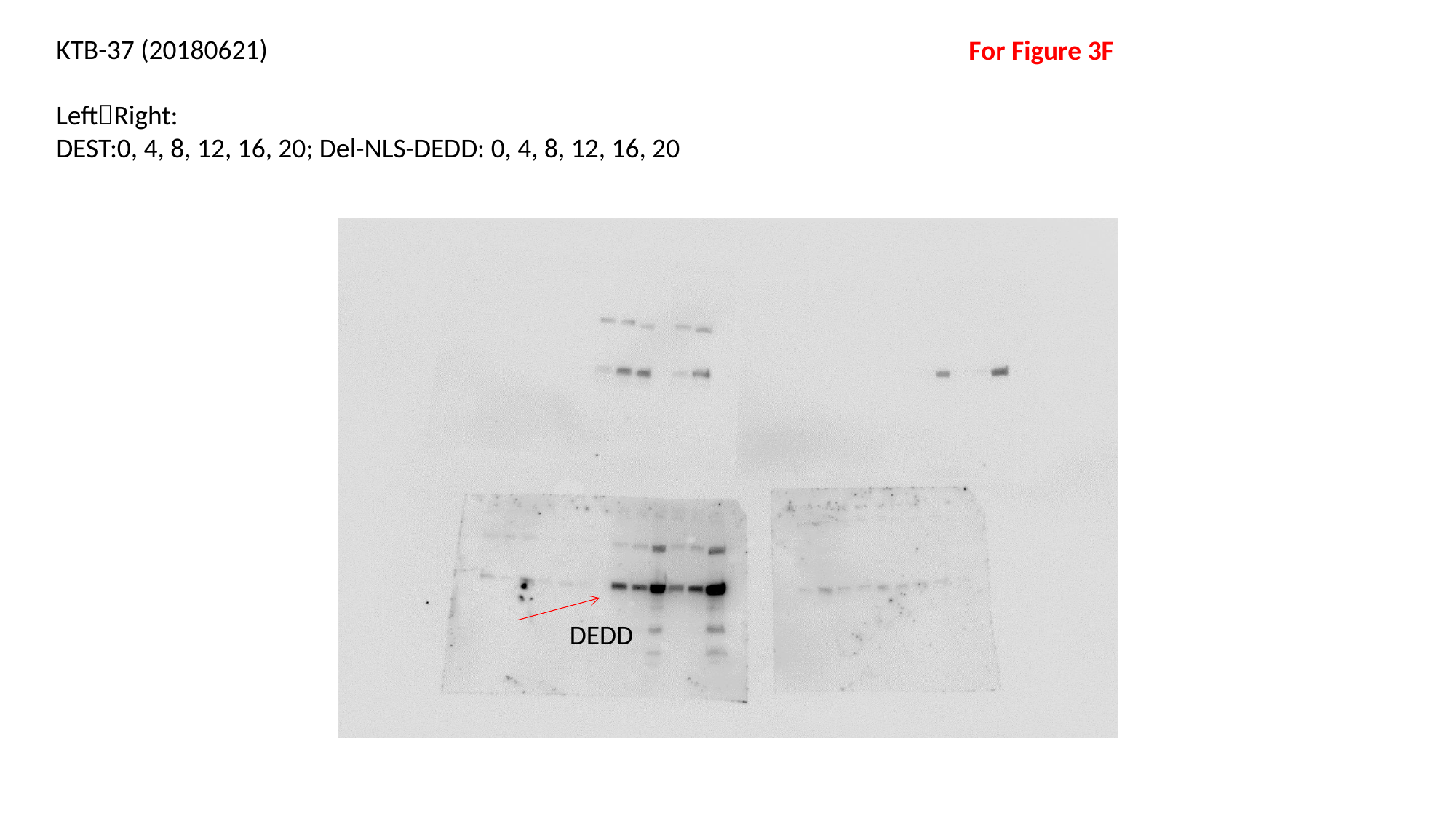

KTB-37 (20180621)
LeftRight:
DEST:0, 4, 8, 12, 16, 20; Del-NLS-DEDD: 0, 4, 8, 12, 16, 20
For Figure 3F
DEDD

## Slide 19
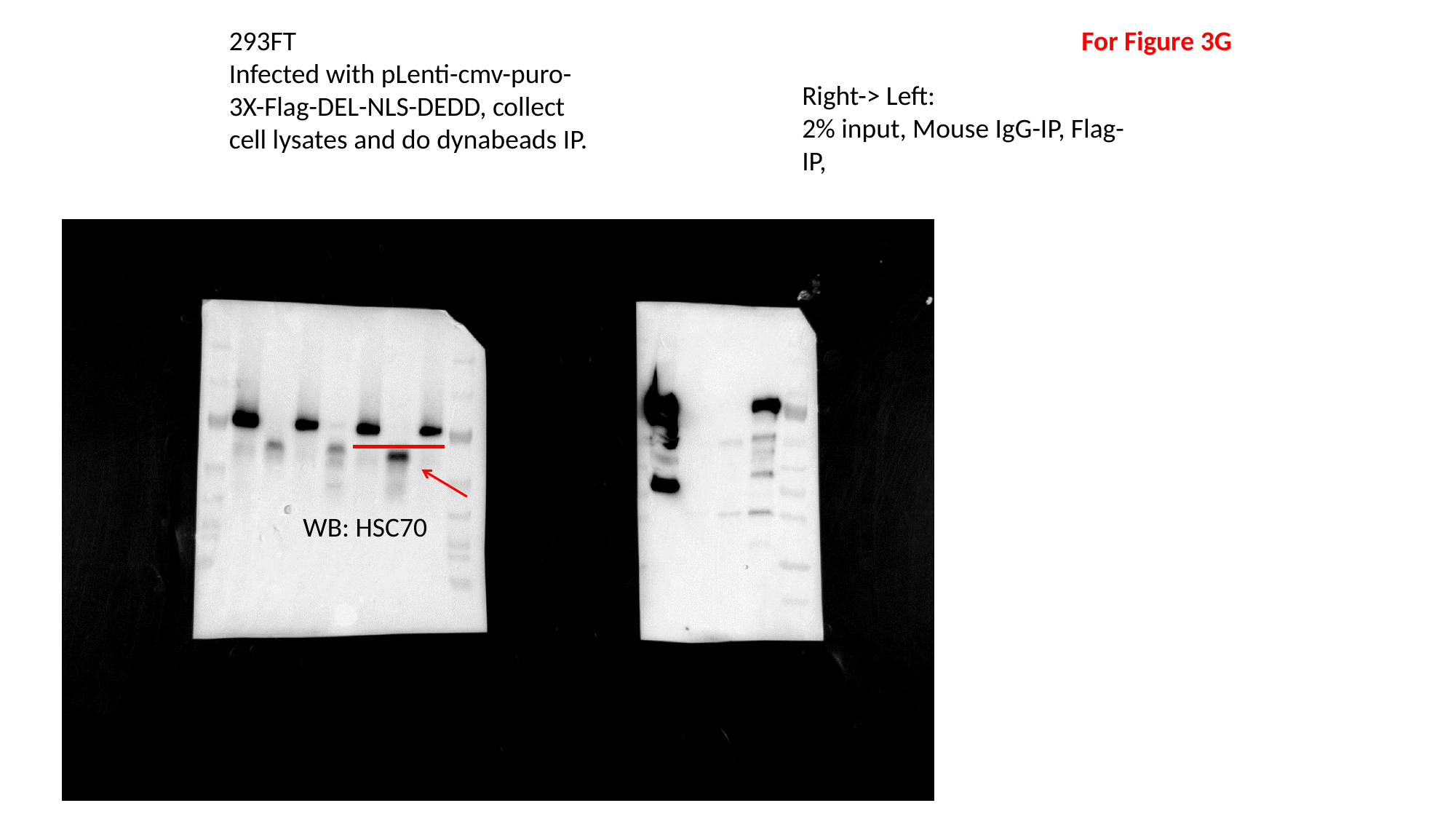

293FT
Infected with pLenti-cmv-puro-3X-Flag-DEL-NLS-DEDD, collect cell lysates and do dynabeads IP.
For Figure 3G
Right-> Left:
2% input, Mouse IgG-IP, Flag-IP,
WB: HSC70

## Slide 20
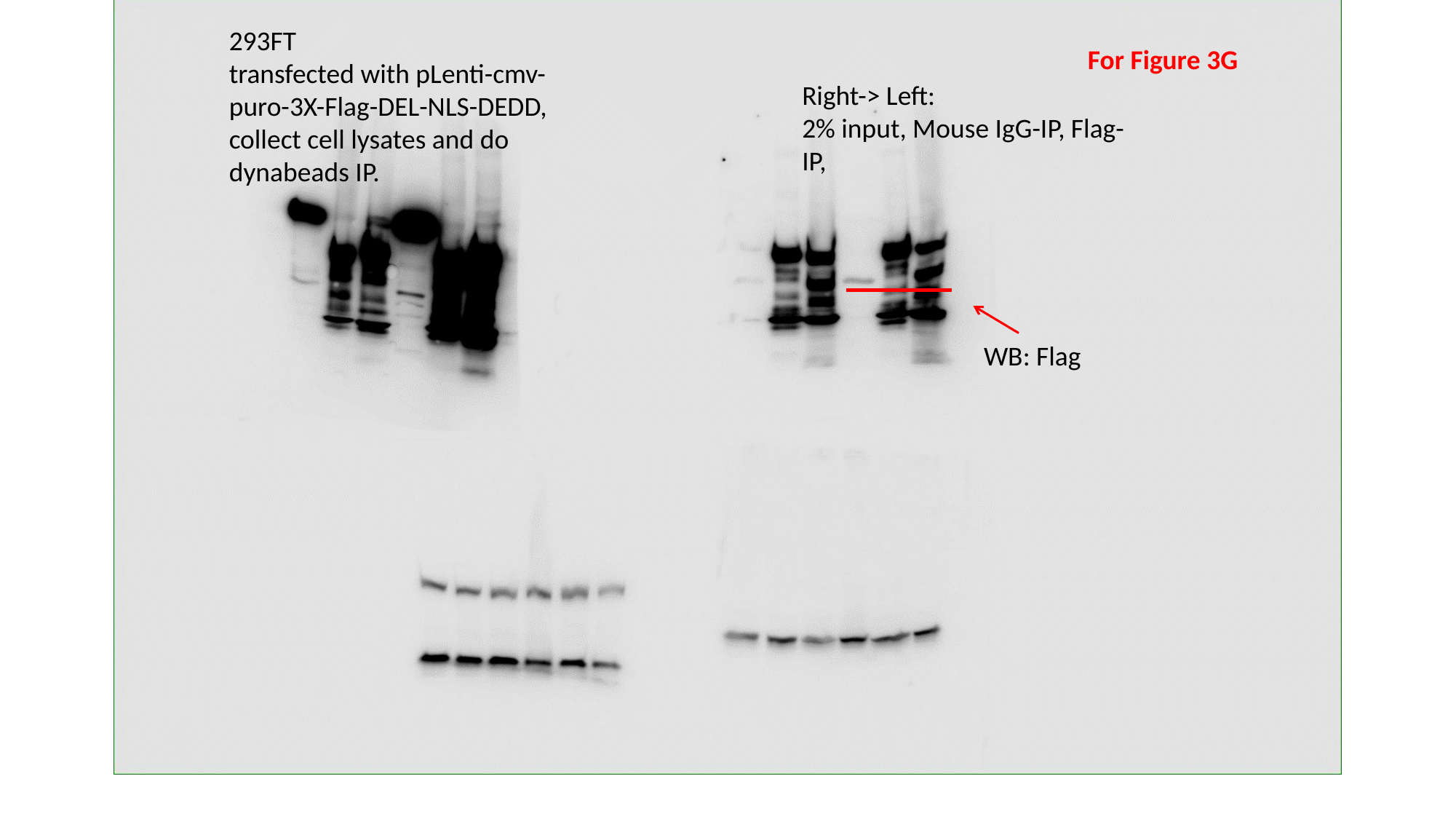

293FT
transfected with pLenti-cmv-puro-3X-Flag-DEL-NLS-DEDD, collect cell lysates and do dynabeads IP.
For Figure 3G
Right-> Left:
2% input, Mouse IgG-IP, Flag-IP,
WB: Flag

## Slide 21
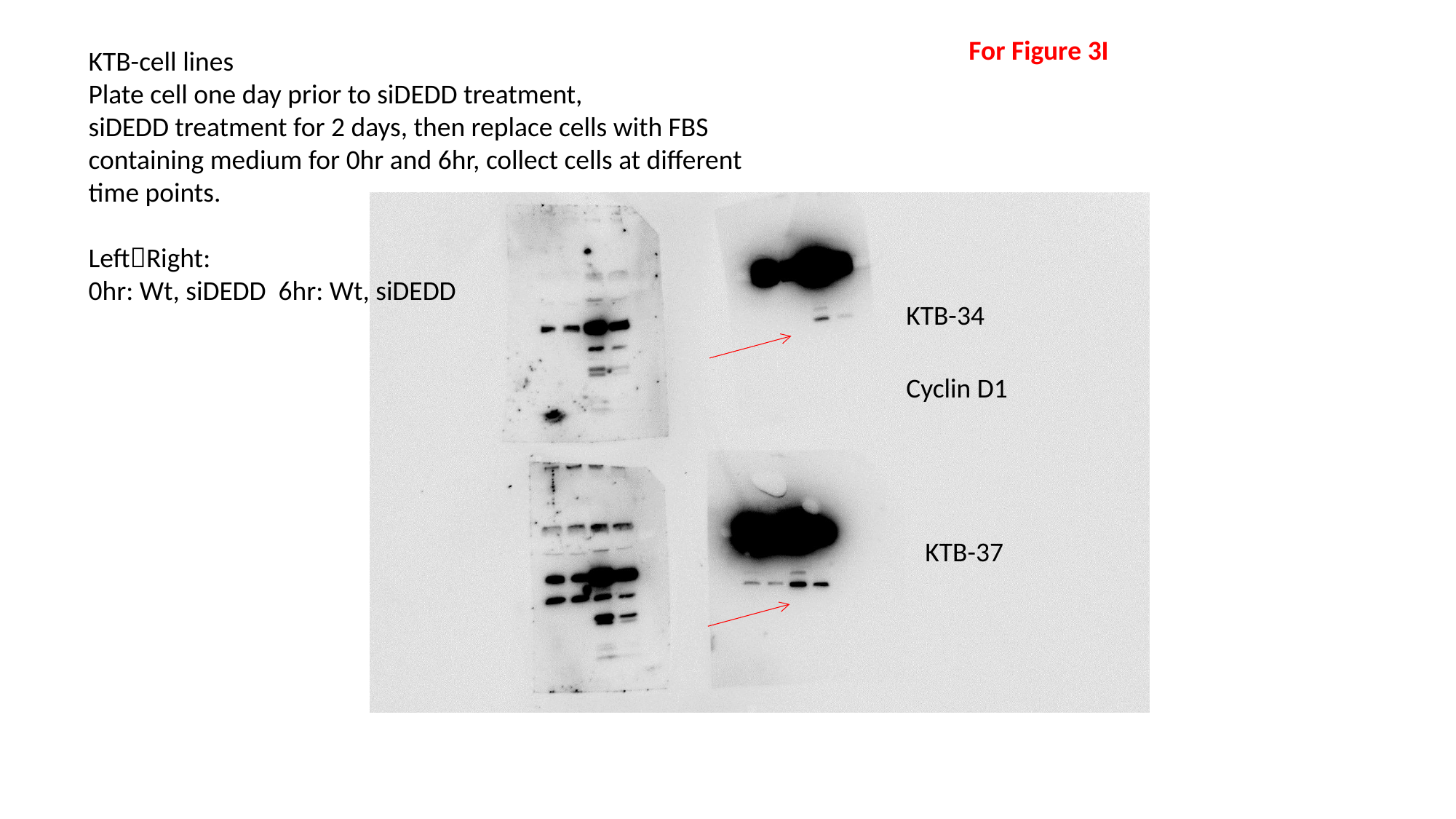

For Figure 3I
KTB-cell lines
Plate cell one day prior to siDEDD treatment,
siDEDD treatment for 2 days, then replace cells with FBS containing medium for 0hr and 6hr, collect cells at different time points.
LeftRight:
0hr: Wt, siDEDD 6hr: Wt, siDEDD
KTB-34
Cyclin D1
KTB-37

## Slide 22
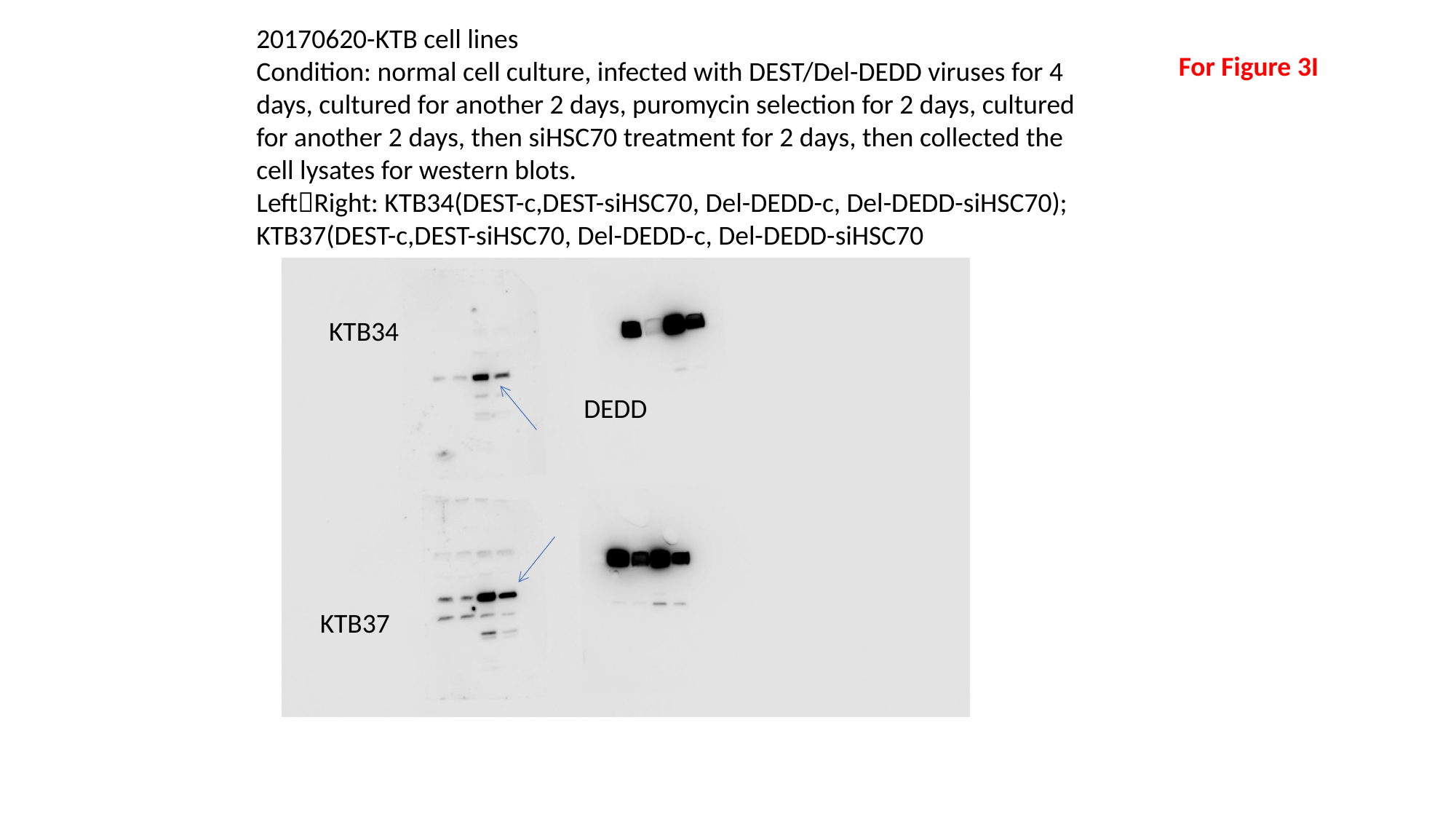

20170620-KTB cell lines
Condition: normal cell culture, infected with DEST/Del-DEDD viruses for 4 days, cultured for another 2 days, puromycin selection for 2 days, cultured for another 2 days, then siHSC70 treatment for 2 days, then collected the cell lysates for western blots.
LeftRight: KTB34(DEST-c,DEST-siHSC70, Del-DEDD-c, Del-DEDD-siHSC70); KTB37(DEST-c,DEST-siHSC70, Del-DEDD-c, Del-DEDD-siHSC70
For Figure 3I
KTB34
DEDD
KTB37

## Slide 23
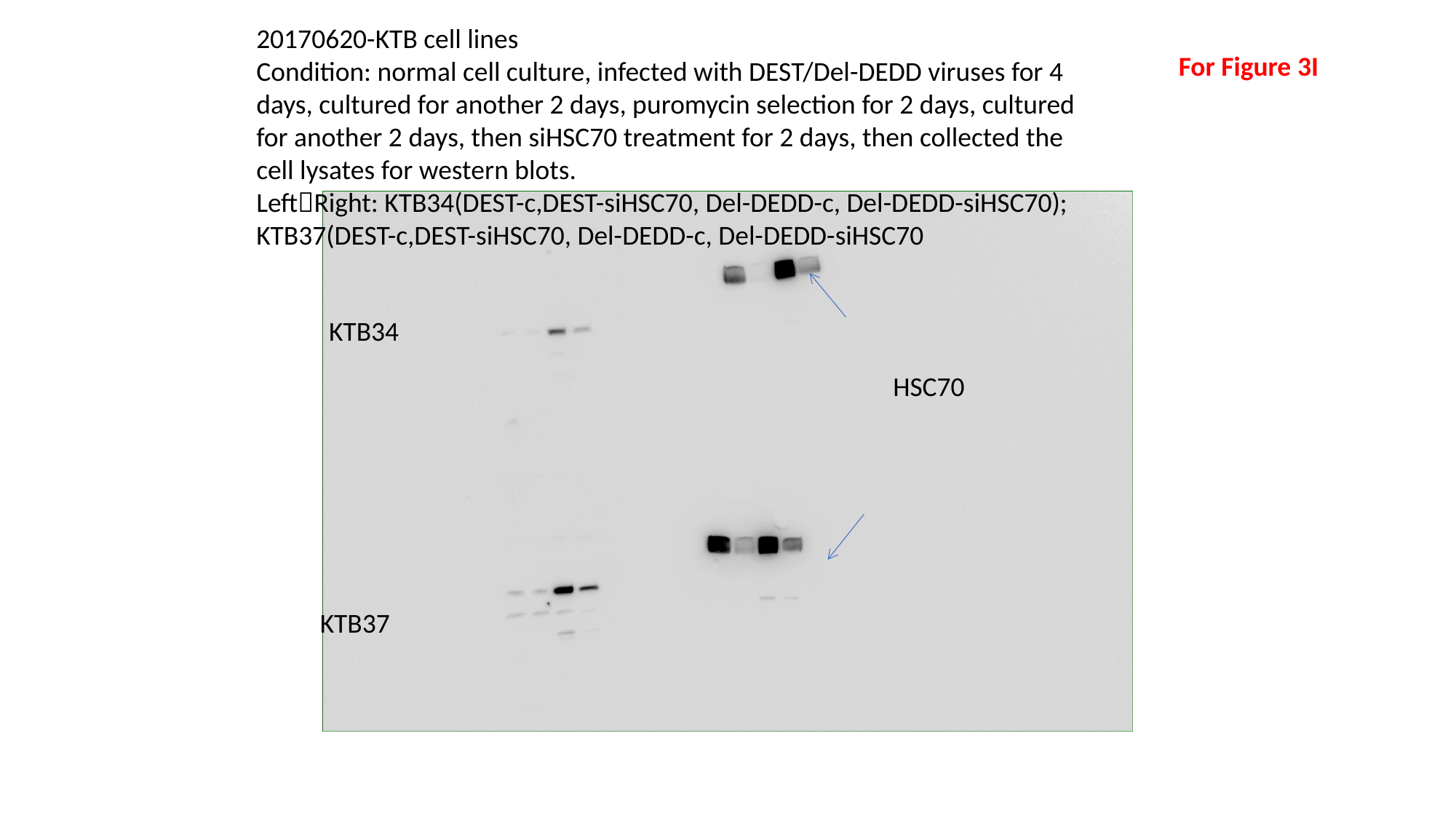

20170620-KTB cell lines
Condition: normal cell culture, infected with DEST/Del-DEDD viruses for 4 days, cultured for another 2 days, puromycin selection for 2 days, cultured for another 2 days, then siHSC70 treatment for 2 days, then collected the cell lysates for western blots.
LeftRight: KTB34(DEST-c,DEST-siHSC70, Del-DEDD-c, Del-DEDD-siHSC70); KTB37(DEST-c,DEST-siHSC70, Del-DEDD-c, Del-DEDD-siHSC70
For Figure 3I
KTB34
HSC70
KTB37

## Slide 24
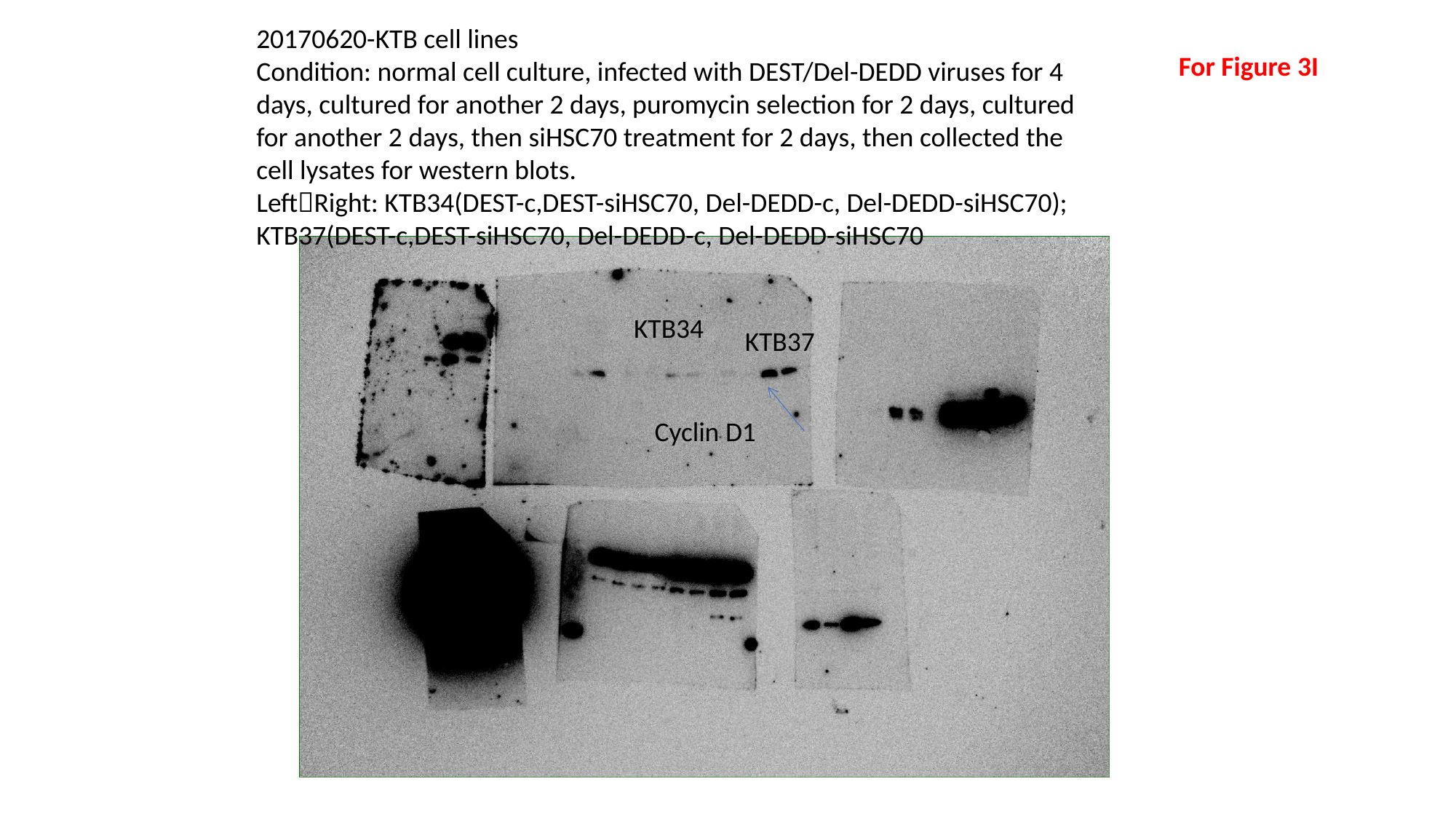

20170620-KTB cell lines
Condition: normal cell culture, infected with DEST/Del-DEDD viruses for 4 days, cultured for another 2 days, puromycin selection for 2 days, cultured for another 2 days, then siHSC70 treatment for 2 days, then collected the cell lysates for western blots.
LeftRight: KTB34(DEST-c,DEST-siHSC70, Del-DEDD-c, Del-DEDD-siHSC70); KTB37(DEST-c,DEST-siHSC70, Del-DEDD-c, Del-DEDD-siHSC70
For Figure 3I
KTB34
KTB37
Cyclin D1

## Slide 25
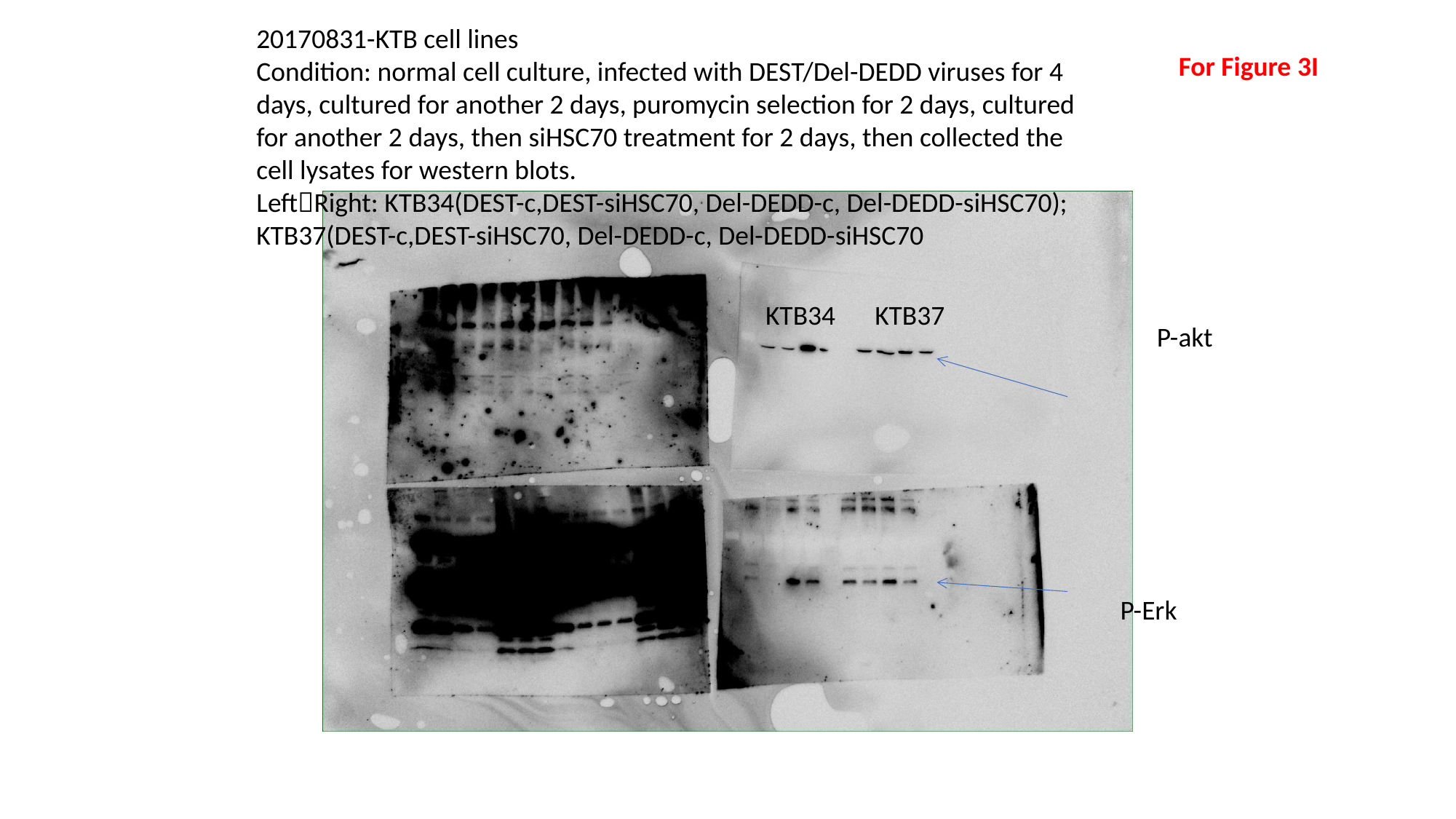

20170831-KTB cell lines
Condition: normal cell culture, infected with DEST/Del-DEDD viruses for 4 days, cultured for another 2 days, puromycin selection for 2 days, cultured for another 2 days, then siHSC70 treatment for 2 days, then collected the cell lysates for western blots.
LeftRight: KTB34(DEST-c,DEST-siHSC70, Del-DEDD-c, Del-DEDD-siHSC70); KTB37(DEST-c,DEST-siHSC70, Del-DEDD-c, Del-DEDD-siHSC70
For Figure 3I
KTB34
KTB37
P-akt
P-Erk

## Slide 26
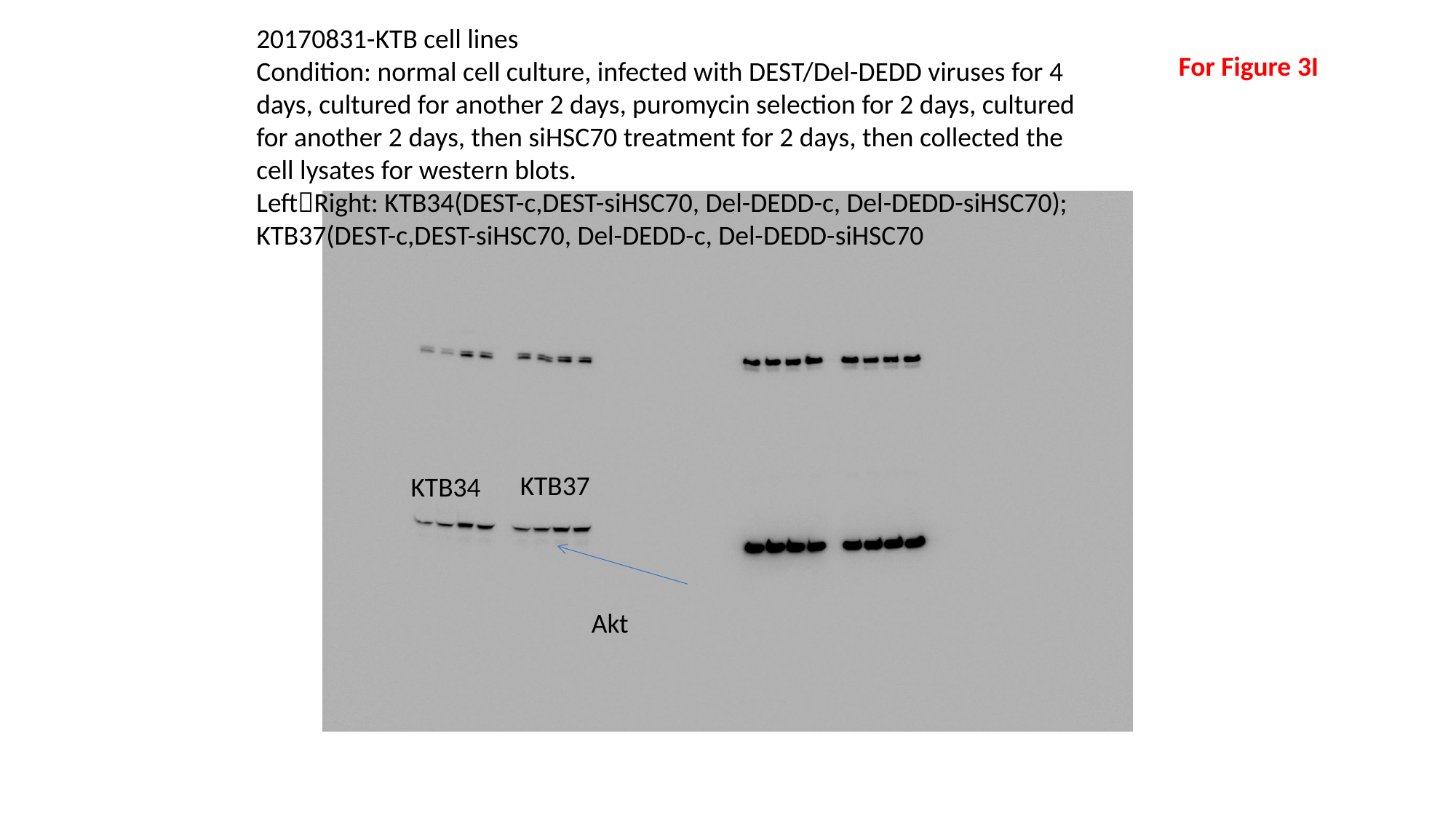

20170831-KTB cell lines
Condition: normal cell culture, infected with DEST/Del-DEDD viruses for 4 days, cultured for another 2 days, puromycin selection for 2 days, cultured for another 2 days, then siHSC70 treatment for 2 days, then collected the cell lysates for western blots.
LeftRight: KTB34(DEST-c,DEST-siHSC70, Del-DEDD-c, Del-DEDD-siHSC70); KTB37(DEST-c,DEST-siHSC70, Del-DEDD-c, Del-DEDD-siHSC70
For Figure 3I
KTB37
KTB34
Akt

## Slide 27
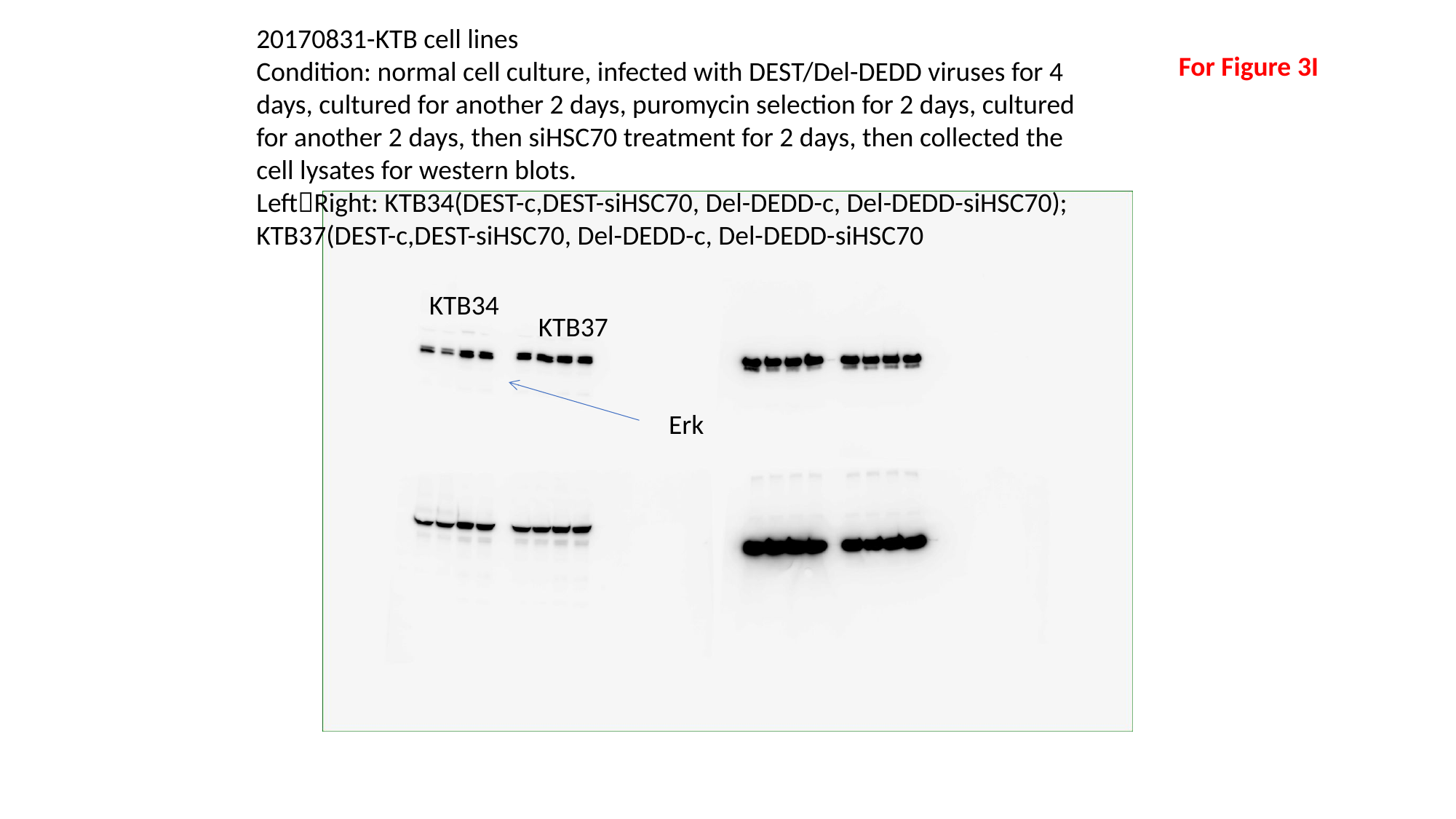

20170831-KTB cell lines
Condition: normal cell culture, infected with DEST/Del-DEDD viruses for 4 days, cultured for another 2 days, puromycin selection for 2 days, cultured for another 2 days, then siHSC70 treatment for 2 days, then collected the cell lysates for western blots.
LeftRight: KTB34(DEST-c,DEST-siHSC70, Del-DEDD-c, Del-DEDD-siHSC70); KTB37(DEST-c,DEST-siHSC70, Del-DEDD-c, Del-DEDD-siHSC70
For Figure 3I
KTB34
KTB37
Erk

## Slide 28
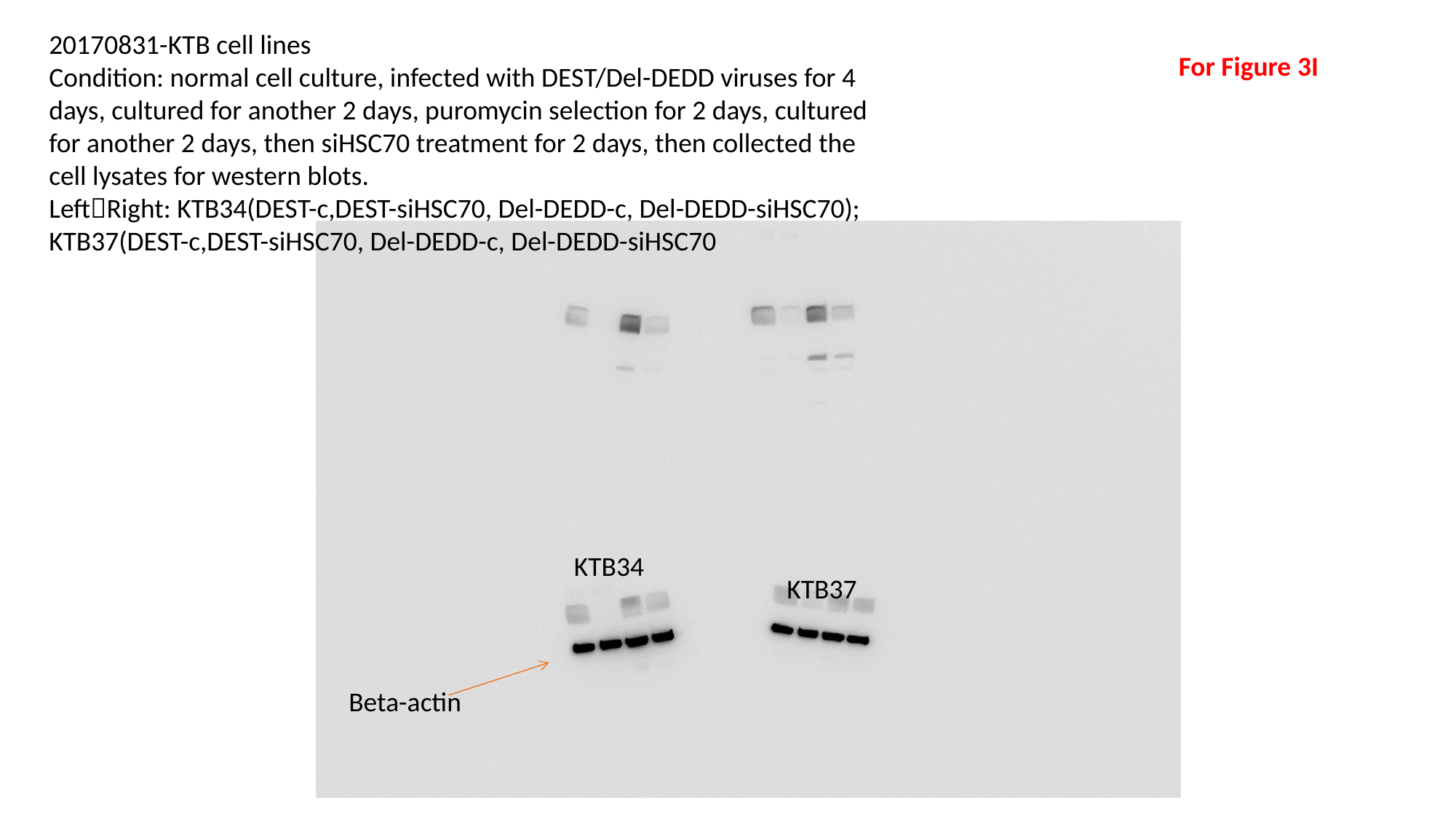

20170831-KTB cell lines
Condition: normal cell culture, infected with DEST/Del-DEDD viruses for 4 days, cultured for another 2 days, puromycin selection for 2 days, cultured for another 2 days, then siHSC70 treatment for 2 days, then collected the cell lysates for western blots.
LeftRight: KTB34(DEST-c,DEST-siHSC70, Del-DEDD-c, Del-DEDD-siHSC70); KTB37(DEST-c,DEST-siHSC70, Del-DEDD-c, Del-DEDD-siHSC70
For Figure 3I
KTB34
KTB37
Beta-actin

## Slide 29
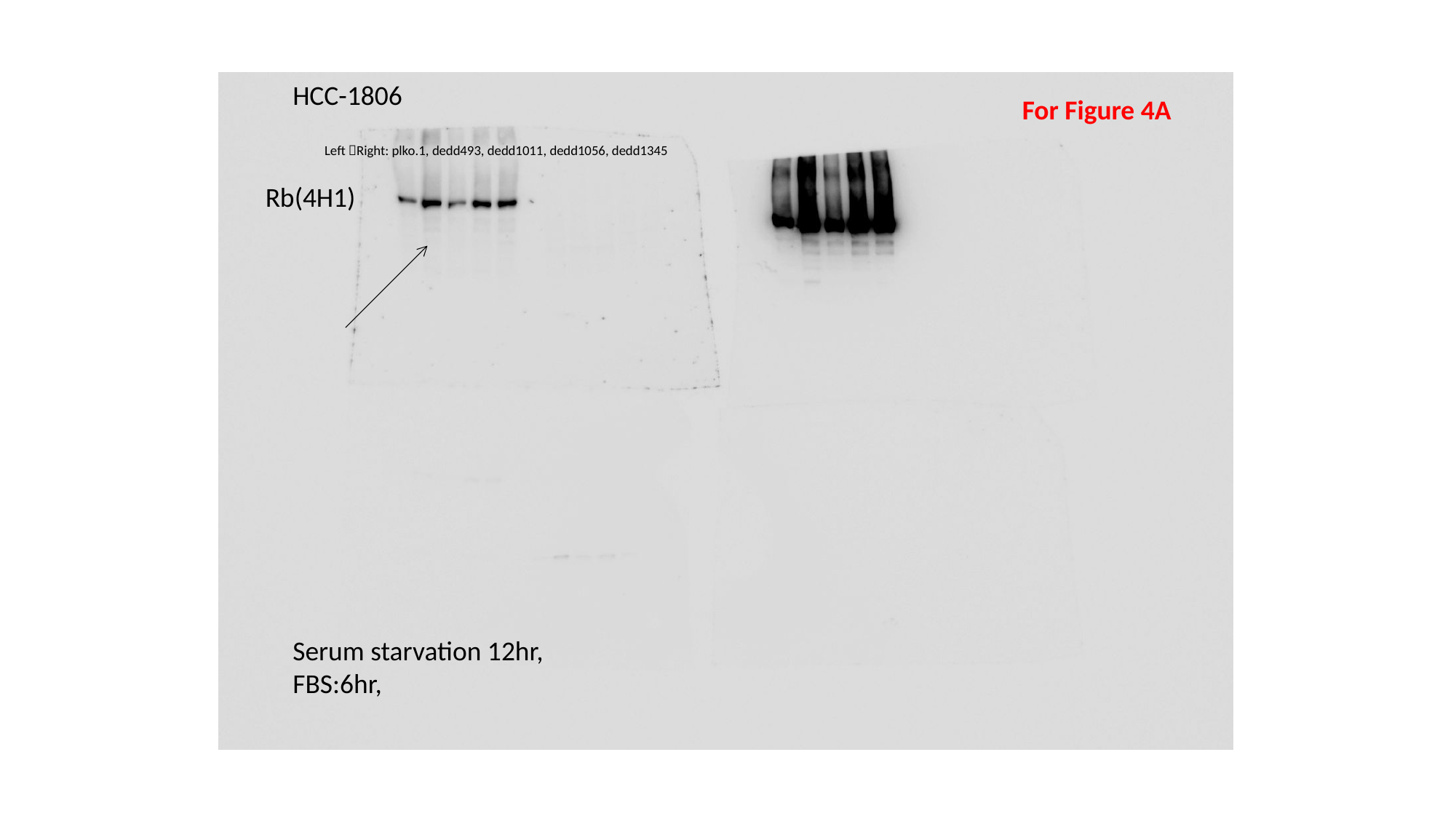

#
HCC-1806
For Figure 4A
Left Right: plko.1, dedd493, dedd1011, dedd1056, dedd1345
Rb(4H1)
Serum starvation 12hr, FBS:6hr,

## Slide 30
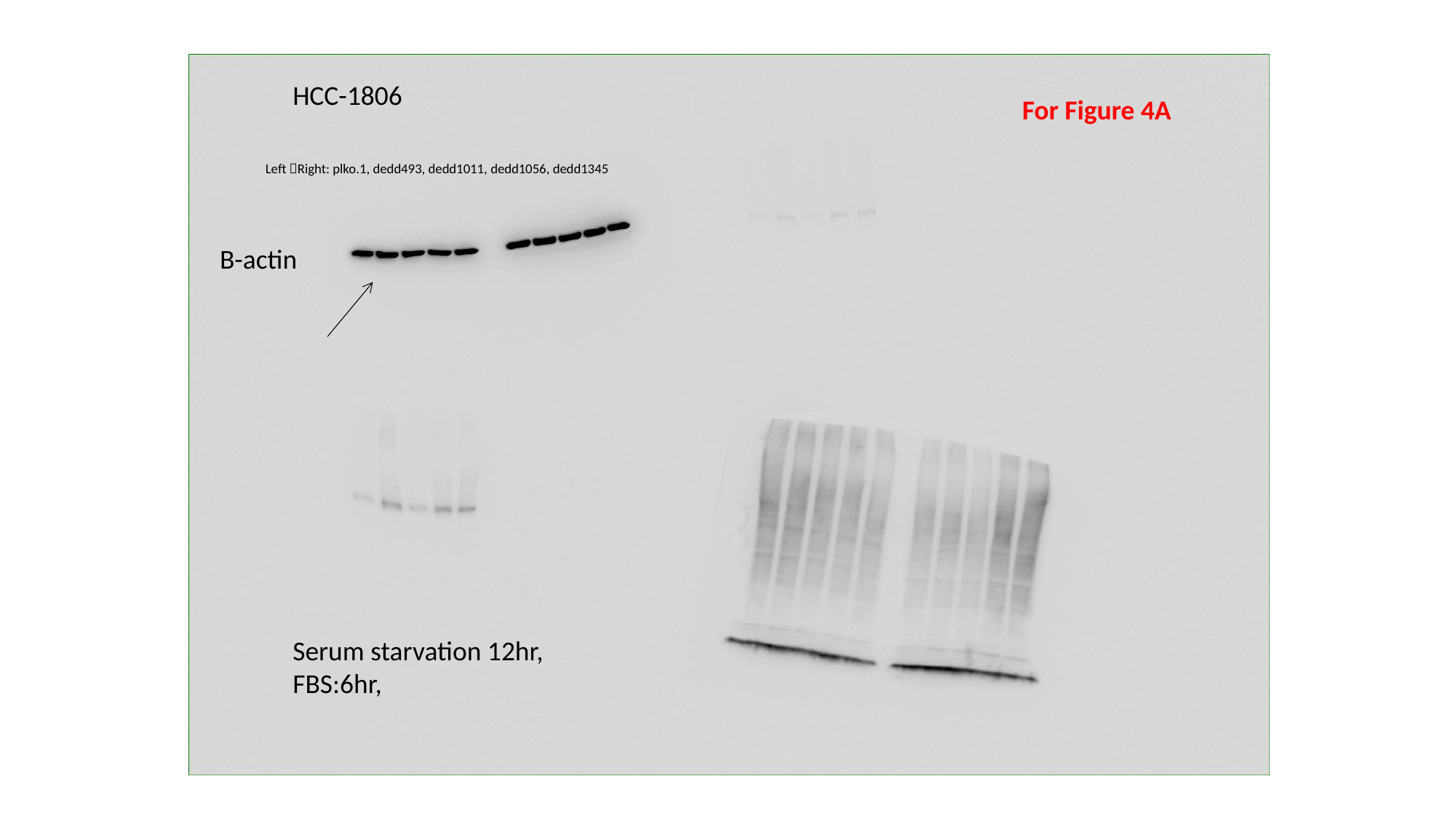

#
HCC-1806
For Figure 4A
Left Right: plko.1, dedd493, dedd1011, dedd1056, dedd1345
B-actin
Serum starvation 12hr, FBS:6hr,

## Slide 31
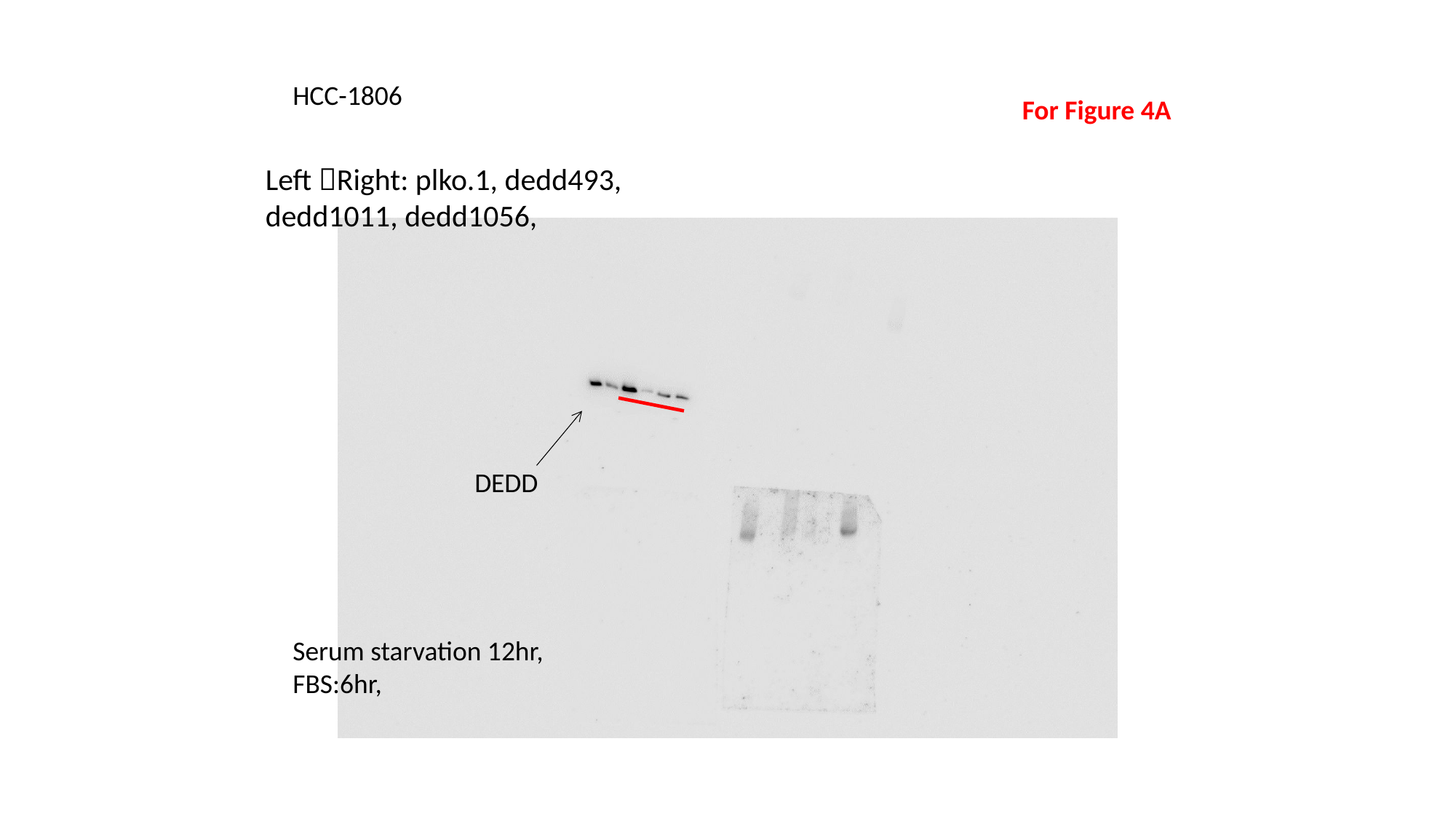

HCC-1806
For Figure 4A
Left Right: plko.1, dedd493, dedd1011, dedd1056,
DEDD
Serum starvation 12hr, FBS:6hr,

## Slide 32
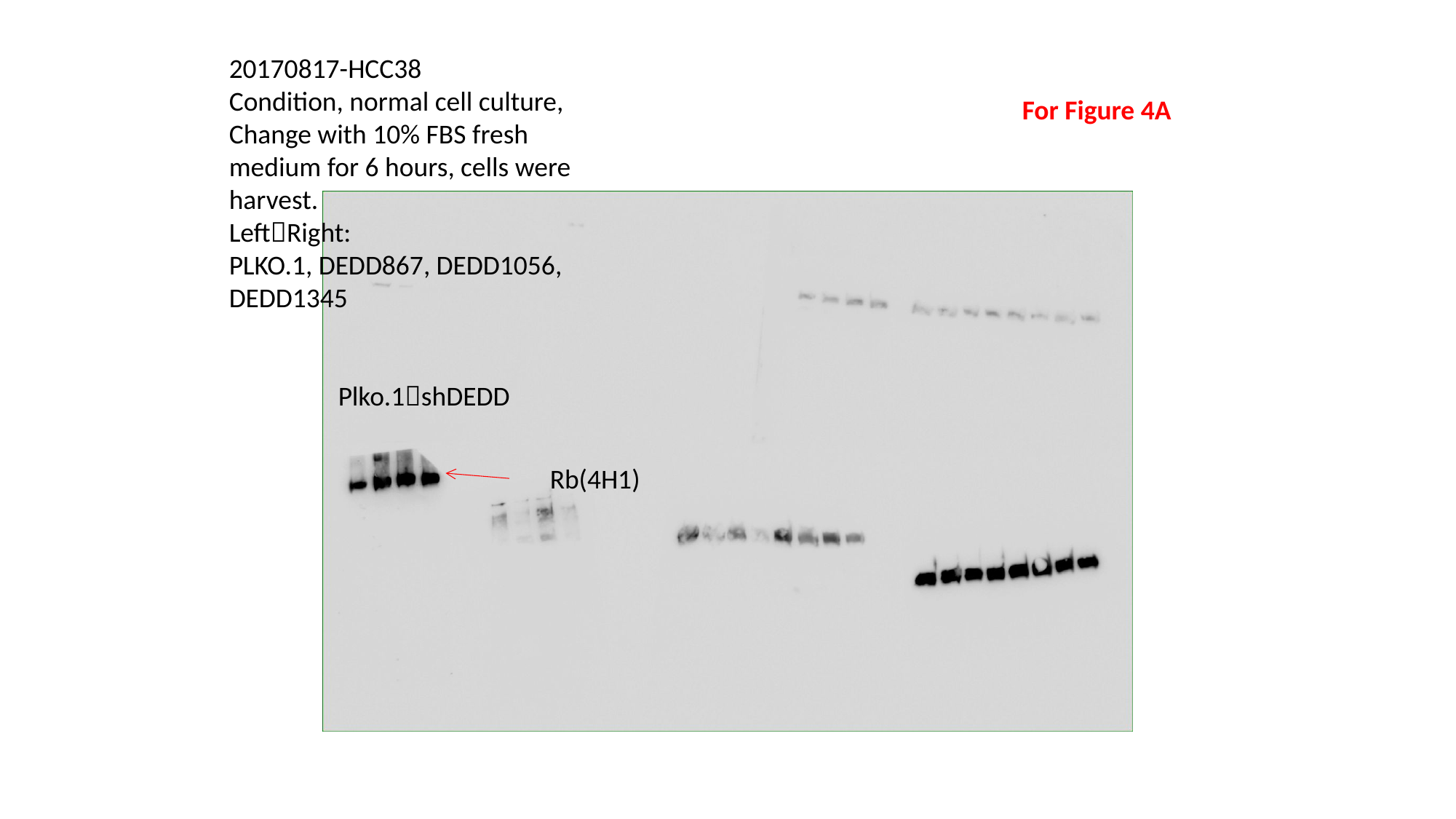

20170817-HCC38
Condition, normal cell culture, Change with 10% FBS fresh medium for 6 hours, cells were harvest.
LeftRight:
PLKO.1, DEDD867, DEDD1056, DEDD1345
For Figure 4A
Plko.1shDEDD
Rb(4H1)

## Slide 33
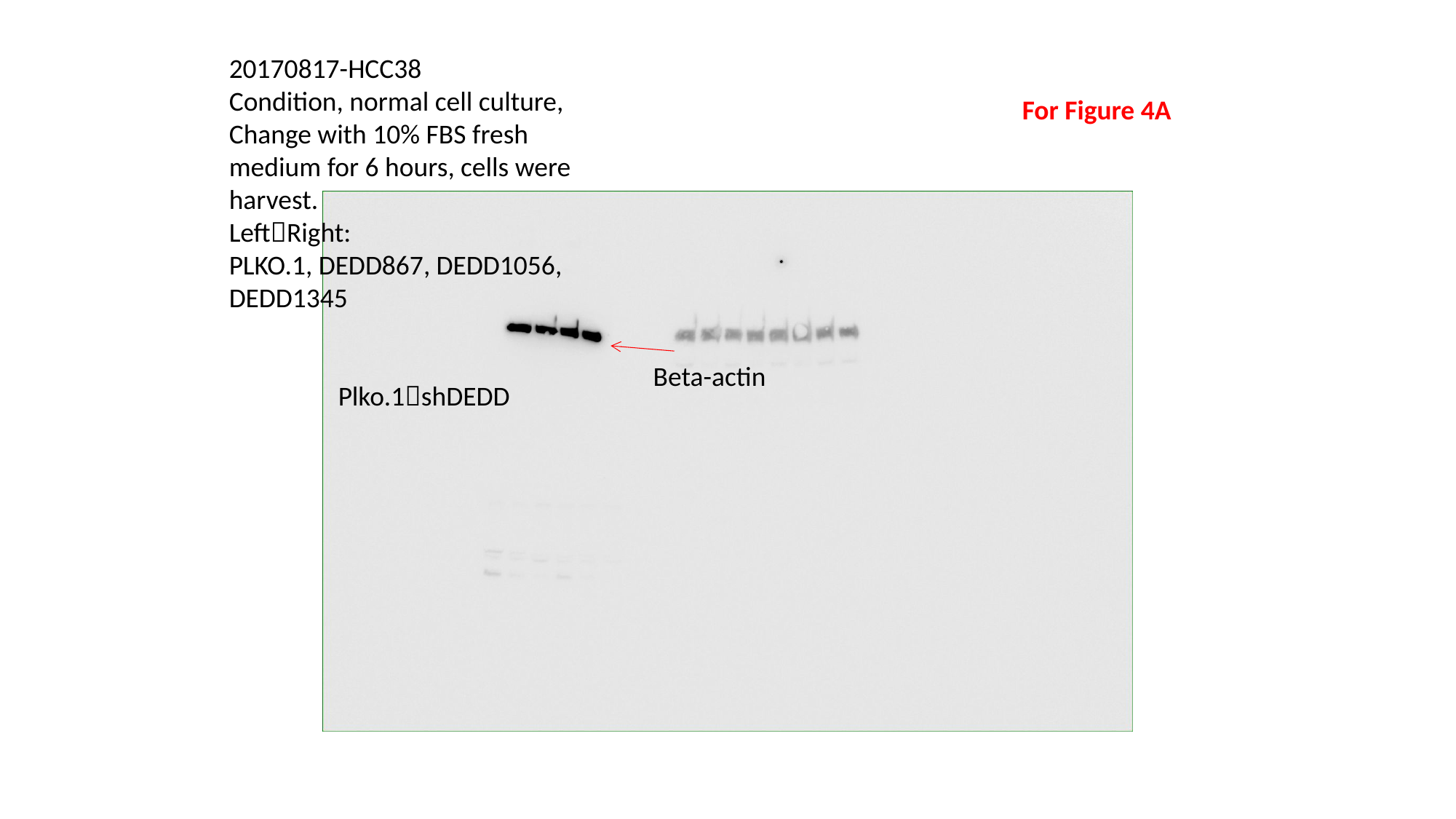

#
20170817-HCC38
Condition, normal cell culture, Change with 10% FBS fresh medium for 6 hours, cells were harvest.
LeftRight:
PLKO.1, DEDD867, DEDD1056, DEDD1345
For Figure 4A
Beta-actin
Plko.1shDEDD

## Slide 34
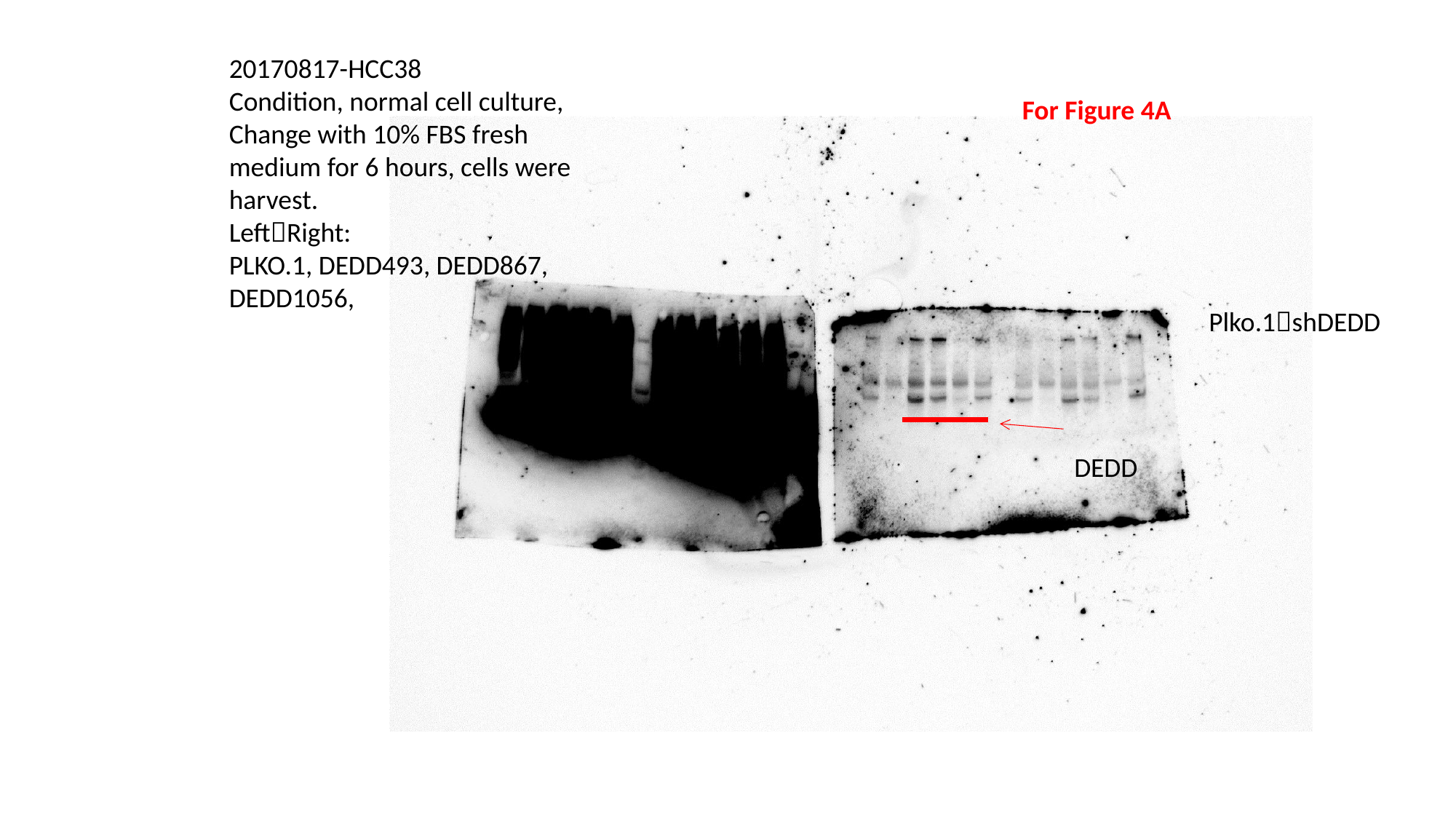

20170817-HCC38
Condition, normal cell culture, Change with 10% FBS fresh medium for 6 hours, cells were harvest.
LeftRight:
PLKO.1, DEDD493, DEDD867, DEDD1056,
For Figure 4A
Plko.1shDEDD
DEDD

## Slide 35
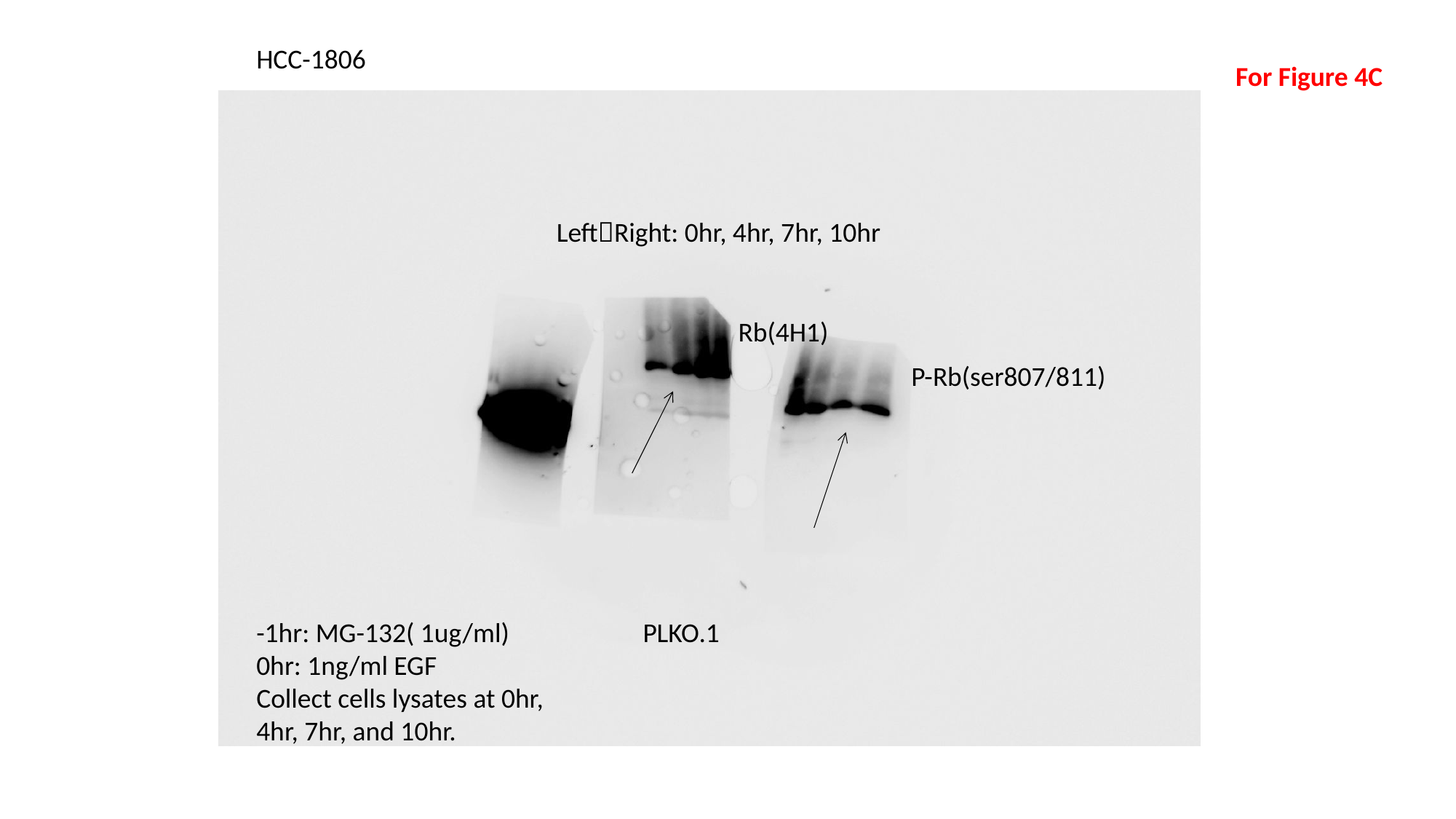

HCC-1806
For Figure 4C
#
LeftRight: 0hr, 4hr, 7hr, 10hr
Rb(4H1)
P-Rb(ser807/811)
PLKO.1
-1hr: MG-132( 1ug/ml)
0hr: 1ng/ml EGF
Collect cells lysates at 0hr, 4hr, 7hr, and 10hr.

## Slide 36
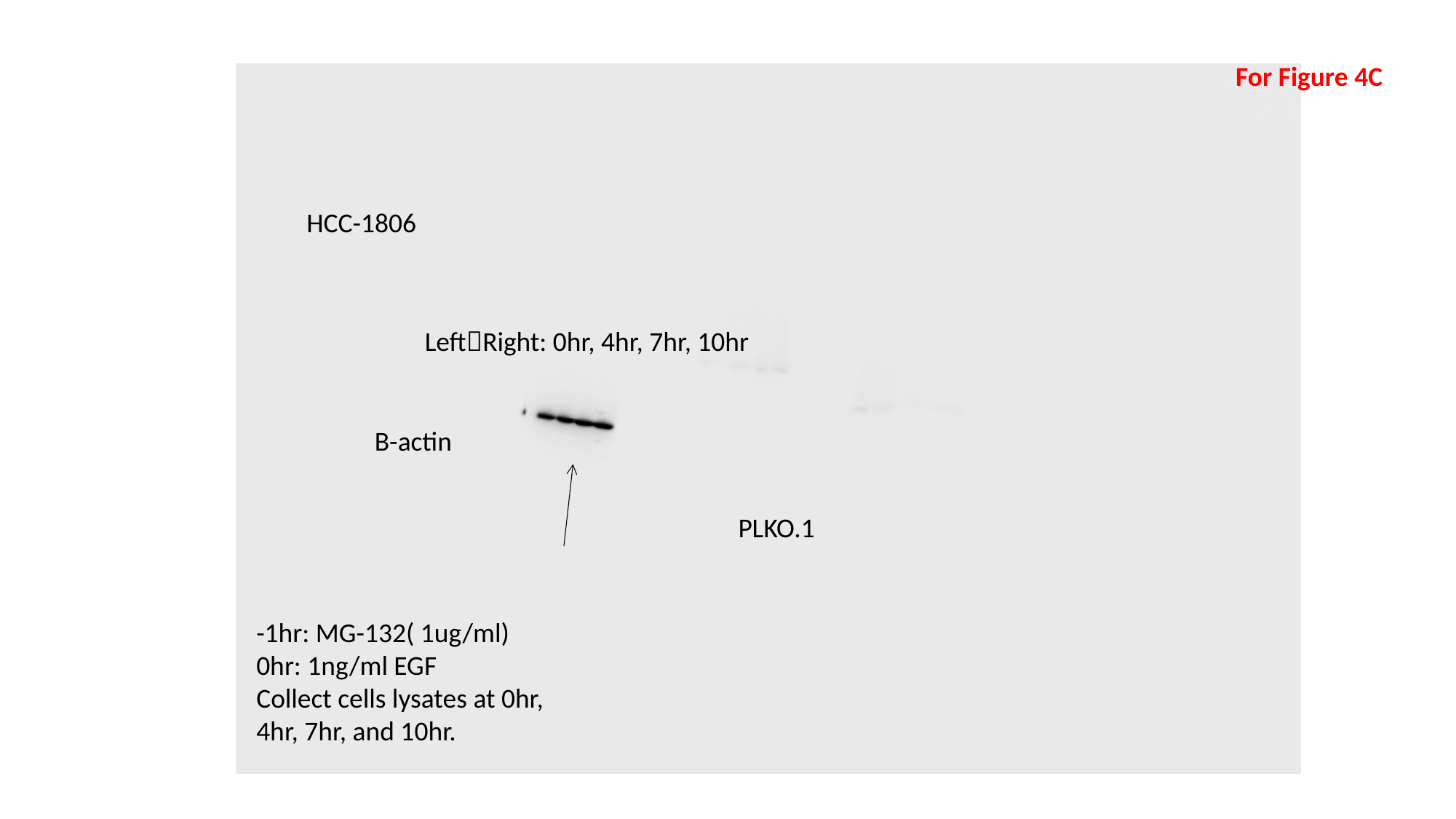

#
For Figure 4C
HCC-1806
LeftRight: 0hr, 4hr, 7hr, 10hr
B-actin
PLKO.1
-1hr: MG-132( 1ug/ml)
0hr: 1ng/ml EGF
Collect cells lysates at 0hr, 4hr, 7hr, and 10hr.

## Slide 37
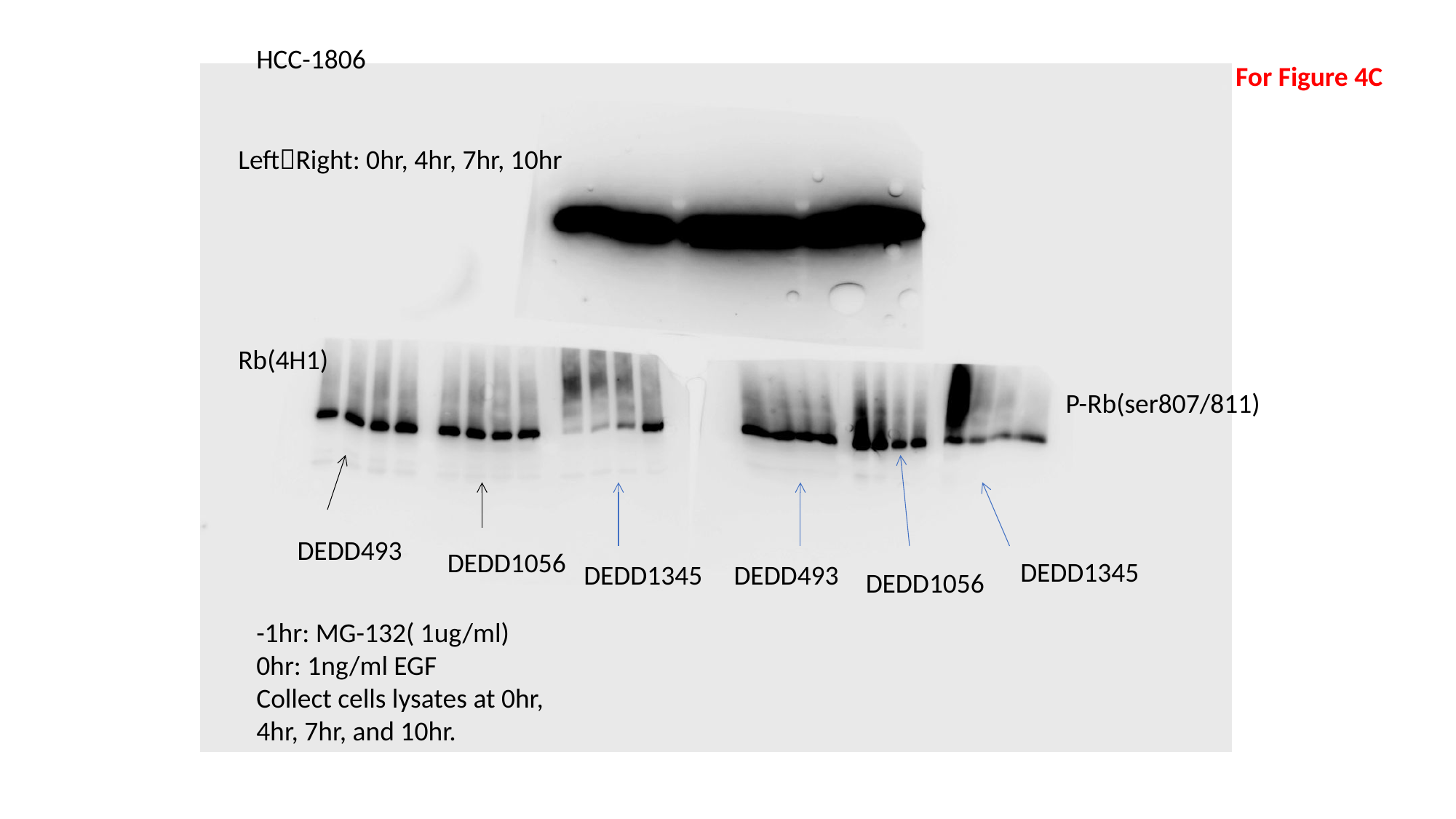

HCC-1806
#
For Figure 4C
LeftRight: 0hr, 4hr, 7hr, 10hr
Rb(4H1)
P-Rb(ser807/811)
DEDD493
DEDD1056
DEDD1345
DEDD1345
DEDD493
DEDD1056
-1hr: MG-132( 1ug/ml)
0hr: 1ng/ml EGF
Collect cells lysates at 0hr, 4hr, 7hr, and 10hr.

## Slide 38
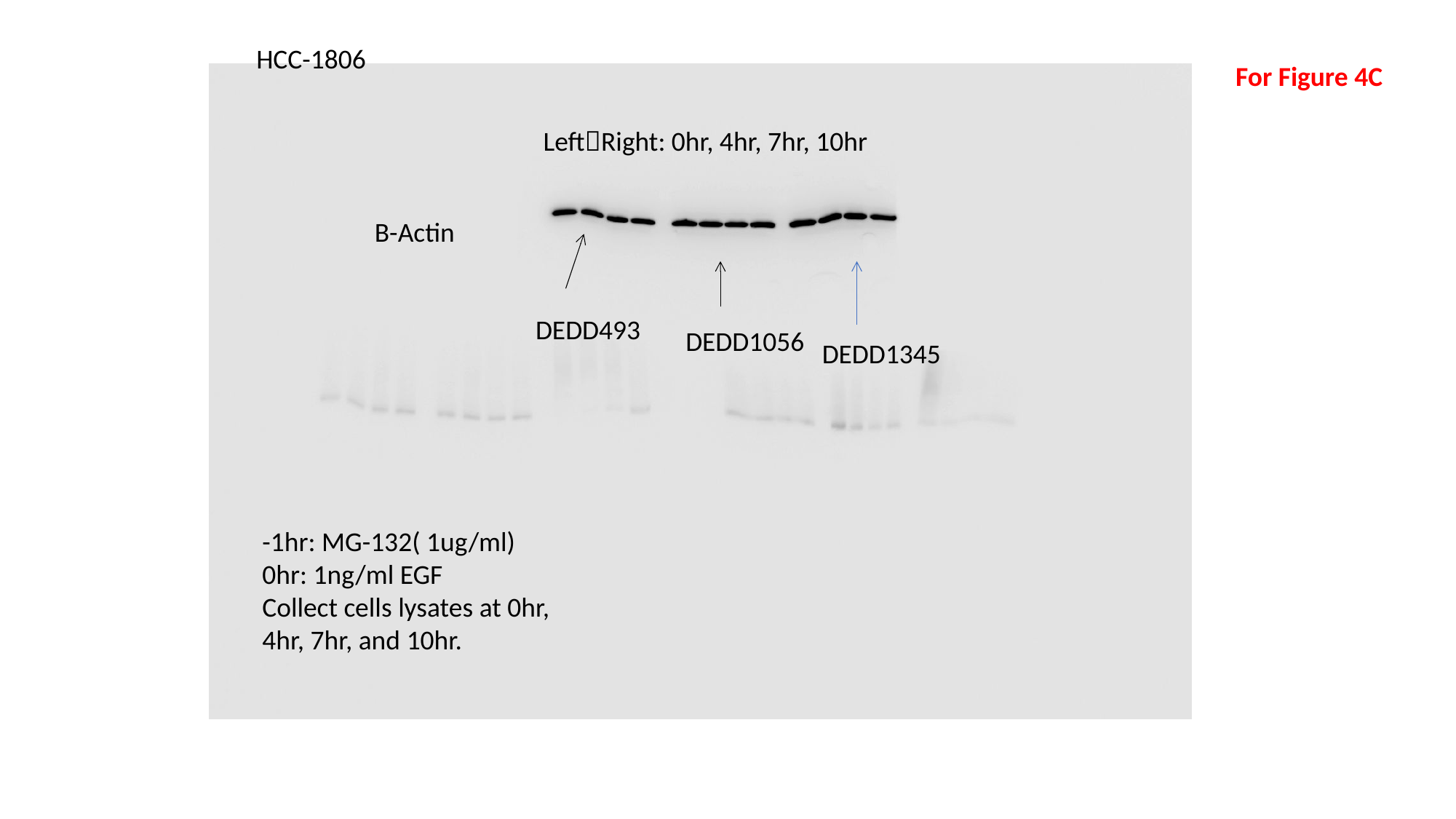

HCC-1806
#
For Figure 4C
LeftRight: 0hr, 4hr, 7hr, 10hr
B-Actin
DEDD493
DEDD1056
DEDD1345
-1hr: MG-132( 1ug/ml)
0hr: 1ng/ml EGF
Collect cells lysates at 0hr, 4hr, 7hr, and 10hr.

## Slide 39
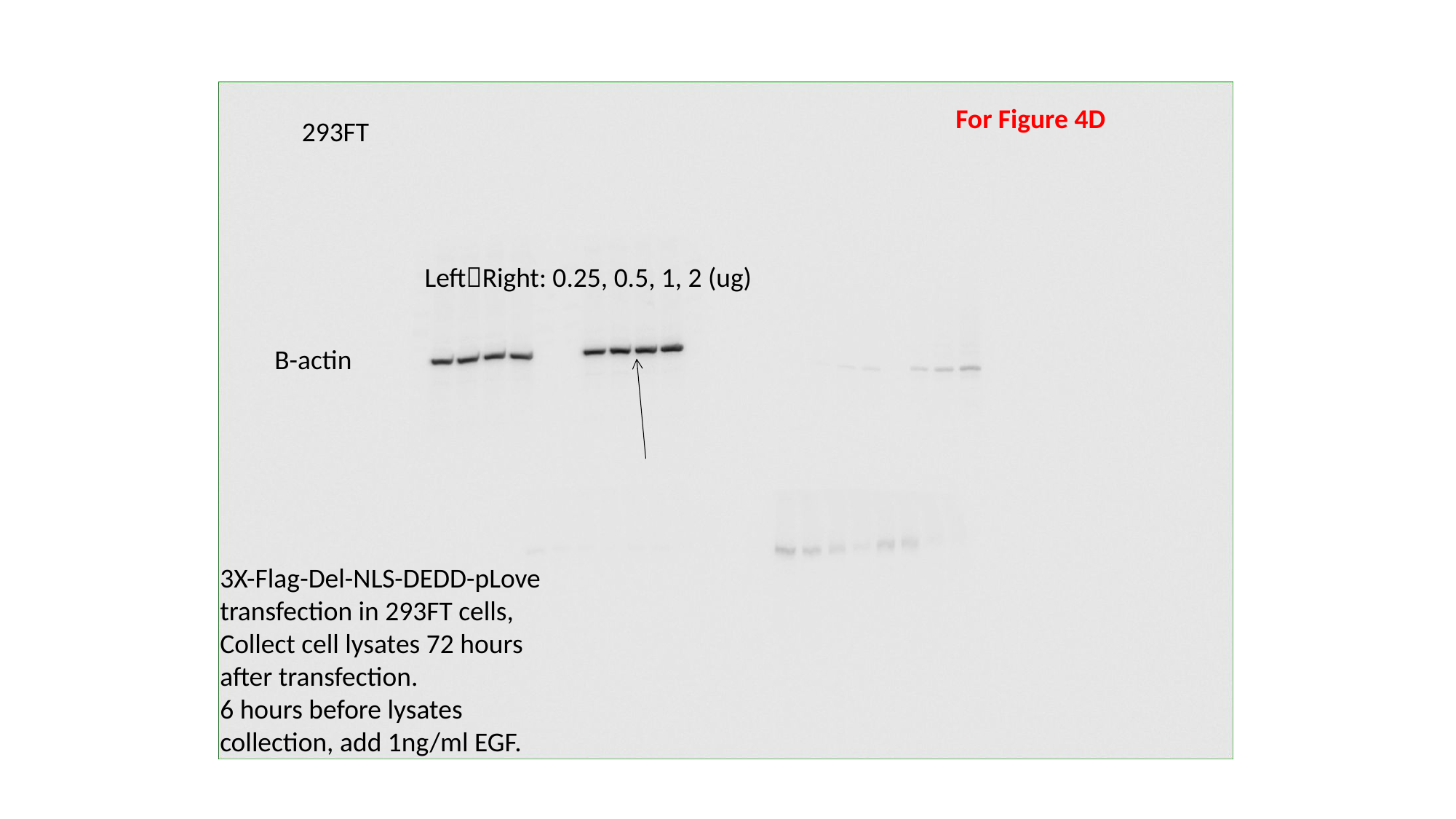

For Figure 4D
293FT
#
LeftRight: 0.25, 0.5, 1, 2 (ug)
B-actin
3X-Flag-Del-NLS-DEDD-pLove transfection in 293FT cells,
Collect cell lysates 72 hours after transfection.
6 hours before lysates collection, add 1ng/ml EGF.

## Slide 40
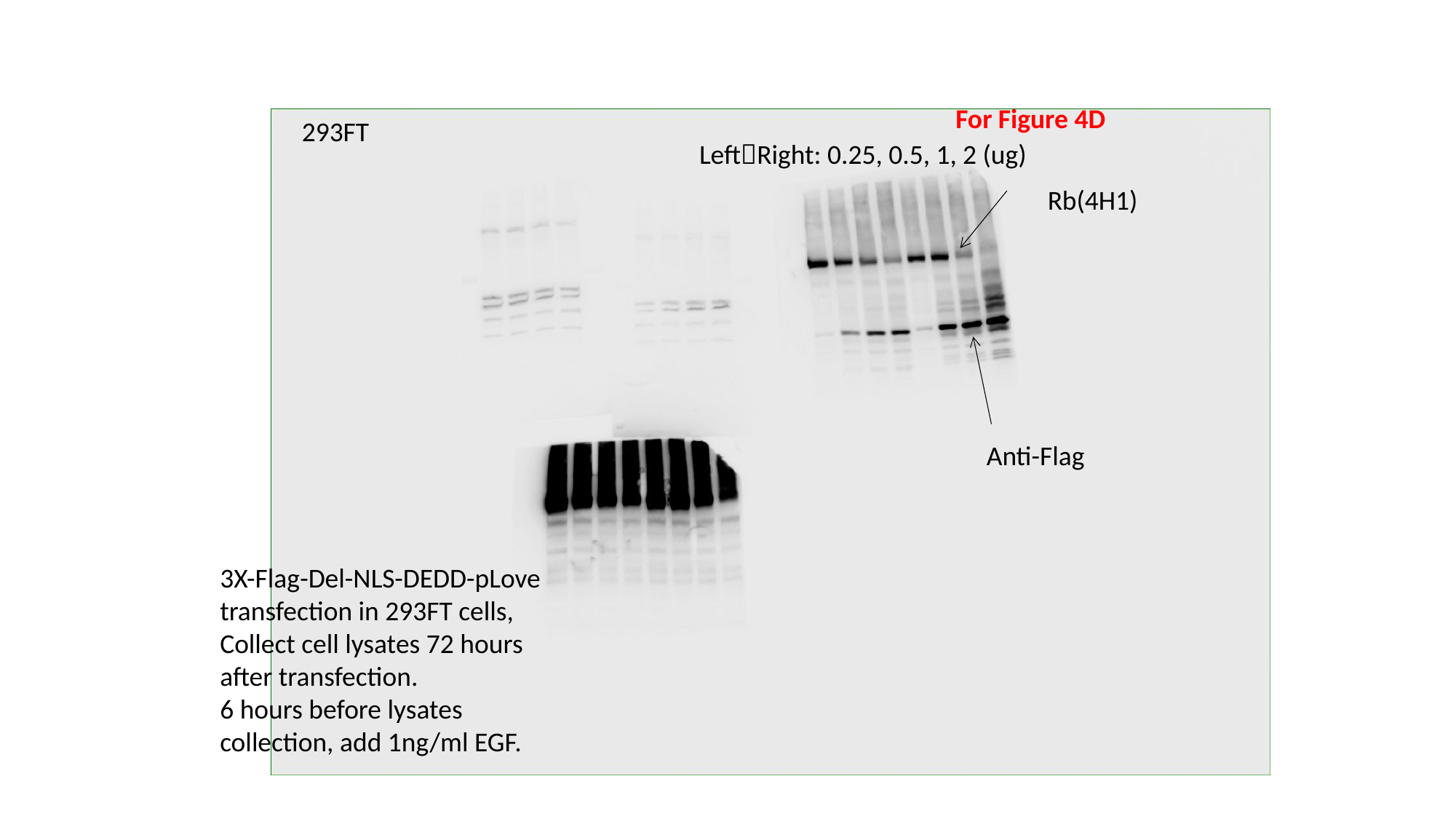

#
 For Figure 4D
293FT
LeftRight: 0.25, 0.5, 1, 2 (ug)
Rb(4H1)
Anti-Flag
3X-Flag-Del-NLS-DEDD-pLove transfection in 293FT cells,
Collect cell lysates 72 hours after transfection.
6 hours before lysates collection, add 1ng/ml EGF.

## Slide 41
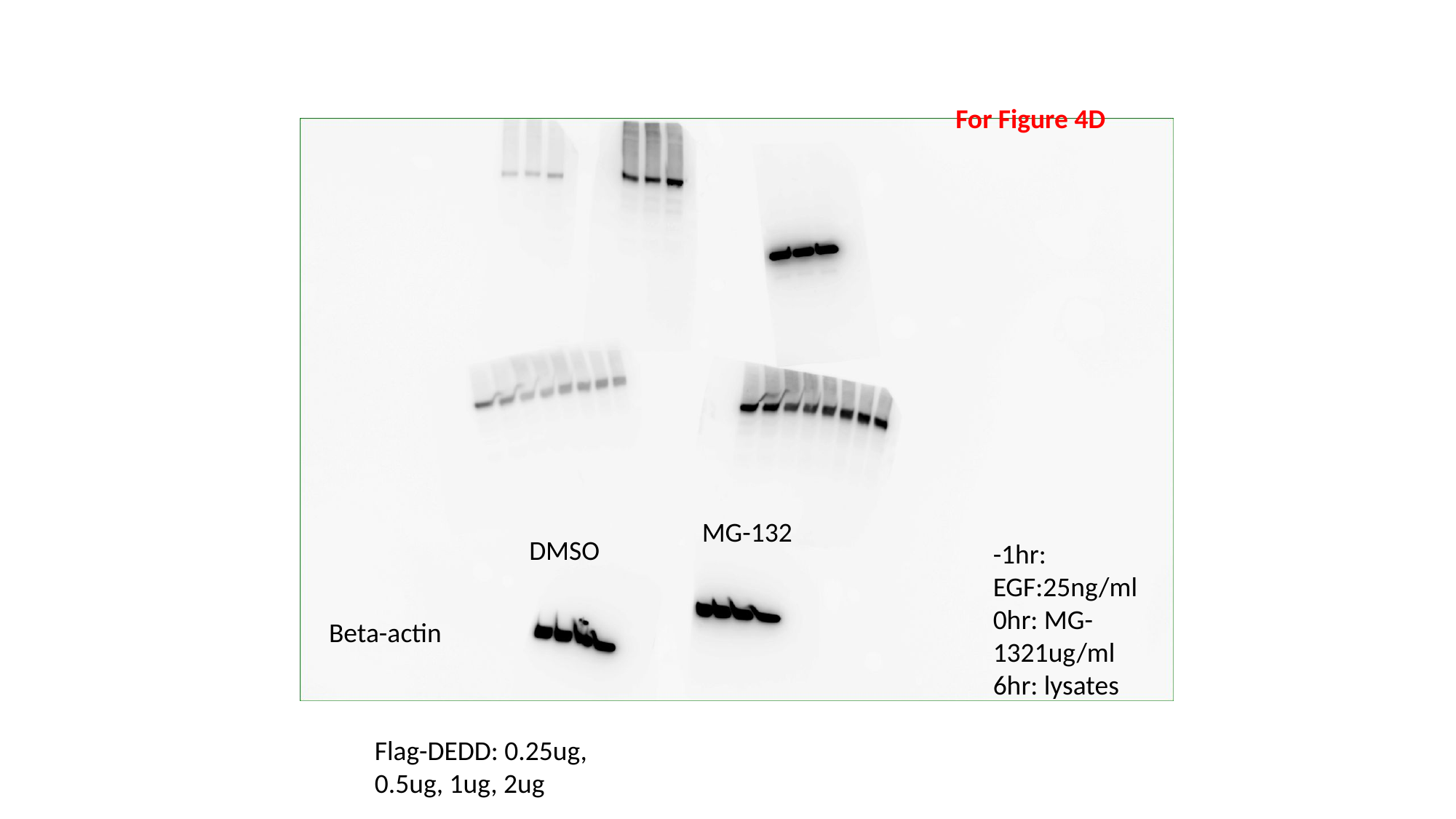

For Figure 4D
#
MG-132
DMSO
-1hr: EGF:25ng/ml
0hr: MG-1321ug/ml
6hr: lysates
Beta-actin
Flag-DEDD: 0.25ug, 0.5ug, 1ug, 2ug

## Slide 42
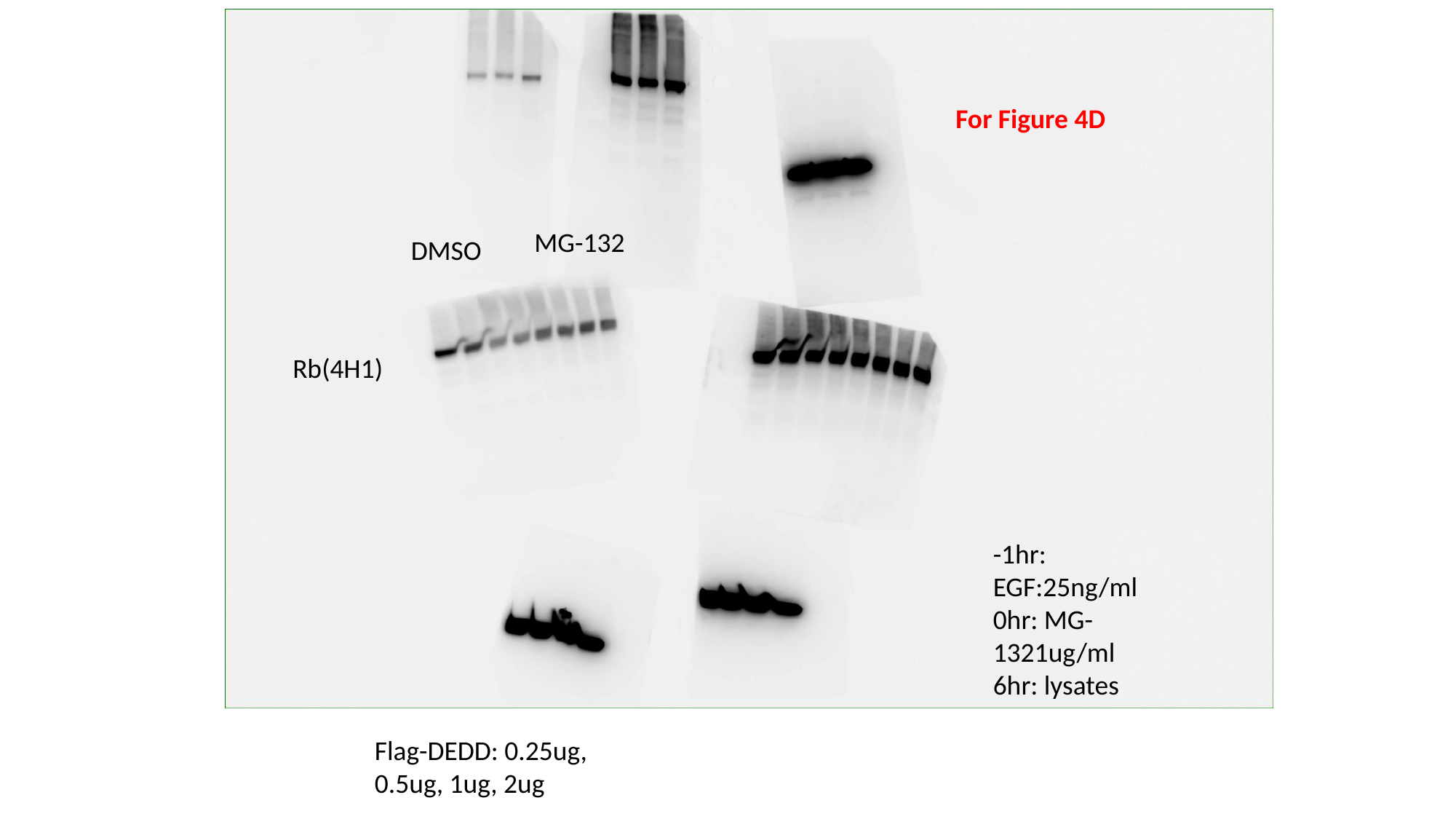

#
 For Figure 4D
MG-132
DMSO
Rb(4H1)
-1hr: EGF:25ng/ml
0hr: MG-1321ug/ml
6hr: lysates
Flag-DEDD: 0.25ug, 0.5ug, 1ug, 2ug

## Slide 43
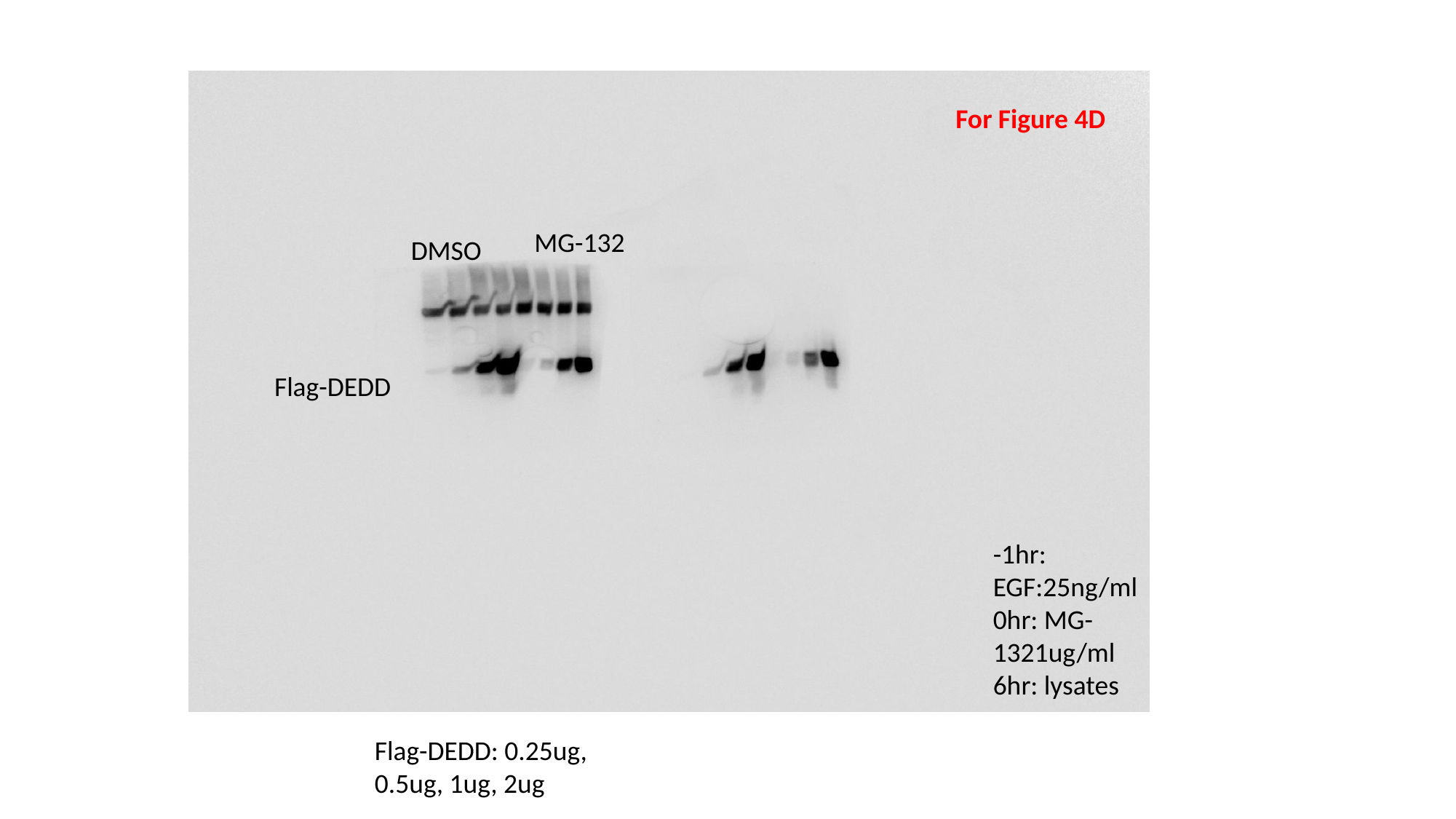

#
 For Figure 4D
MG-132
DMSO
Flag-DEDD
-1hr: EGF:25ng/ml
0hr: MG-1321ug/ml
6hr: lysates
Flag-DEDD: 0.25ug, 0.5ug, 1ug, 2ug

## Slide 44
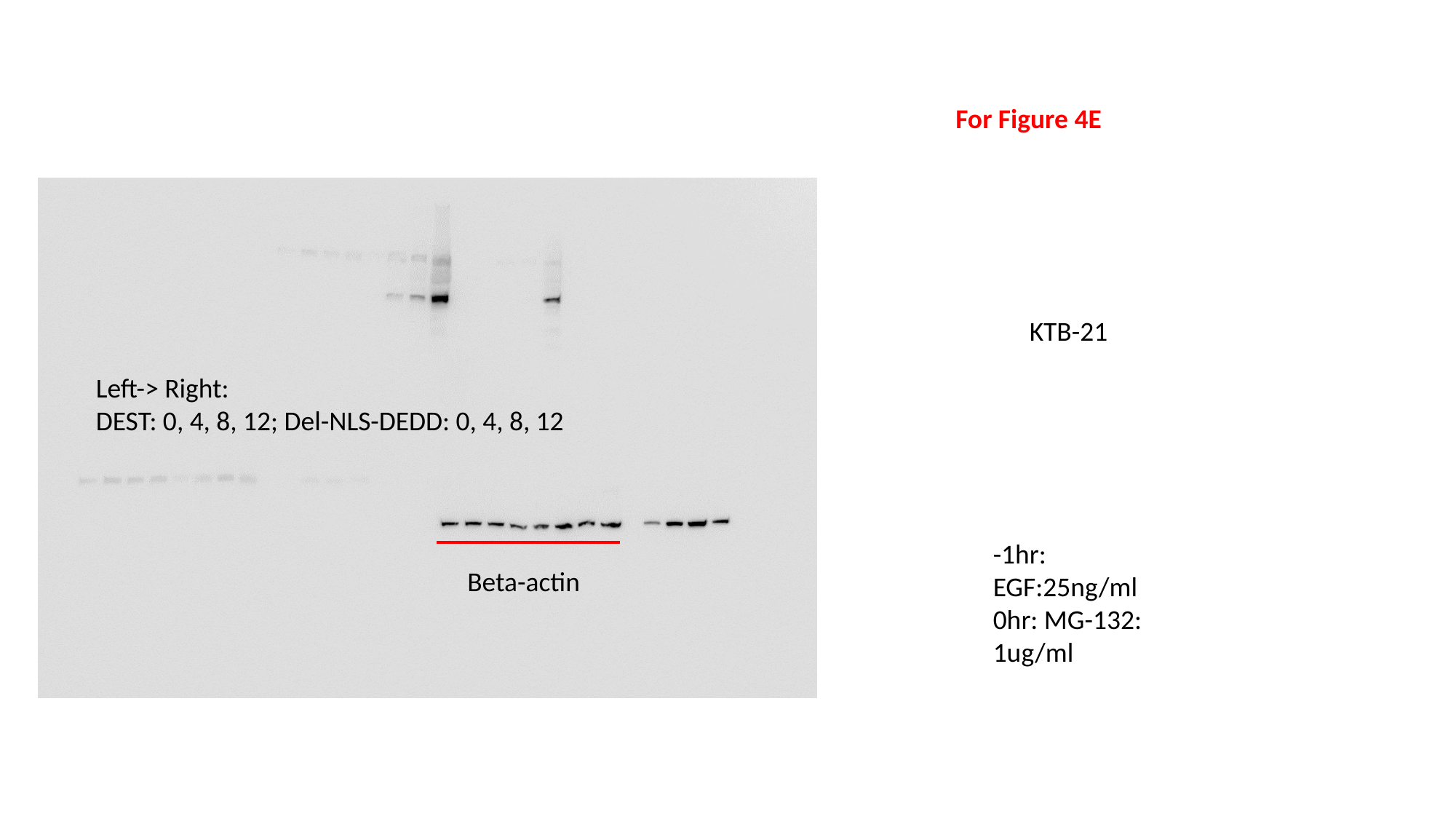

#
 For Figure 4E
KTB-21
Left-> Right:
DEST: 0, 4, 8, 12; Del-NLS-DEDD: 0, 4, 8, 12
-1hr: EGF:25ng/ml
0hr: MG-132: 1ug/ml
Beta-actin

## Slide 45
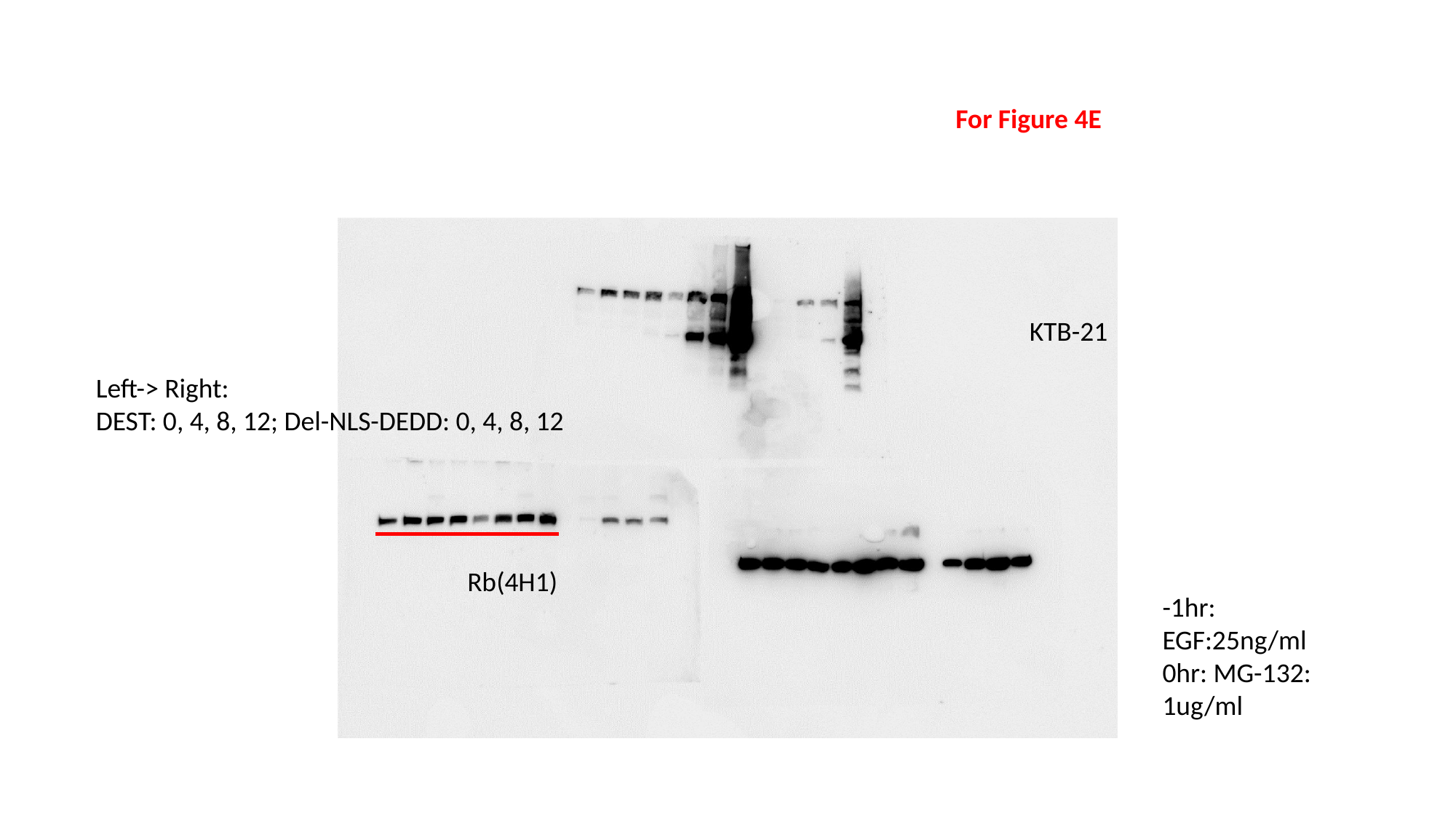

#
 For Figure 4E
KTB-21
Left-> Right:
DEST: 0, 4, 8, 12; Del-NLS-DEDD: 0, 4, 8, 12
Rb(4H1)
-1hr: EGF:25ng/ml
0hr: MG-132: 1ug/ml

## Slide 46
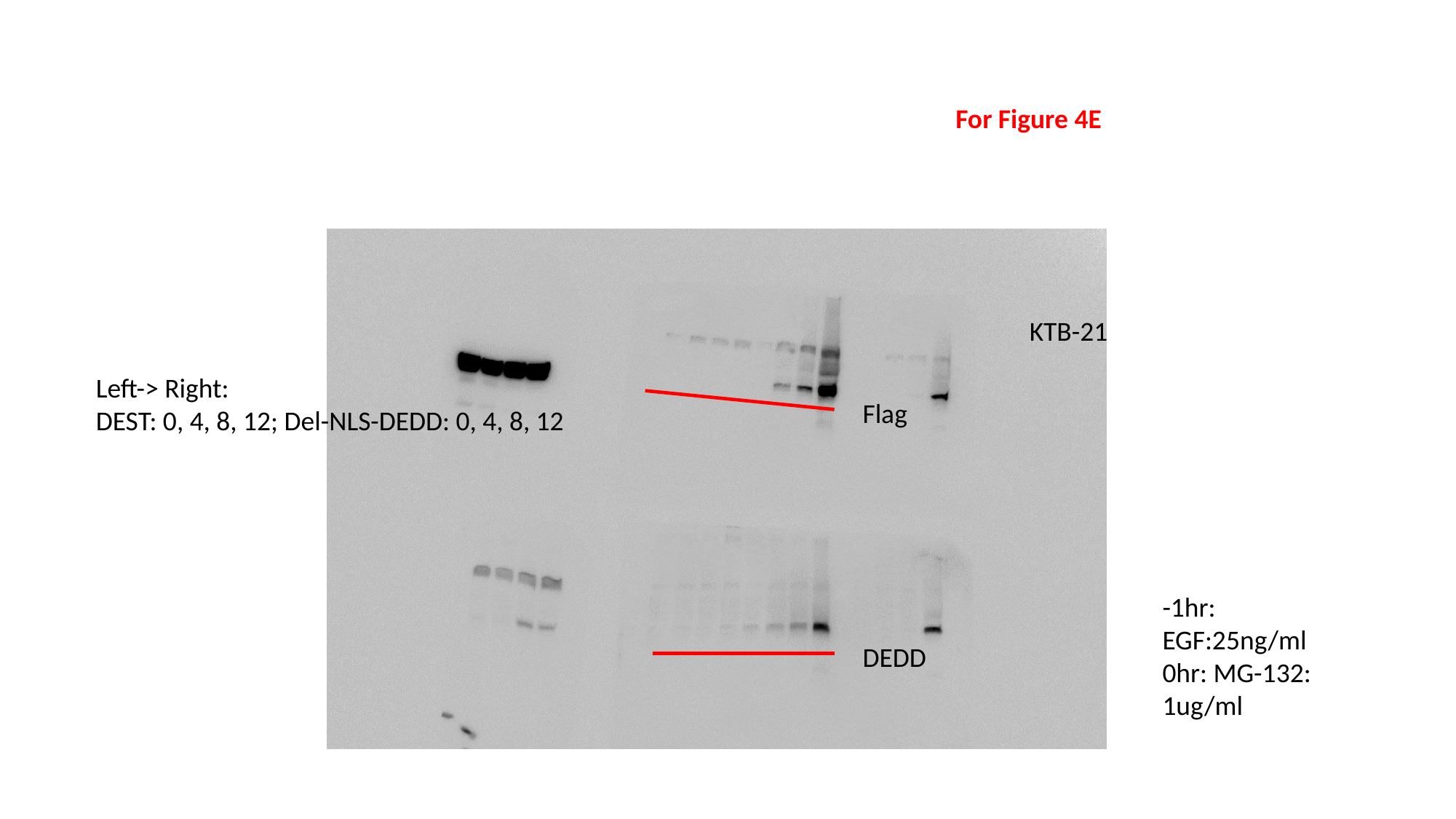

#
 For Figure 4E
KTB-21
Left-> Right:
DEST: 0, 4, 8, 12; Del-NLS-DEDD: 0, 4, 8, 12
Flag
-1hr: EGF:25ng/ml
0hr: MG-132: 1ug/ml
DEDD

## Slide 47
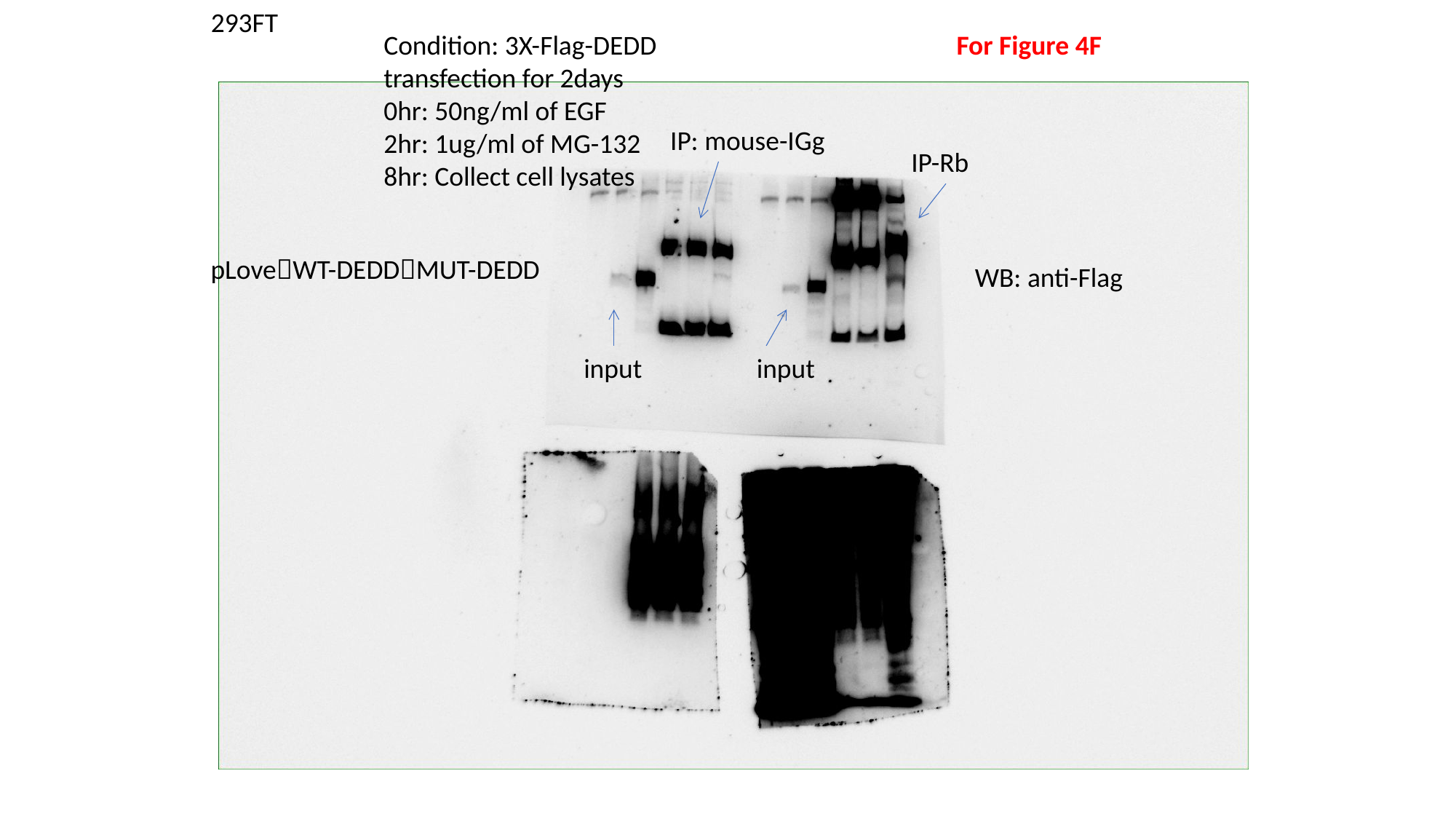

293FT
Condition: 3X-Flag-DEDD transfection for 2days
0hr: 50ng/ml of EGF
2hr: 1ug/ml of MG-132
8hr: Collect cell lysates
For Figure 4F
IP: mouse-IGg
IP-Rb
pLoveWT-DEDDMUT-DEDD
WB: anti-Flag
input
input

## Slide 48
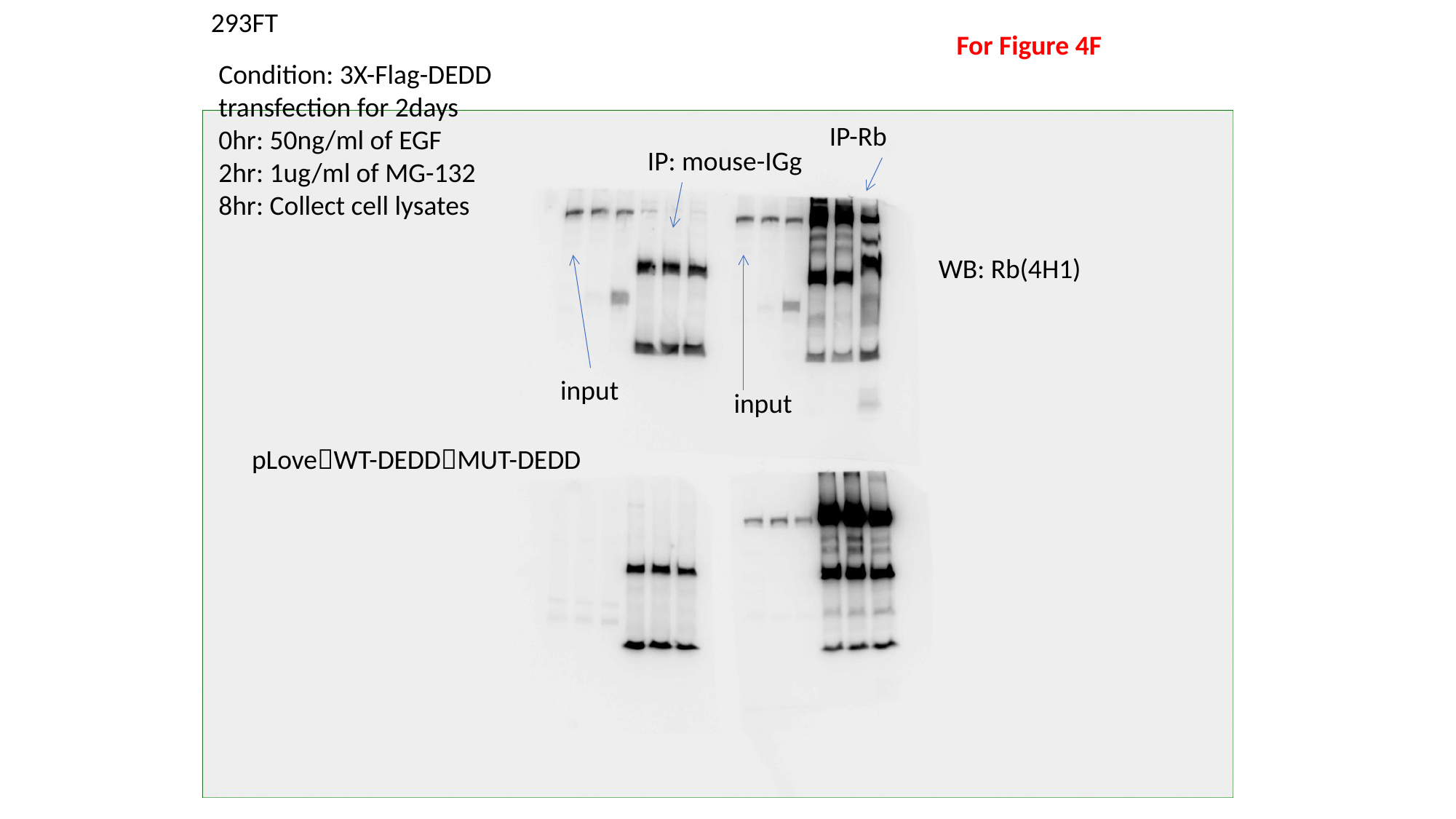

293FT
For Figure 4F
Condition: 3X-Flag-DEDD transfection for 2days
0hr: 50ng/ml of EGF
2hr: 1ug/ml of MG-132
8hr: Collect cell lysates
IP-Rb
#
IP: mouse-IGg
WB: Rb(4H1)
input
input
pLoveWT-DEDDMUT-DEDD

## Slide 49
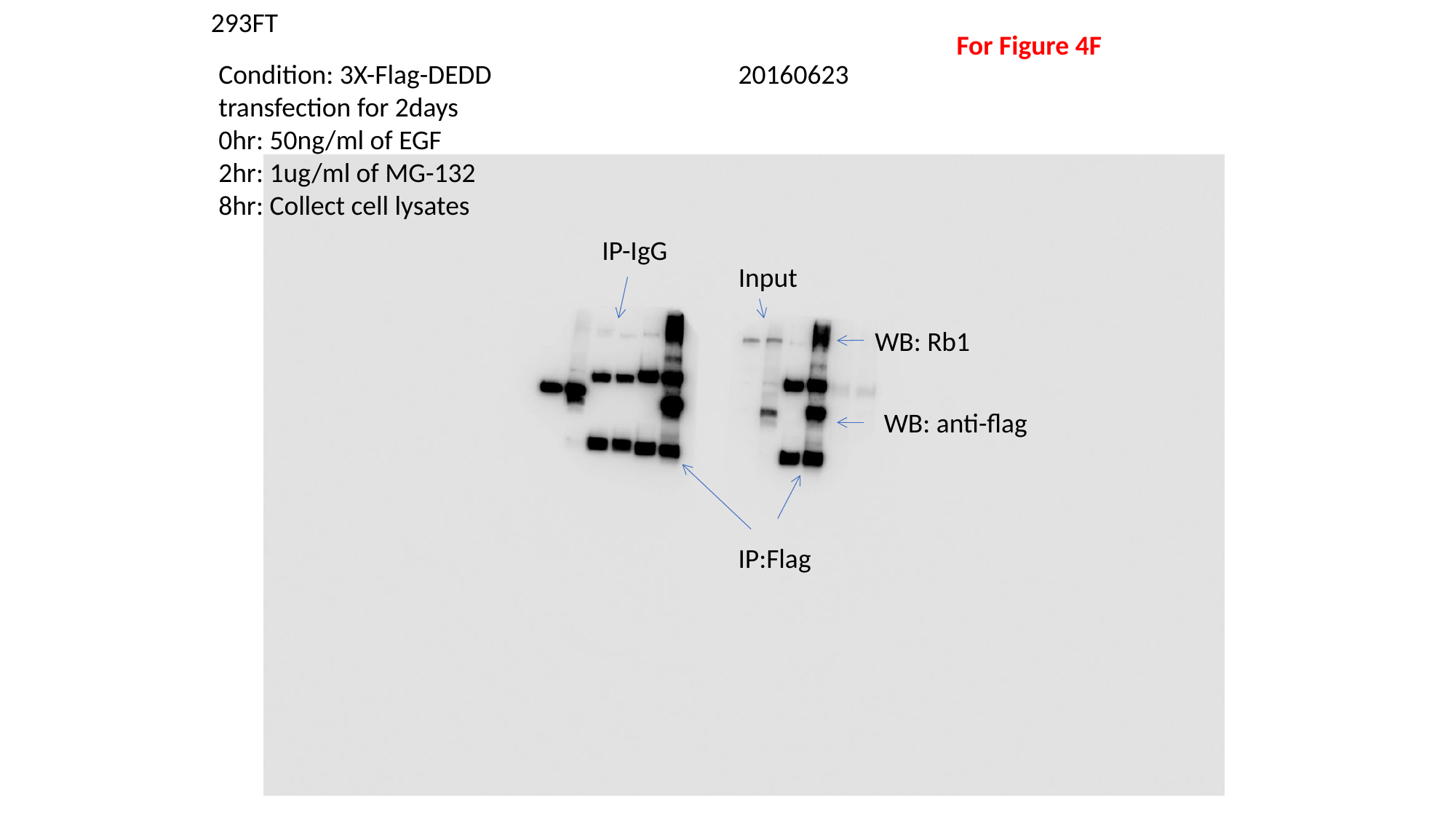

293FT
For Figure 4F
Condition: 3X-Flag-DEDD transfection for 2days
0hr: 50ng/ml of EGF
2hr: 1ug/ml of MG-132
8hr: Collect cell lysates
20160623
IP-IgG
Input
WB: Rb1
WB: anti-flag
IP:Flag

## Slide 50
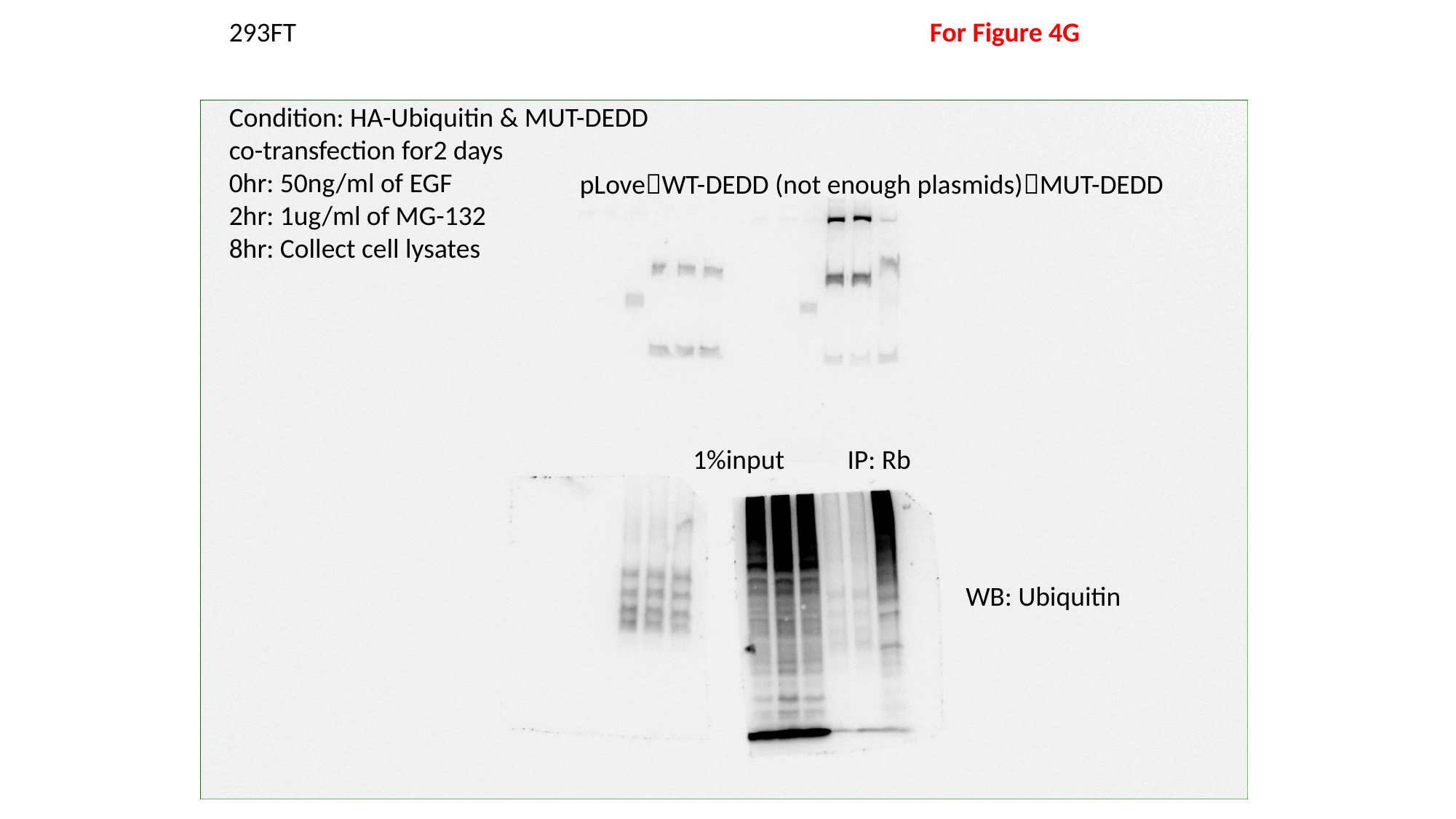

293FT
For Figure 4G
Condition: HA-Ubiquitin & MUT-DEDD co-transfection for2 days
0hr: 50ng/ml of EGF
2hr: 1ug/ml of MG-132
8hr: Collect cell lysates
#
pLoveWT-DEDD (not enough plasmids)MUT-DEDD
1%input
IP: Rb
WB: Ubiquitin

## Slide 51
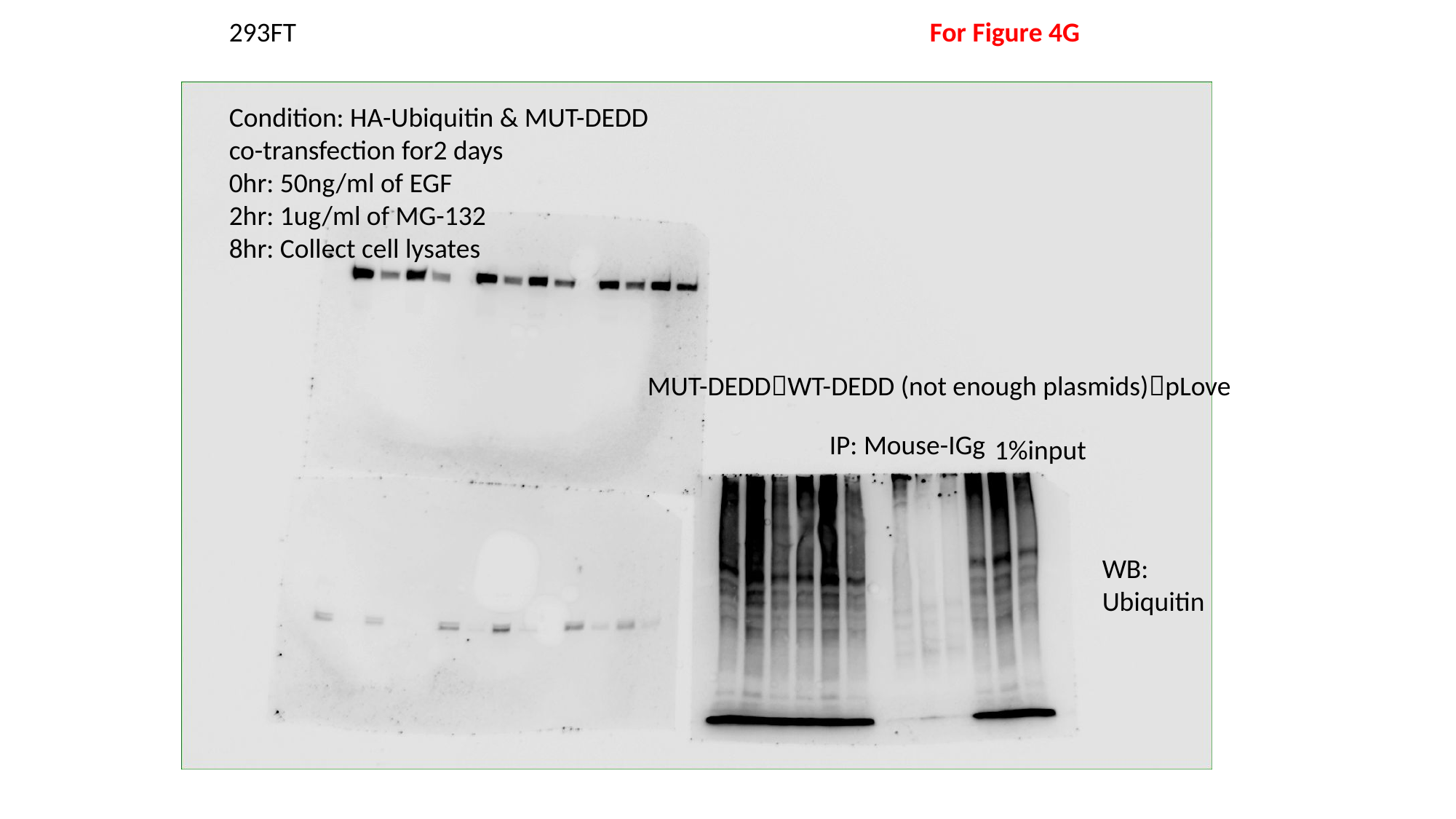

293FT
For Figure 4G
#
Condition: HA-Ubiquitin & MUT-DEDD co-transfection for2 days
0hr: 50ng/ml of EGF
2hr: 1ug/ml of MG-132
8hr: Collect cell lysates
MUT-DEDDWT-DEDD (not enough plasmids)pLove
IP: Mouse-IGg
1%input
WB: Ubiquitin

## Slide 52
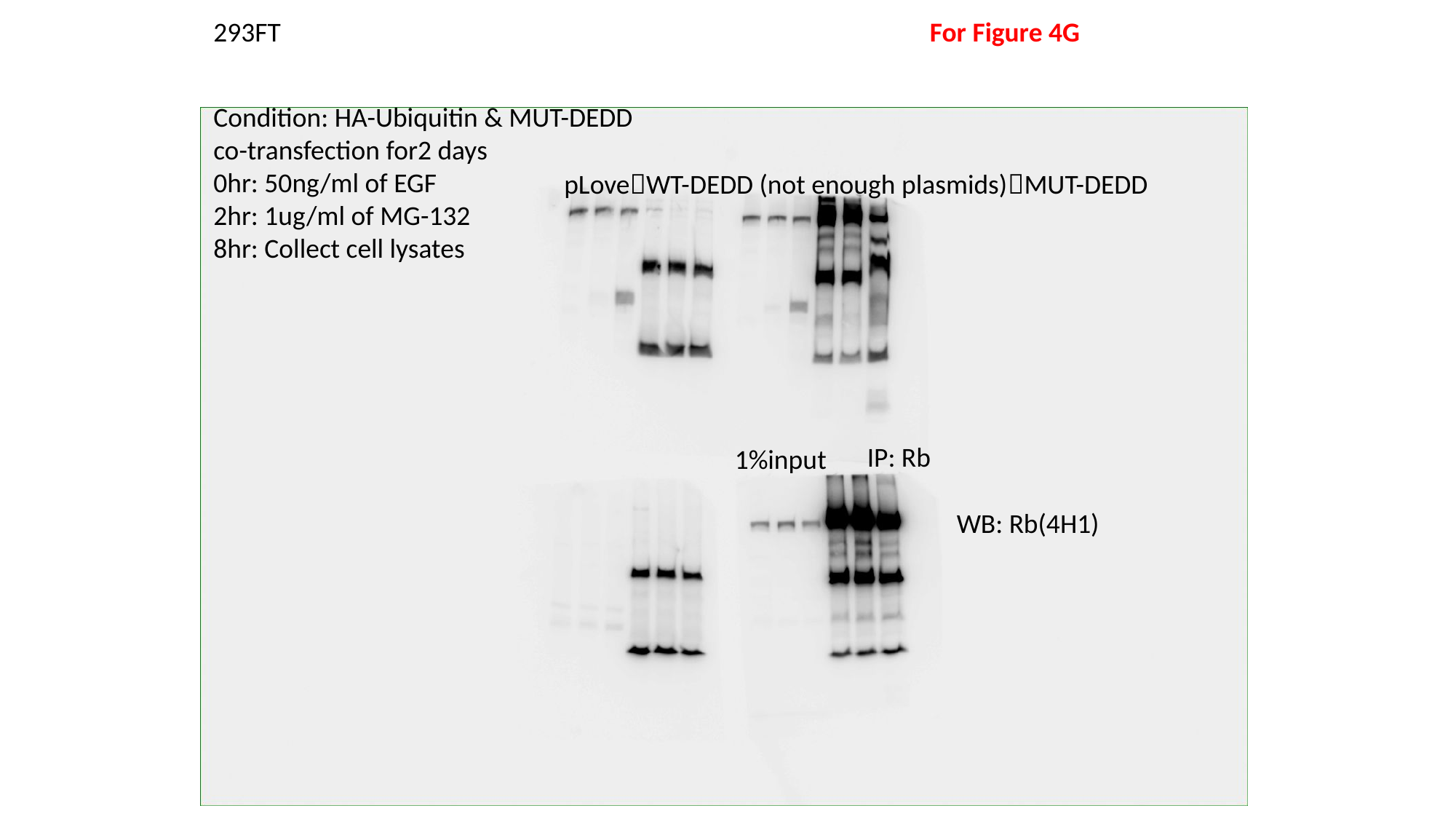

293FT
For Figure 4G
Condition: HA-Ubiquitin & MUT-DEDD co-transfection for2 days
0hr: 50ng/ml of EGF
2hr: 1ug/ml of MG-132
8hr: Collect cell lysates
pLoveWT-DEDD (not enough plasmids)MUT-DEDD
IP: Rb
1%input
WB: Rb(4H1)

## Slide 53
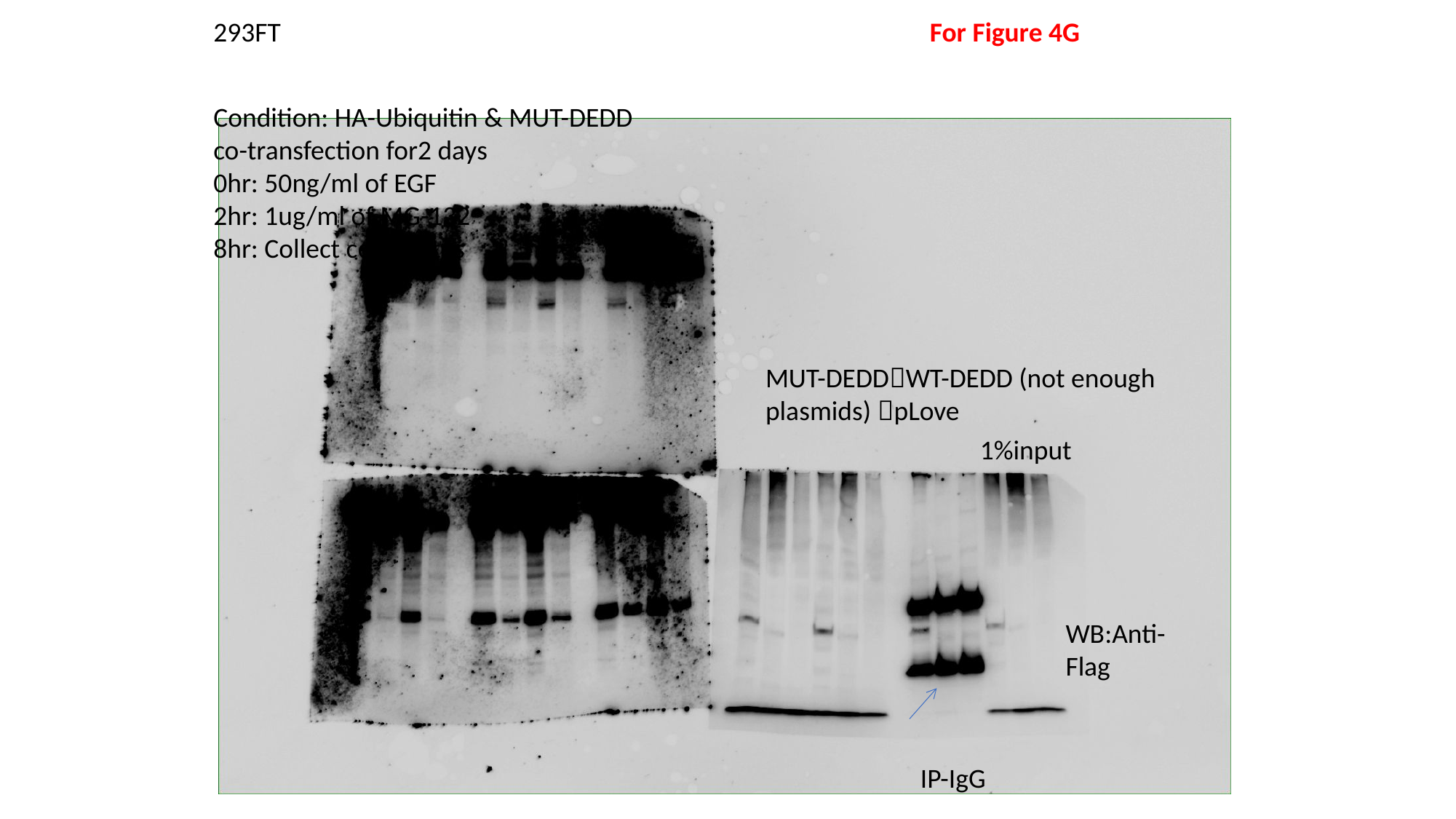

293FT
For Figure 4G
Condition: HA-Ubiquitin & MUT-DEDD co-transfection for2 days
0hr: 50ng/ml of EGF
2hr: 1ug/ml of MG-132
8hr: Collect cell lysates
MUT-DEDDWT-DEDD (not enough plasmids) pLove
1%input
WB:Anti-Flag
IP-IgG

## Slide 54
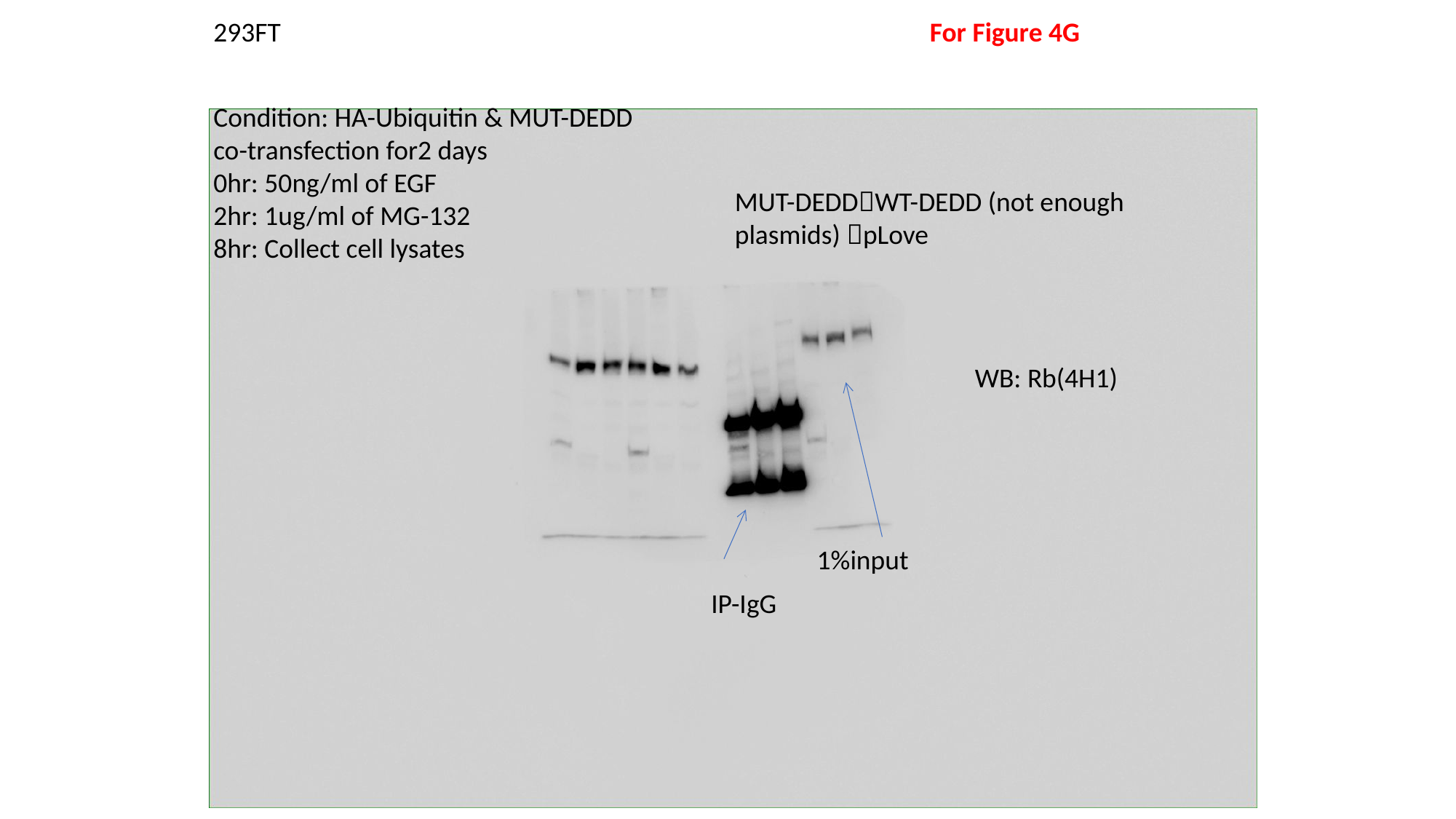

293FT
For Figure 4G
Condition: HA-Ubiquitin & MUT-DEDD co-transfection for2 days
0hr: 50ng/ml of EGF
2hr: 1ug/ml of MG-132
8hr: Collect cell lysates
MUT-DEDDWT-DEDD (not enough plasmids) pLove
WB: Rb(4H1)
1%input
IP-IgG

## Slide 55
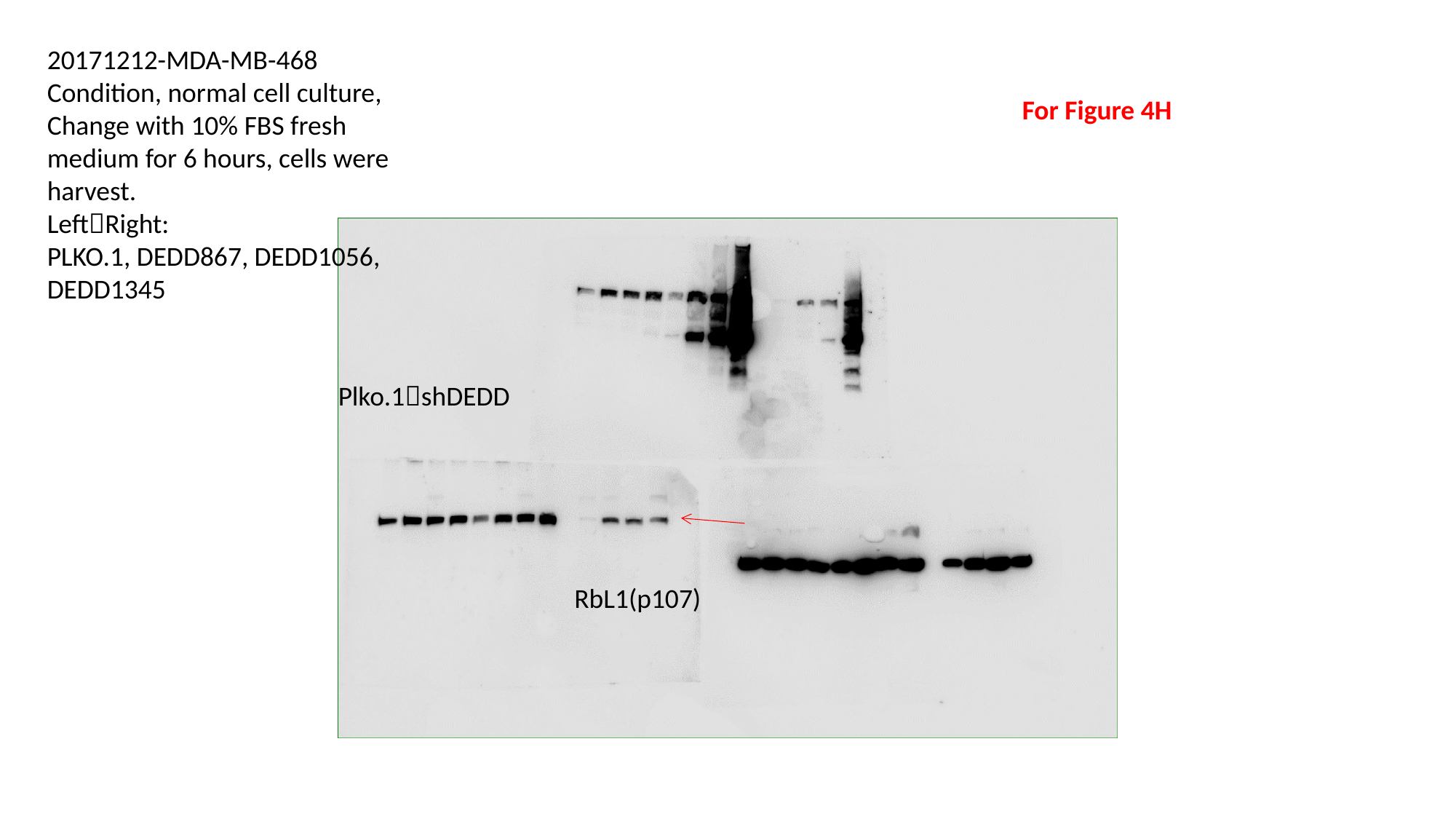

20171212-MDA-MB-468
Condition, normal cell culture, Change with 10% FBS fresh medium for 6 hours, cells were harvest.
LeftRight:
PLKO.1, DEDD867, DEDD1056, DEDD1345
For Figure 4H
Plko.1shDEDD
RbL1(p107)

## Slide 56
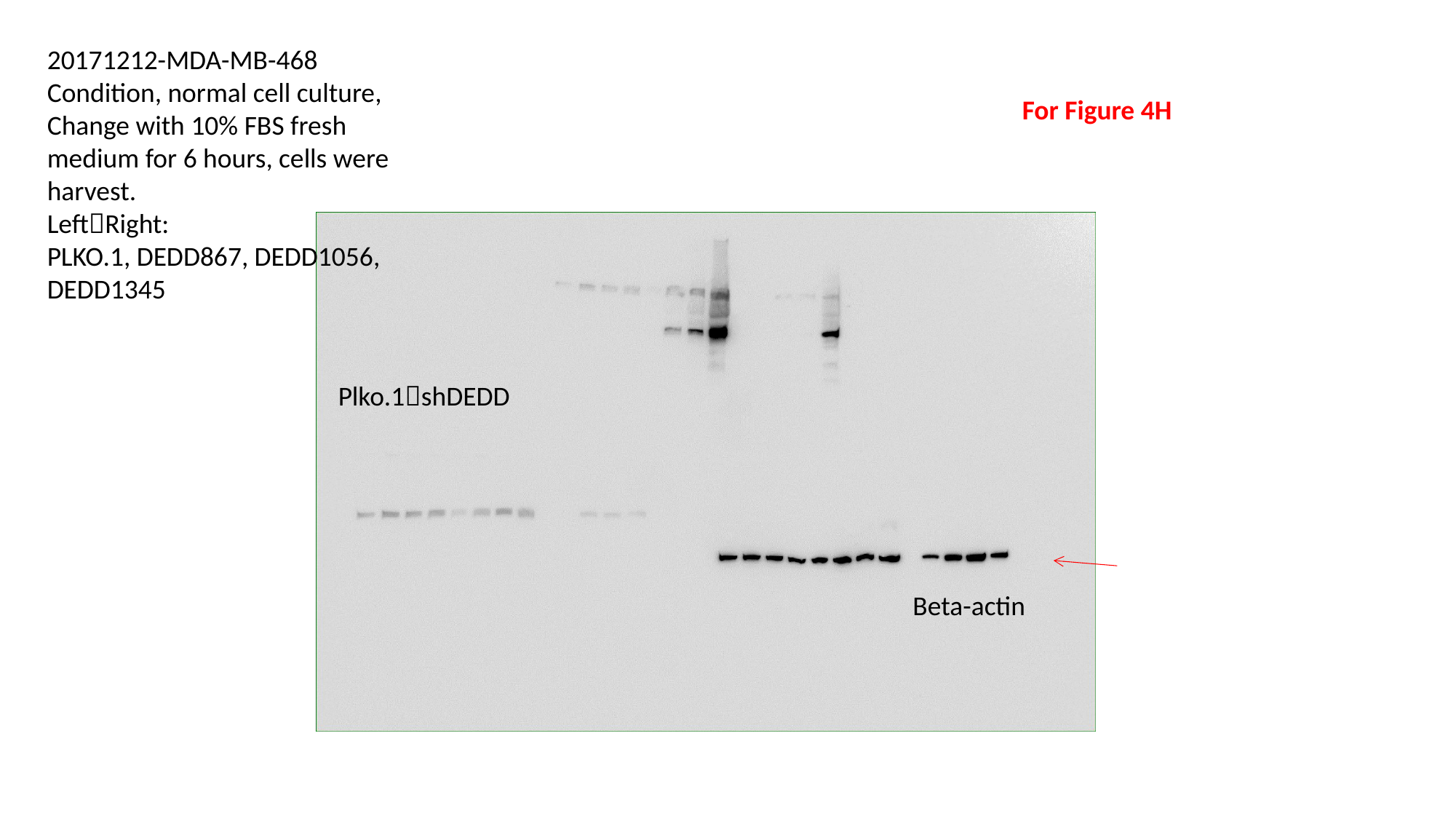

20171212-MDA-MB-468
Condition, normal cell culture, Change with 10% FBS fresh medium for 6 hours, cells were harvest.
LeftRight:
PLKO.1, DEDD867, DEDD1056, DEDD1345
For Figure 4H
Plko.1shDEDD
Beta-actin

## Slide 57
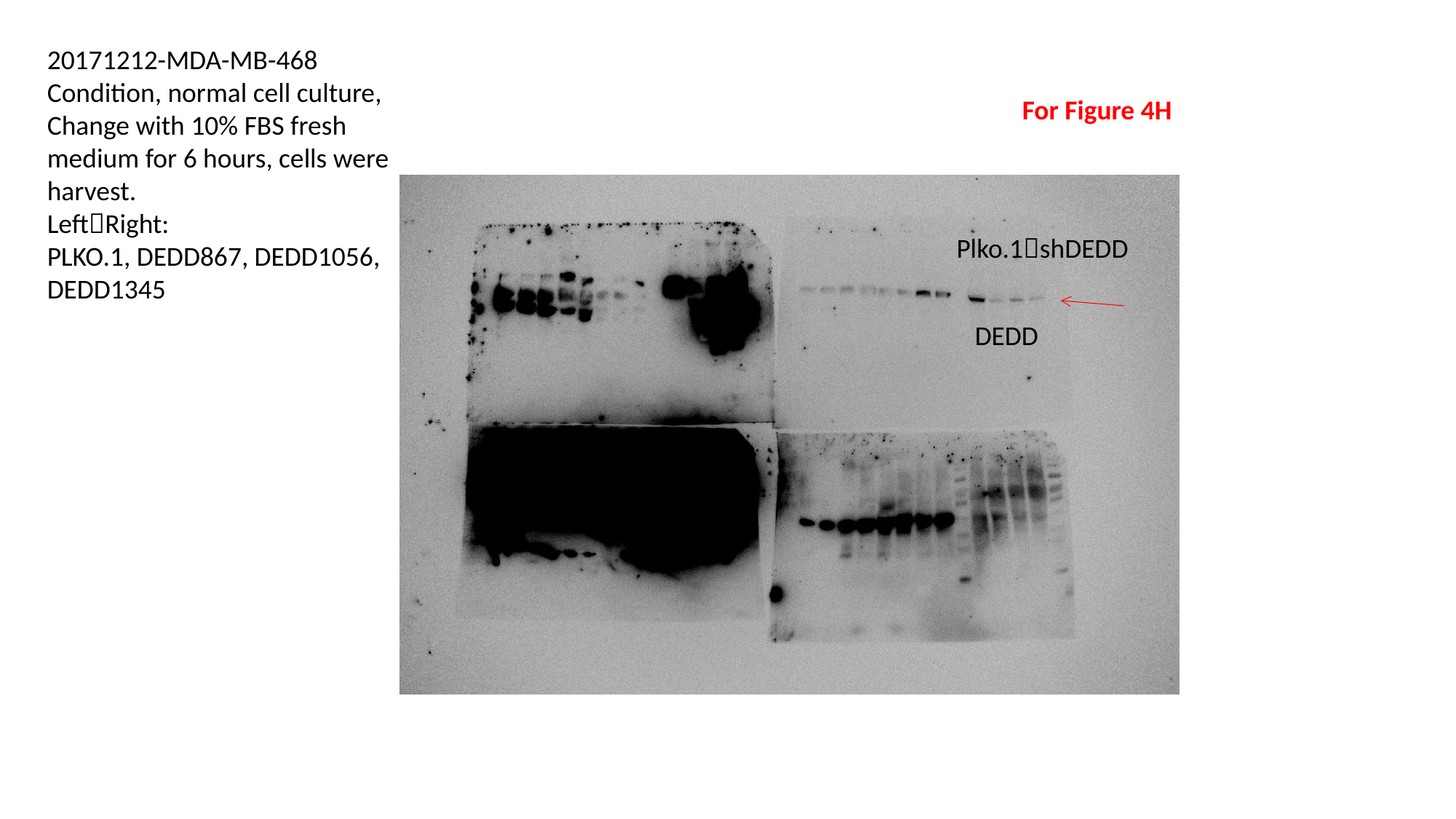

20171212-MDA-MB-468
Condition, normal cell culture, Change with 10% FBS fresh medium for 6 hours, cells were harvest.
LeftRight:
PLKO.1, DEDD867, DEDD1056, DEDD1345
For Figure 4H
Plko.1shDEDD
DEDD

## Slide 58
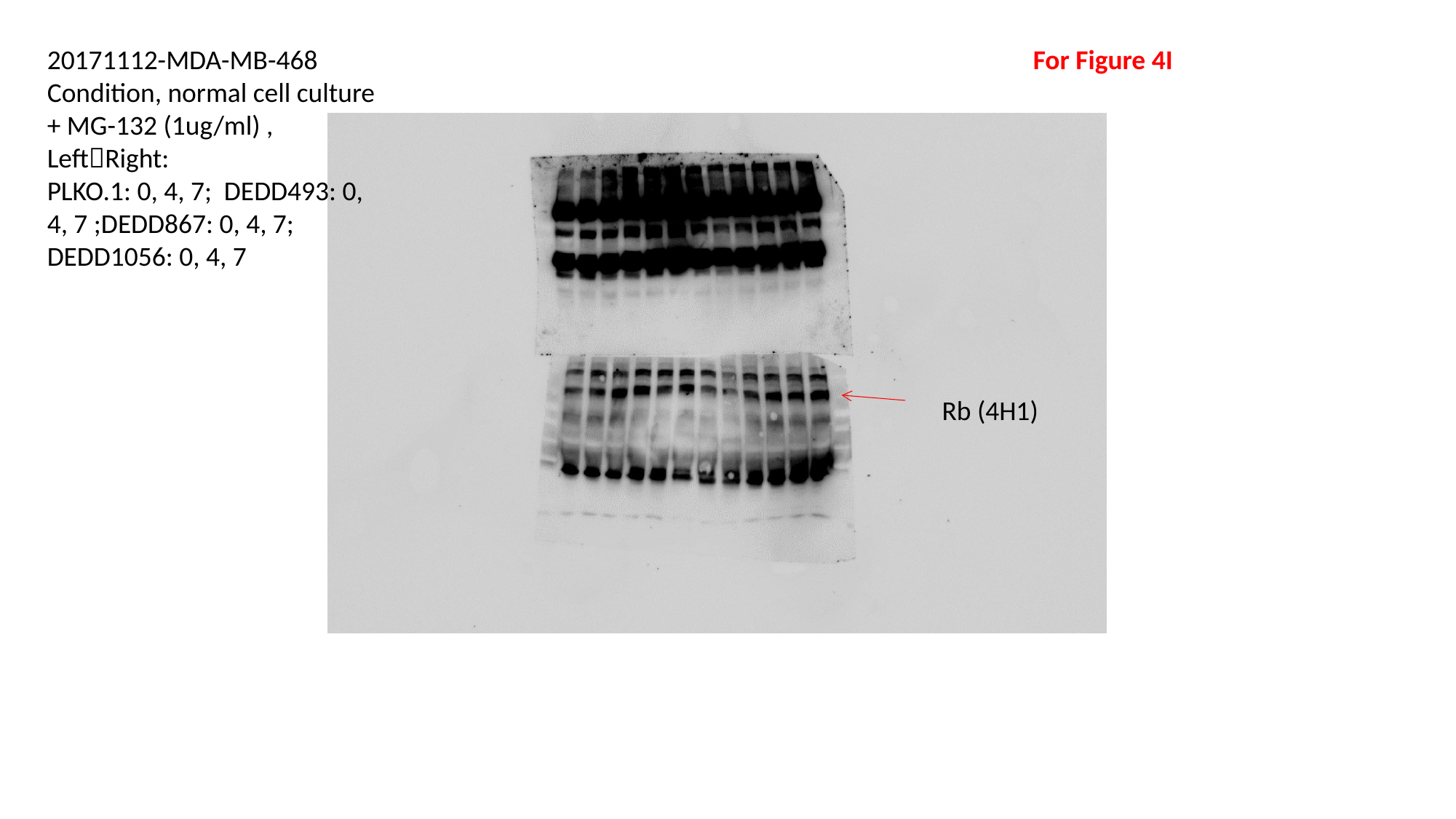

20171112-MDA-MB-468
Condition, normal cell culture + MG-132 (1ug/ml) , LeftRight:
PLKO.1: 0, 4, 7; DEDD493: 0, 4, 7 ;DEDD867: 0, 4, 7; DEDD1056: 0, 4, 7
For Figure 4I
Rb (4H1)

## Slide 59
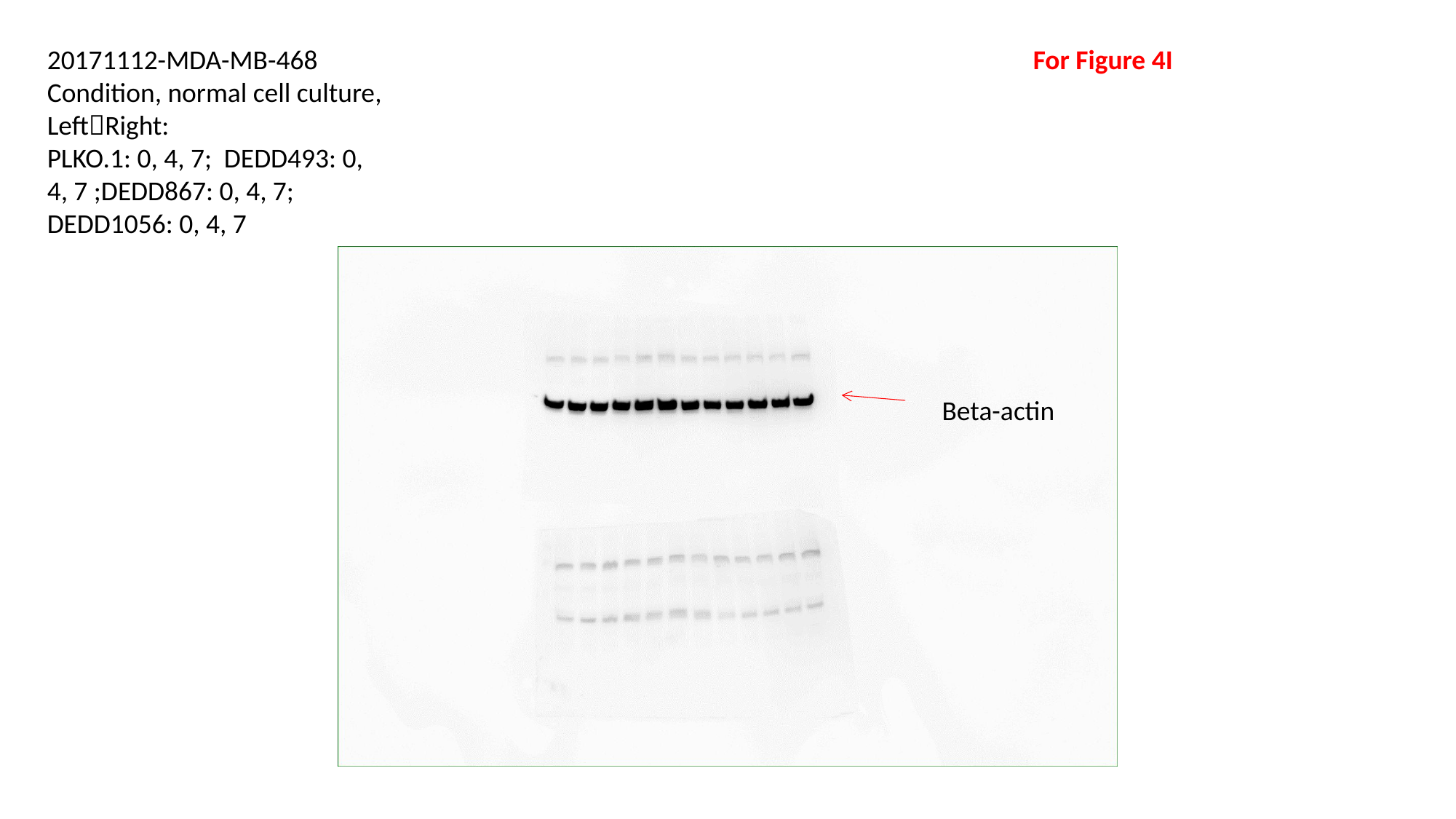

20171112-MDA-MB-468
Condition, normal cell culture, LeftRight:
PLKO.1: 0, 4, 7; DEDD493: 0, 4, 7 ;DEDD867: 0, 4, 7; DEDD1056: 0, 4, 7
For Figure 4I
Beta-actin

## Slide 60
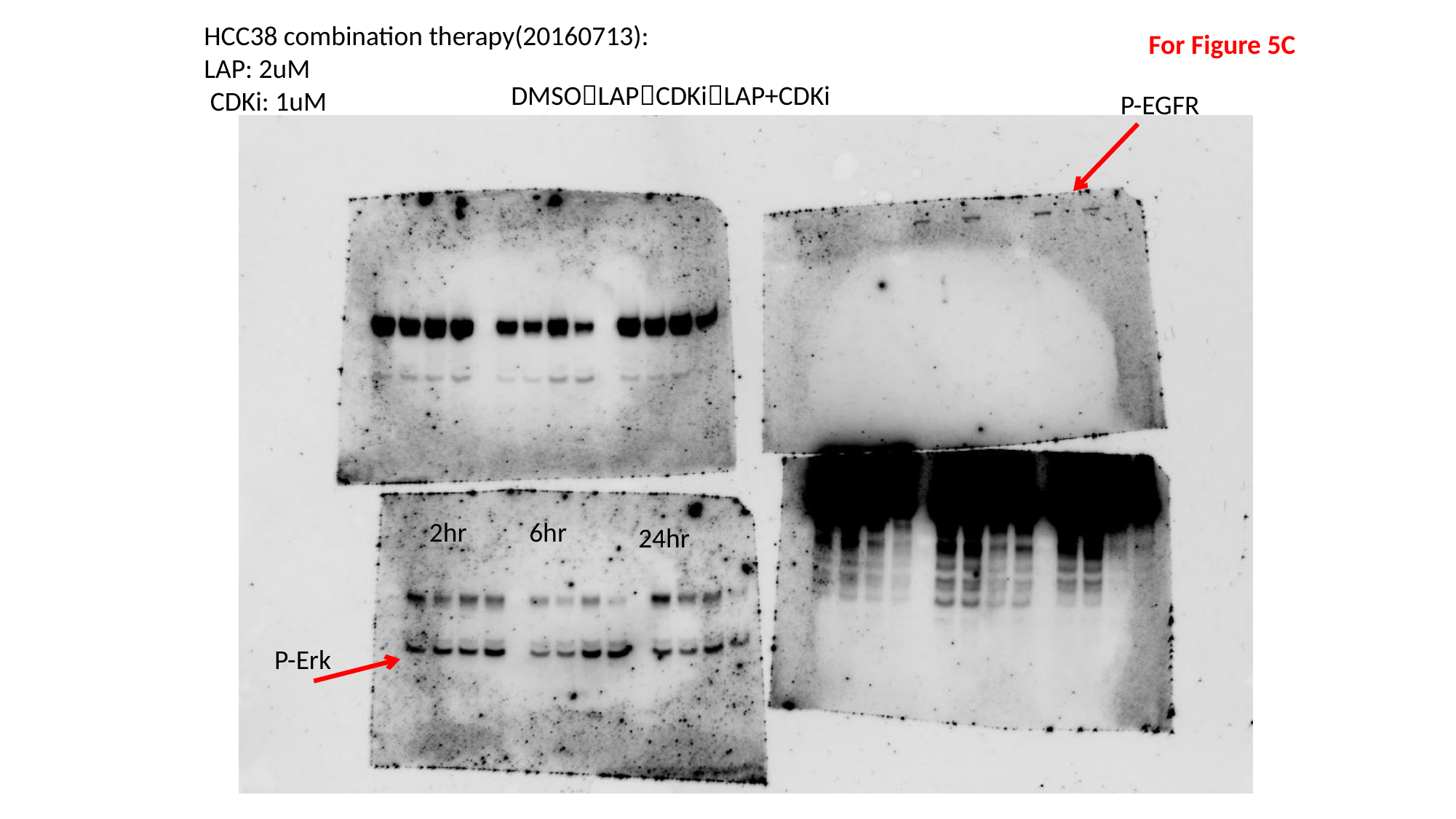

HCC38 combination therapy(20160713):
LAP: 2uM
 CDKi: 1uM
For Figure 5C
DMSOLAPCDKiLAP+CDKi
P-EGFR
6hr
2hr
24hr
P-Erk

## Slide 61
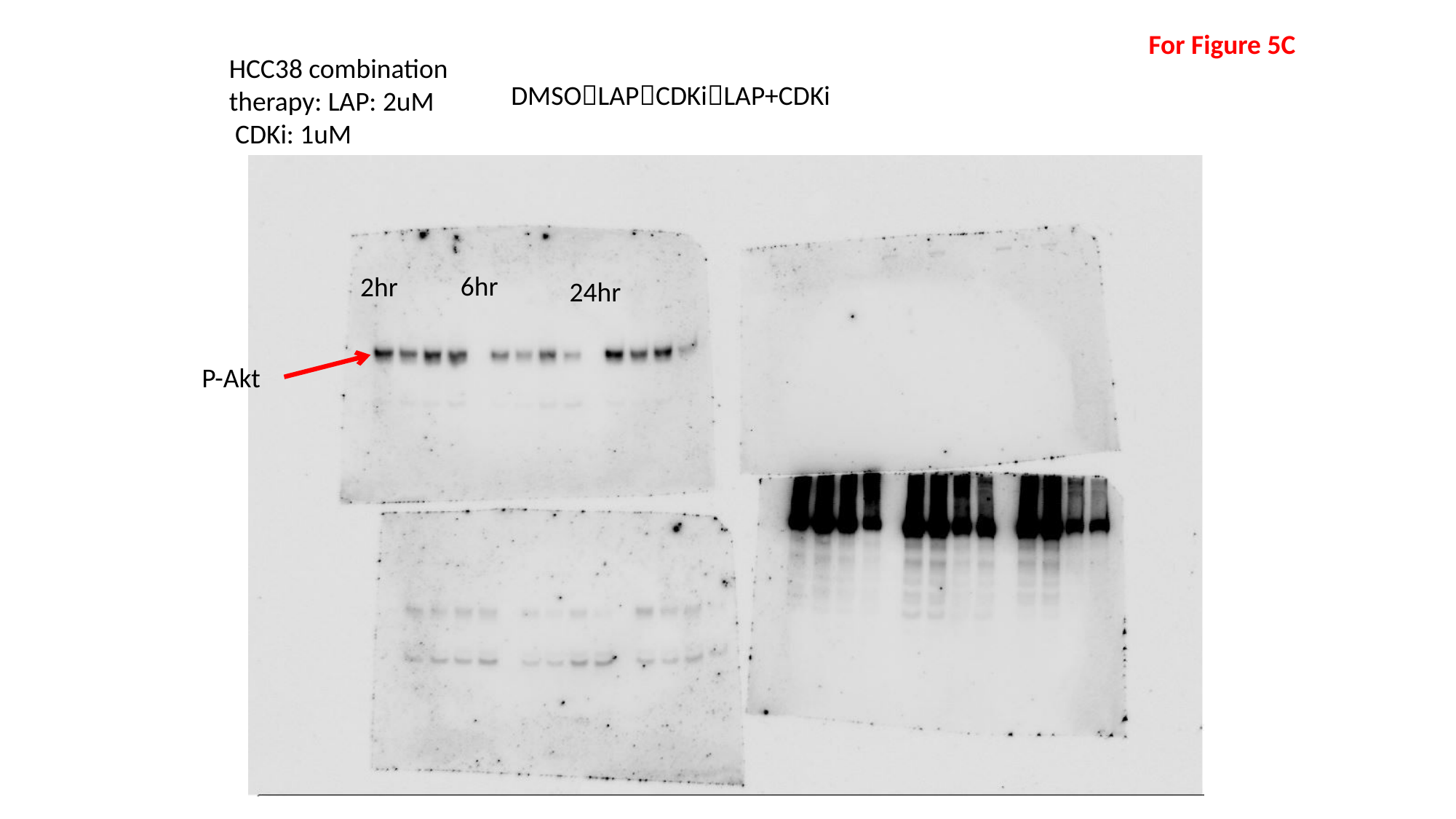

For Figure 5C
HCC38 combination therapy: LAP: 2uM
 CDKi: 1uM
DMSOLAPCDKiLAP+CDKi
6hr
2hr
24hr
P-Akt

## Slide 62
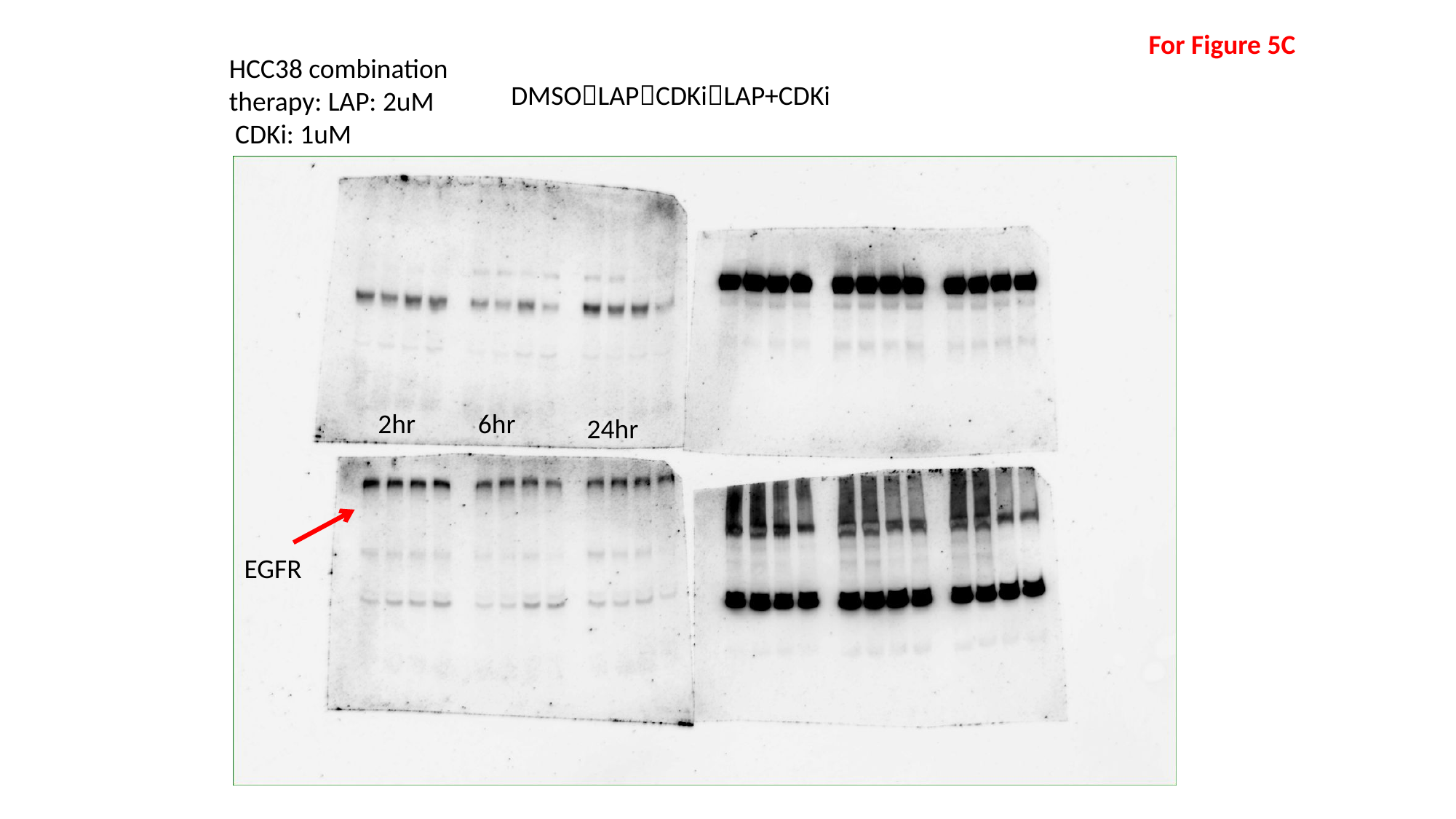

For Figure 5C
HCC38 combination therapy: LAP: 2uM
 CDKi: 1uM
DMSOLAPCDKiLAP+CDKi
6hr
2hr
24hr
EGFR

## Slide 63
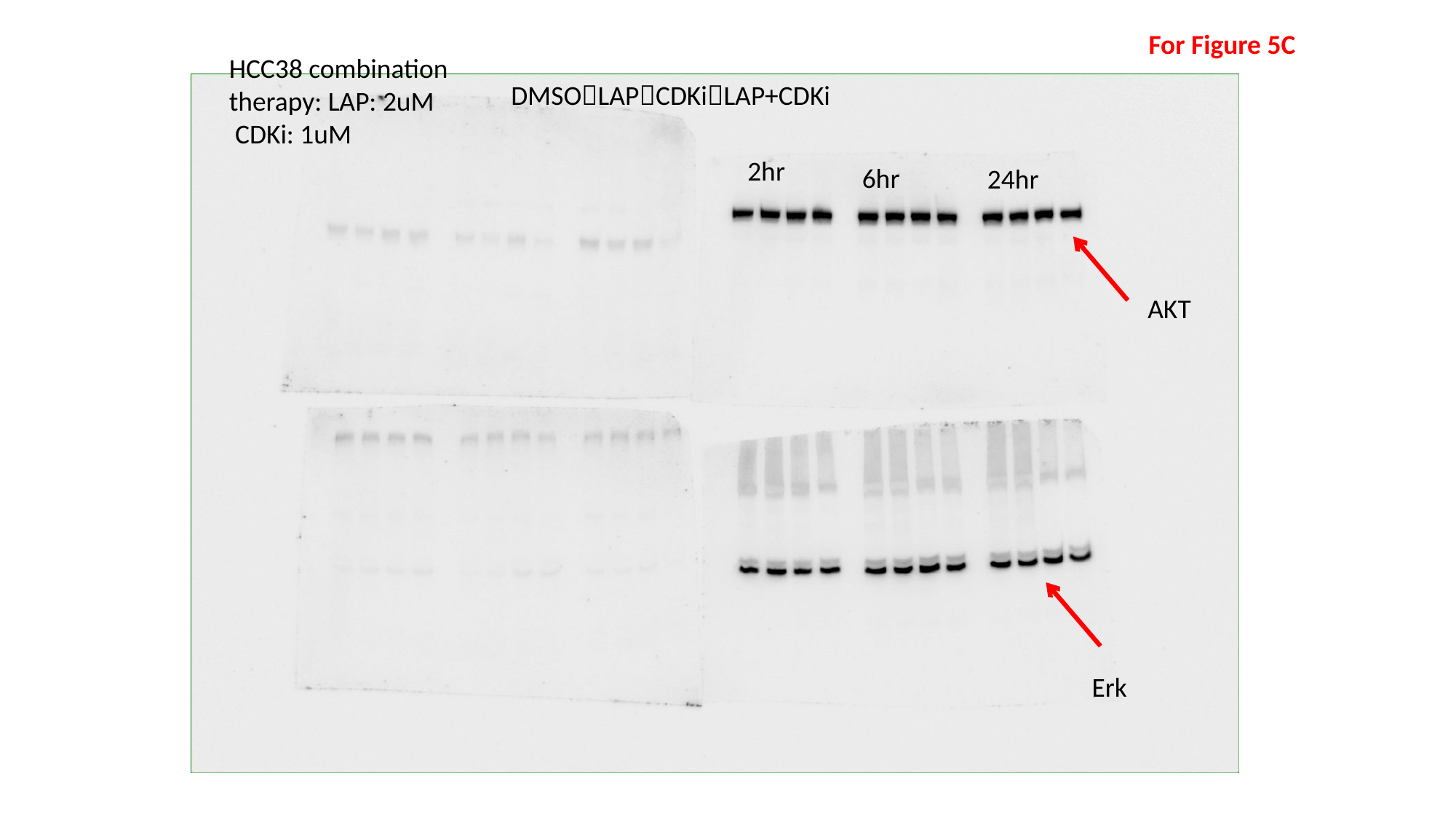

For Figure 5C
HCC38 combination therapy: LAP: 2uM
 CDKi: 1uM
DMSOLAPCDKiLAP+CDKi
2hr
6hr
24hr
AKT
Erk

## Slide 64
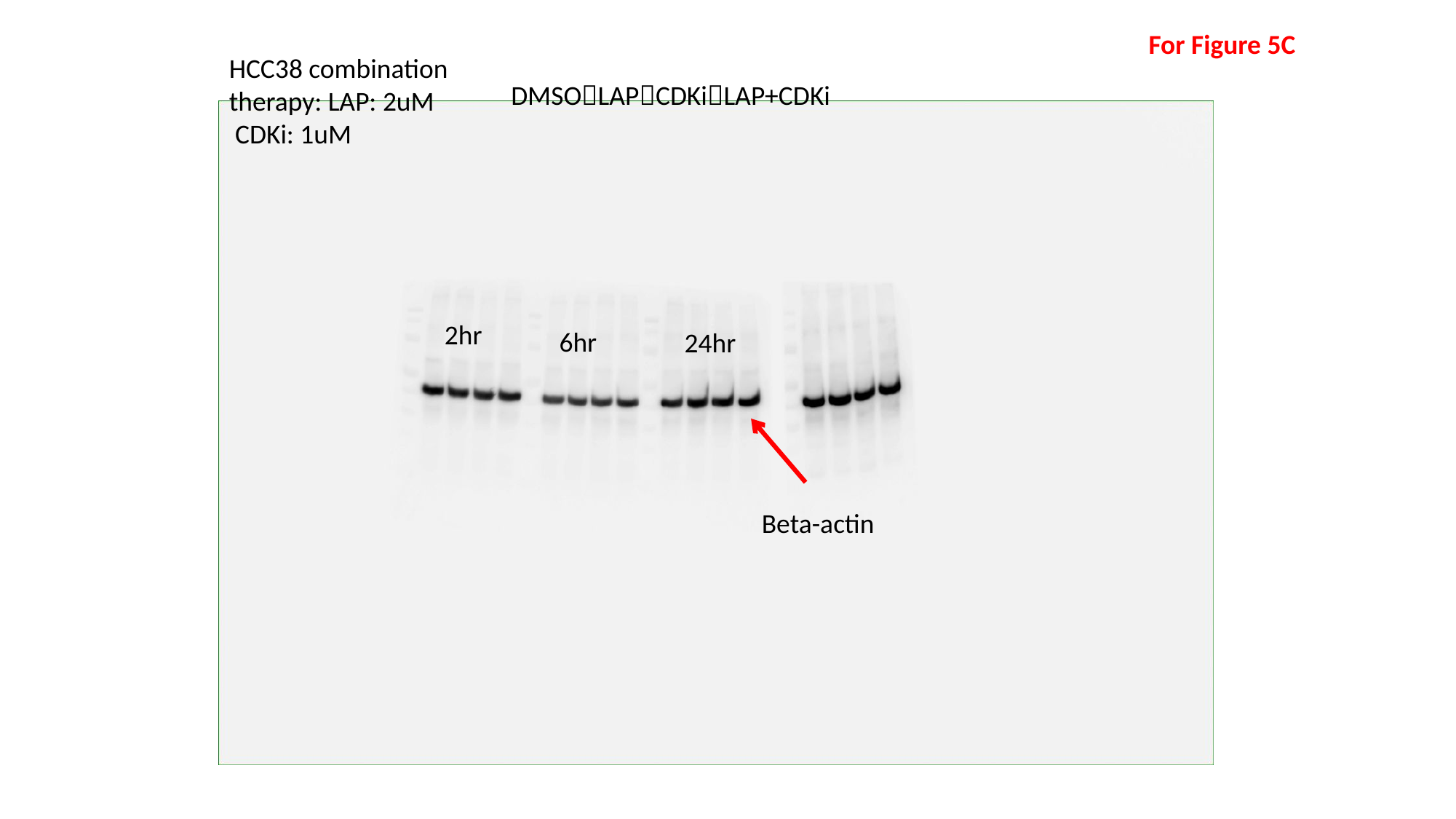

For Figure 5C
HCC38 combination therapy: LAP: 2uM
 CDKi: 1uM
DMSOLAPCDKiLAP+CDKi
2hr
6hr
24hr
Beta-actin

## Slide 65
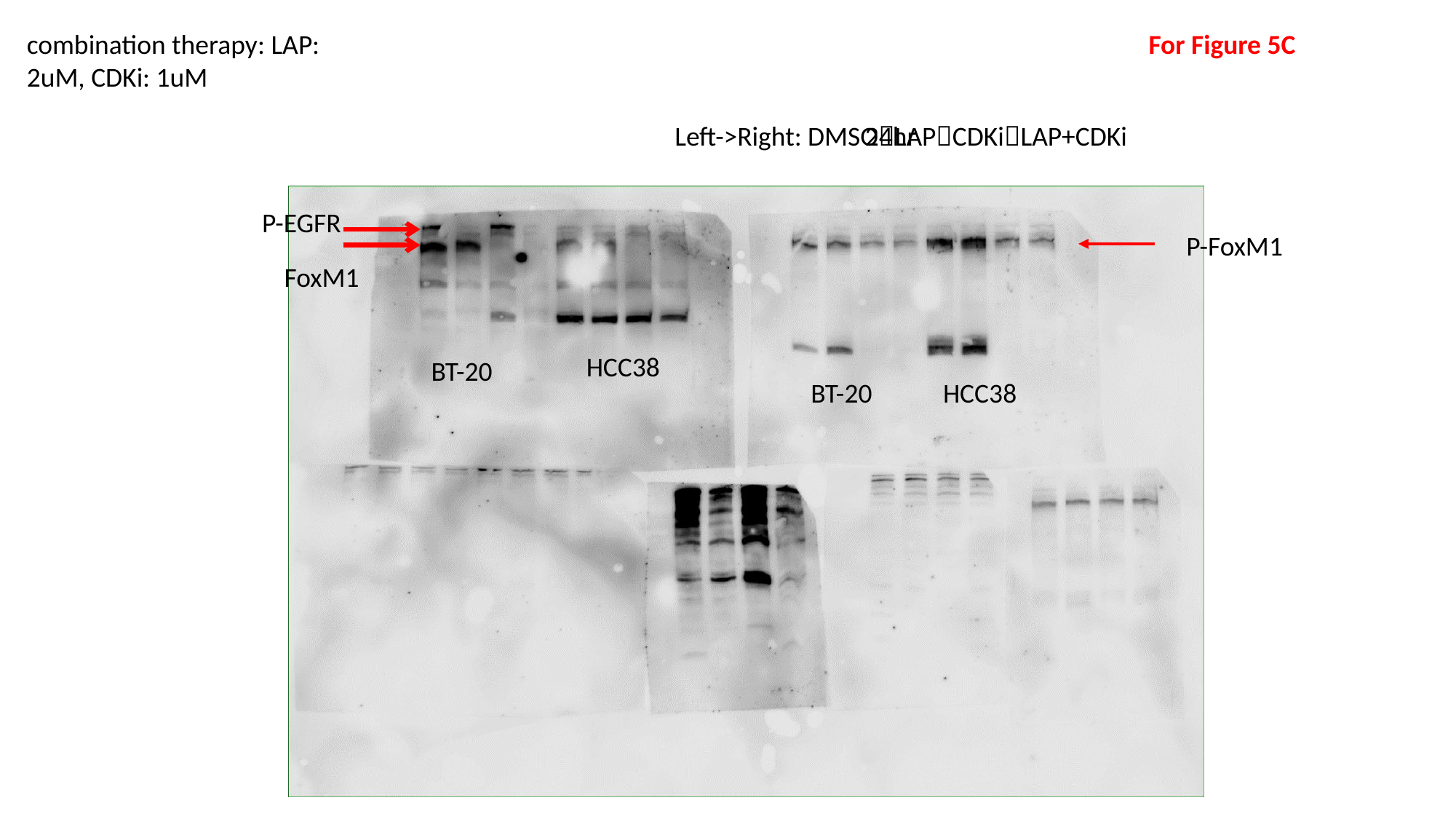

combination therapy: LAP: 2uM, CDKi: 1uM
For Figure 5C
24hr
Left->Right: DMSOLAPCDKiLAP+CDKi
P-EGFR
P-FoxM1
FoxM1
HCC38
BT-20
BT-20
HCC38

## Slide 66
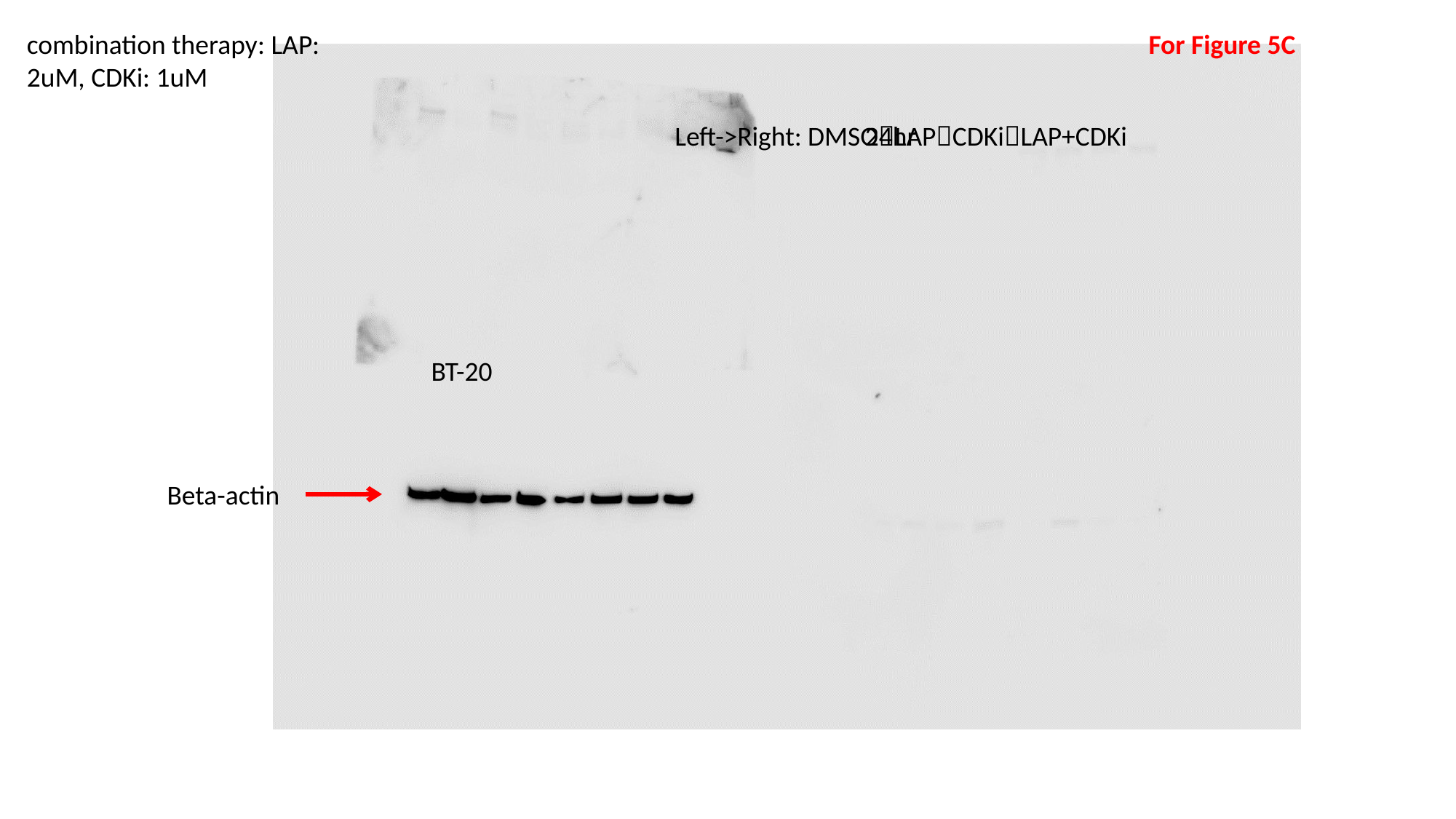

combination therapy: LAP: 2uM, CDKi: 1uM
For Figure 5C
24hr
Left->Right: DMSOLAPCDKiLAP+CDKi
BT-20
Beta-actin

## Slide 67
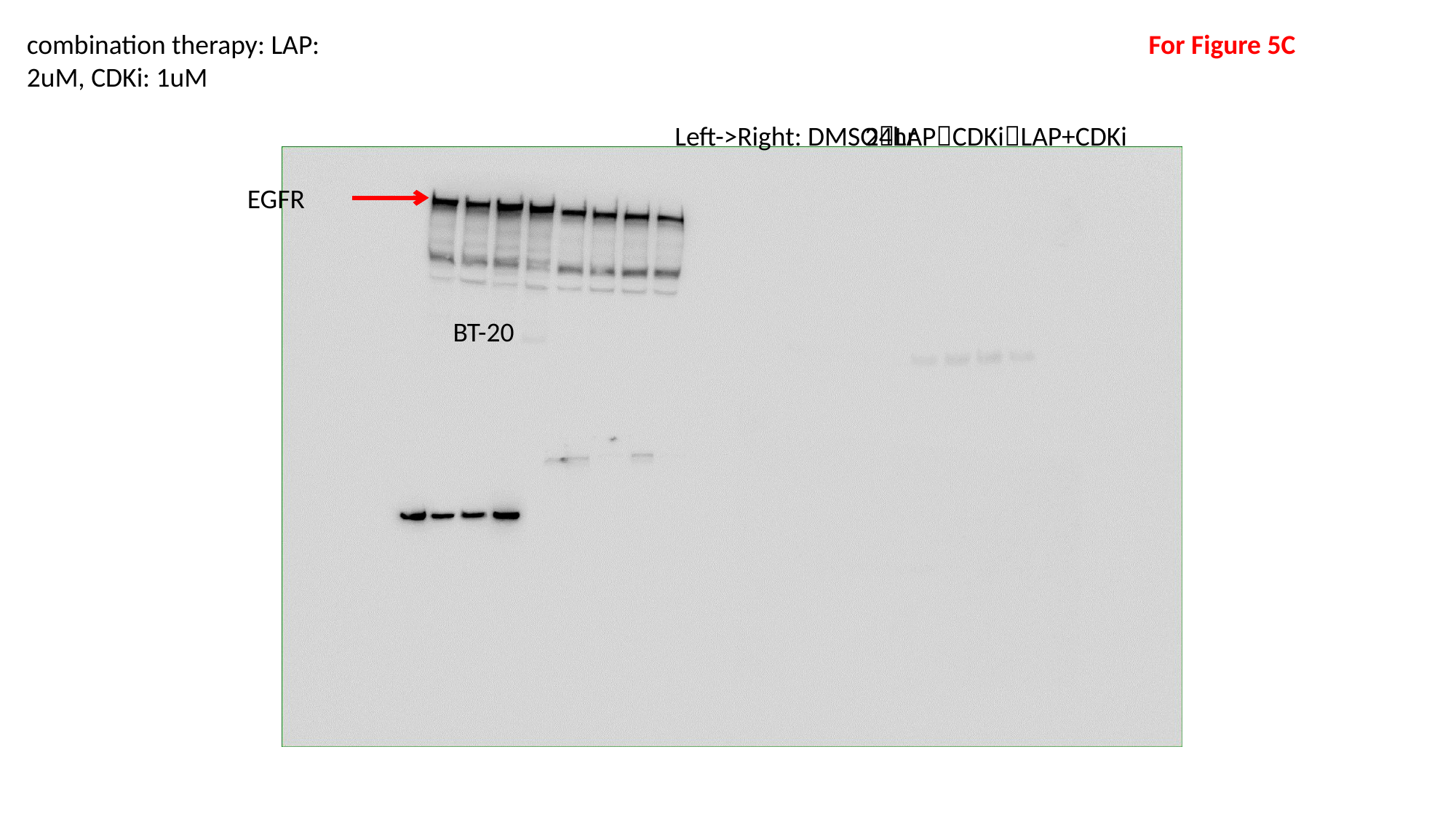

combination therapy: LAP: 2uM, CDKi: 1uM
For Figure 5C
24hr
Left->Right: DMSOLAPCDKiLAP+CDKi
EGFR
BT-20

## Slide 68
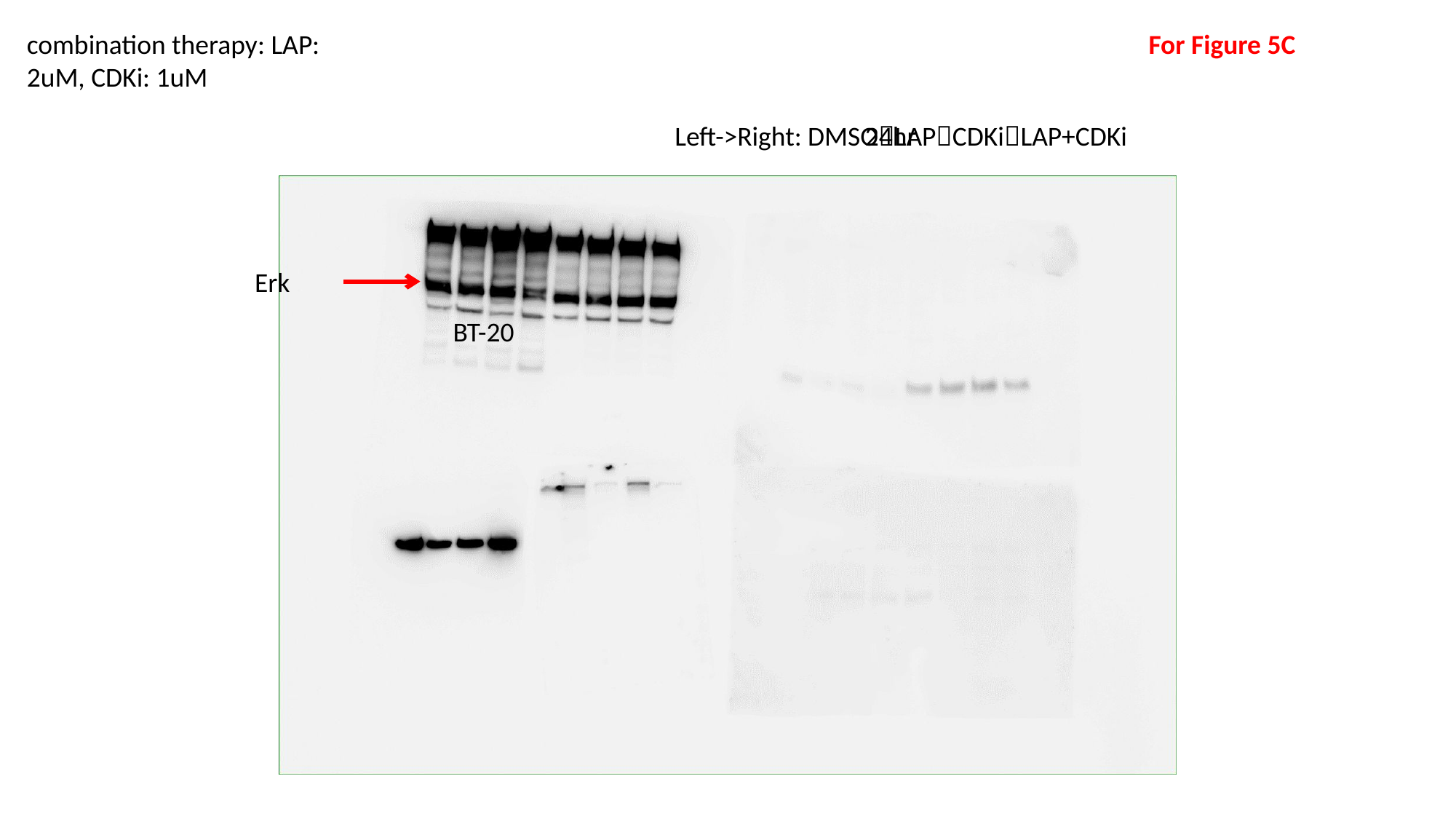

combination therapy: LAP: 2uM, CDKi: 1uM
For Figure 5C
24hr
Left->Right: DMSOLAPCDKiLAP+CDKi
Erk
BT-20

## Slide 69
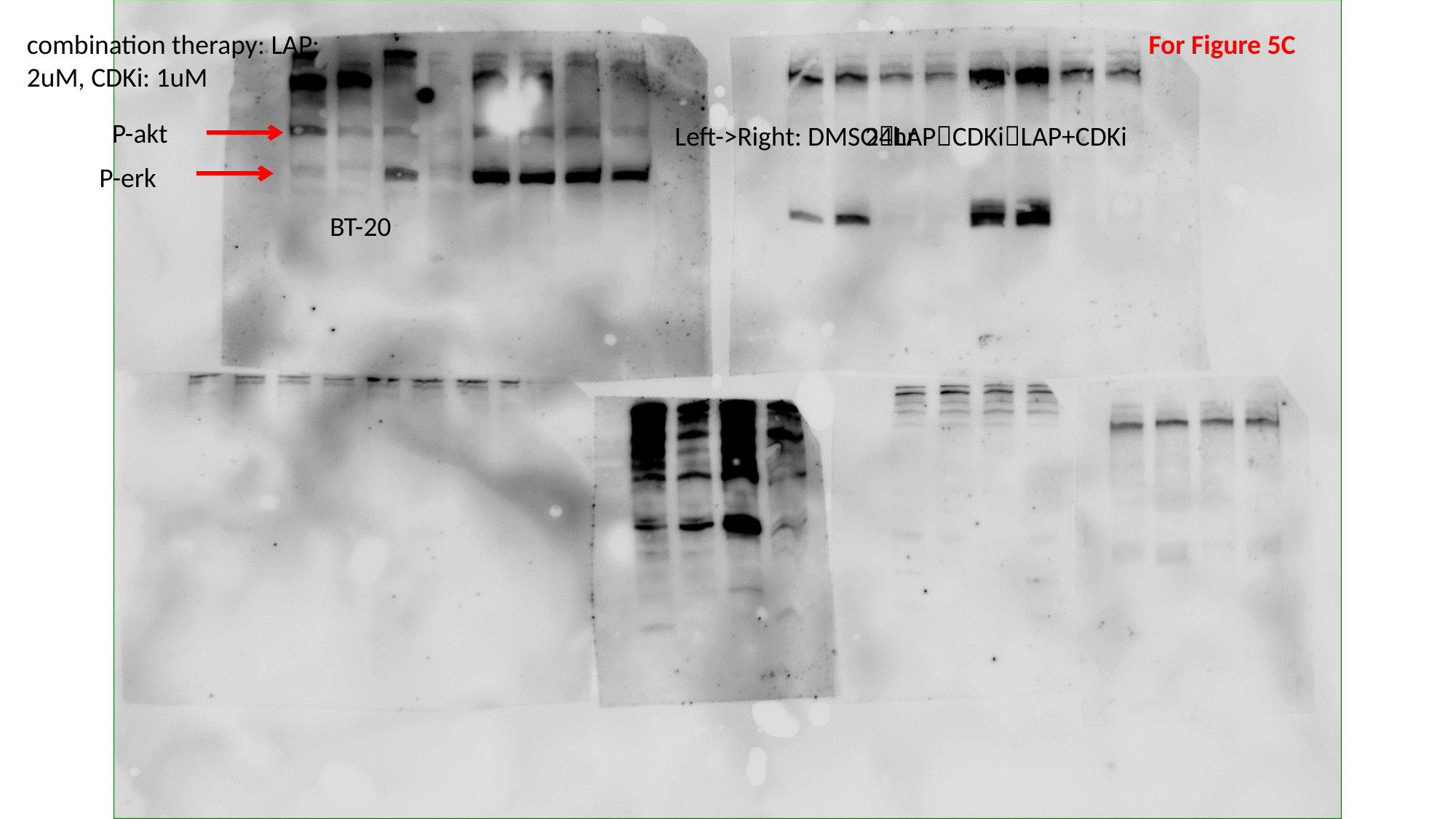

combination therapy: LAP: 2uM, CDKi: 1uM
For Figure 5C
P-akt
24hr
Left->Right: DMSOLAPCDKiLAP+CDKi
P-erk
BT-20

## Slide 70
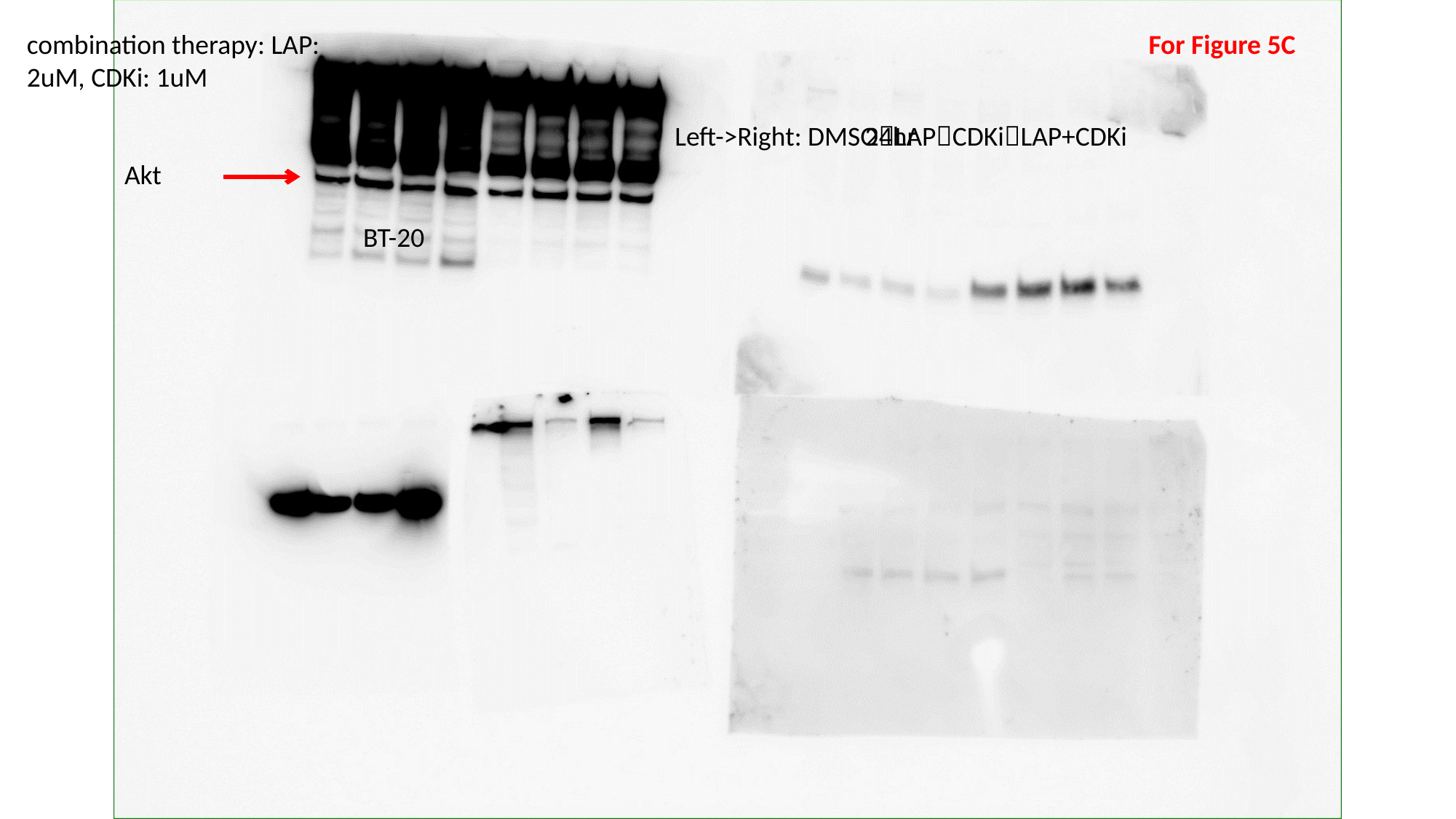

combination therapy: LAP: 2uM, CDKi: 1uM
For Figure 5C
24hr
Left->Right: DMSOLAPCDKiLAP+CDKi
Akt
BT-20

## Slide 71
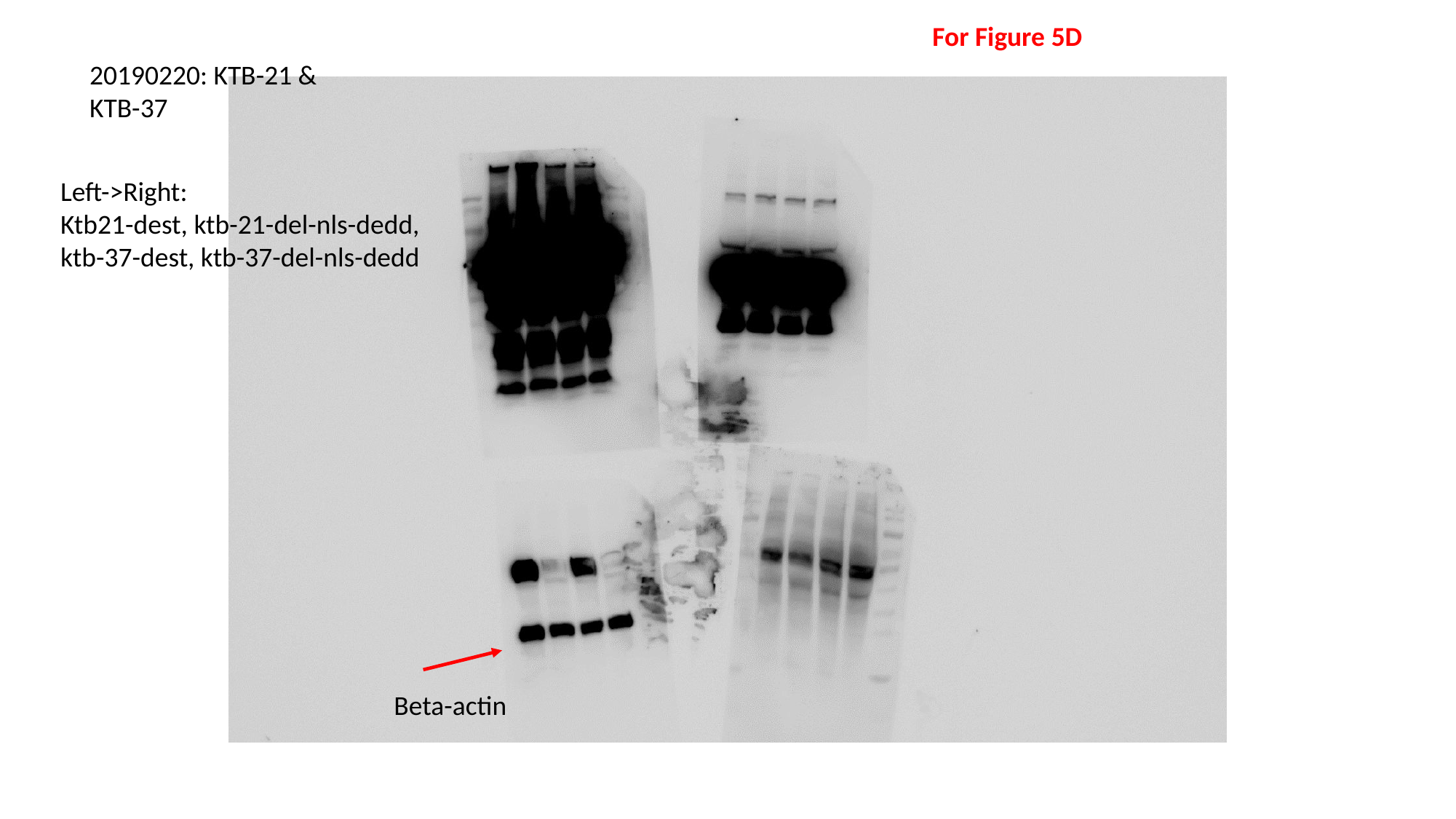

For Figure 5D
20190220: KTB-21 & KTB-37
Left->Right:
Ktb21-dest, ktb-21-del-nls-dedd, ktb-37-dest, ktb-37-del-nls-dedd
Beta-actin

## Slide 72
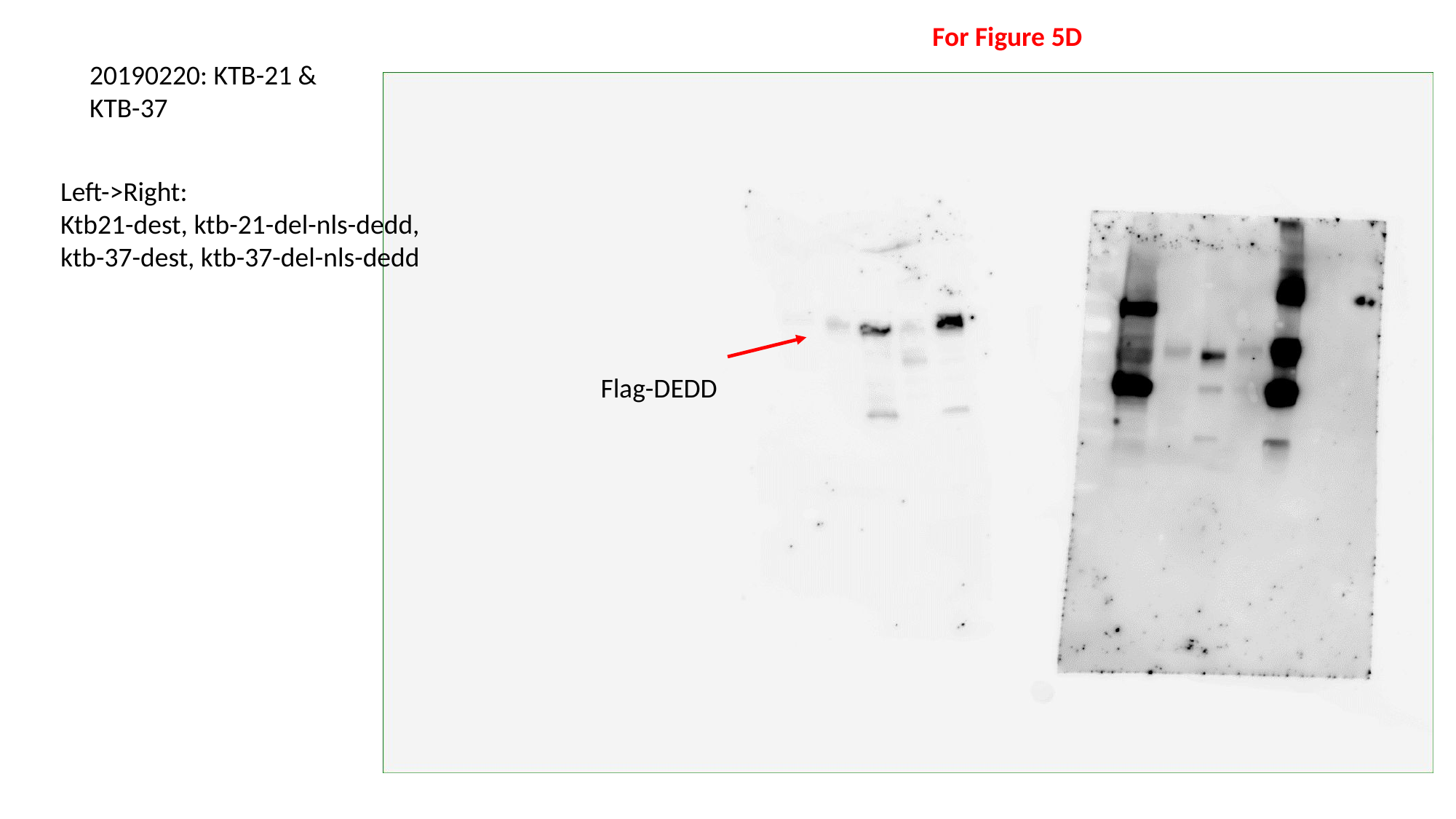

For Figure 5D
20190220: KTB-21 & KTB-37
Left->Right:
Ktb21-dest, ktb-21-del-nls-dedd, ktb-37-dest, ktb-37-del-nls-dedd
Flag-DEDD

## Slide 73
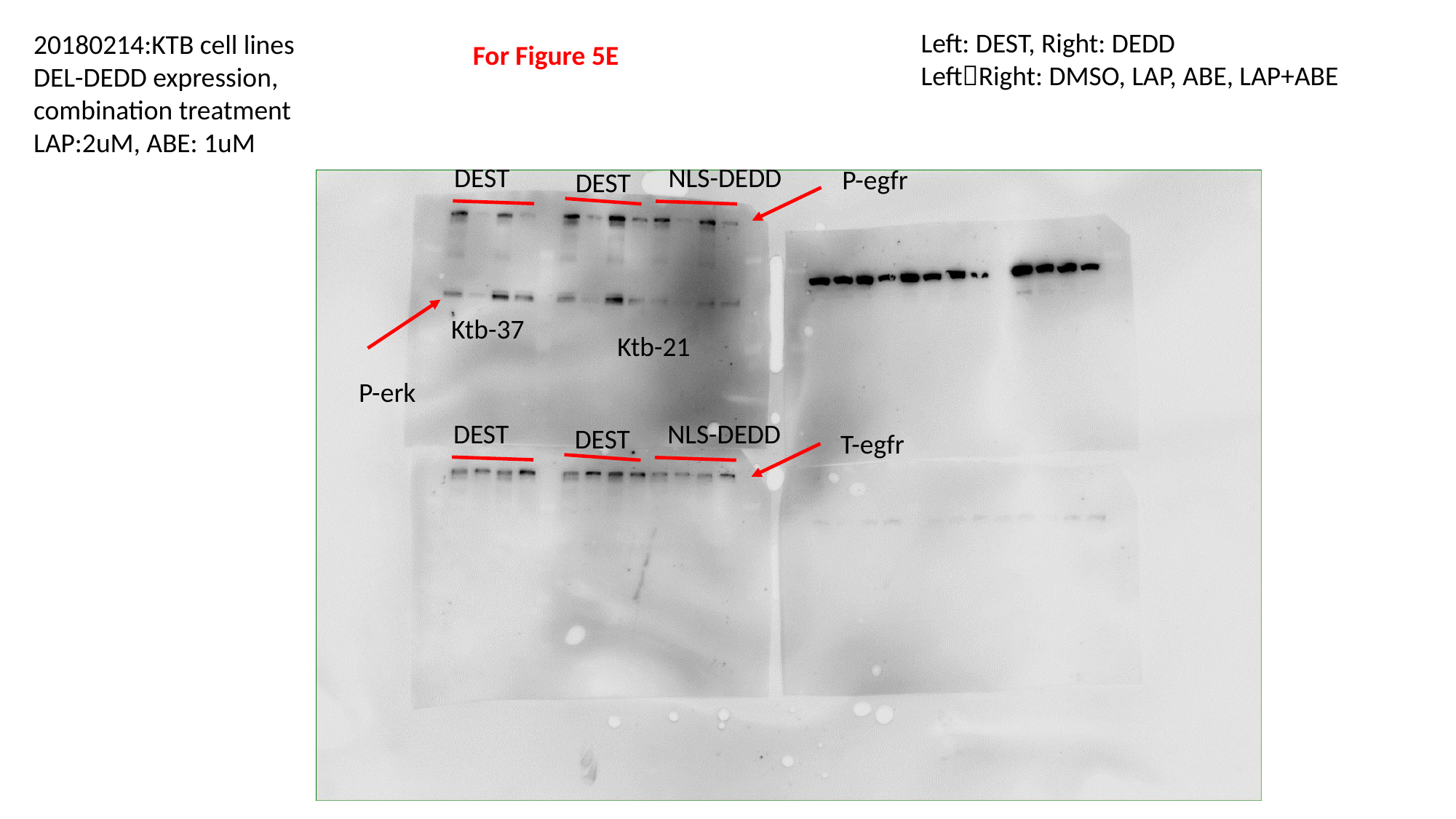

Left: DEST, Right: DEDD
LeftRight: DMSO, LAP, ABE, LAP+ABE
20180214:KTB cell lines DEL-DEDD expression, combination treatment
LAP:2uM, ABE: 1uM
For Figure 5E
DEST
NLS-DEDD
P-egfr
DEST
Ktb-37
Ktb-21
P-erk
DEST
NLS-DEDD
DEST
T-egfr

## Slide 74
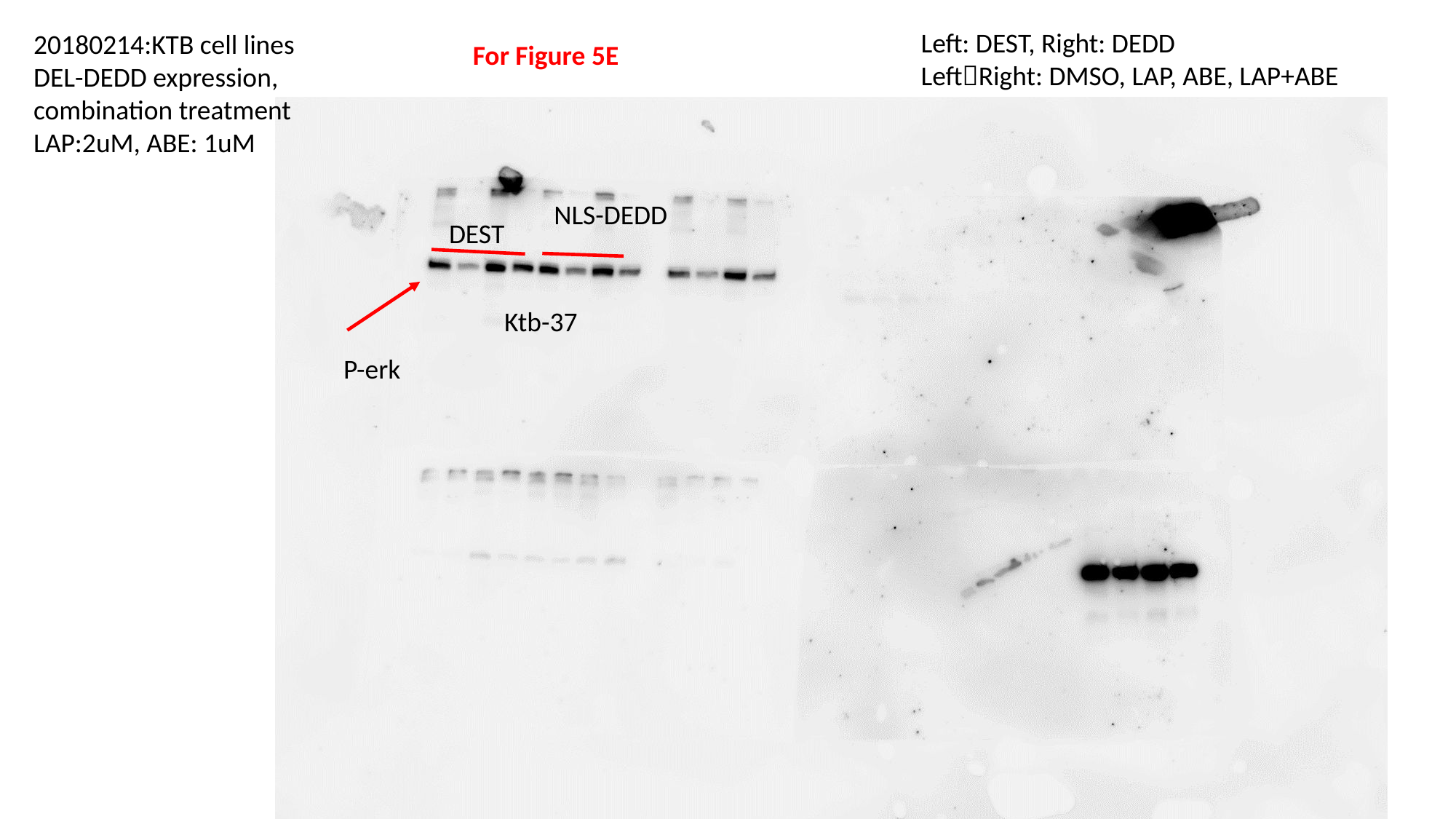

Left: DEST, Right: DEDD
LeftRight: DMSO, LAP, ABE, LAP+ABE
20180214:KTB cell lines DEL-DEDD expression, combination treatment
LAP:2uM, ABE: 1uM
For Figure 5E
NLS-DEDD
DEST
Ktb-37
P-erk

## Slide 75
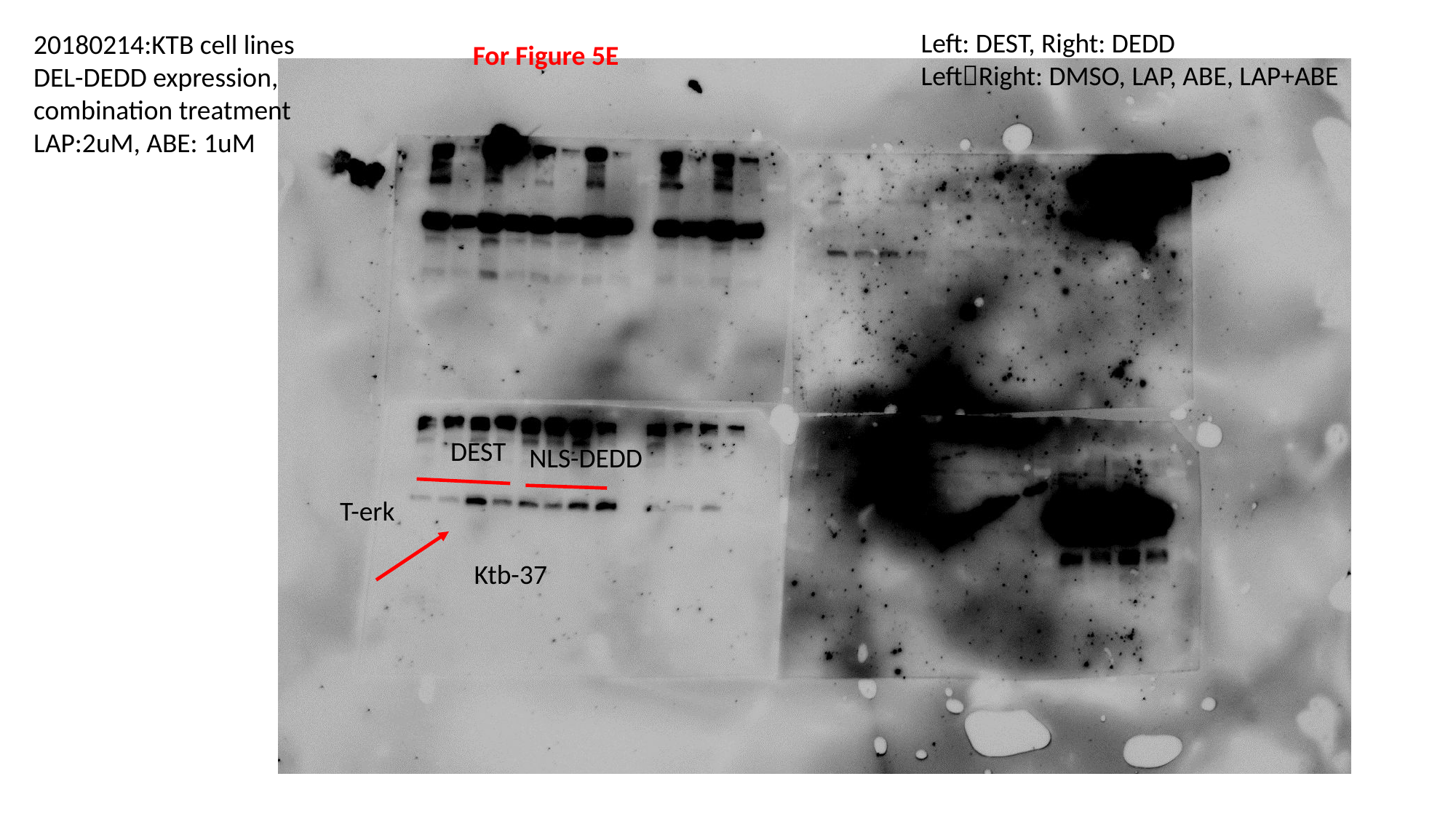

Left: DEST, Right: DEDD
LeftRight: DMSO, LAP, ABE, LAP+ABE
20180214:KTB cell lines DEL-DEDD expression, combination treatment
LAP:2uM, ABE: 1uM
For Figure 5E
DEST
NLS-DEDD
T-erk
Ktb-37

## Slide 76
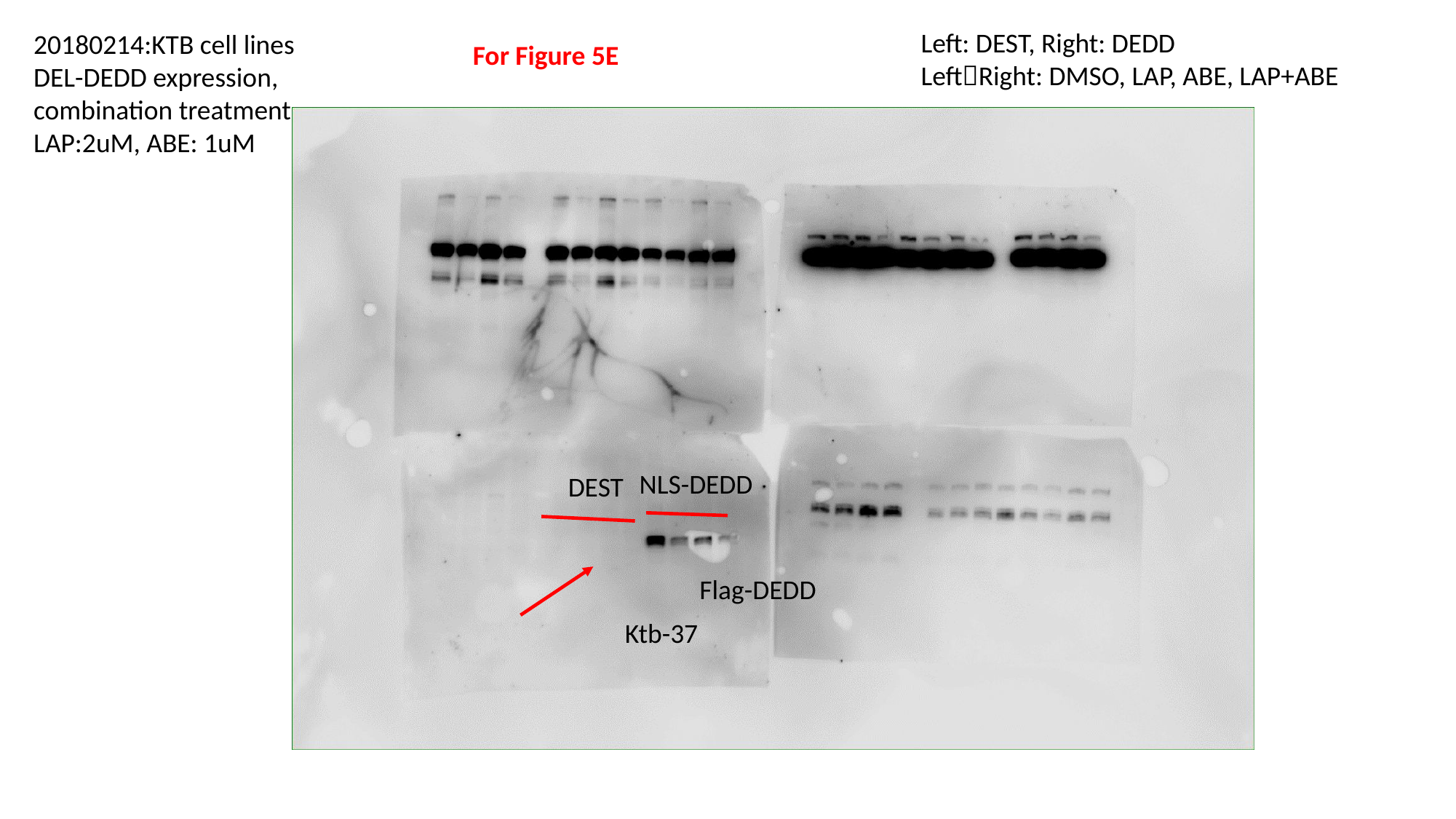

Left: DEST, Right: DEDD
LeftRight: DMSO, LAP, ABE, LAP+ABE
20180214:KTB cell lines DEL-DEDD expression, combination treatment
LAP:2uM, ABE: 1uM
For Figure 5E
NLS-DEDD
DEST
Flag-DEDD
Ktb-37

## Slide 77
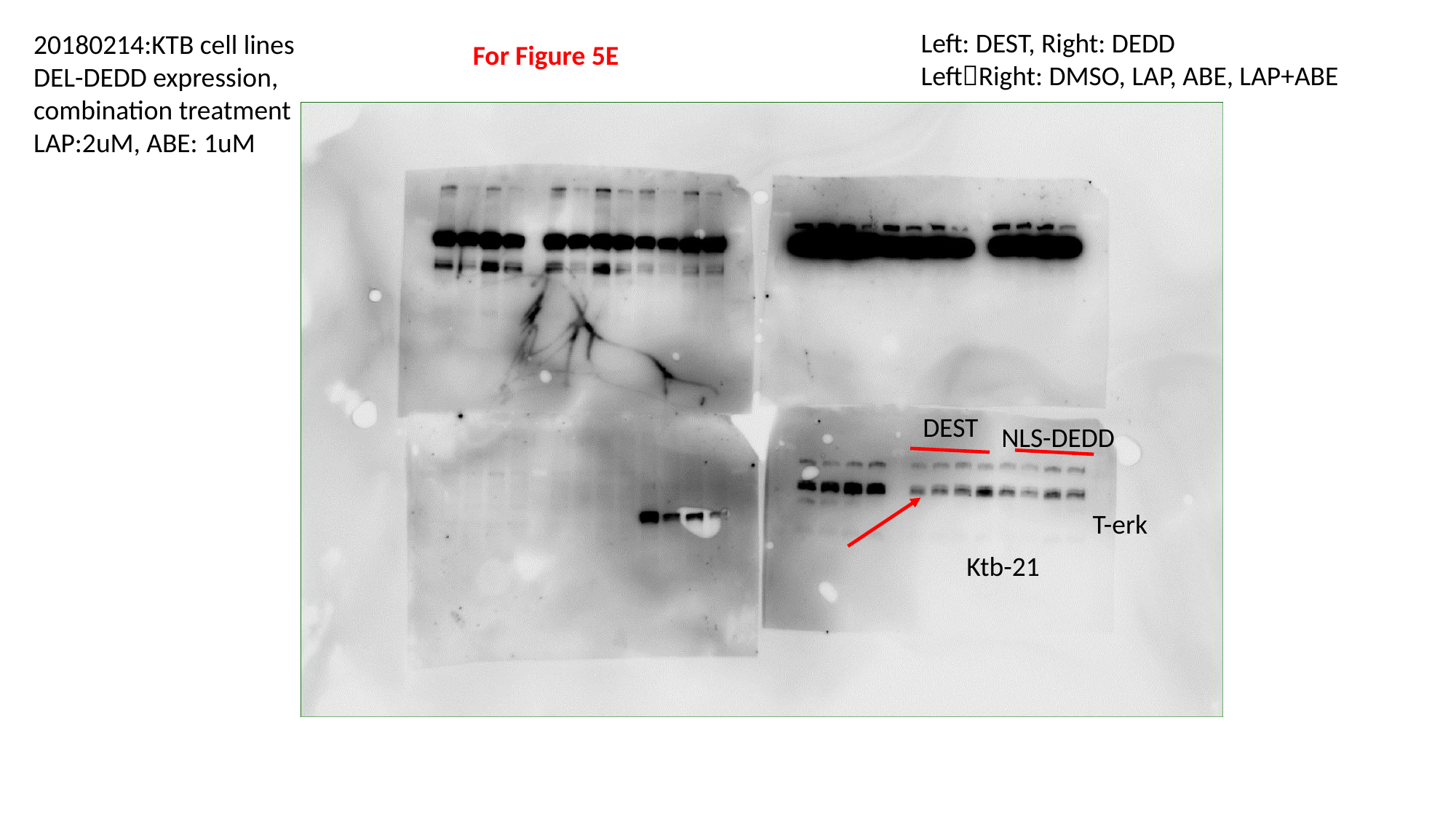

Left: DEST, Right: DEDD
LeftRight: DMSO, LAP, ABE, LAP+ABE
20180214:KTB cell lines DEL-DEDD expression, combination treatment
LAP:2uM, ABE: 1uM
For Figure 5E
DEST
NLS-DEDD
T-erk
Ktb-21

## Slide 78
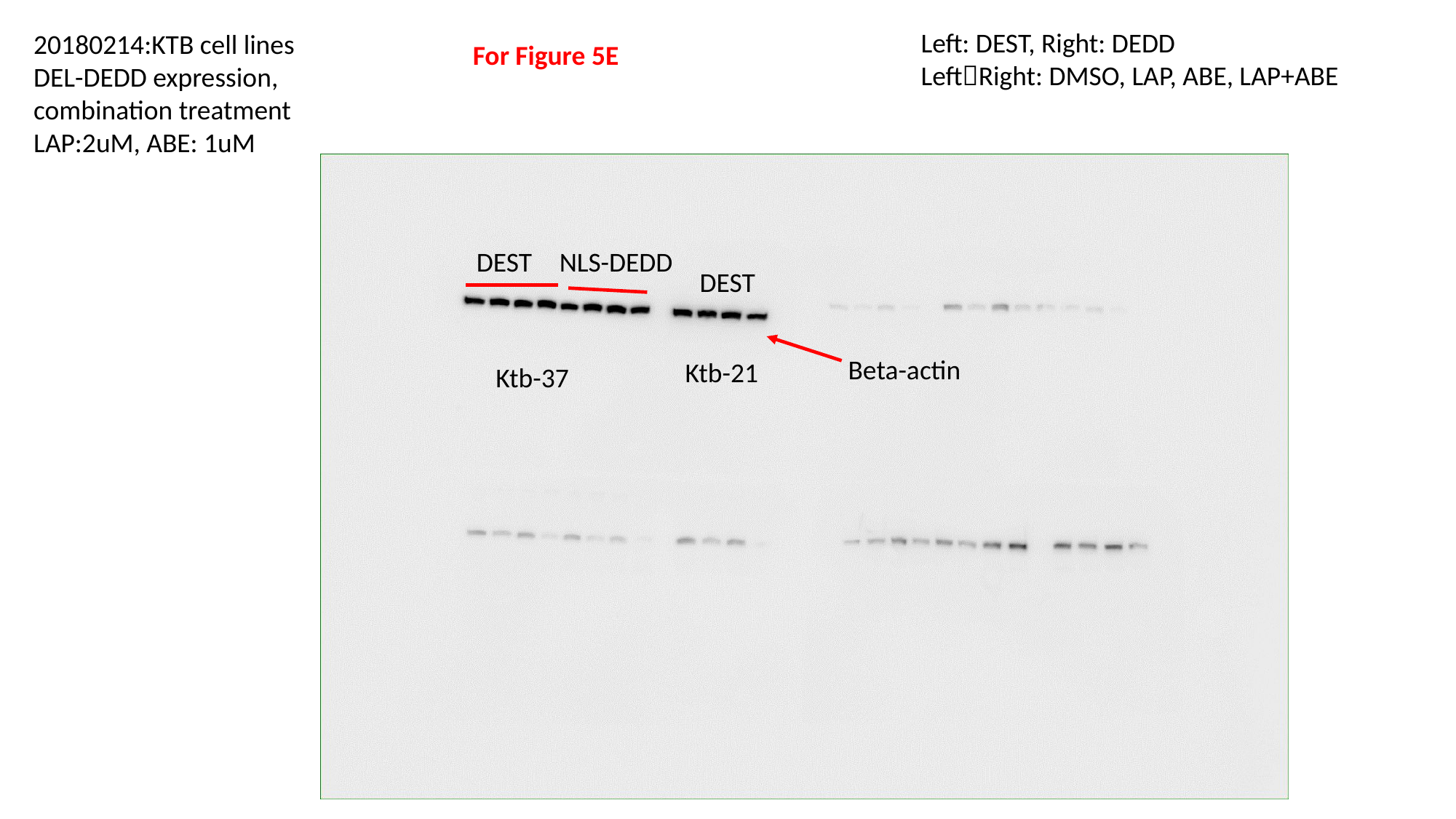

Left: DEST, Right: DEDD
LeftRight: DMSO, LAP, ABE, LAP+ABE
20180214:KTB cell lines DEL-DEDD expression, combination treatment
LAP:2uM, ABE: 1uM
For Figure 5E
DEST
NLS-DEDD
DEST
Beta-actin
Ktb-21
Ktb-37

## Slide 79
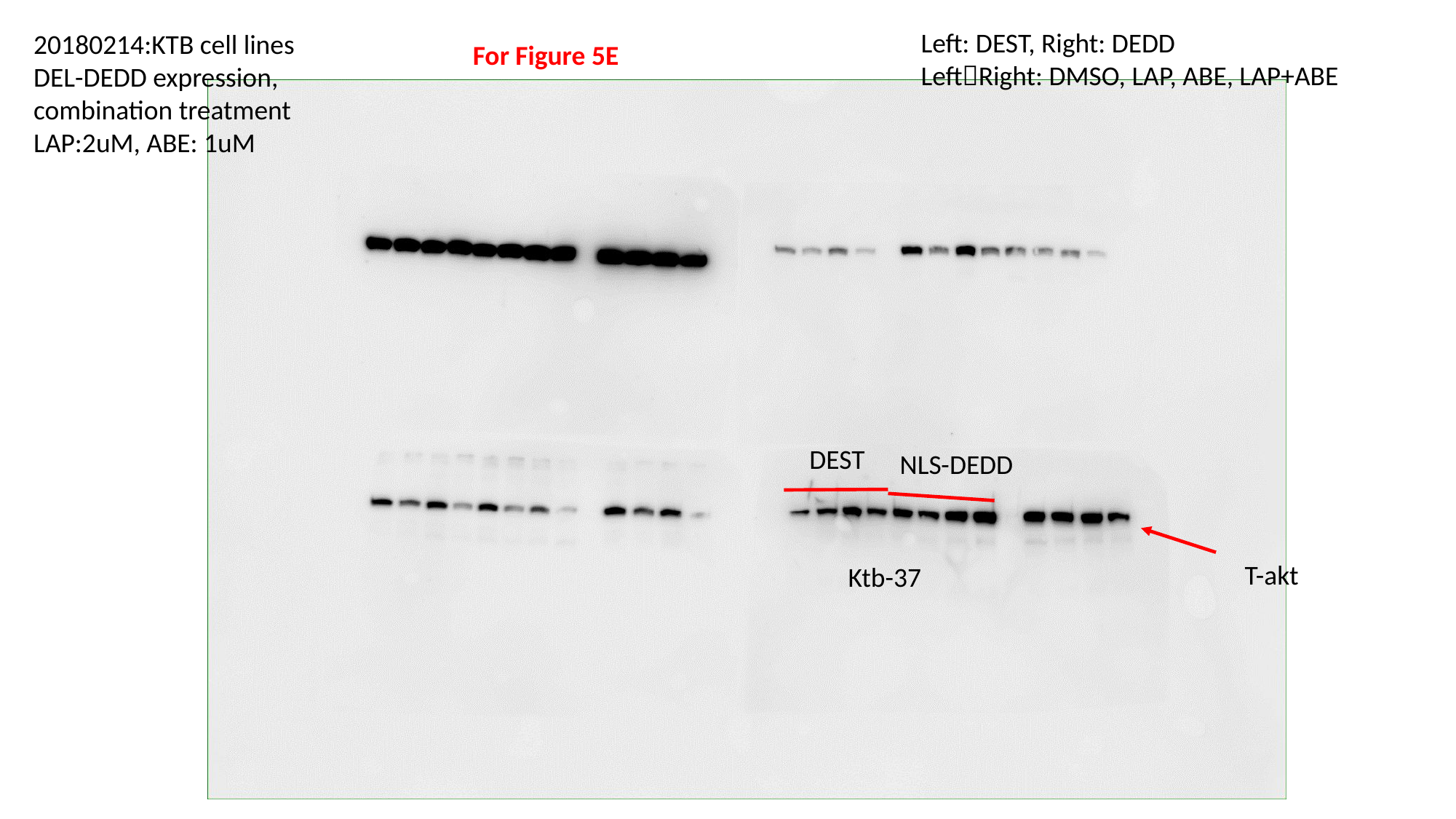

Left: DEST, Right: DEDD
LeftRight: DMSO, LAP, ABE, LAP+ABE
20180214:KTB cell lines DEL-DEDD expression, combination treatment
LAP:2uM, ABE: 1uM
For Figure 5E
DEST
NLS-DEDD
T-akt
Ktb-37

## Slide 80
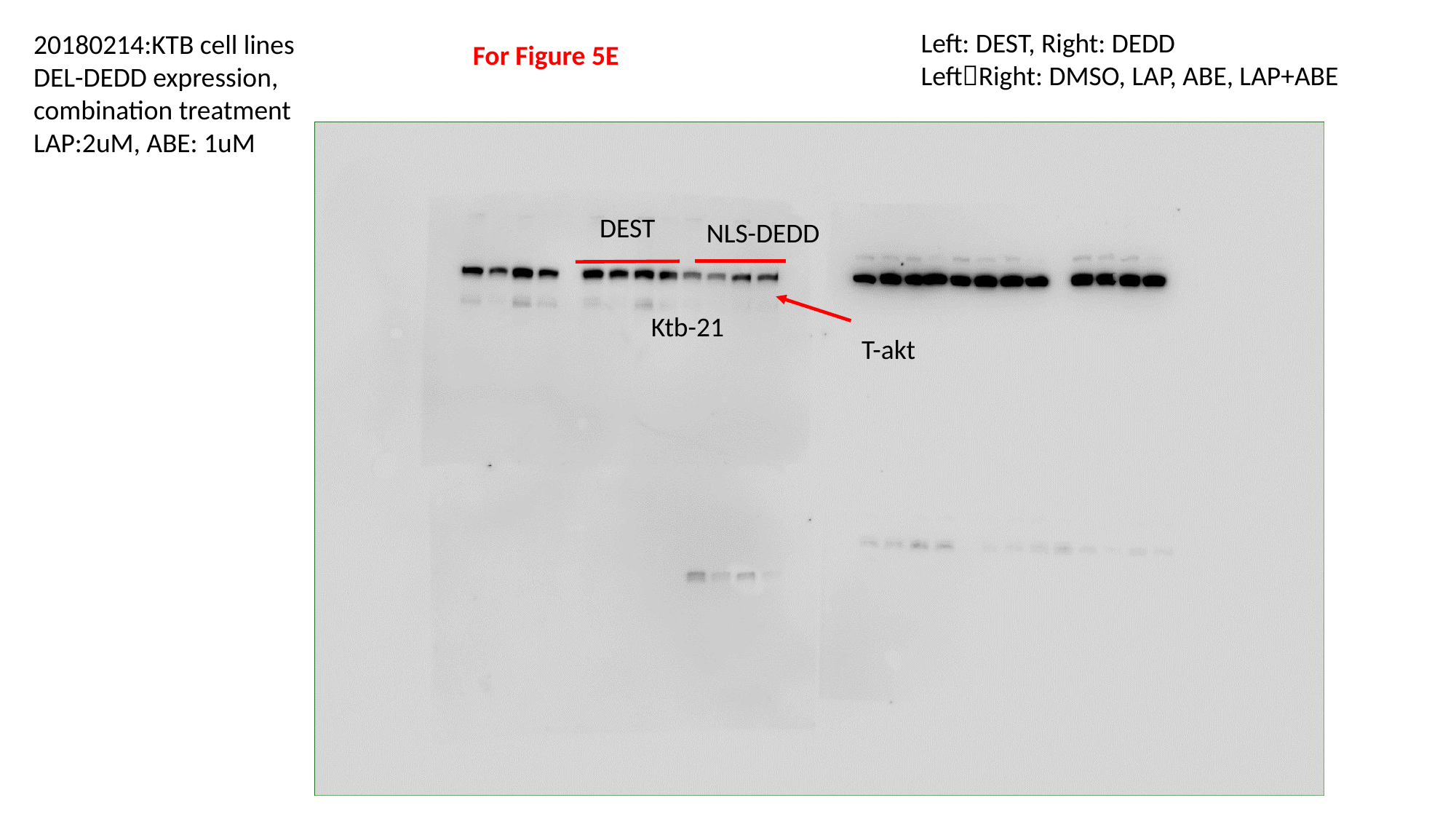

Left: DEST, Right: DEDD
LeftRight: DMSO, LAP, ABE, LAP+ABE
20180214:KTB cell lines DEL-DEDD expression, combination treatment
LAP:2uM, ABE: 1uM
For Figure 5E
DEST
NLS-DEDD
Ktb-21
T-akt

## Slide 81
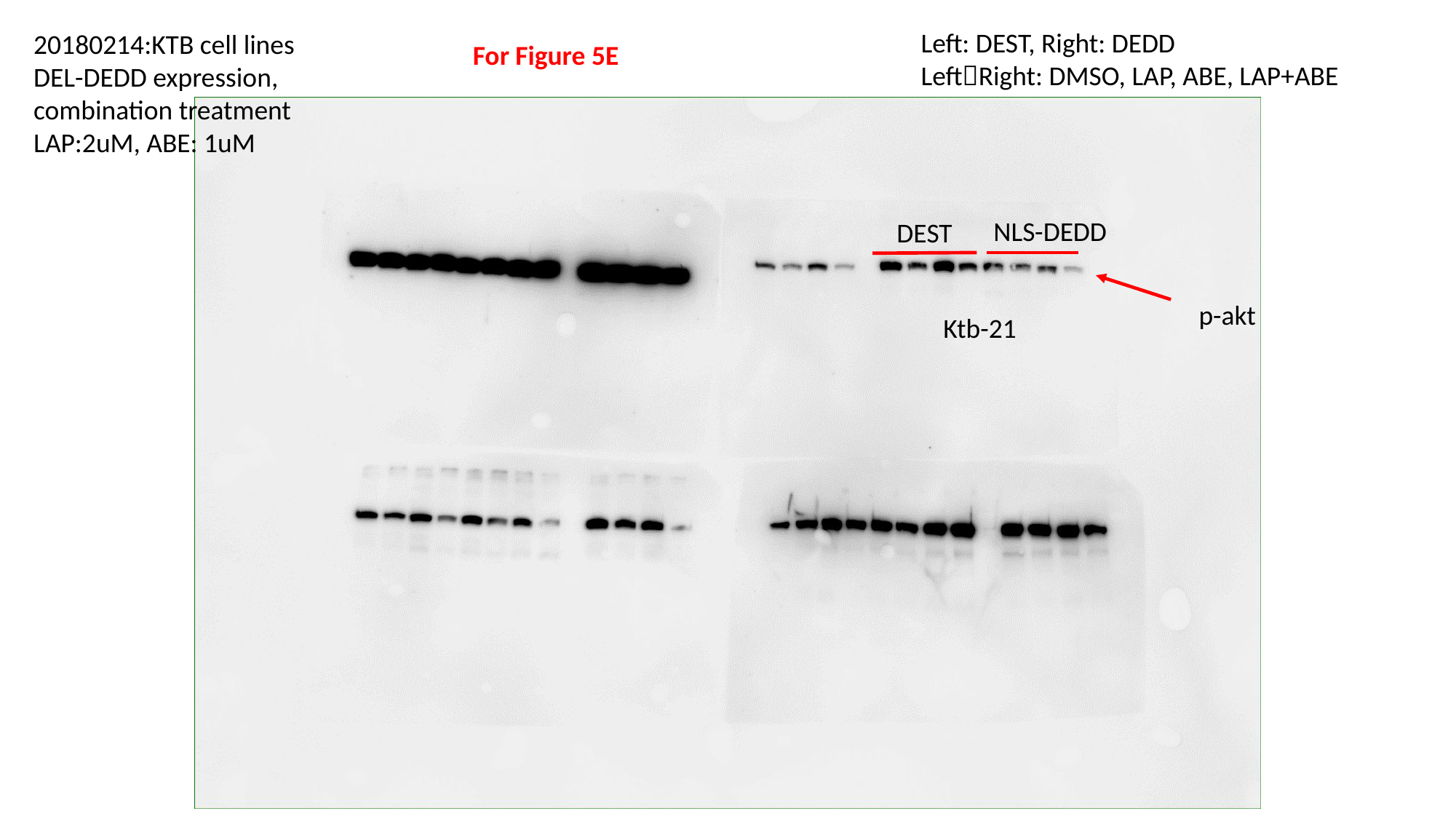

Left: DEST, Right: DEDD
LeftRight: DMSO, LAP, ABE, LAP+ABE
20180214:KTB cell lines DEL-DEDD expression, combination treatment
LAP:2uM, ABE: 1uM
For Figure 5E
NLS-DEDD
DEST
p-akt
Ktb-21

## Slide 82
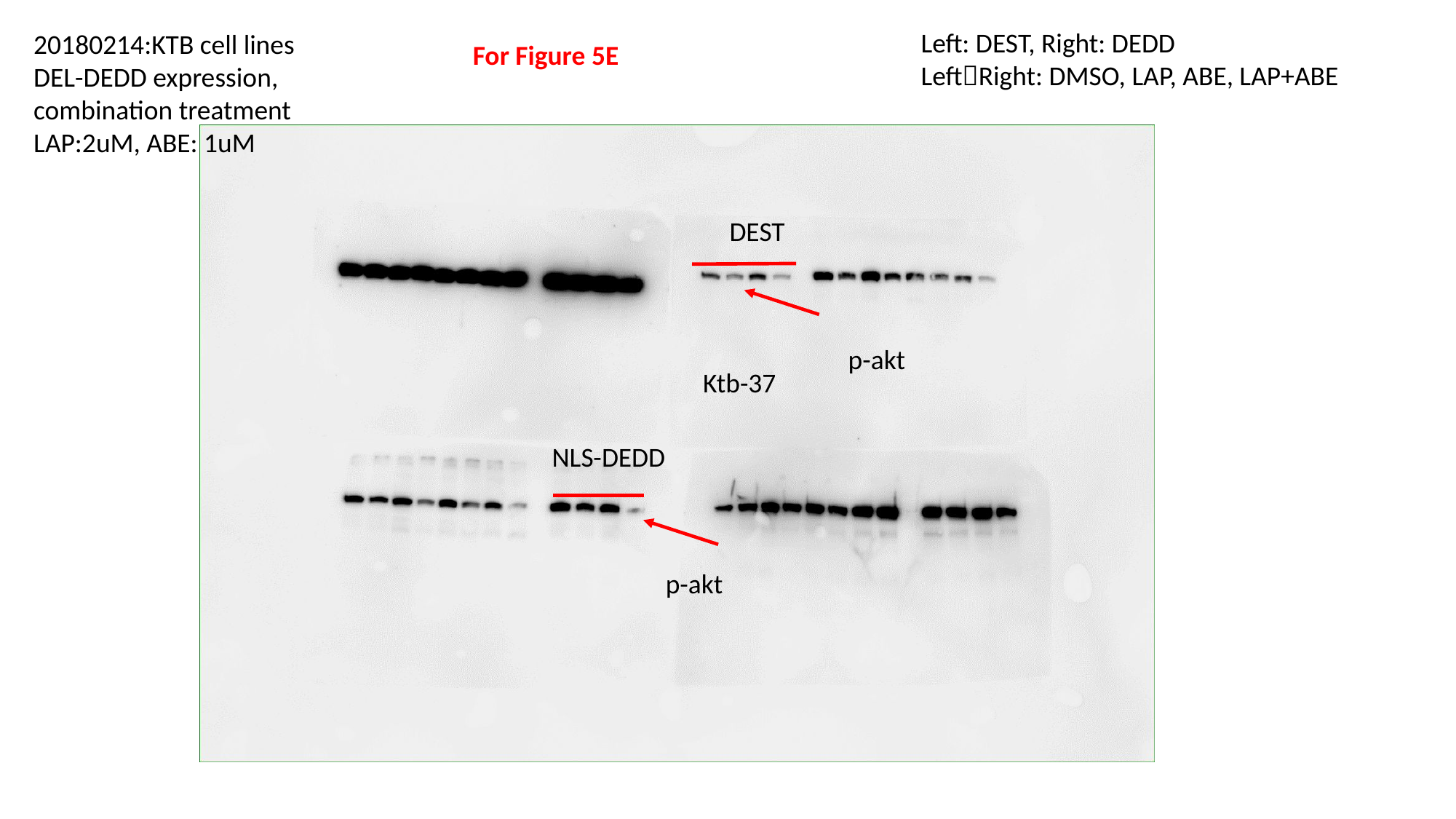

Left: DEST, Right: DEDD
LeftRight: DMSO, LAP, ABE, LAP+ABE
20180214:KTB cell lines DEL-DEDD expression, combination treatment
LAP:2uM, ABE: 1uM
For Figure 5E
DEST
p-akt
Ktb-37
NLS-DEDD
p-akt

## Slide 83
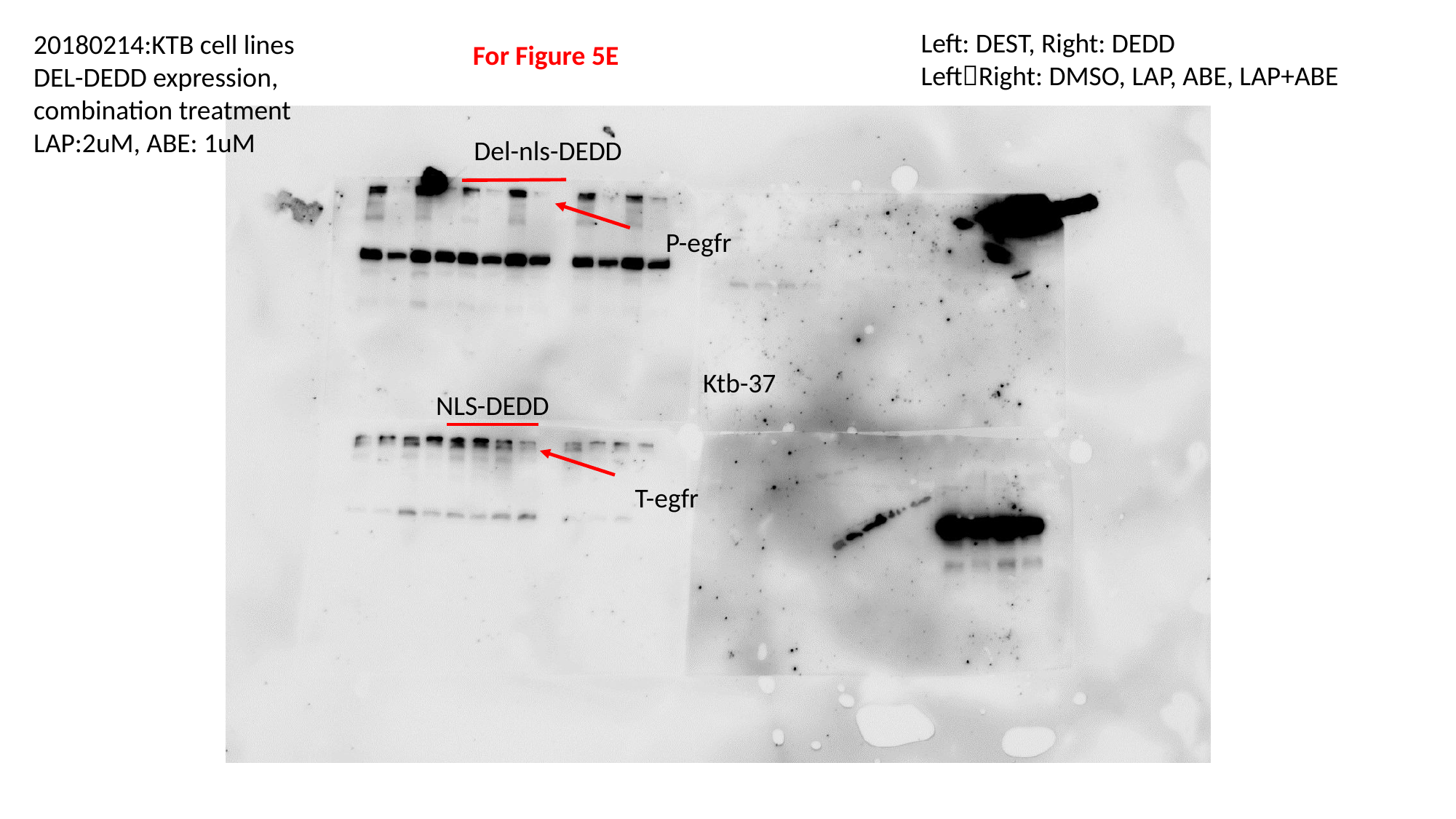

Left: DEST, Right: DEDD
LeftRight: DMSO, LAP, ABE, LAP+ABE
20180214:KTB cell lines DEL-DEDD expression, combination treatment
LAP:2uM, ABE: 1uM
For Figure 5E
Del-nls-DEDD
P-egfr
Ktb-37
NLS-DEDD
T-egfr

## Slide 84
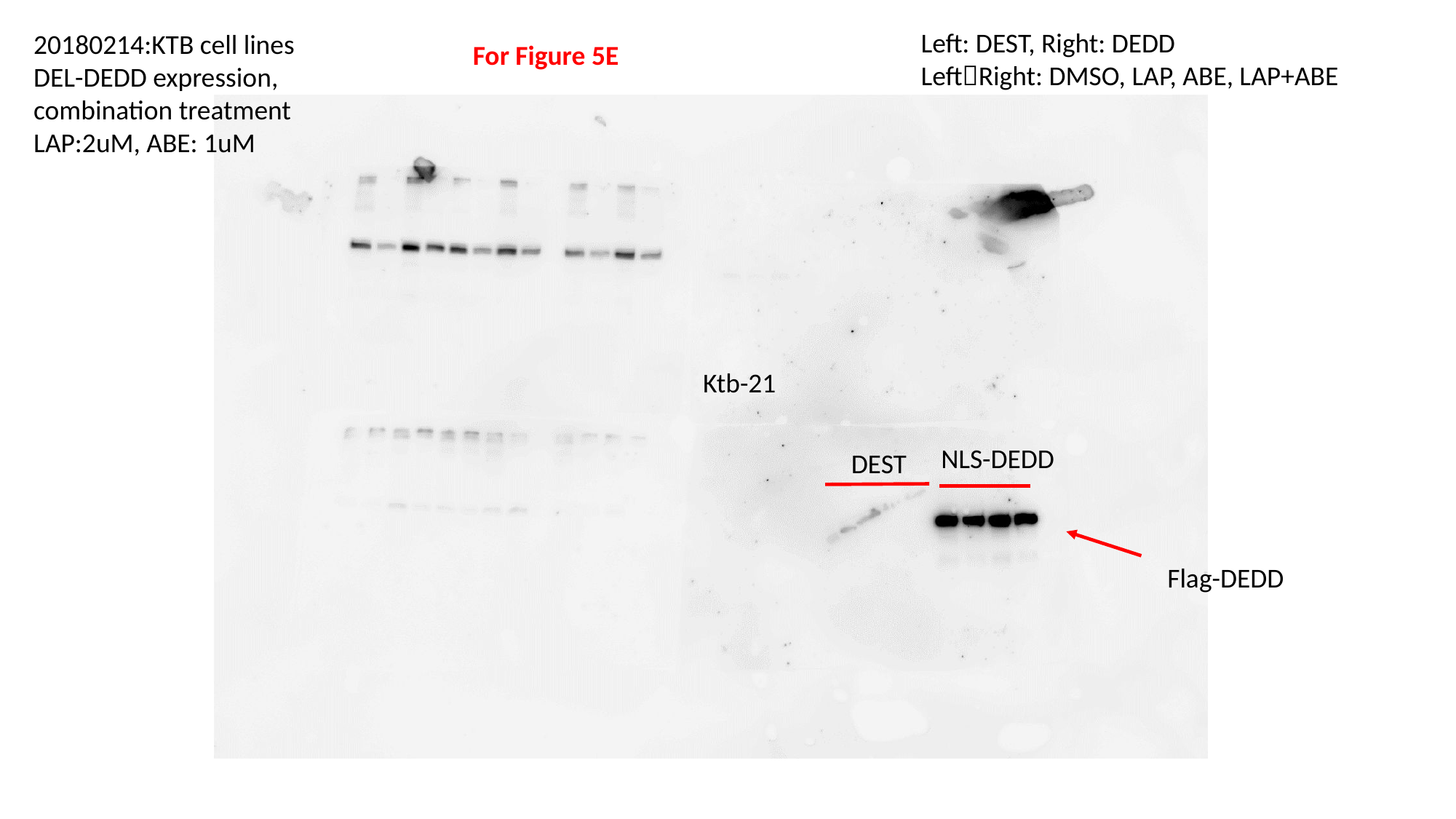

Left: DEST, Right: DEDD
LeftRight: DMSO, LAP, ABE, LAP+ABE
20180214:KTB cell lines DEL-DEDD expression, combination treatment
LAP:2uM, ABE: 1uM
For Figure 5E
Ktb-21
NLS-DEDD
DEST
Flag-DEDD

## Slide 85
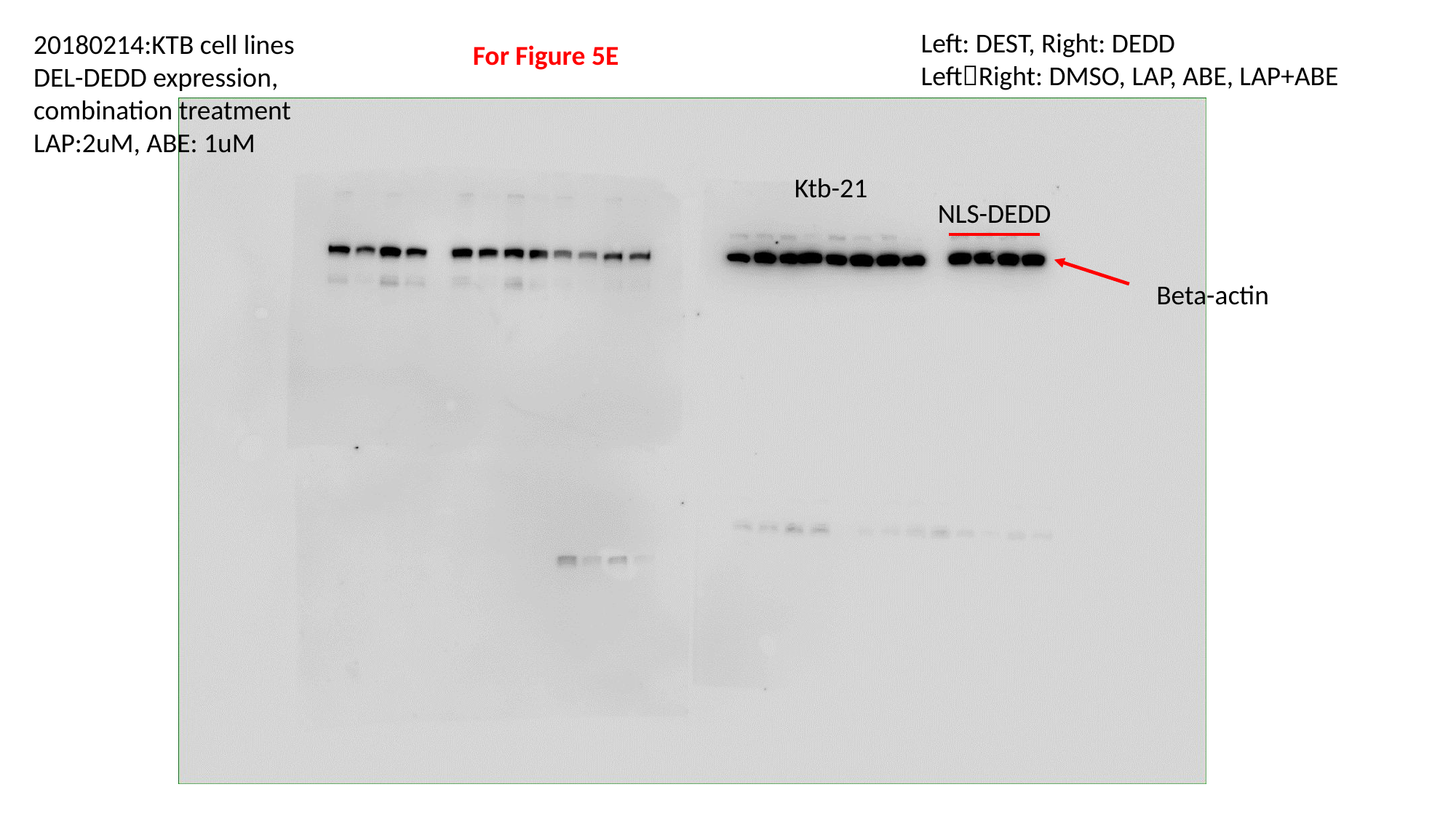

Left: DEST, Right: DEDD
LeftRight: DMSO, LAP, ABE, LAP+ABE
20180214:KTB cell lines DEL-DEDD expression, combination treatment
LAP:2uM, ABE: 1uM
For Figure 5E
Ktb-21
NLS-DEDD
Beta-actin

## Slide 86
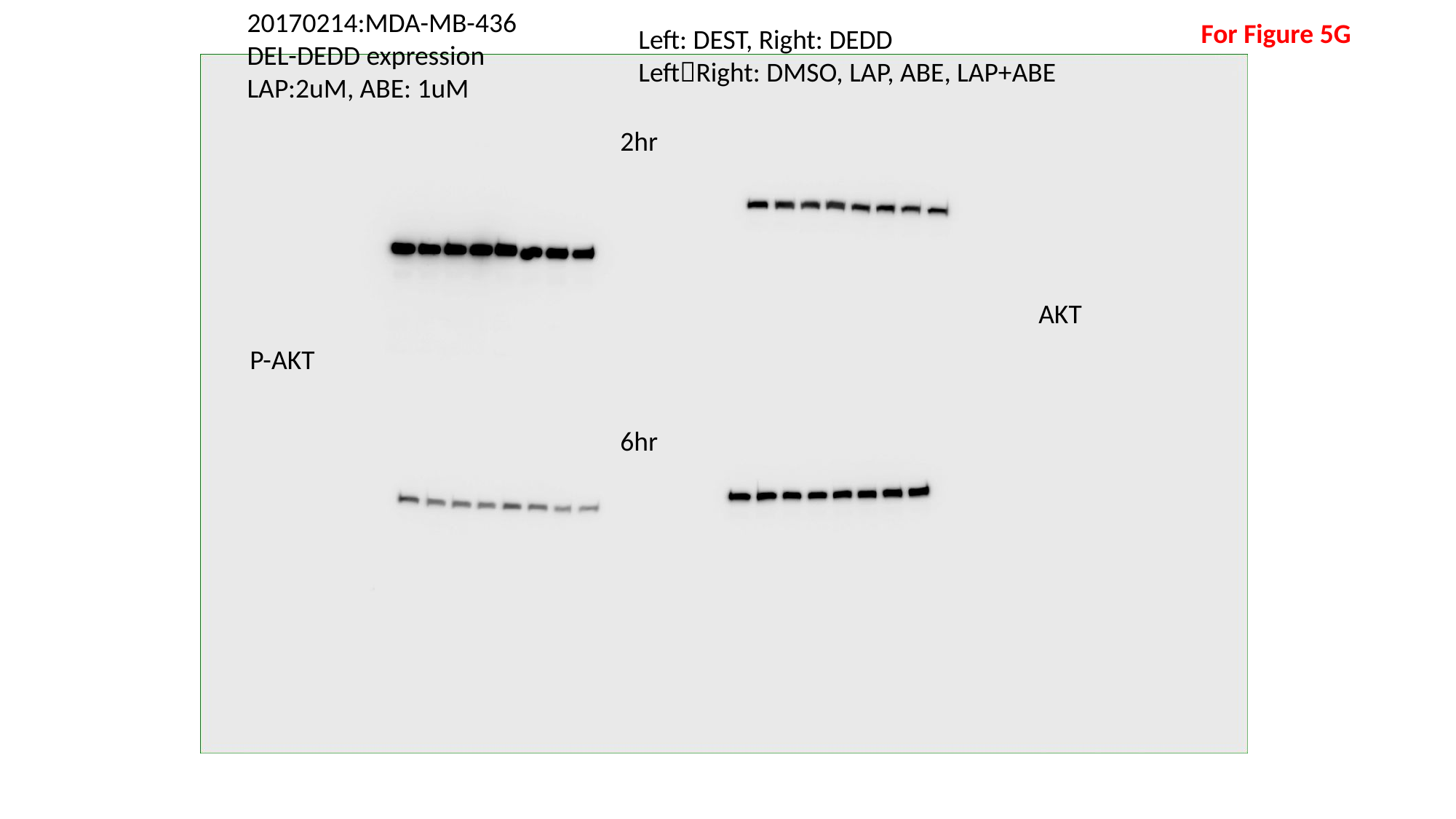

20170214:MDA-MB-436 DEL-DEDD expression
LAP:2uM, ABE: 1uM
For Figure 5G
Left: DEST, Right: DEDD
LeftRight: DMSO, LAP, ABE, LAP+ABE
2hr
#
AKT
P-AKT
6hr

## Slide 87
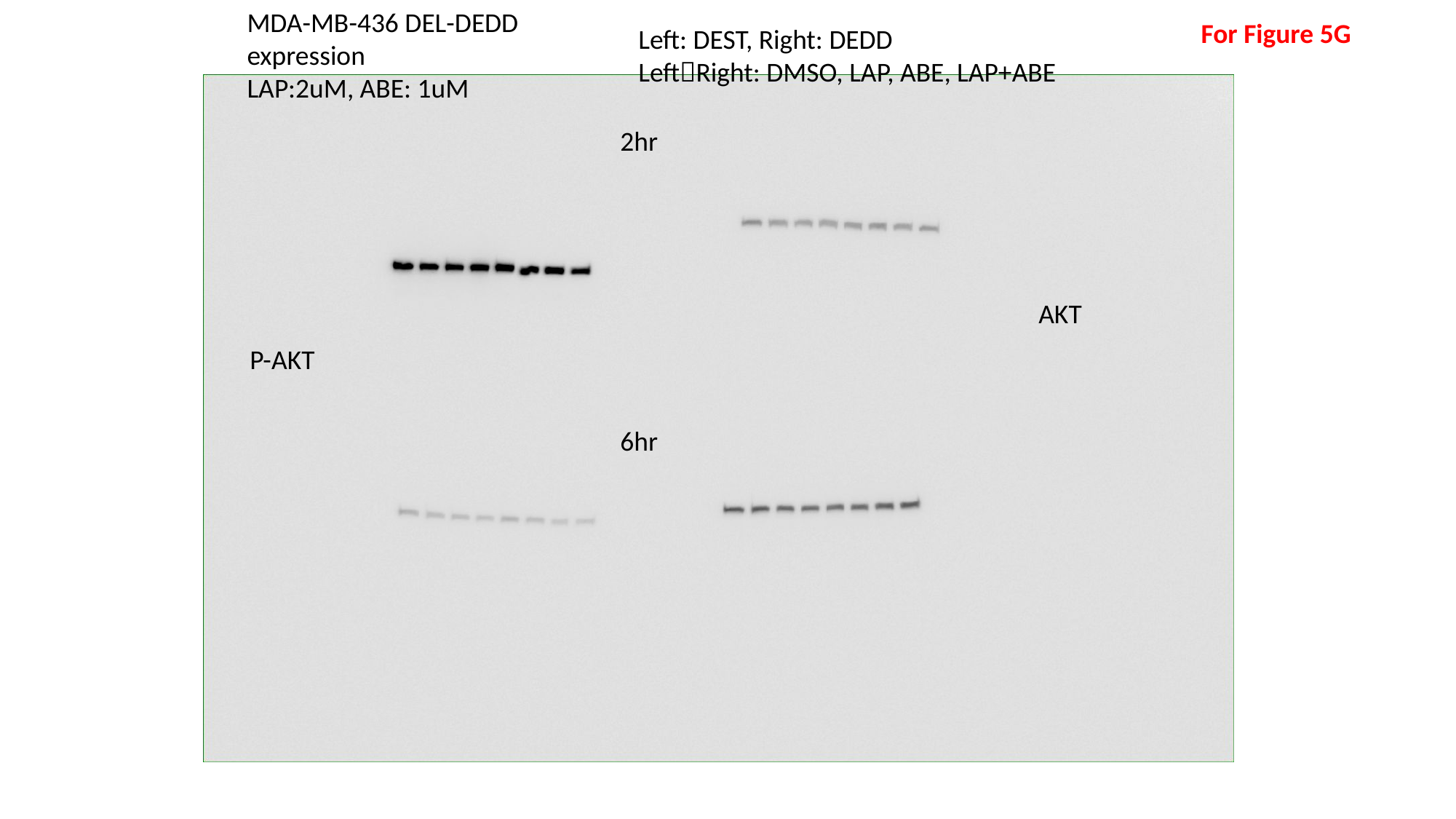

MDA-MB-436 DEL-DEDD expression
LAP:2uM, ABE: 1uM
For Figure 5G
Left: DEST, Right: DEDD
LeftRight: DMSO, LAP, ABE, LAP+ABE
2hr
AKT
P-AKT
6hr

## Slide 88
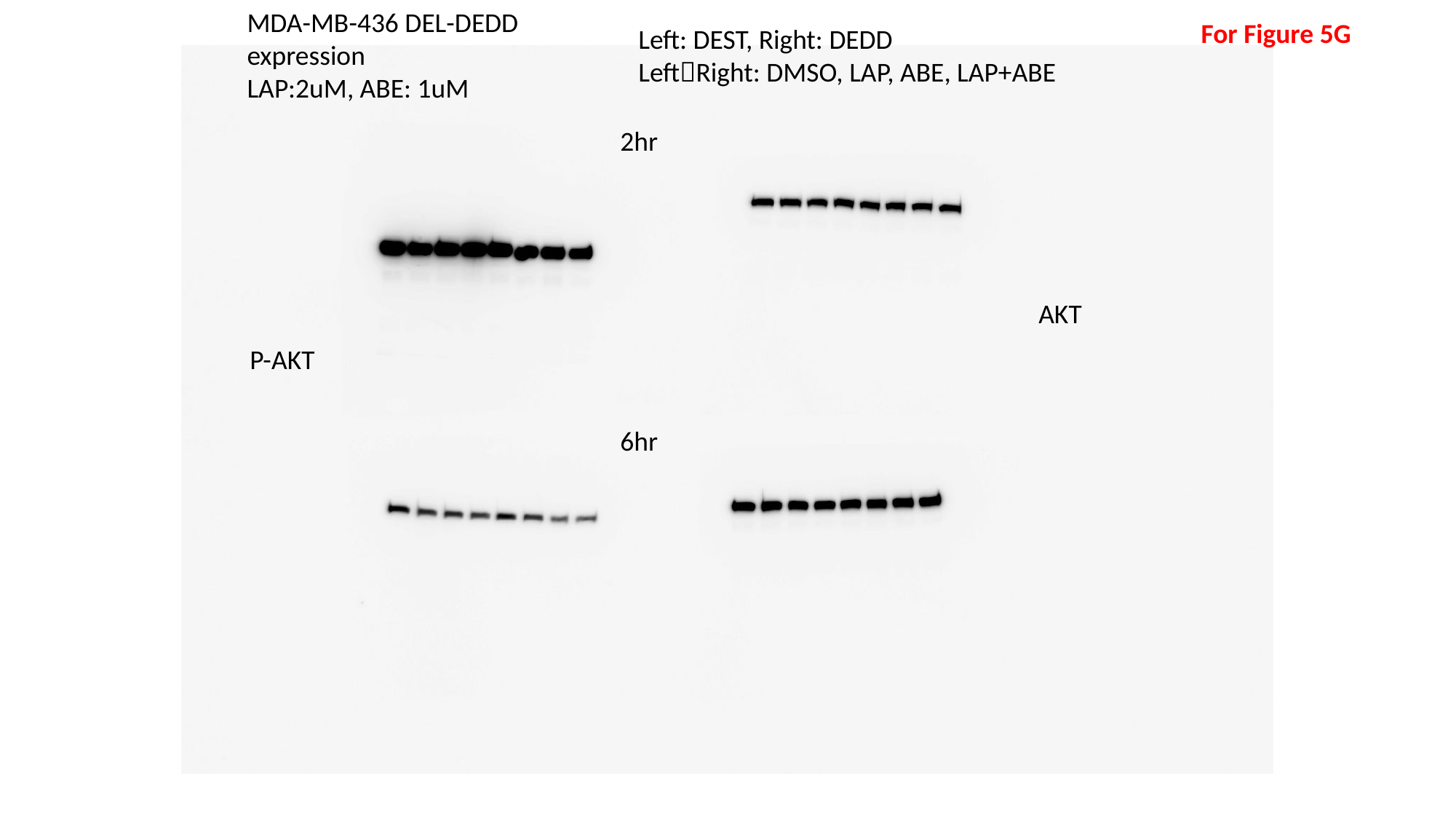

MDA-MB-436 DEL-DEDD expression
LAP:2uM, ABE: 1uM
For Figure 5G
Left: DEST, Right: DEDD
LeftRight: DMSO, LAP, ABE, LAP+ABE
2hr
AKT
P-AKT
6hr

## Slide 89
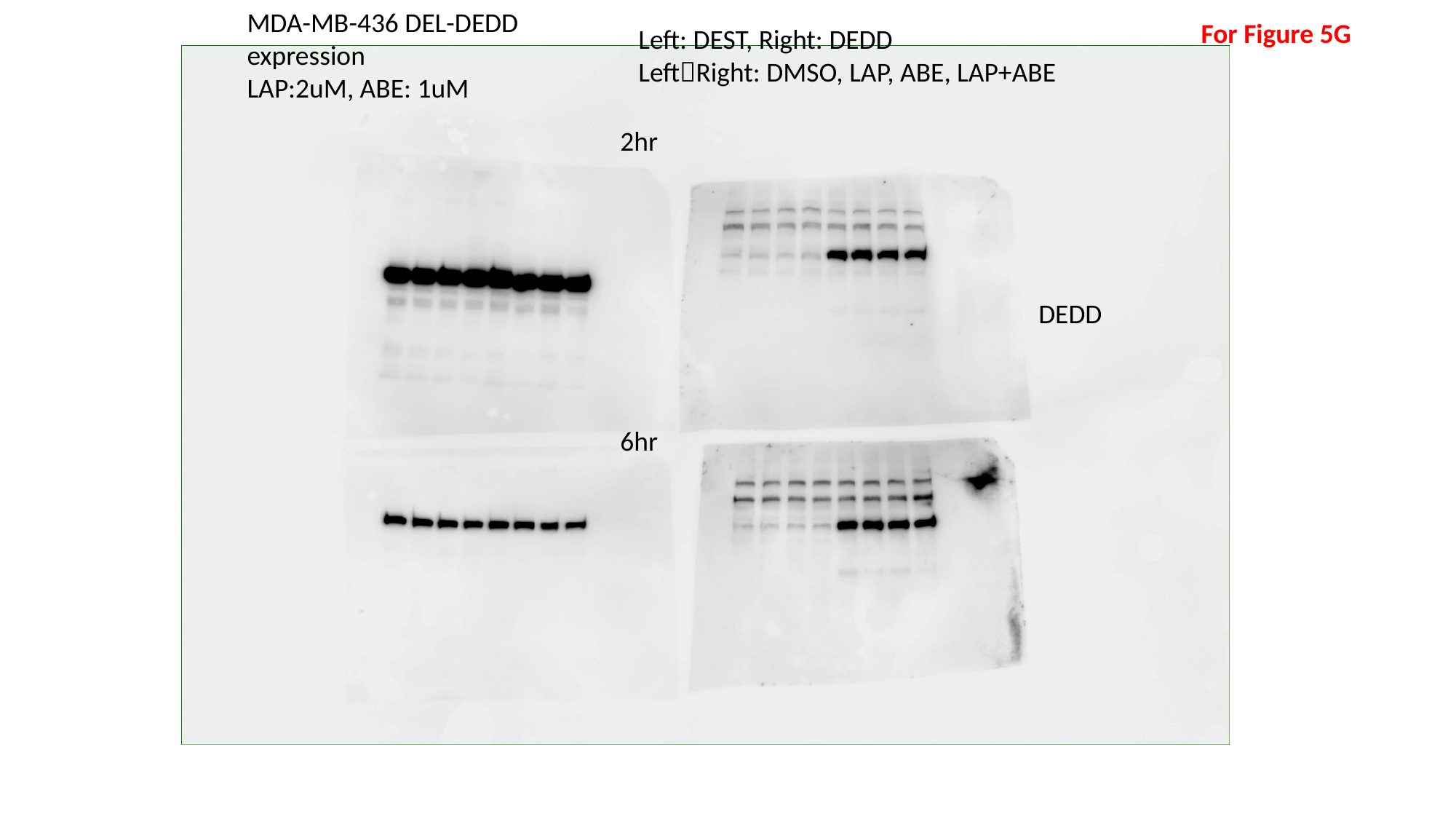

MDA-MB-436 DEL-DEDD expression
LAP:2uM, ABE: 1uM
For Figure 5G
Left: DEST, Right: DEDD
LeftRight: DMSO, LAP, ABE, LAP+ABE
2hr
DEDD
6hr

## Slide 90
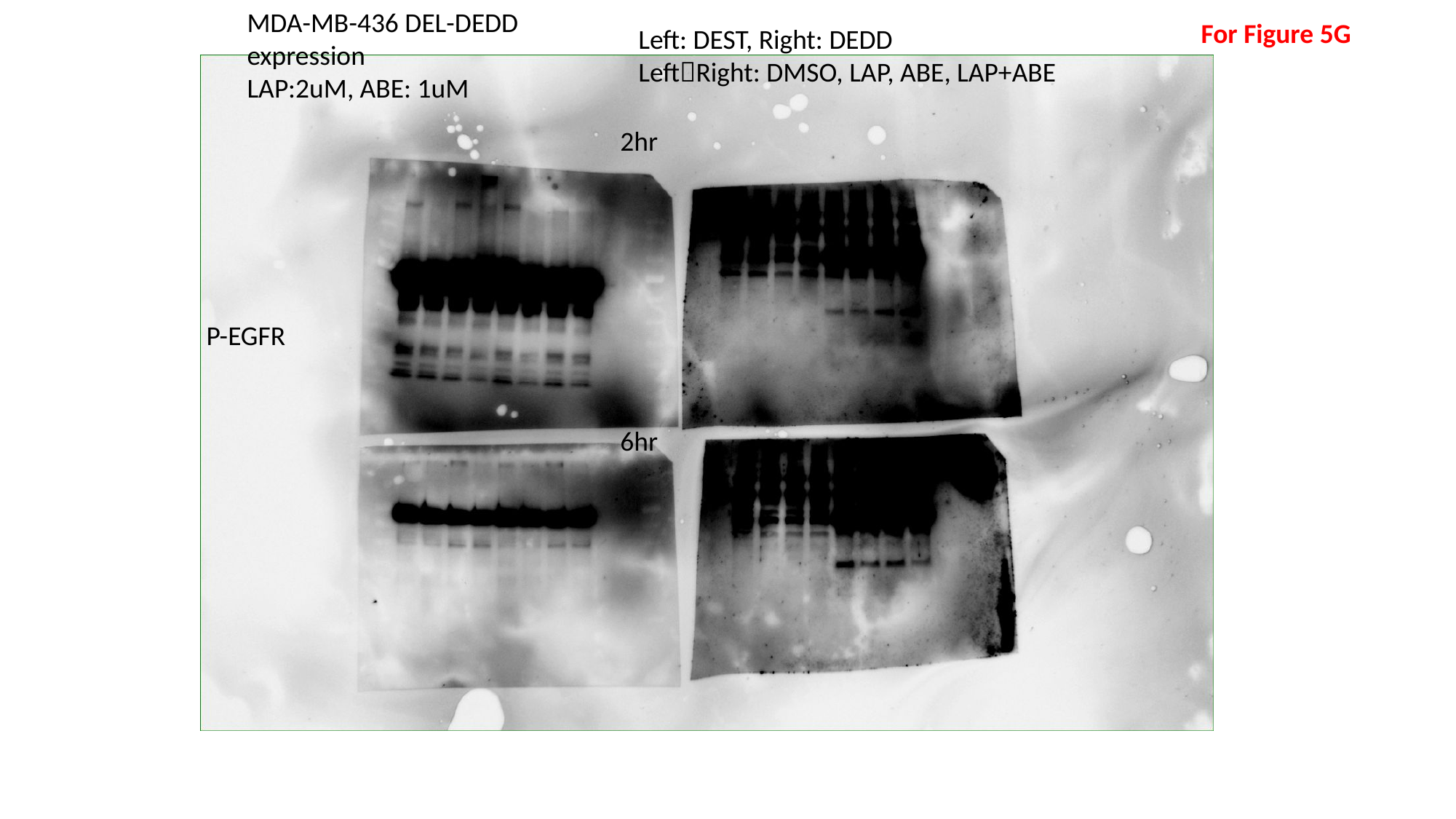

MDA-MB-436 DEL-DEDD expression
LAP:2uM, ABE: 1uM
For Figure 5G
Left: DEST, Right: DEDD
LeftRight: DMSO, LAP, ABE, LAP+ABE
2hr
P-EGFR
6hr

## Slide 91
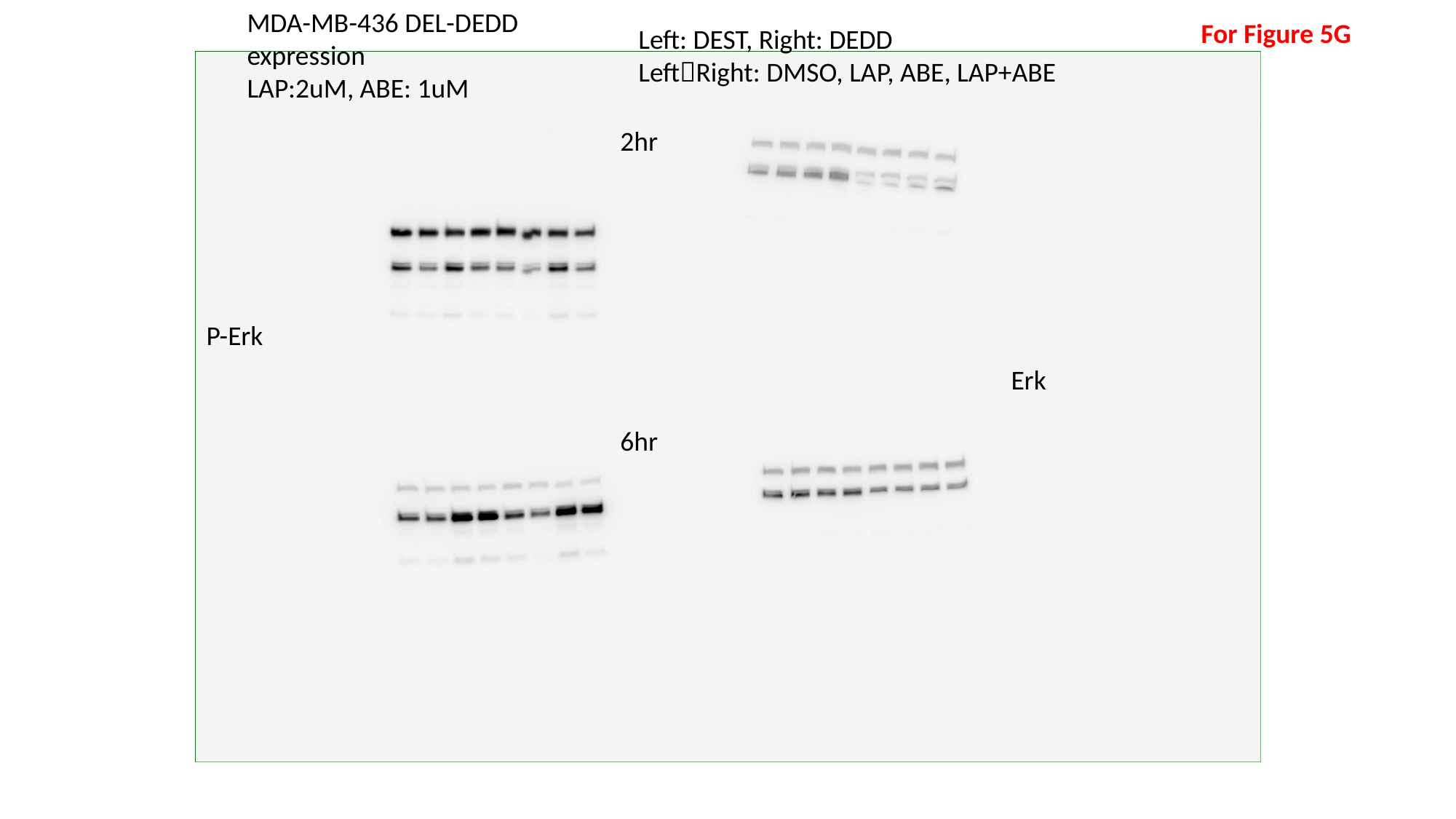

MDA-MB-436 DEL-DEDD expression
LAP:2uM, ABE: 1uM
For Figure 5G
Left: DEST, Right: DEDD
LeftRight: DMSO, LAP, ABE, LAP+ABE
2hr
P-Erk
Erk
6hr

## Slide 92
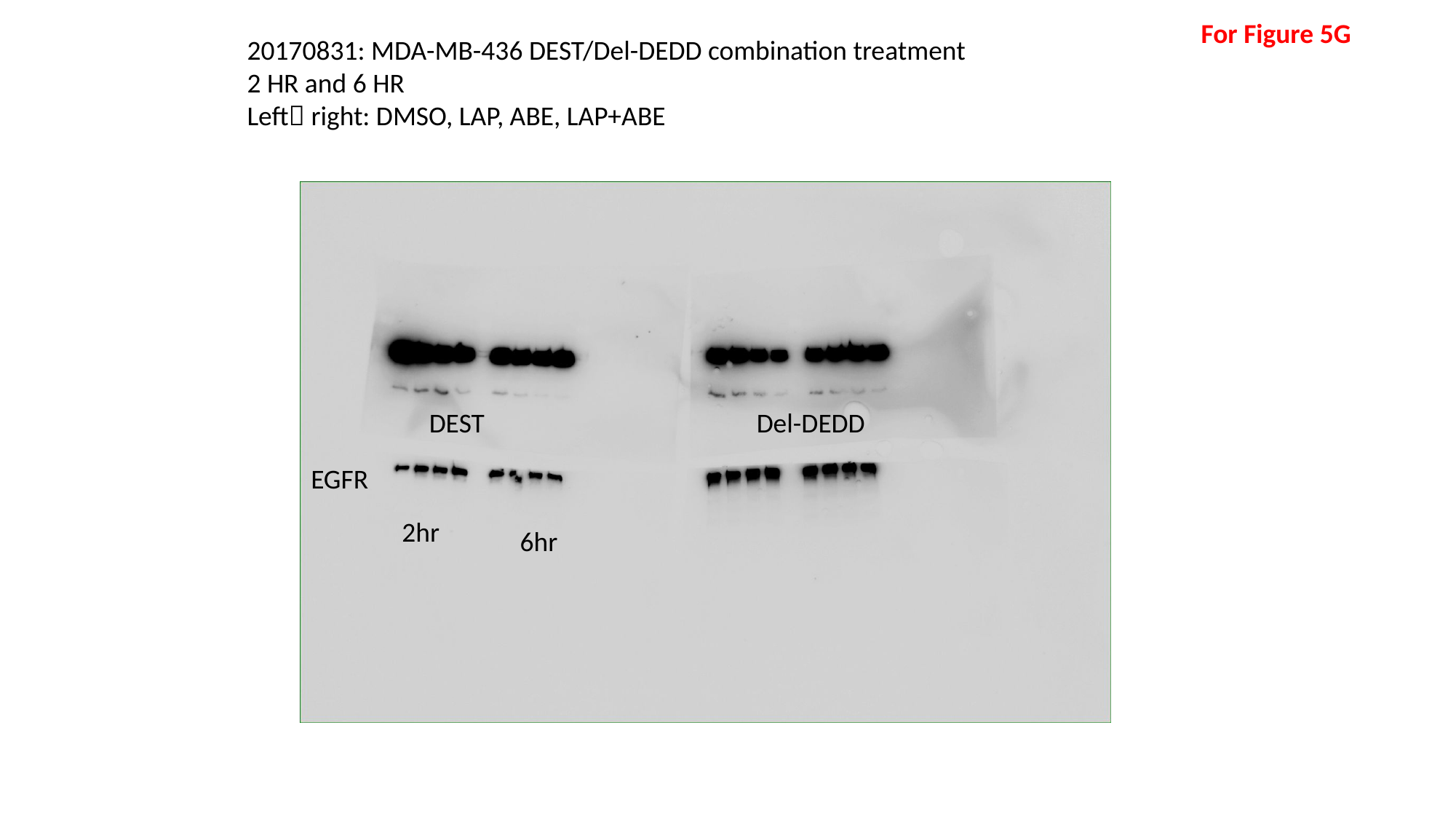

For Figure 5G
20170831: MDA-MB-436 DEST/Del-DEDD combination treatment
2 HR and 6 HR
Left right: DMSO, LAP, ABE, LAP+ABE
DEST
Del-DEDD
EGFR
2hr
6hr

## Slide 93
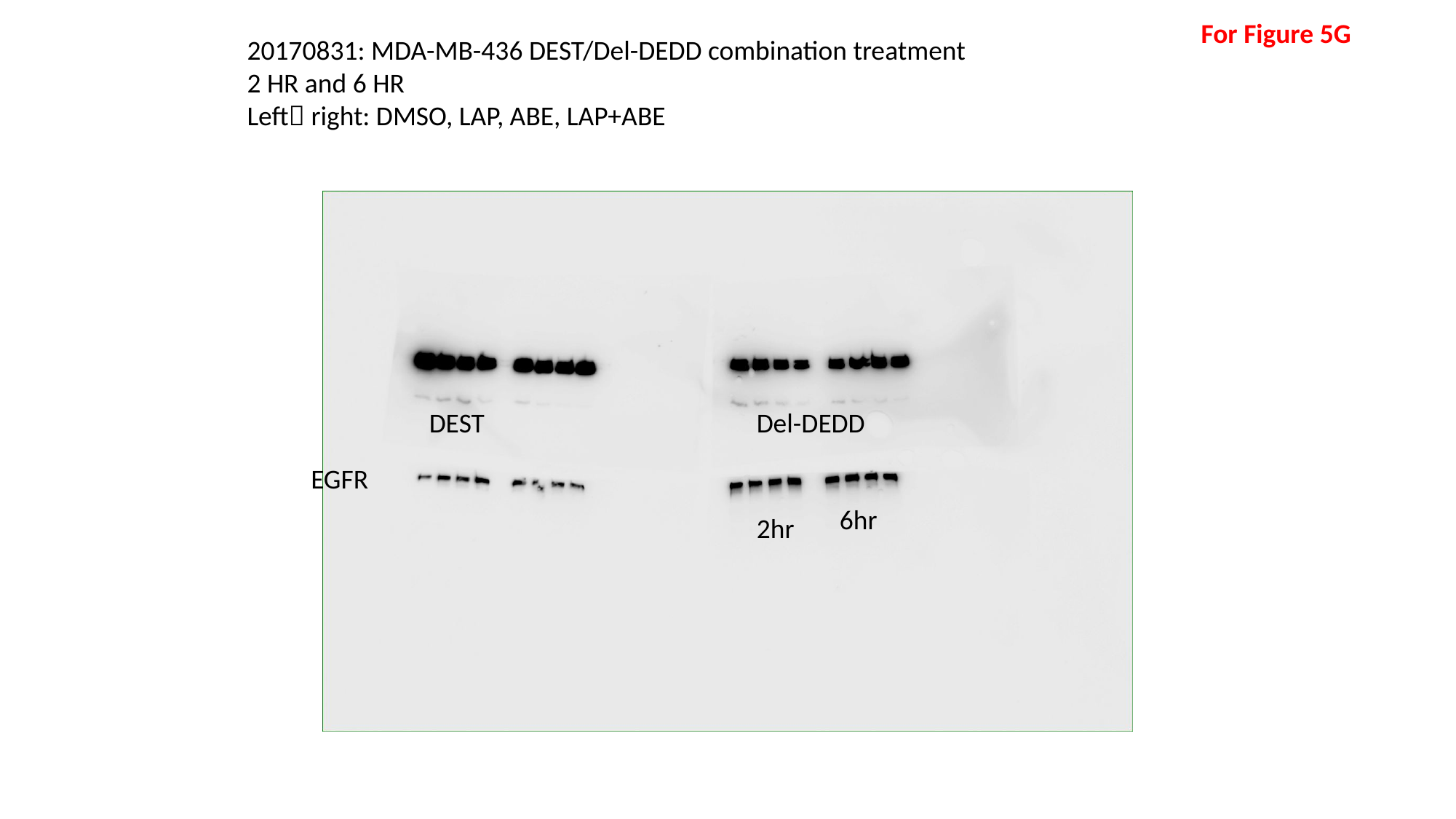

For Figure 5G
20170831: MDA-MB-436 DEST/Del-DEDD combination treatment
2 HR and 6 HR
Left right: DMSO, LAP, ABE, LAP+ABE
DEST
Del-DEDD
EGFR
6hr
2hr

## Slide 94
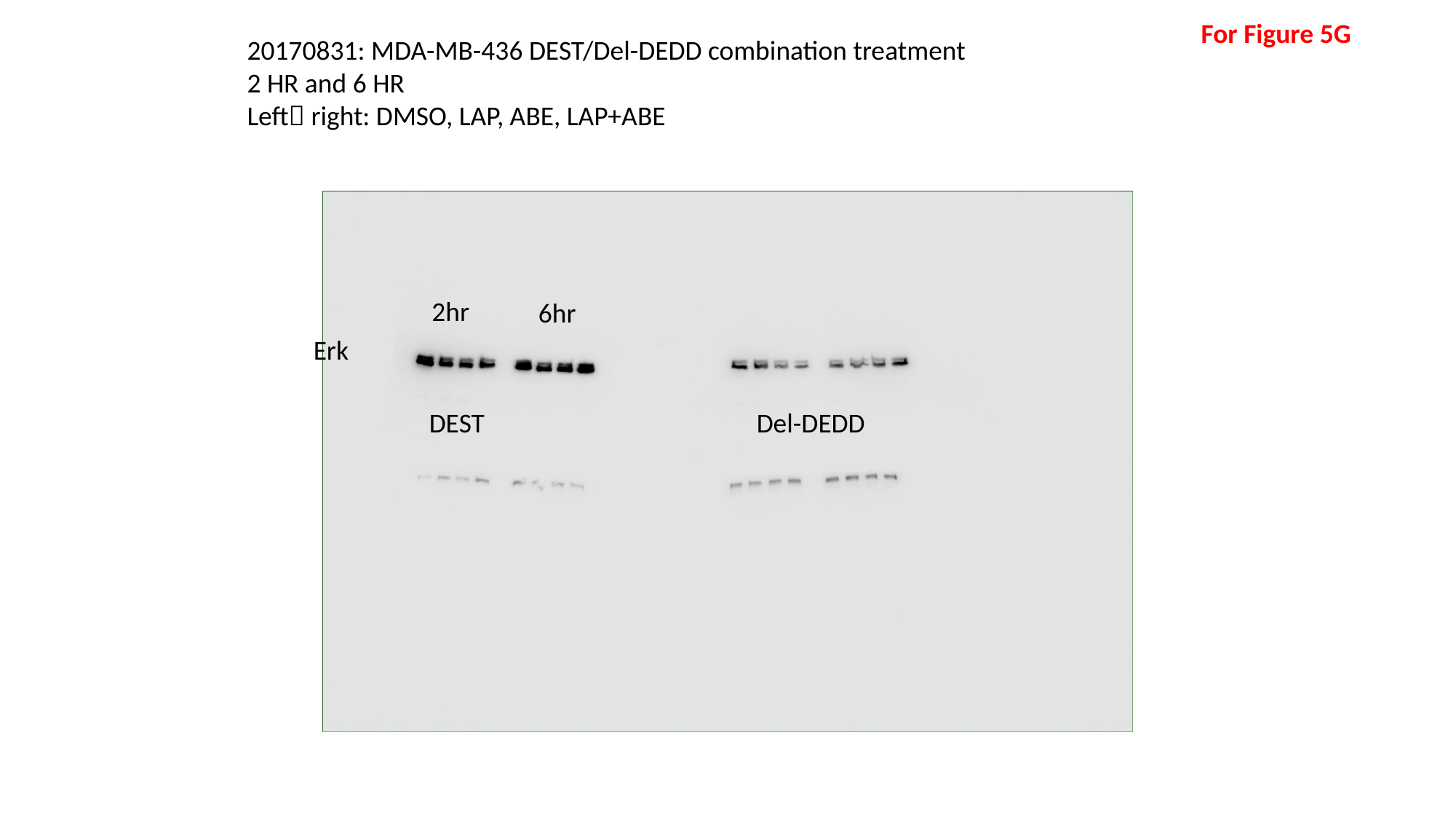

For Figure 5G
20170831: MDA-MB-436 DEST/Del-DEDD combination treatment
2 HR and 6 HR
Left right: DMSO, LAP, ABE, LAP+ABE
2hr
6hr
Erk
DEST
Del-DEDD

## Slide 95
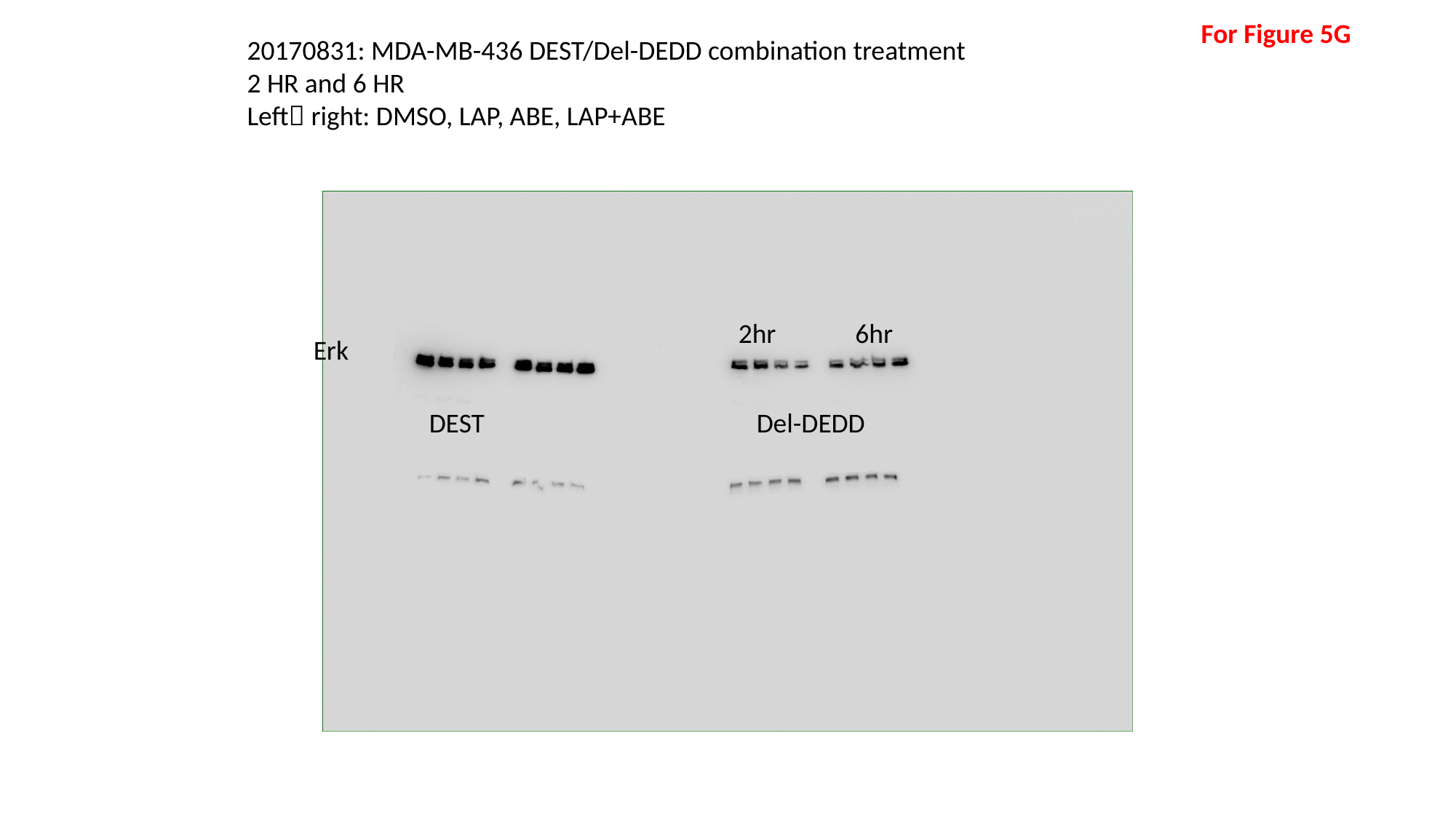

For Figure 5G
20170831: MDA-MB-436 DEST/Del-DEDD combination treatment
2 HR and 6 HR
Left right: DMSO, LAP, ABE, LAP+ABE
2hr
6hr
Erk
DEST
Del-DEDD

## Slide 96
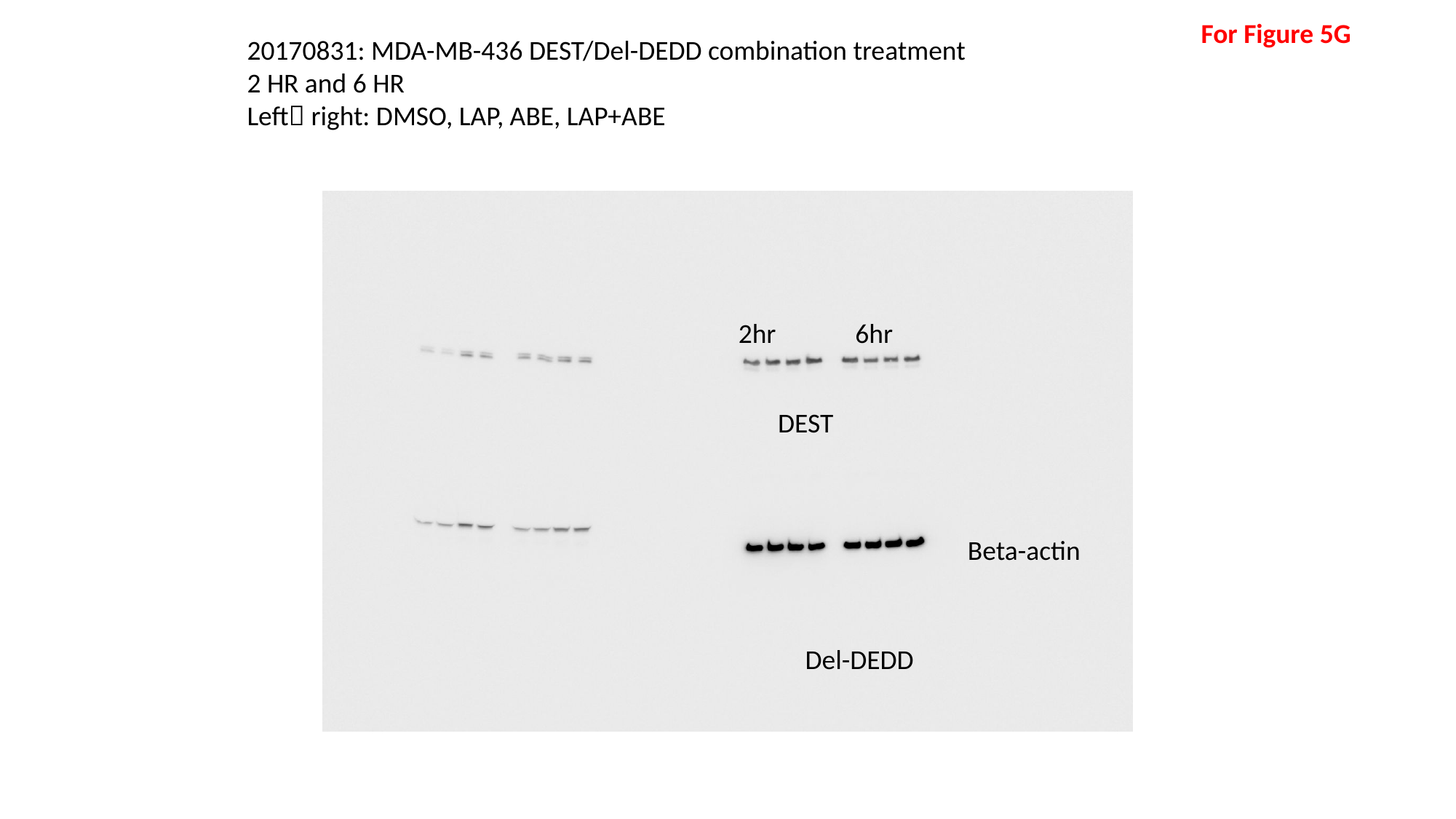

For Figure 5G
20170831: MDA-MB-436 DEST/Del-DEDD combination treatment
2 HR and 6 HR
Left right: DMSO, LAP, ABE, LAP+ABE
2hr
6hr
DEST
Beta-actin
Del-DEDD

## Slide 97
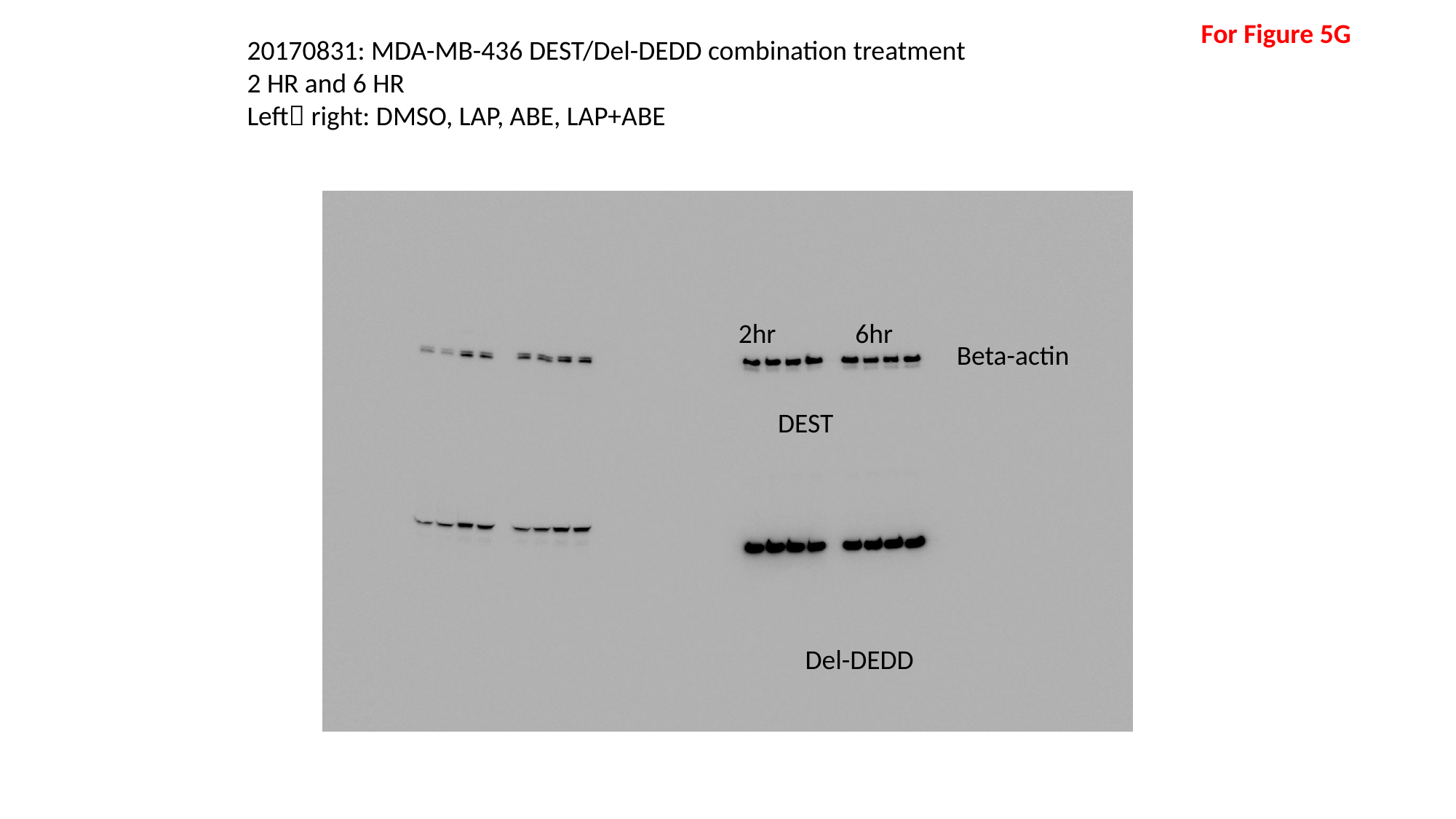

For Figure 5G
20170831: MDA-MB-436 DEST/Del-DEDD combination treatment
2 HR and 6 HR
Left right: DMSO, LAP, ABE, LAP+ABE
2hr
6hr
Beta-actin
DEST
Del-DEDD
